# Supplementary figures and images for: Synergistic Effects of Lenvatinib (E7080) and MEK Inhibitors against Anaplastic Thyroid Cancer in Preclinical Models (part 2 of 2)
Source: Cancers (Basel). 2021 Feb 18;13(4):862. doi: 10.3390/cancers13040862 (PMC7922355; doi:10.3390/cancers13040862)

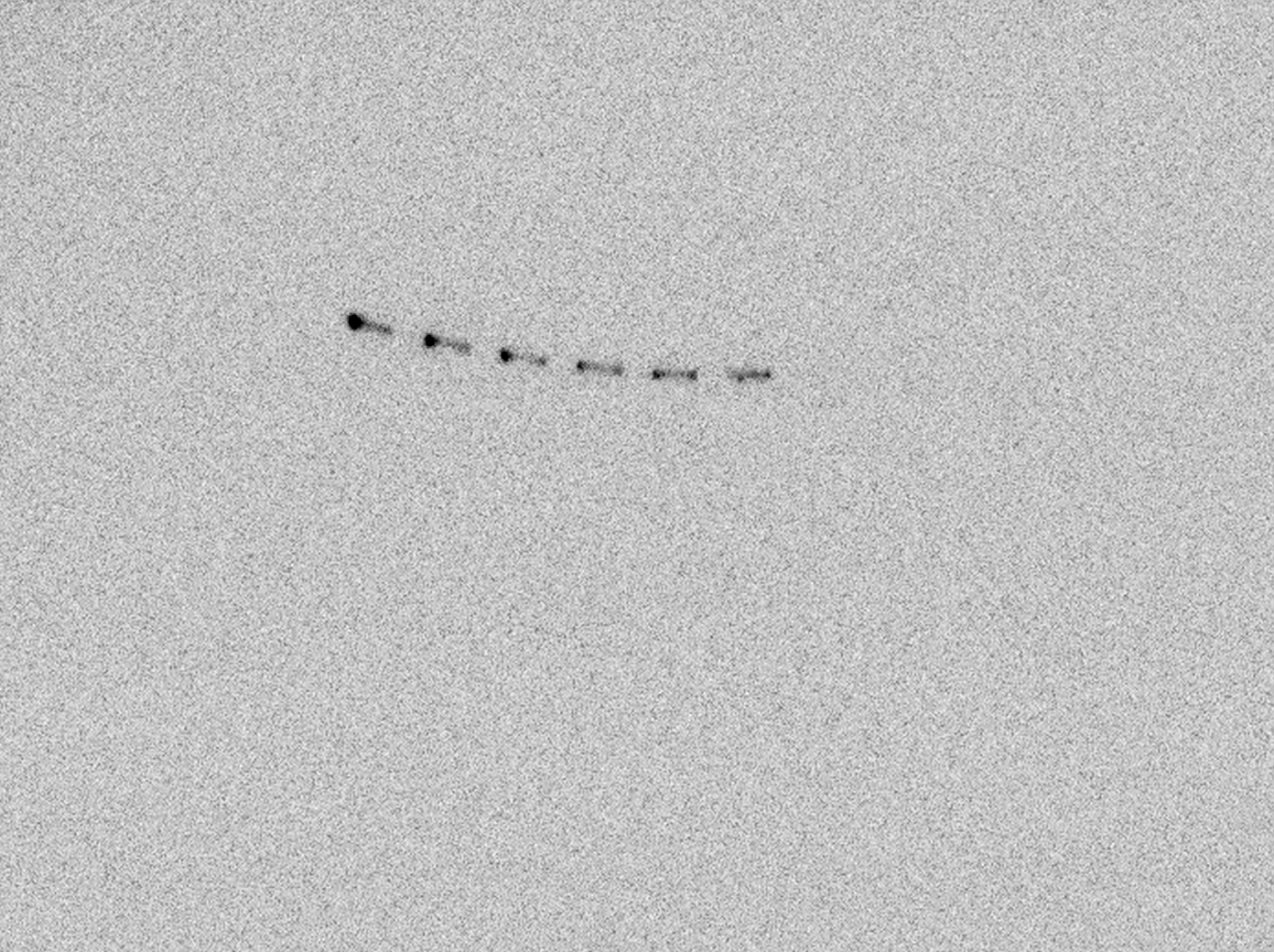

Supplement: Supplementary file 1 [file cancers-13-00862-s001.zip › WBdata_cancers/20201001_TCO1_D1_E7080/201002_TCO1_D1_E7080_c.tif]

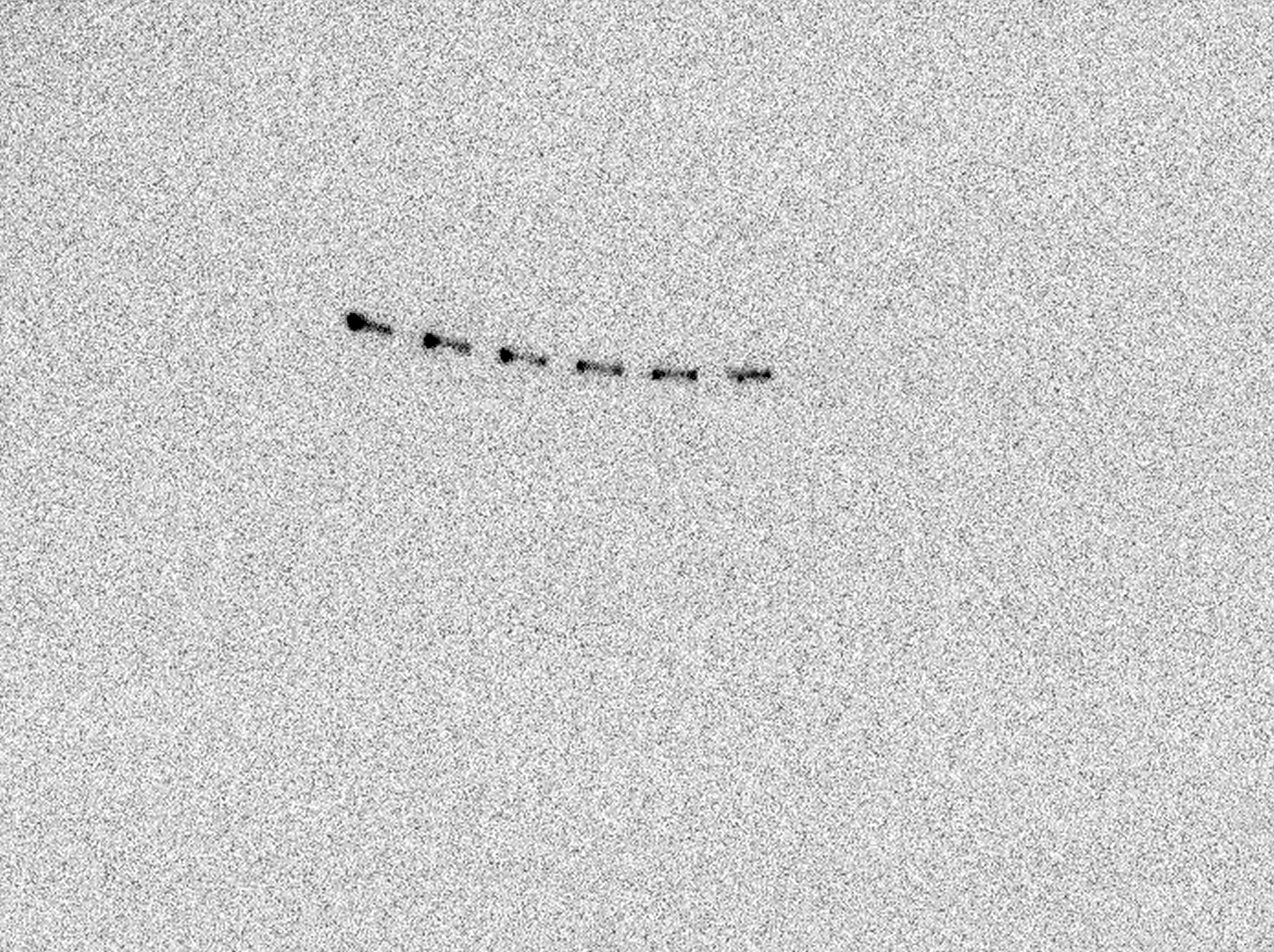

Supplement: Supplementary file 1 [file cancers-13-00862-s001.zip › WBdata_cancers/20201001_TCO1_D1_E7080/201002_TCO1_D1_E7080_d.tif]

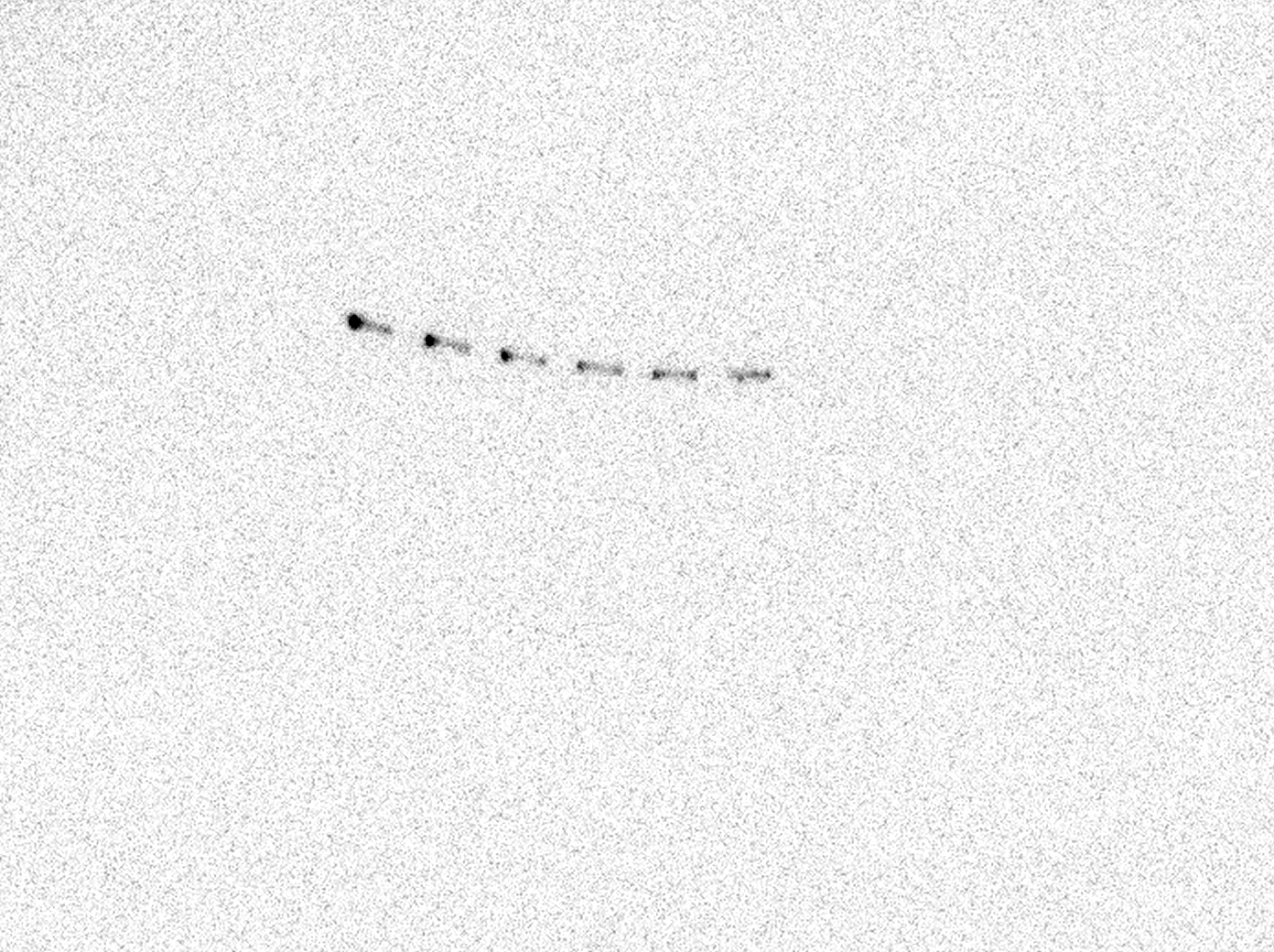

Supplement: Supplementary file 1 [file cancers-13-00862-s001.zip › WBdata_cancers/20201001_TCO1_D1_E7080/201002_TCO1_D1_E7080_e.tif]

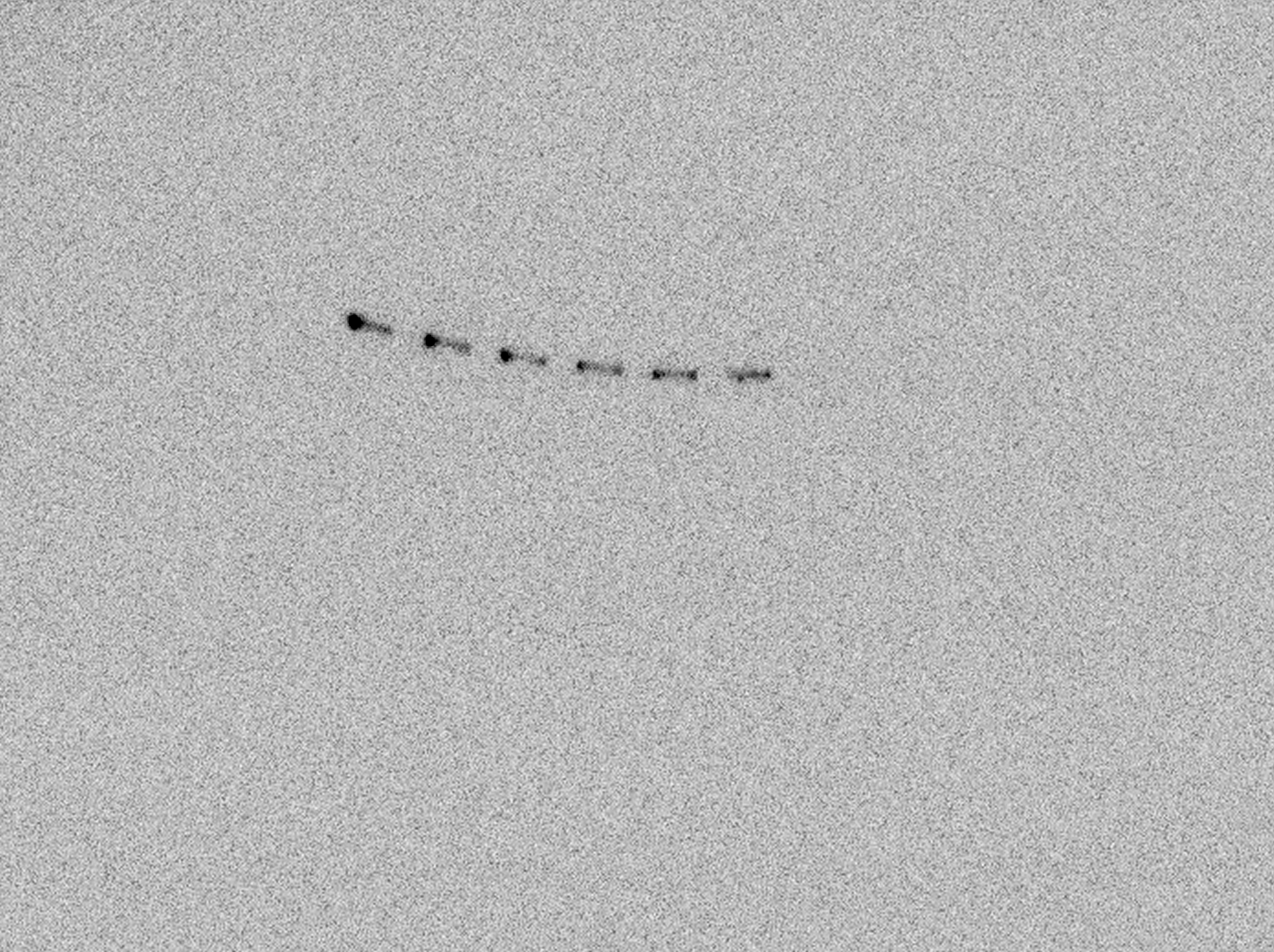

Supplement: Supplementary file 1 [file cancers-13-00862-s001.zip › WBdata_cancers/20201001_TCO1_D1_E7080/201002_TCO1_D1_E7080_f.tif]

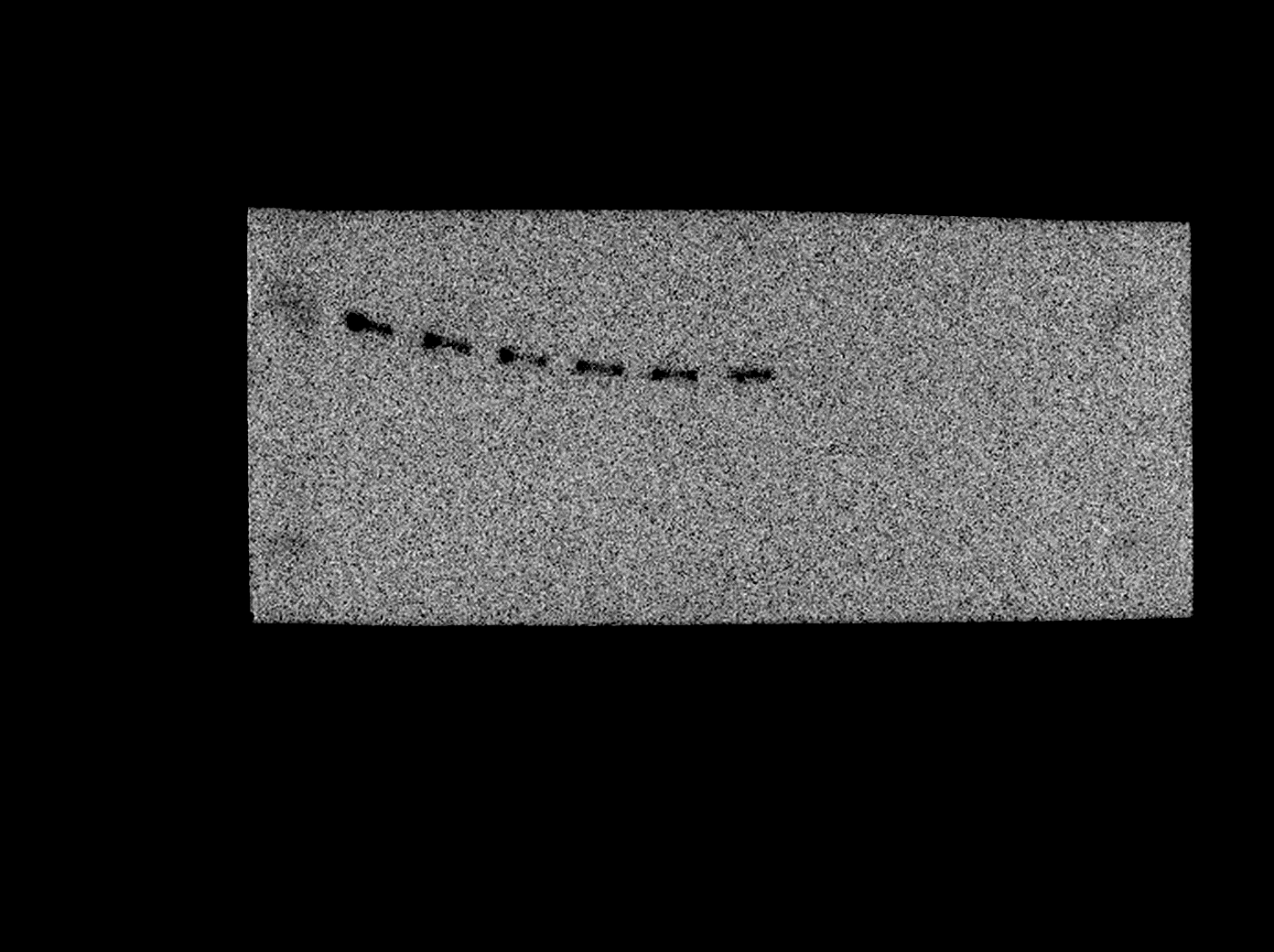

Supplement: Supplementary file 1 [file cancers-13-00862-s001.zip › WBdata_cancers/20201001_TCO1_D1_E7080/201002_TCO1_D1_E7080_Merge.tif]

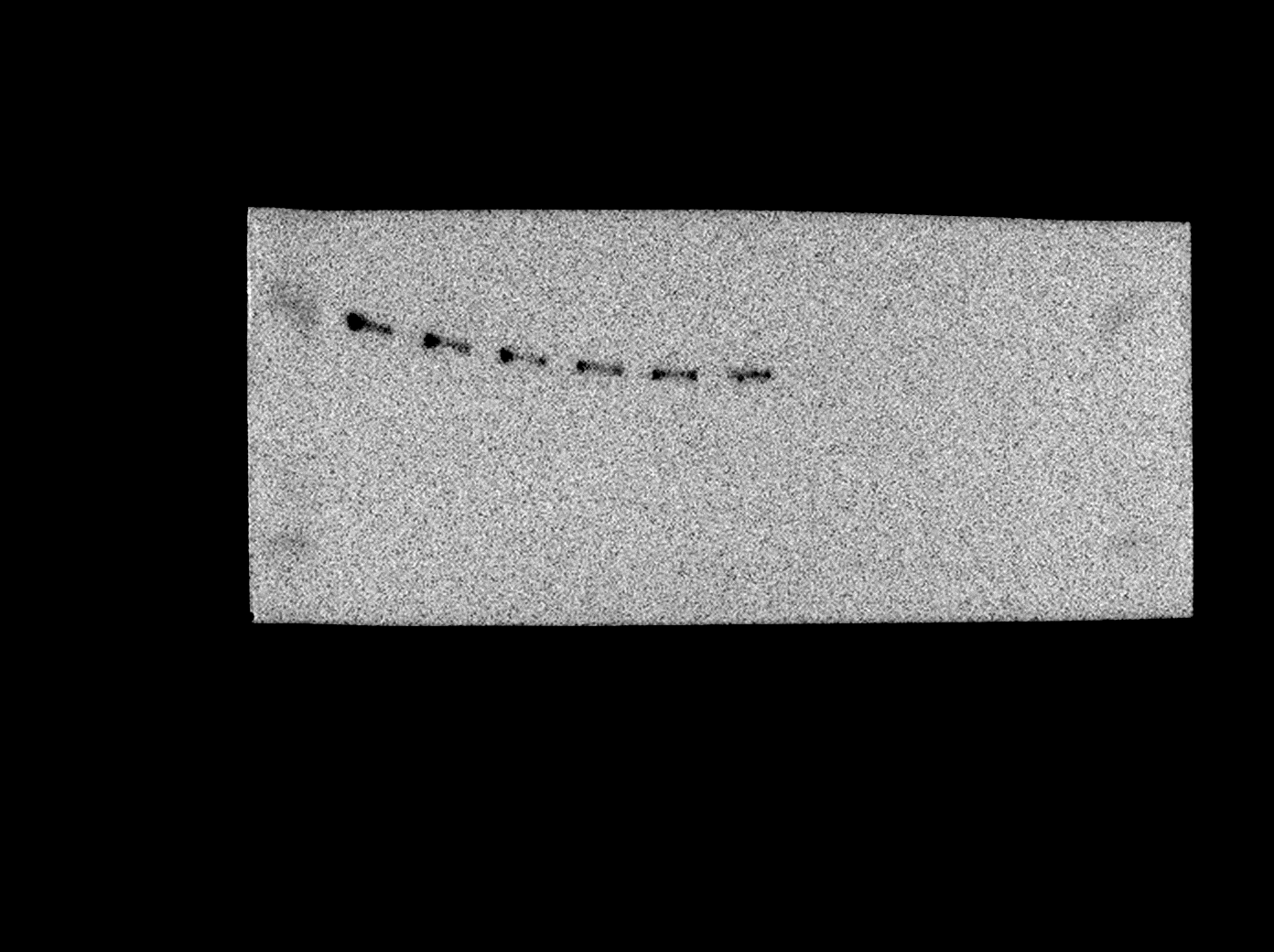

Supplement: Supplementary file 1 [file cancers-13-00862-s001.zip › WBdata_cancers/20201001_TCO1_D1_E7080/201002_TCO1_D1_E7080_Merge2.tif]

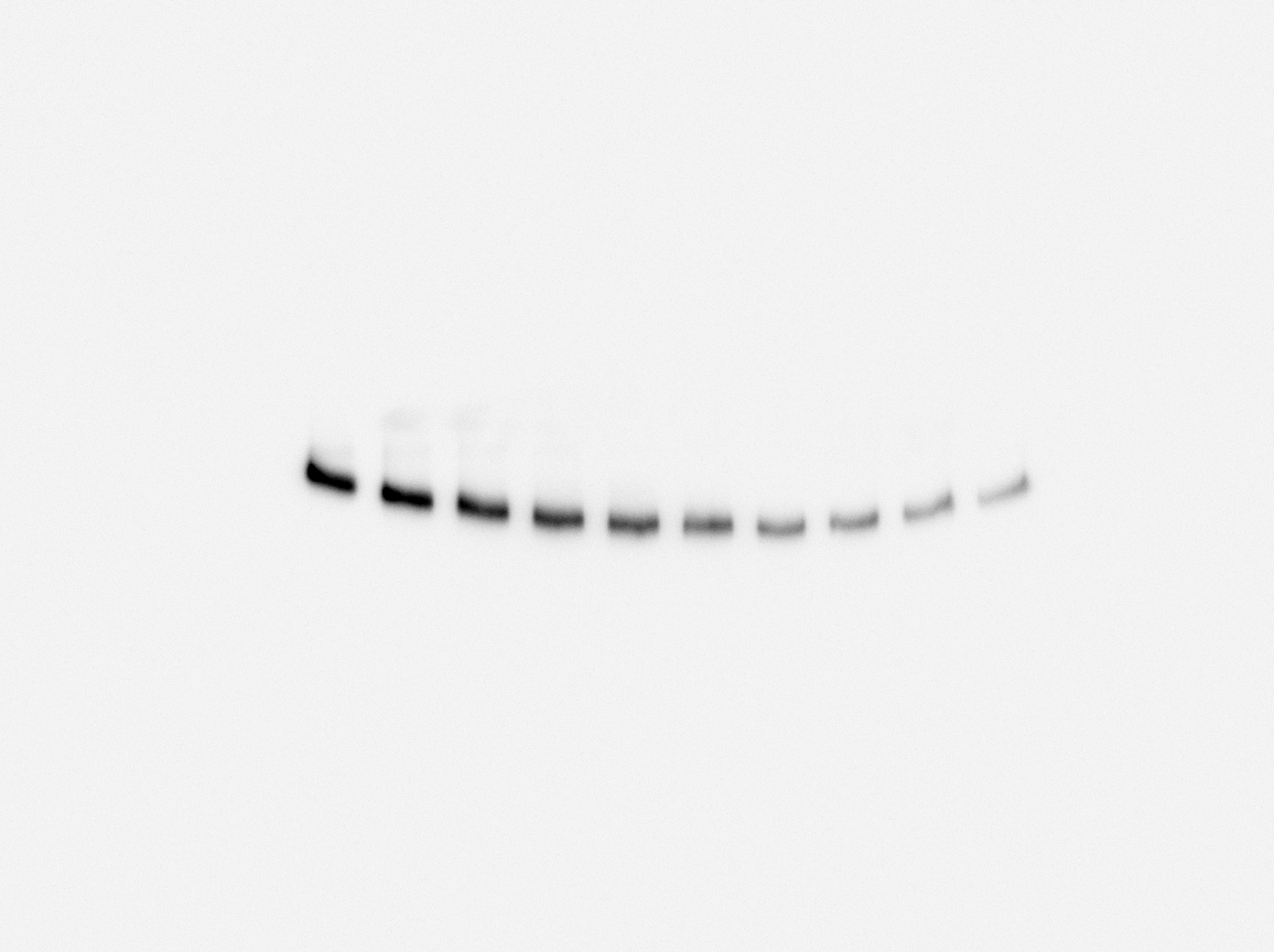

Supplement: Supplementary file 1 [file cancers-13-00862-s001.zip › WBdata_cancers/20201001_TCO1_pAKT_E7080/201001_TC_pAKT_E7080_a.tif]

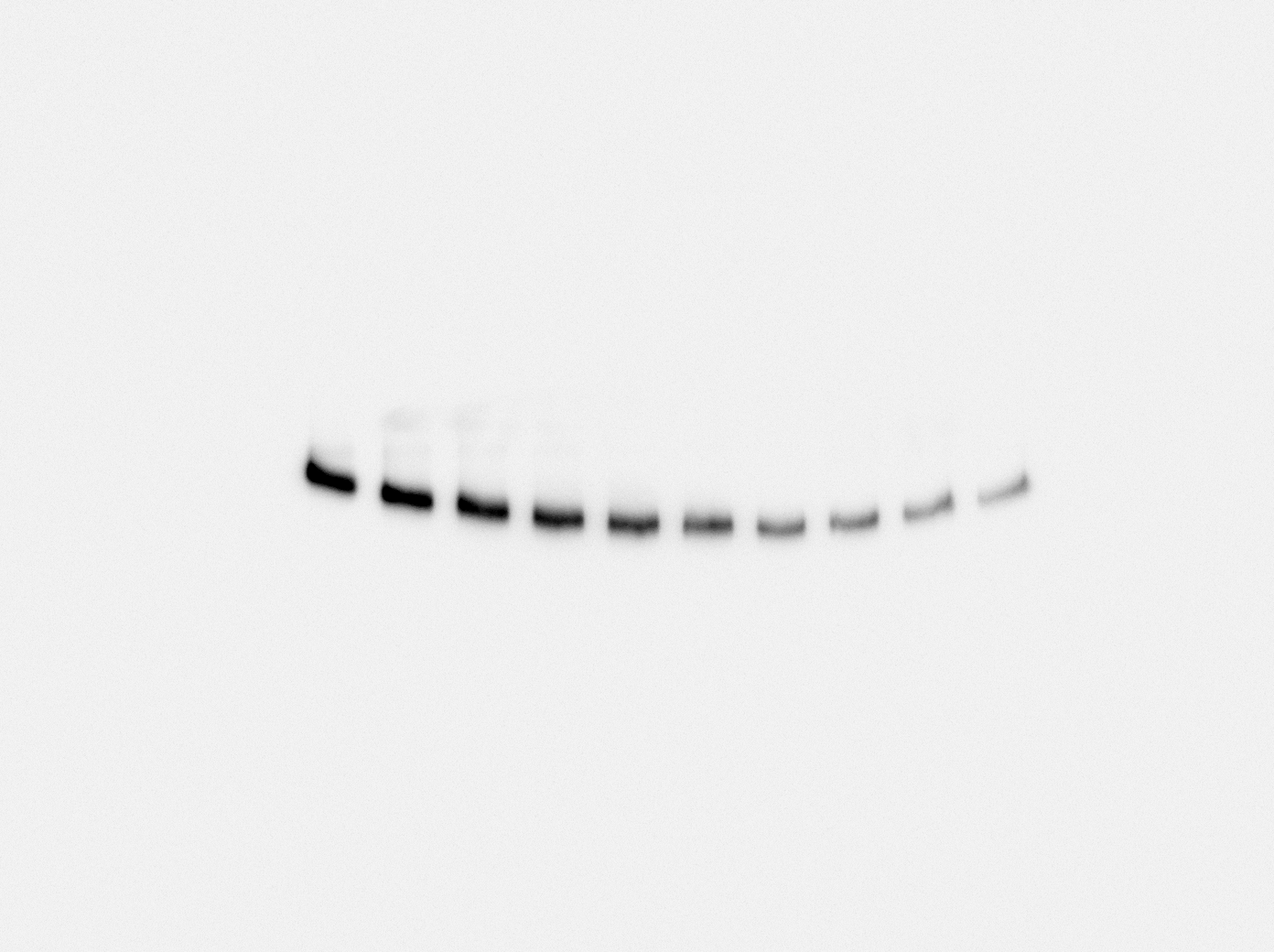

Supplement: Supplementary file 1 [file cancers-13-00862-s001.zip › WBdata_cancers/20201001_TCO1_pAKT_E7080/201001_TC_pAKT_E7080_b.tif]

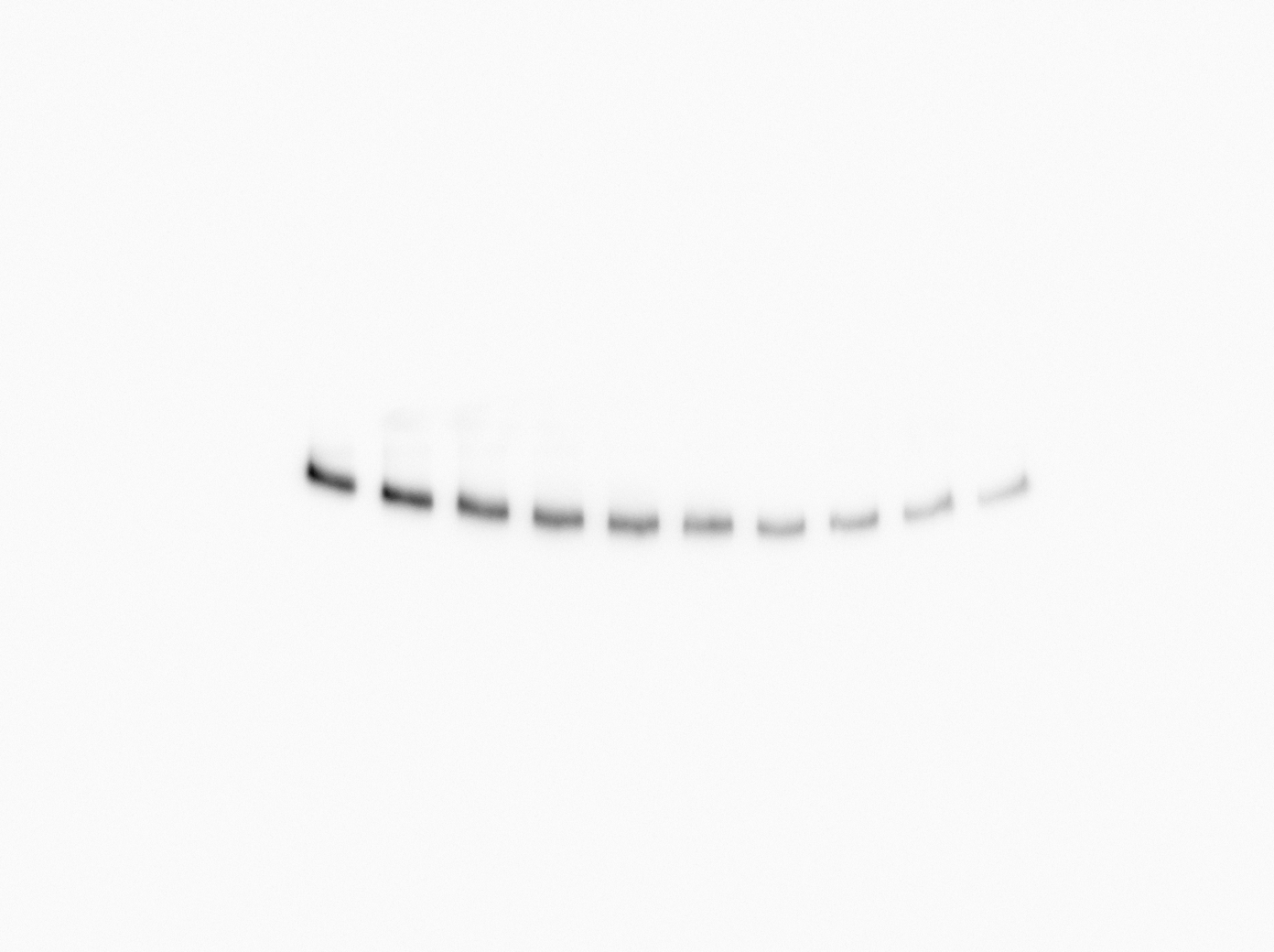

Supplement: Supplementary file 1 [file cancers-13-00862-s001.zip › WBdata_cancers/20201001_TCO1_pAKT_E7080/201001_TC_pAKT_E7080_c.tif]

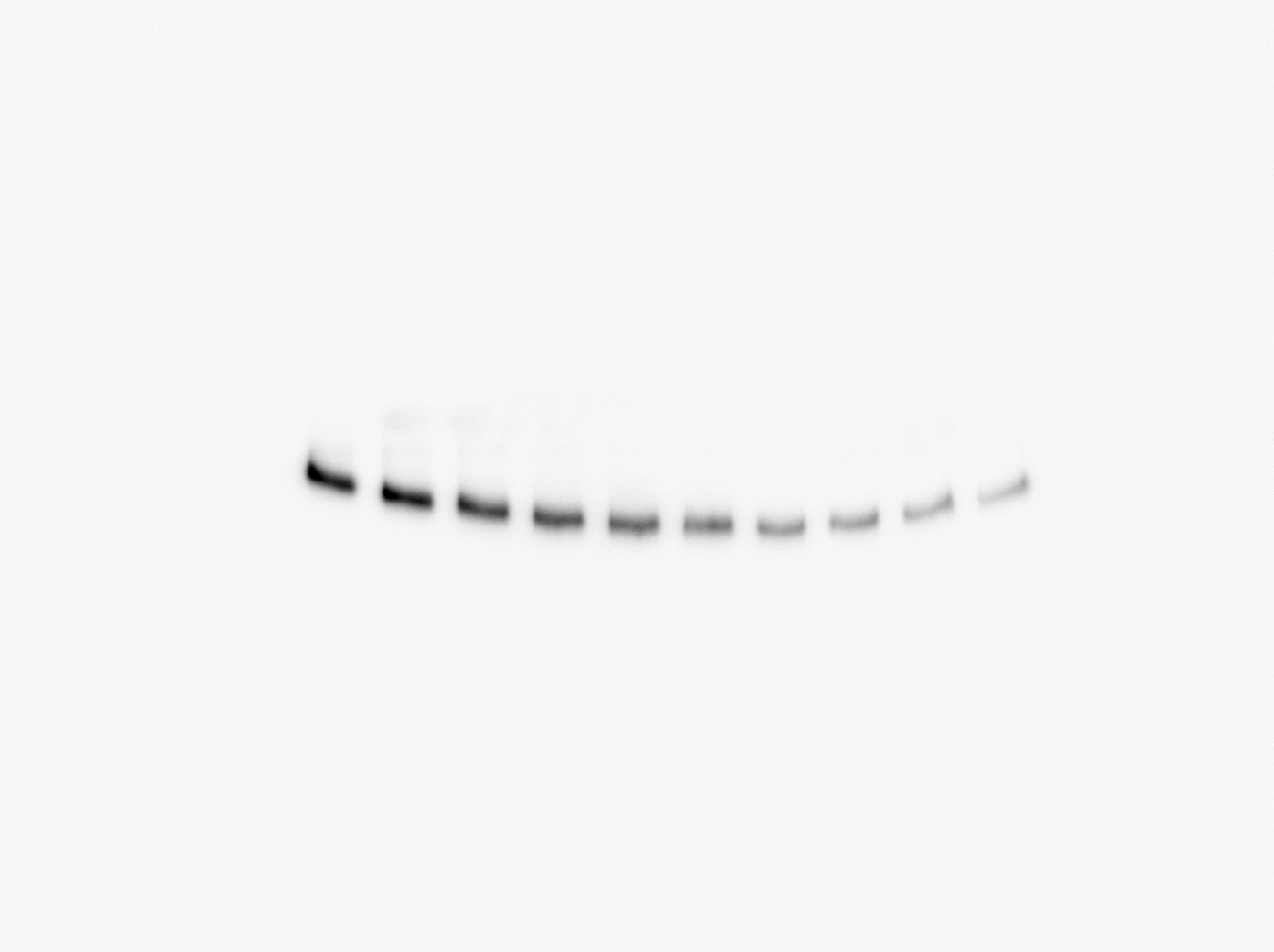

Supplement: Supplementary file 1 [file cancers-13-00862-s001.zip › WBdata_cancers/20201001_TCO1_pAKT_E7080/201001_TC_pAKT_E7080_d.tif]

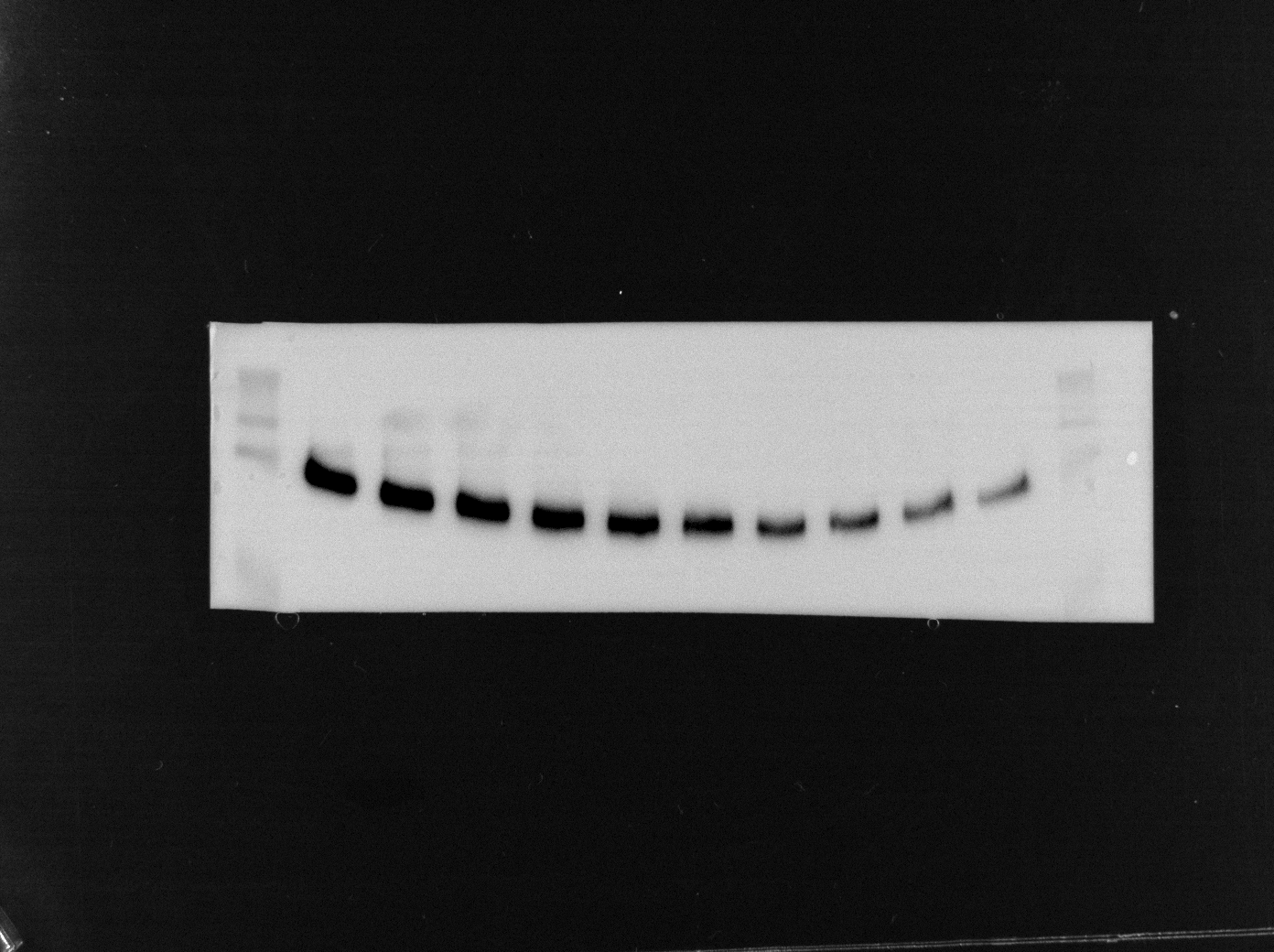

Supplement: Supplementary file 1 [file cancers-13-00862-s001.zip › WBdata_cancers/20201001_TCO1_pAKT_E7080/201001_TC_pAKT_E7080_Merge.tif]

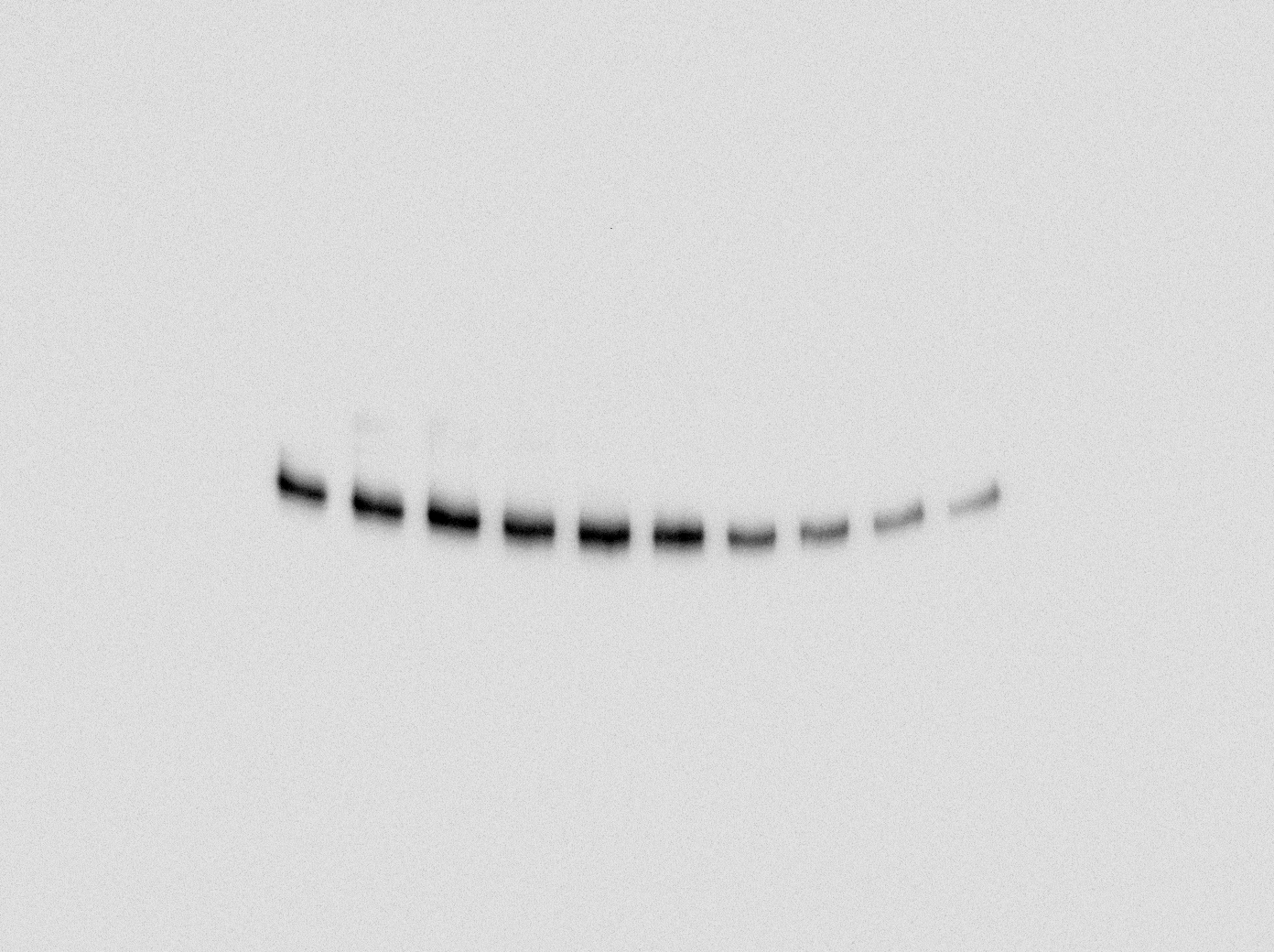

Supplement: Supplementary file 1 [file cancers-13-00862-s001.zip › WBdata_cancers/20201001_TCO1_tAKT_E7080/201002_TCO1_tAKT_E7080_a.tif]

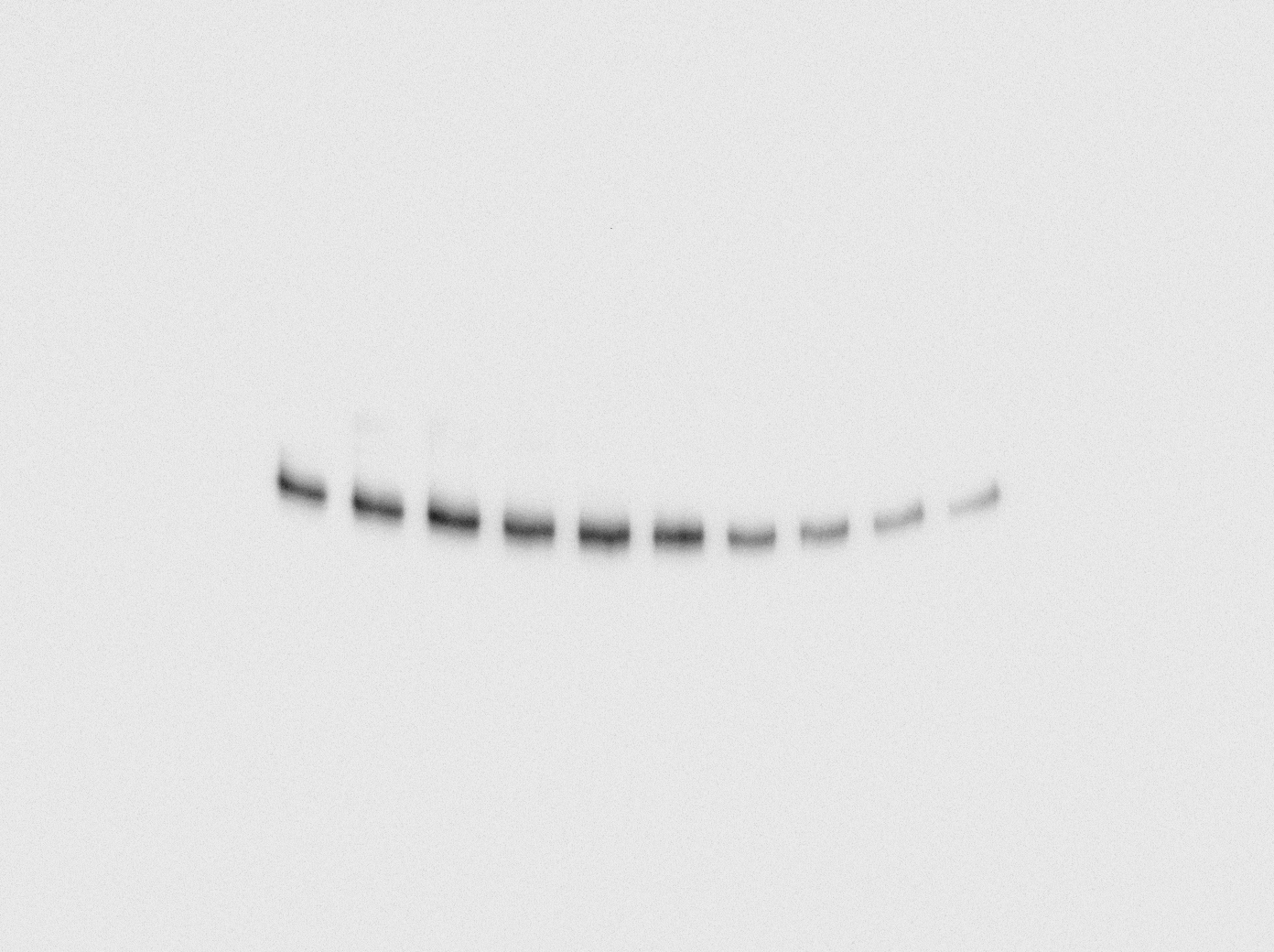

Supplement: Supplementary file 1 [file cancers-13-00862-s001.zip › WBdata_cancers/20201001_TCO1_tAKT_E7080/201002_TCO1_tAKT_E7080_b.tif]

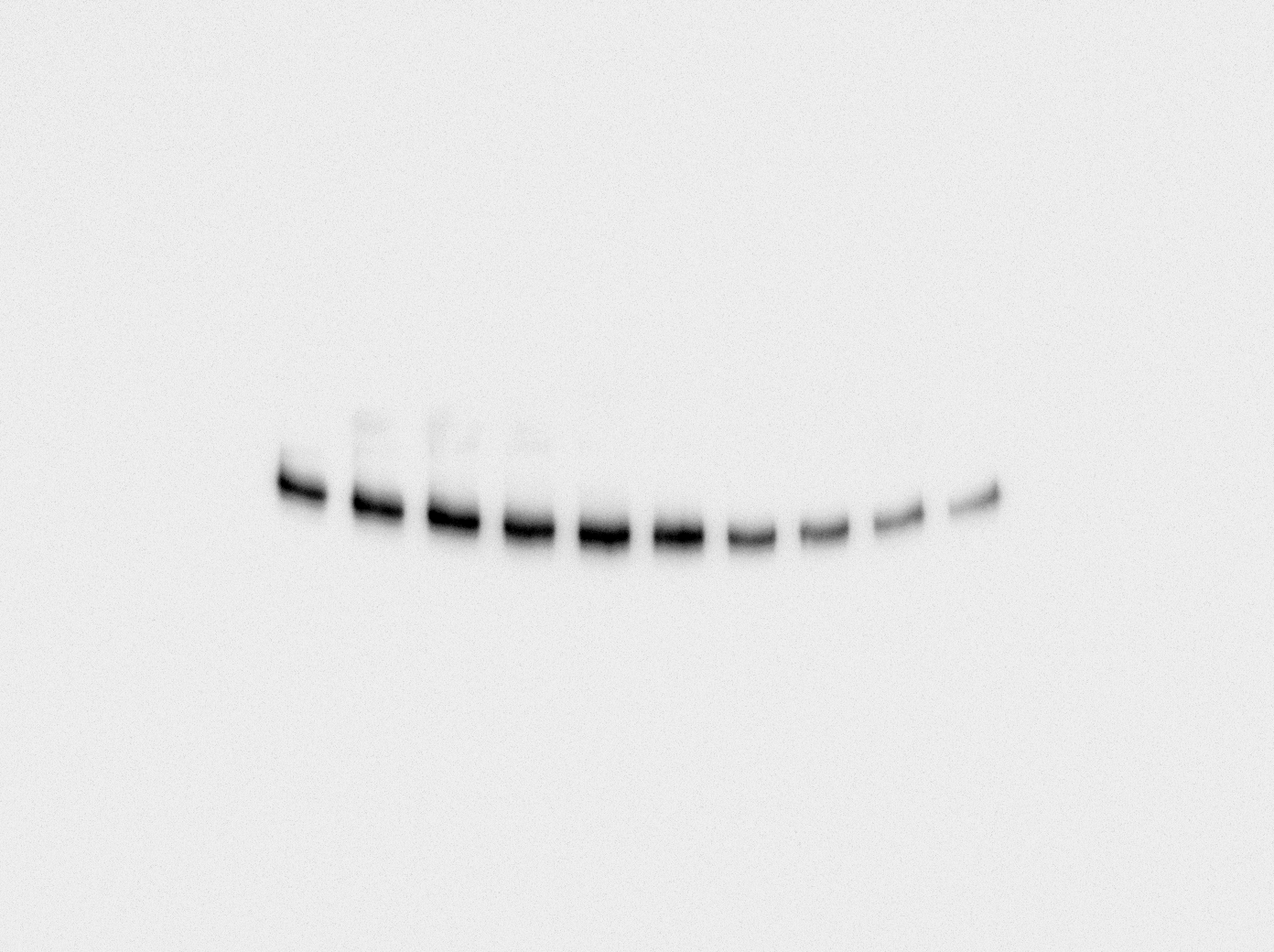

Supplement: Supplementary file 1 [file cancers-13-00862-s001.zip › WBdata_cancers/20201001_TCO1_tAKT_E7080/201002_TCO1_tAKT_E7080_c.tif]

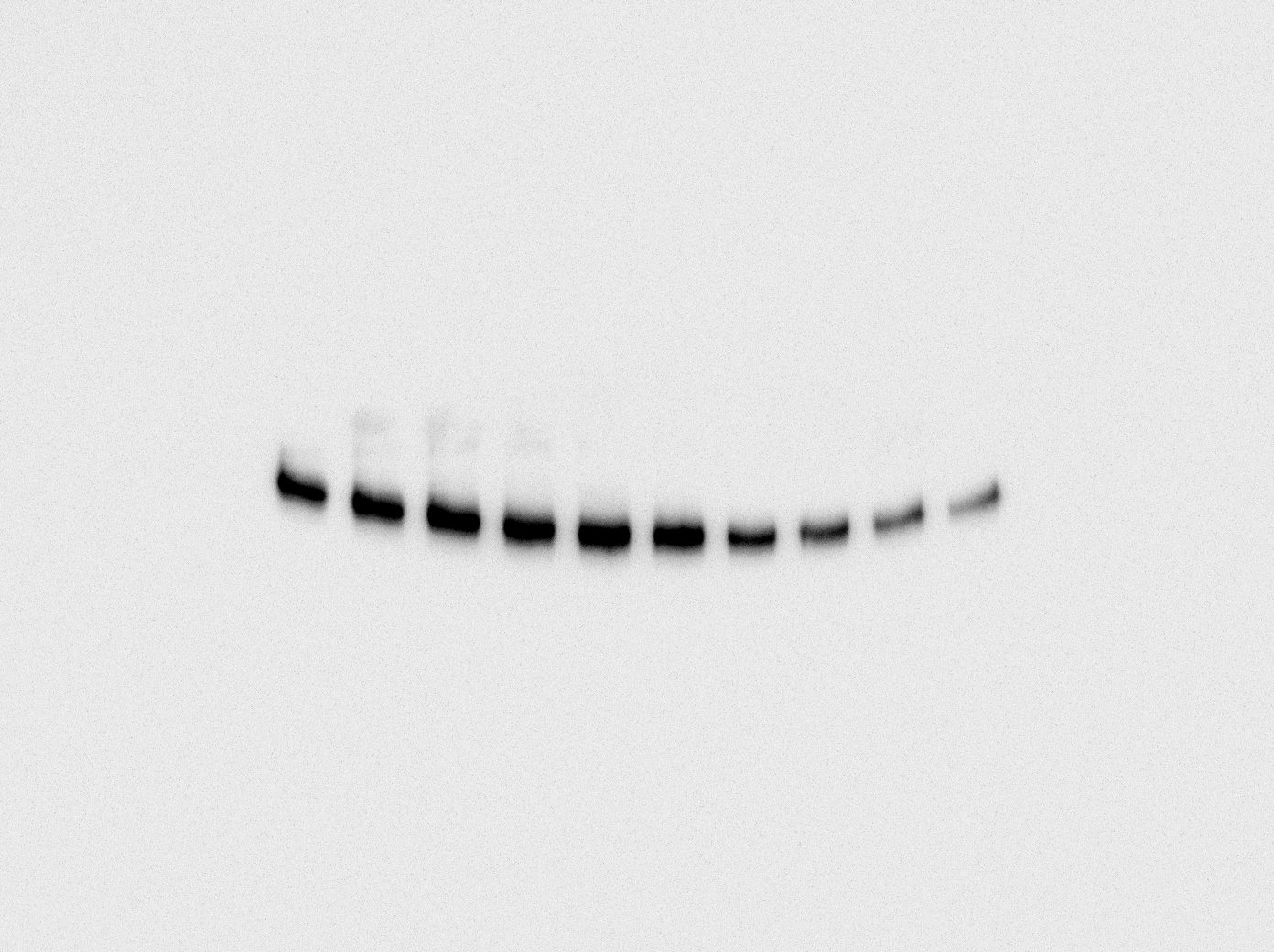

Supplement: Supplementary file 1 [file cancers-13-00862-s001.zip › WBdata_cancers/20201001_TCO1_tAKT_E7080/201002_TCO1_tAKT_E7080_d.tif]

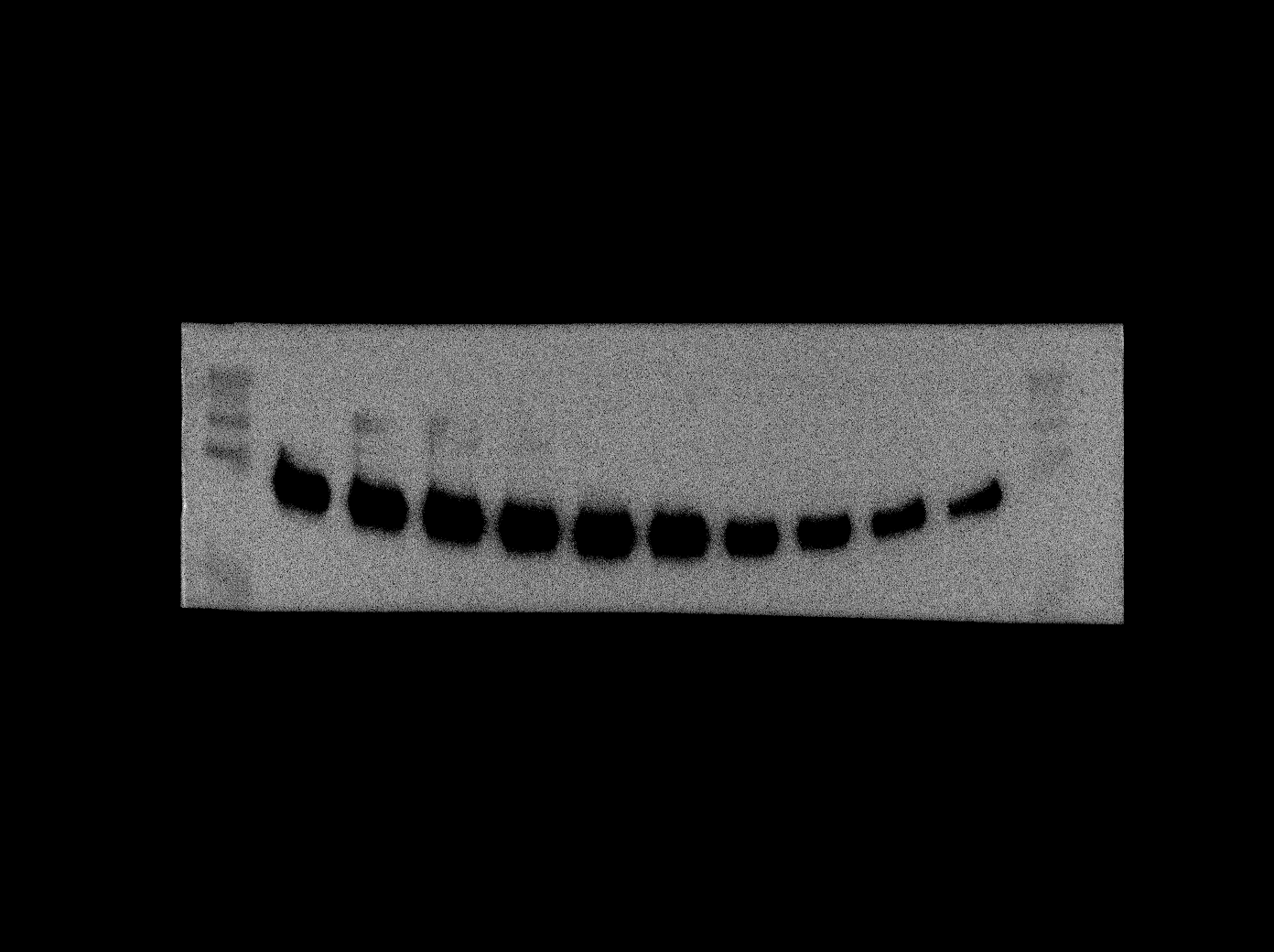

Supplement: Supplementary file 1 [file cancers-13-00862-s001.zip › WBdata_cancers/20201001_TCO1_tAKT_E7080/201002_TCO1_tAKT_E7080_Merge.tif]

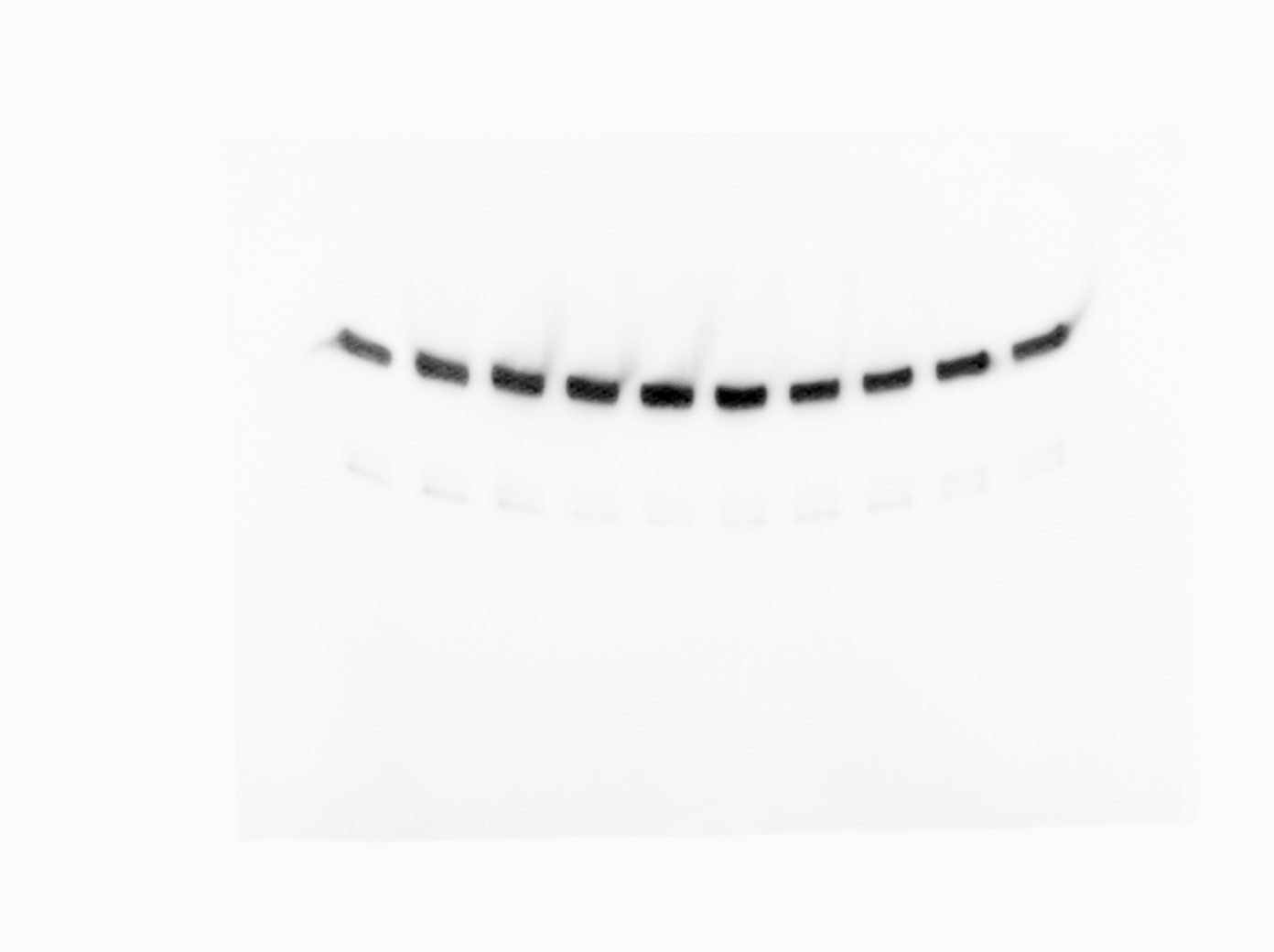

Supplement: Supplementary file 1 [file cancers-13-00862-s001.zip › WBdata_cancers/20201009_TCO1_aTUB_E7080/20201009_TCO1_aTUB_E7080_a.tif]

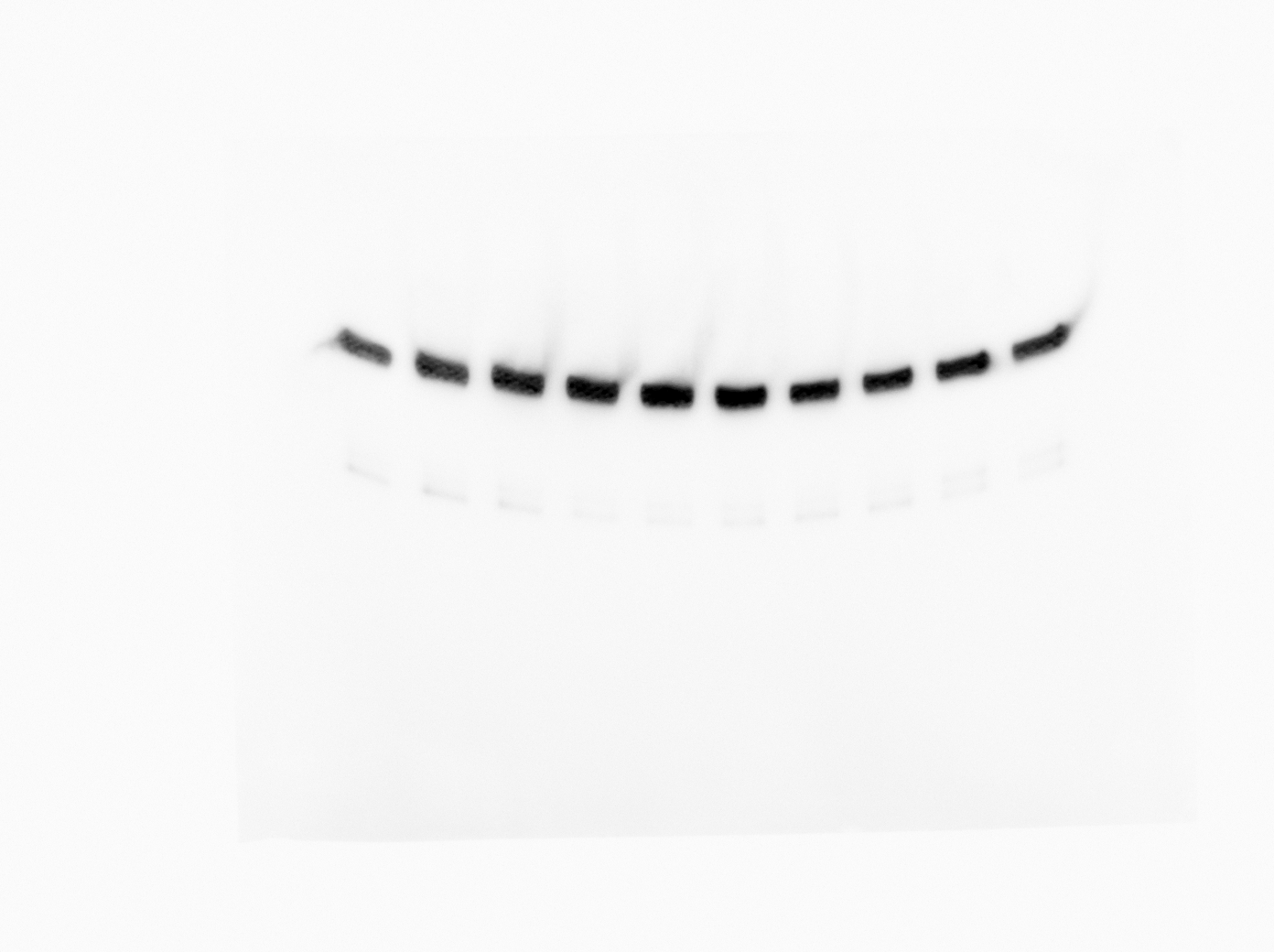

Supplement: Supplementary file 1 [file cancers-13-00862-s001.zip › WBdata_cancers/20201009_TCO1_aTUB_E7080/20201009_TCO1_aTUB_E7080_b.tif]

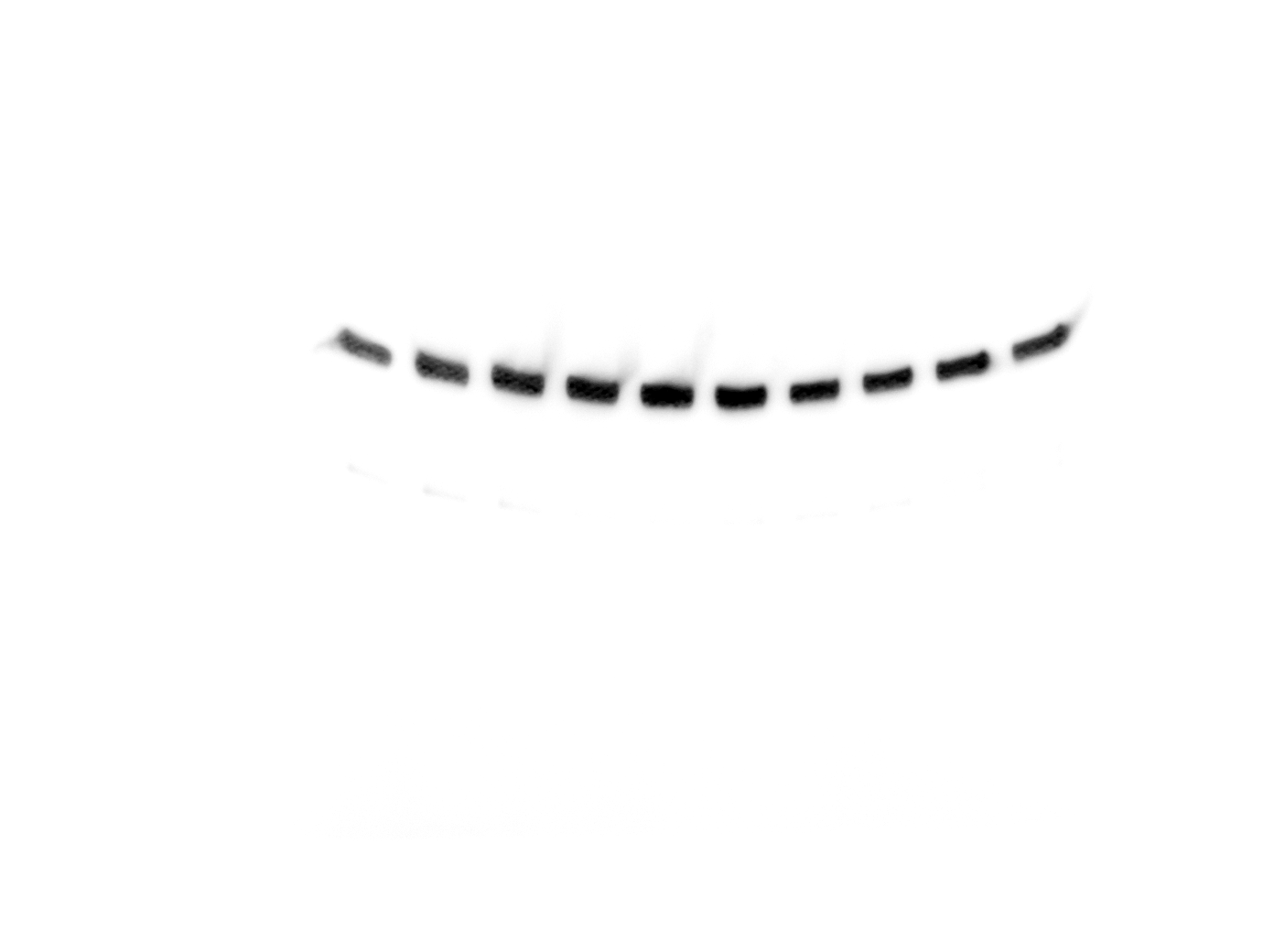

Supplement: Supplementary file 1 [file cancers-13-00862-s001.zip › WBdata_cancers/20201009_TCO1_aTUB_E7080/20201009_TCO1_aTUB_E7080_c.tif]

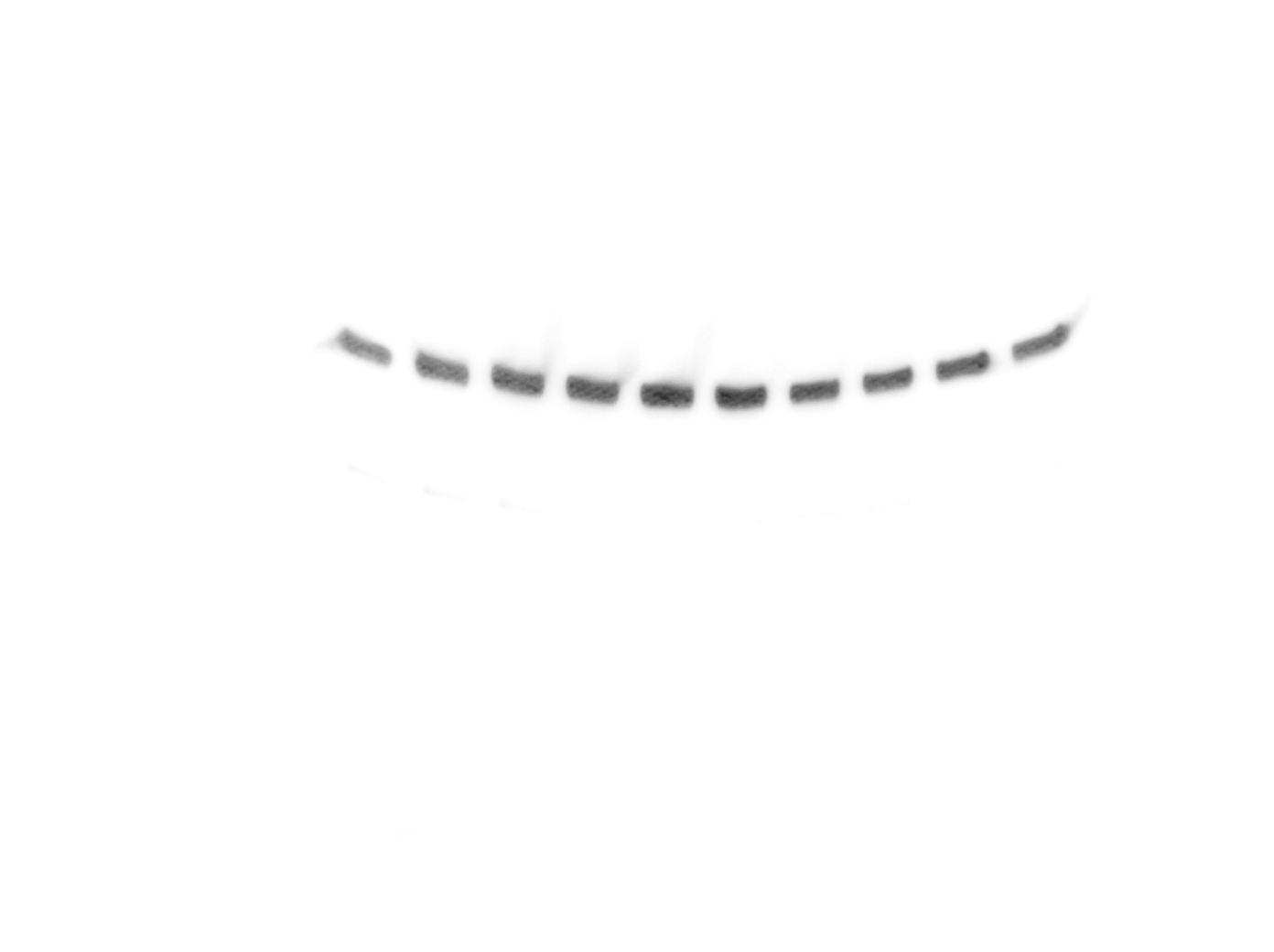

Supplement: Supplementary file 1 [file cancers-13-00862-s001.zip › WBdata_cancers/20201009_TCO1_aTUB_E7080/20201009_TCO1_aTUB_E7080_d.tif]

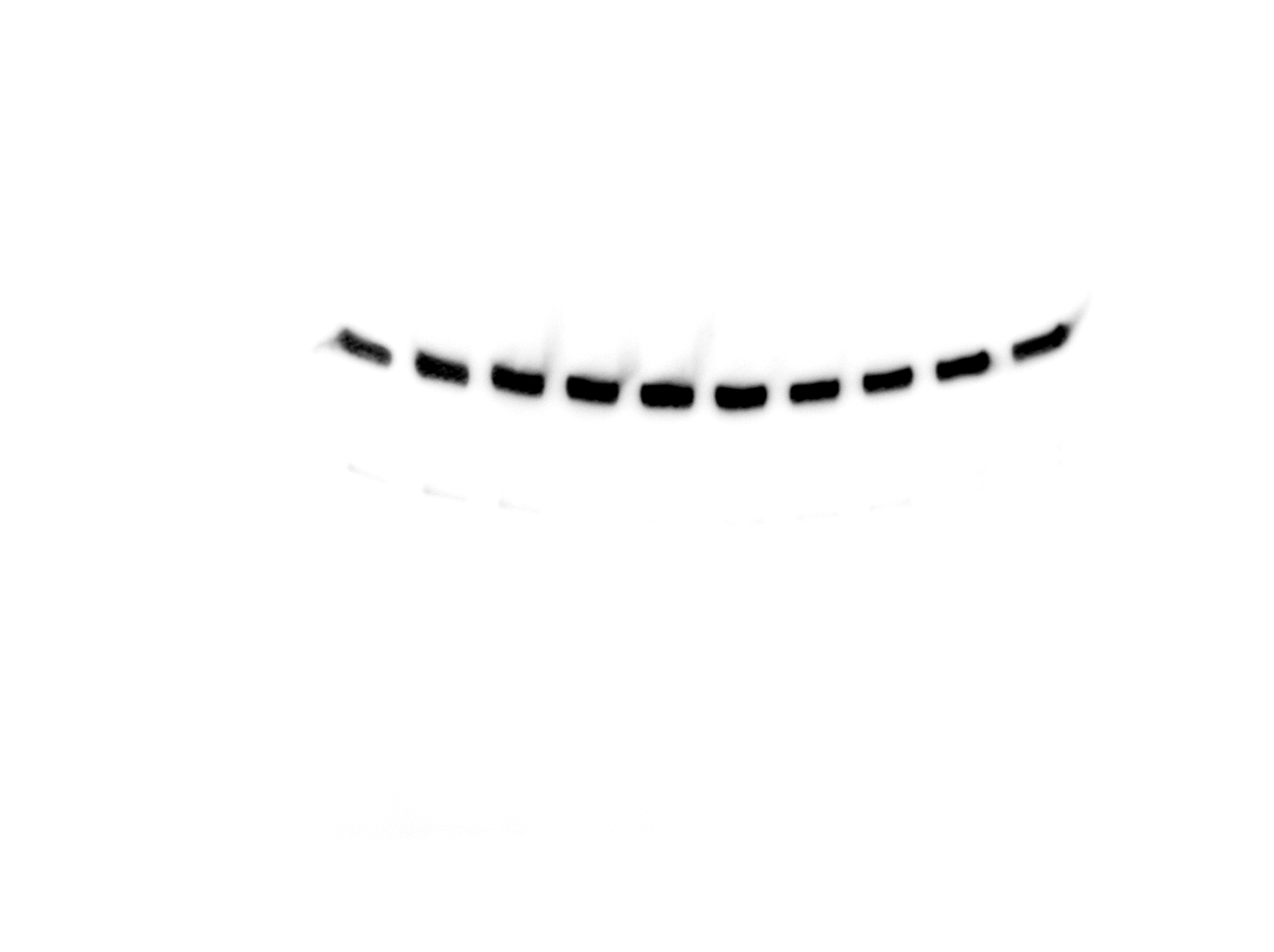

Supplement: Supplementary file 1 [file cancers-13-00862-s001.zip › WBdata_cancers/20201009_TCO1_aTUB_E7080/20201009_TCO1_aTUB_E7080_e.tif]

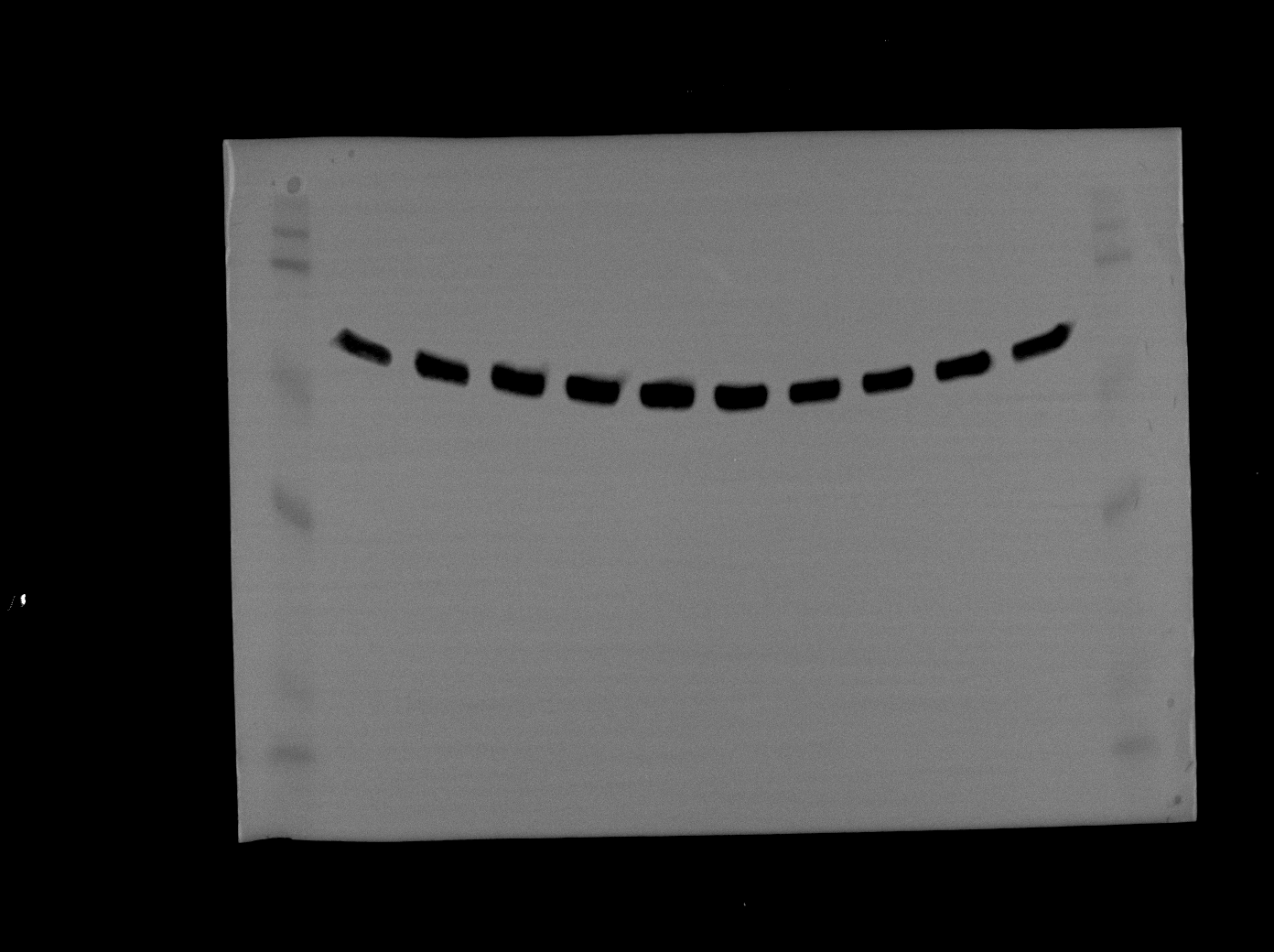

Supplement: Supplementary file 1 [file cancers-13-00862-s001.zip › WBdata_cancers/20201009_TCO1_aTUB_E7080/20201009_TCO1_aTUB_E7080_Merge.tif]

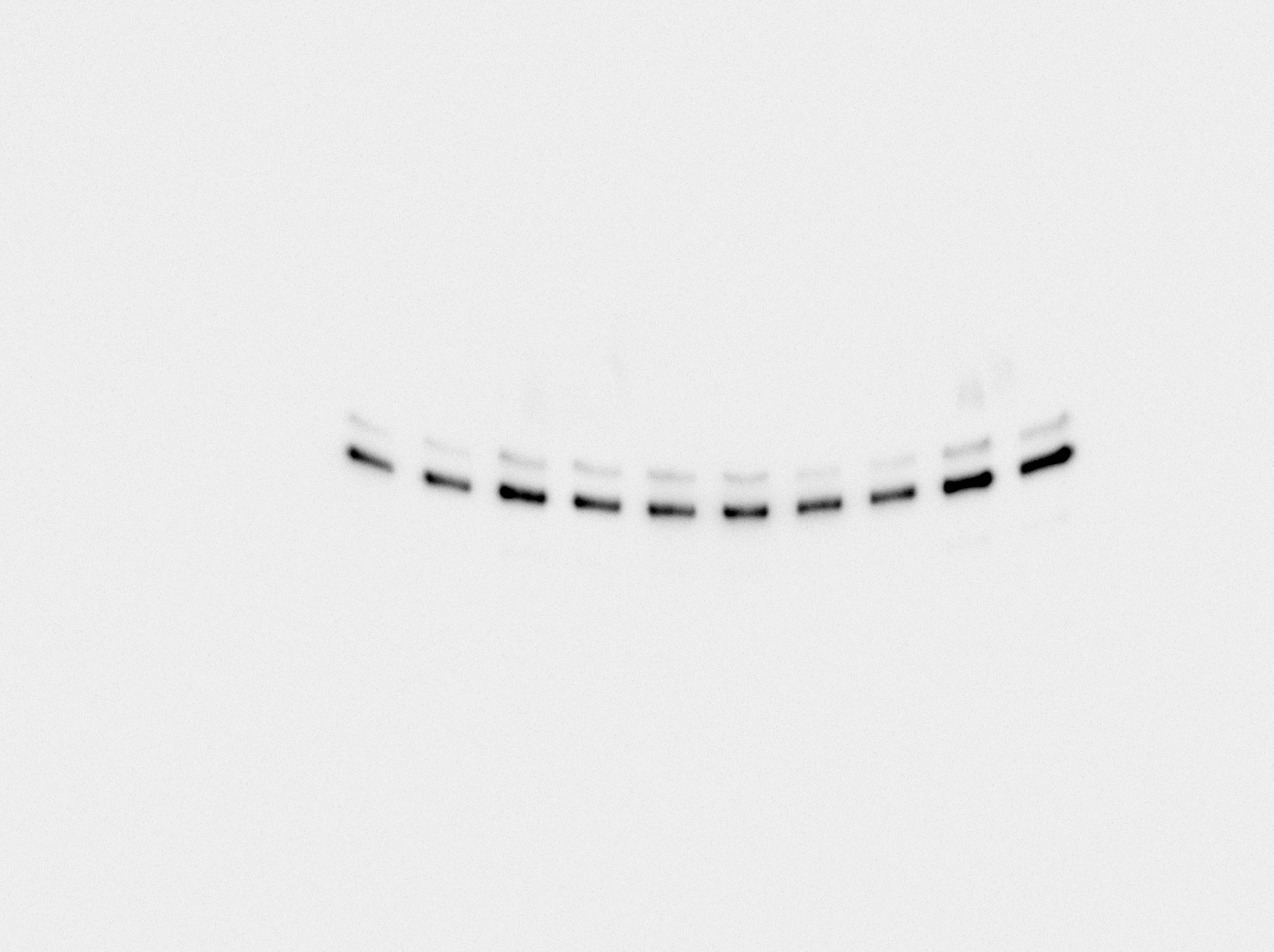

Supplement: Supplementary file 1 [file cancers-13-00862-s001.zip › WBdata_cancers/20201009_TCO1_pERK_E7080/20201009_TCO1_pERK_E7080_a.tif]

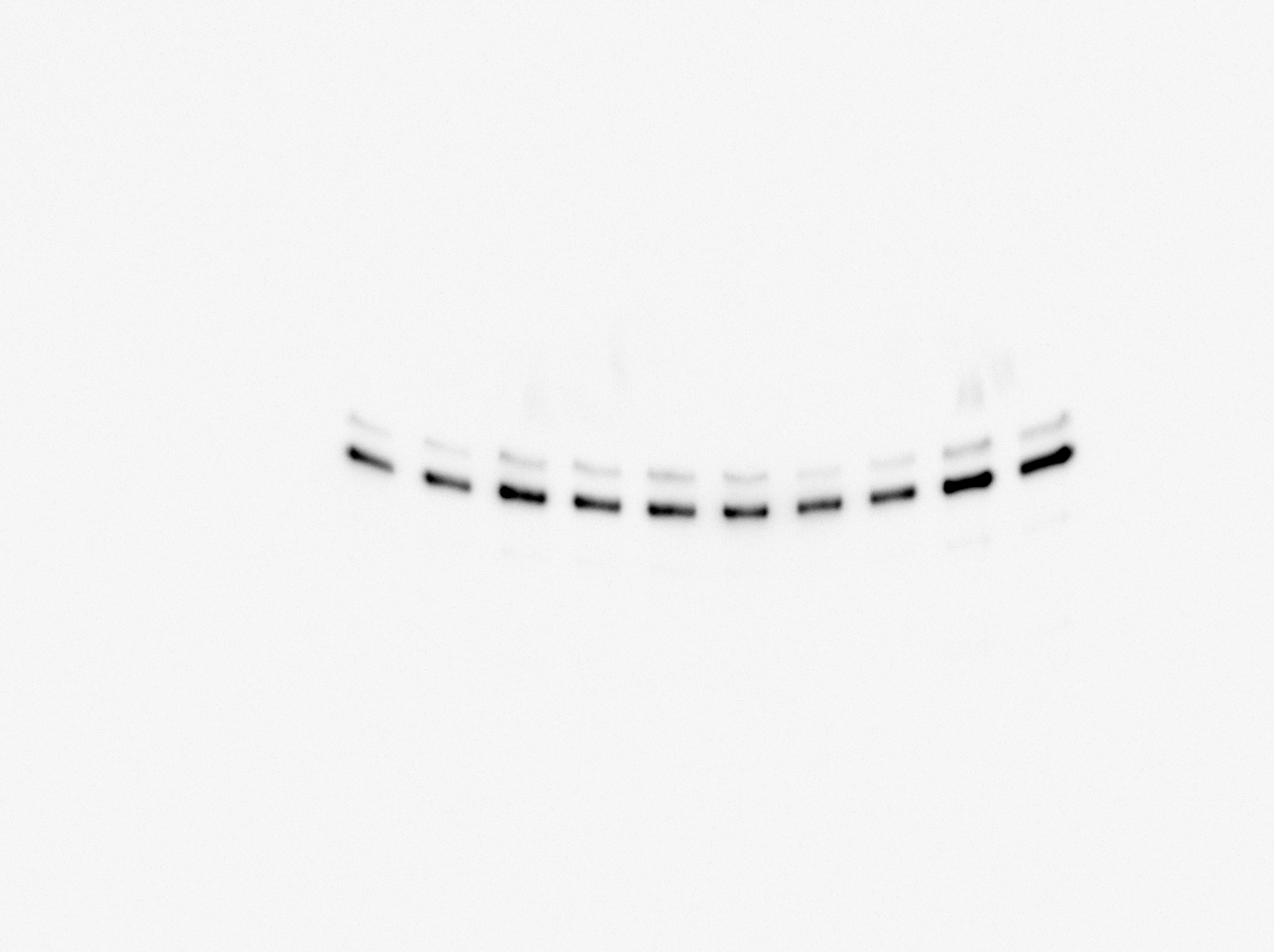

Supplement: Supplementary file 1 [file cancers-13-00862-s001.zip › WBdata_cancers/20201009_TCO1_pERK_E7080/20201009_TCO1_pERK_E7080_b.tif]

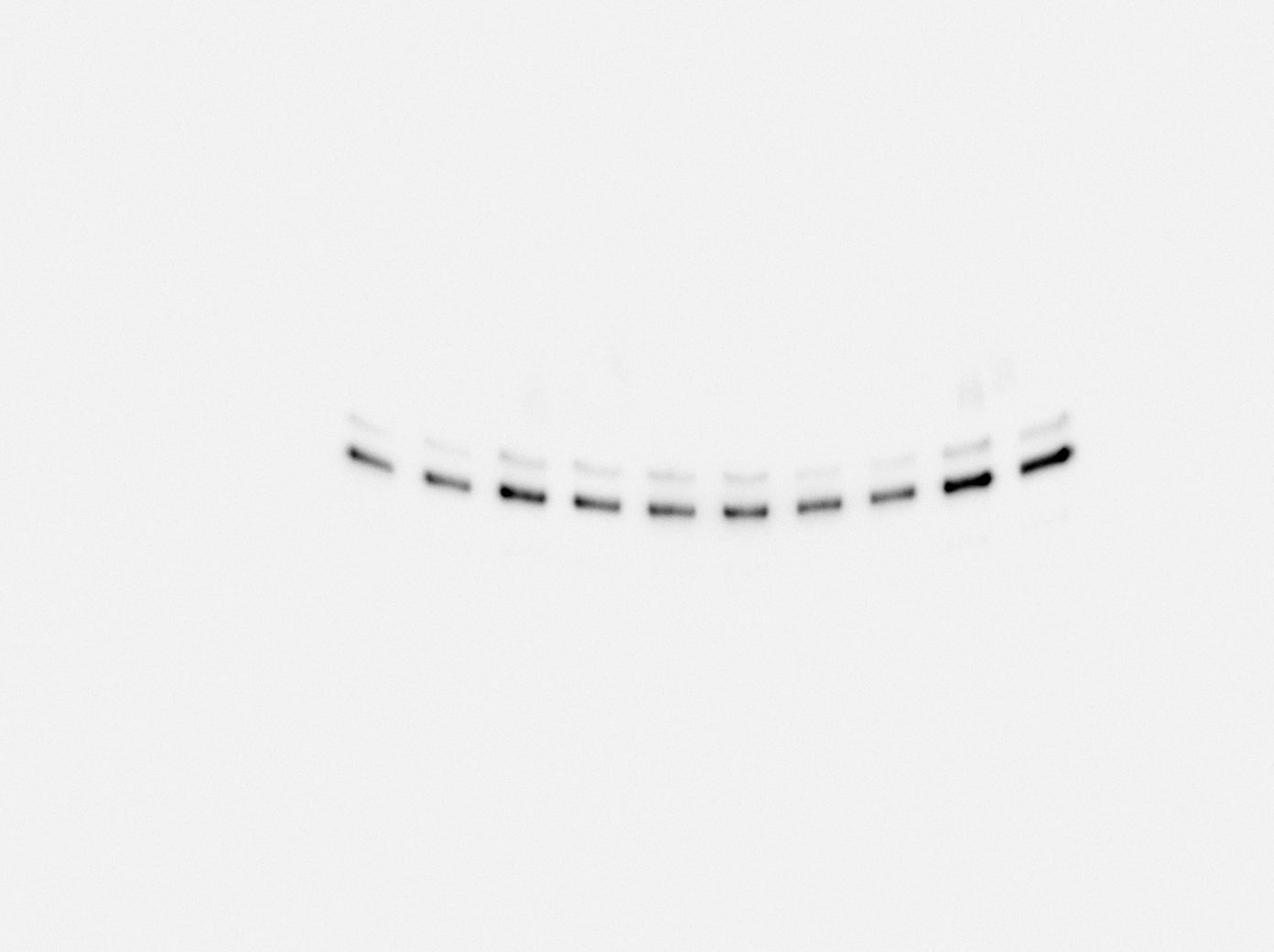

Supplement: Supplementary file 1 [file cancers-13-00862-s001.zip › WBdata_cancers/20201009_TCO1_pERK_E7080/20201009_TCO1_pERK_E7080_c.tif]

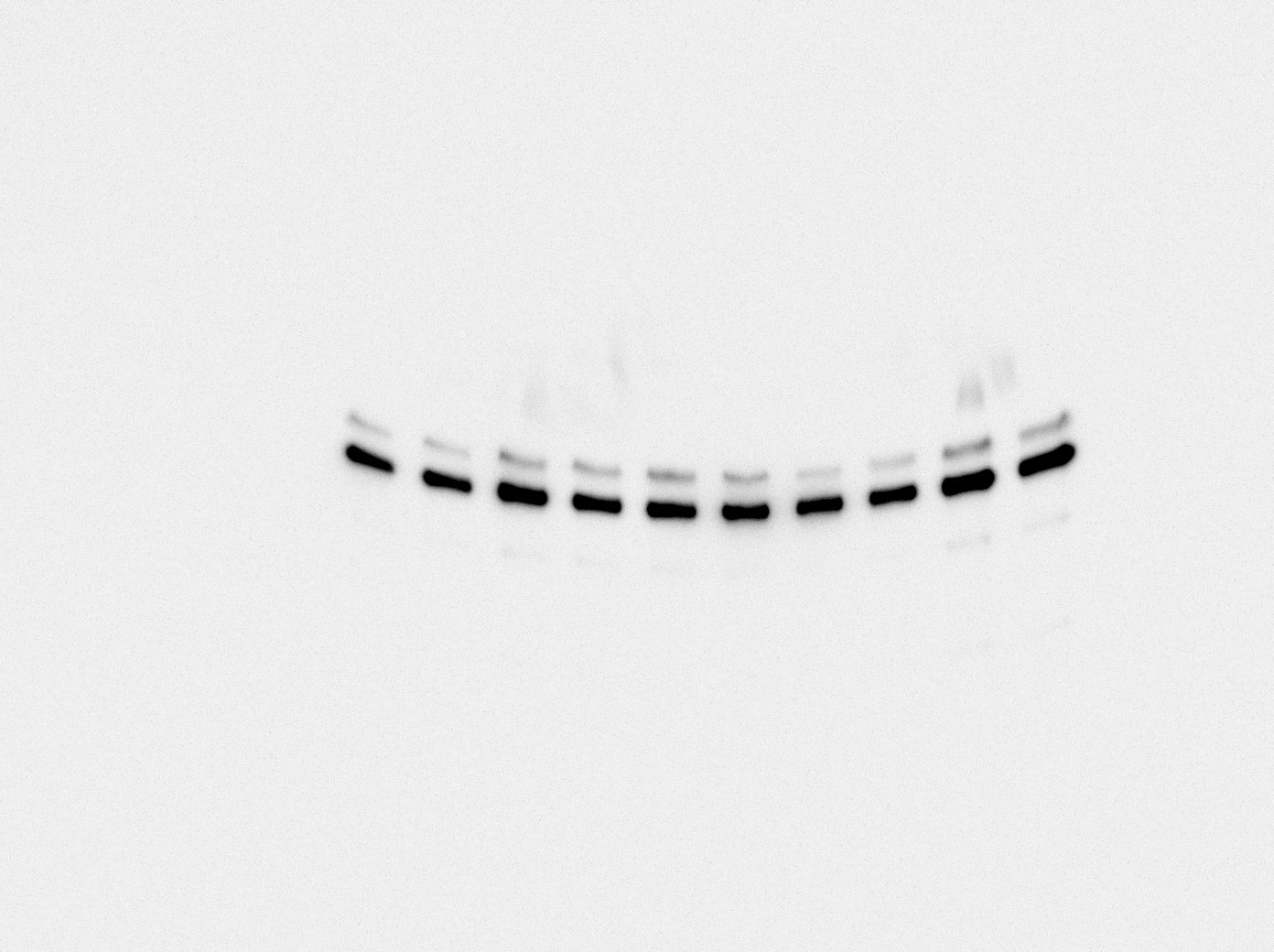

Supplement: Supplementary file 1 [file cancers-13-00862-s001.zip › WBdata_cancers/20201009_TCO1_pERK_E7080/20201009_TCO1_pERK_E7080_d.tif]

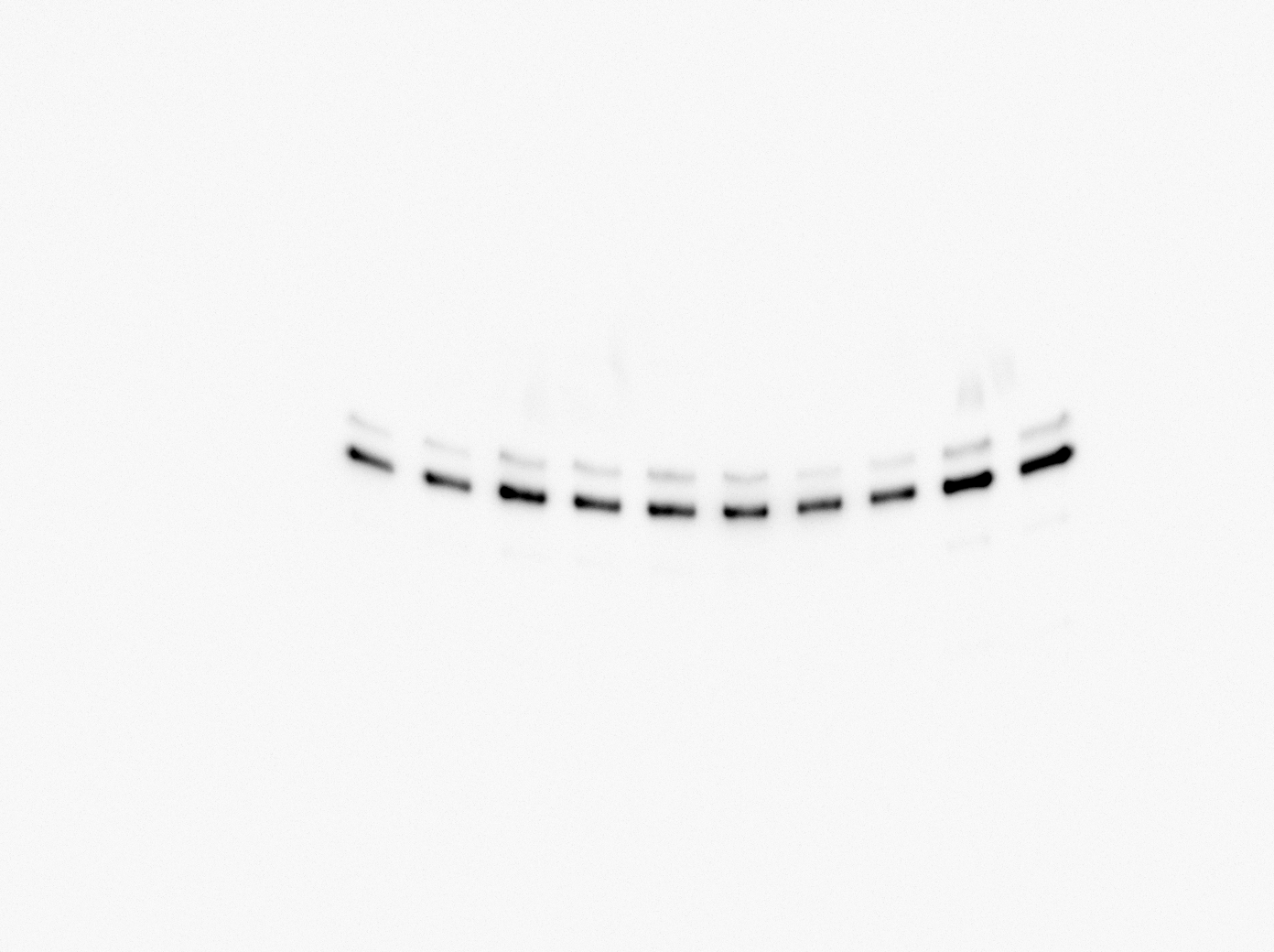

Supplement: Supplementary file 1 [file cancers-13-00862-s001.zip › WBdata_cancers/20201009_TCO1_pERK_E7080/20201009_TCO1_pERK_E7080_e.tif]

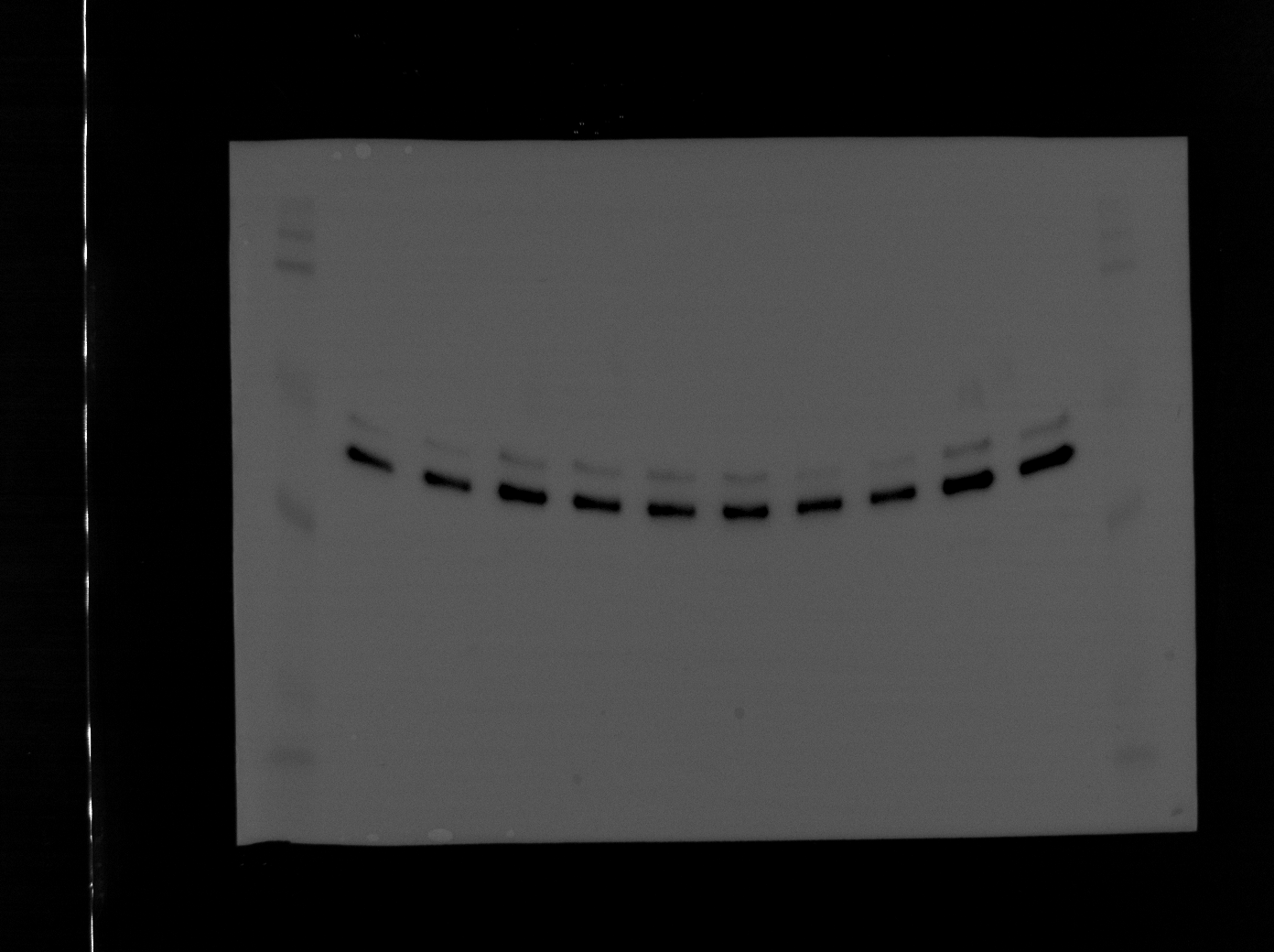

Supplement: Supplementary file 1 [file cancers-13-00862-s001.zip › WBdata_cancers/20201009_TCO1_pERK_E7080/20201009_TCO1_pERK_E7080_Merge.tif]

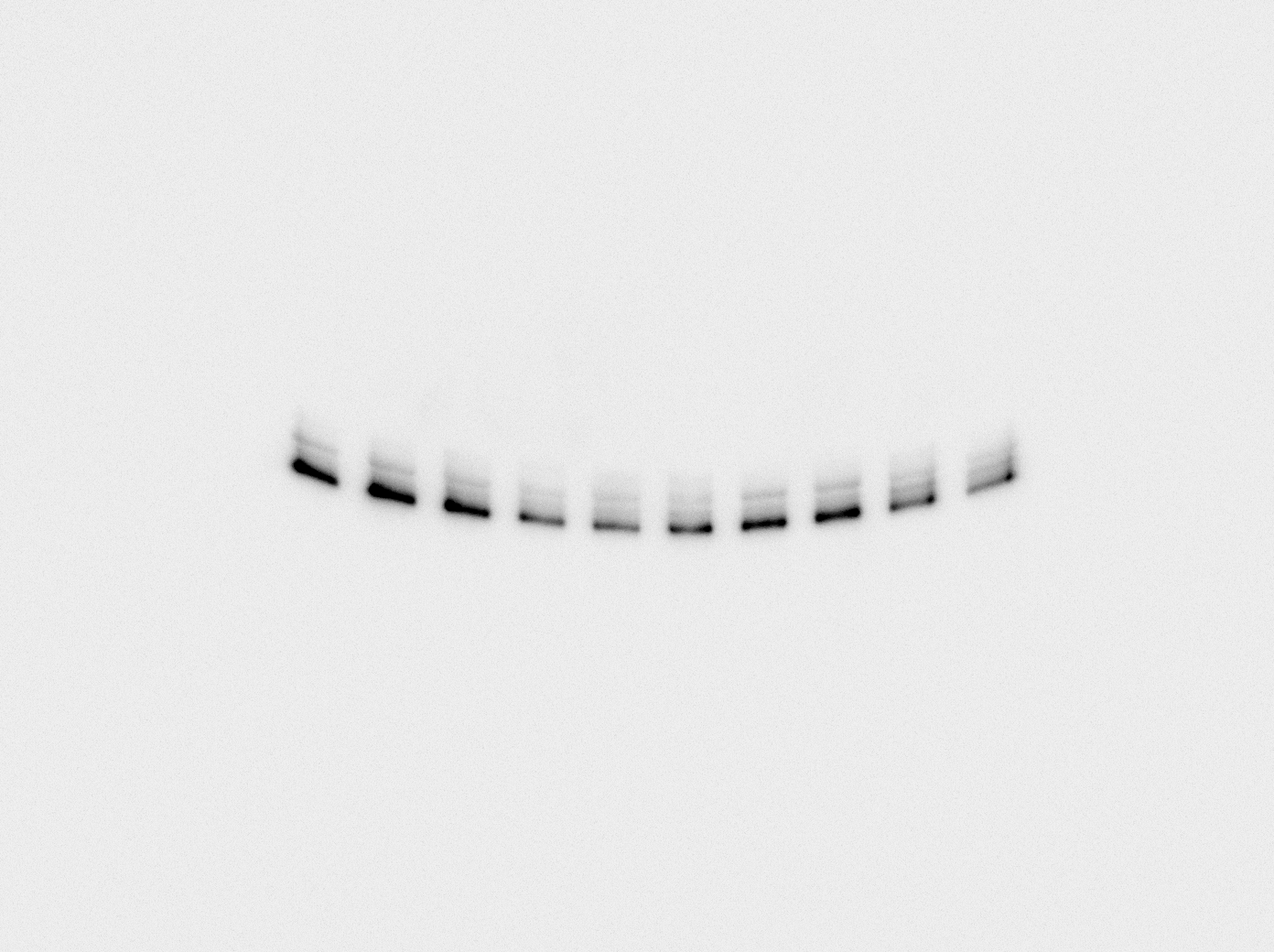

Supplement: Supplementary file 1 [file cancers-13-00862-s001.zip › WBdata_cancers/20201009_TCO1_totalERK_E7080/20201009_TCO1_totalERK_E7080_a.tif]

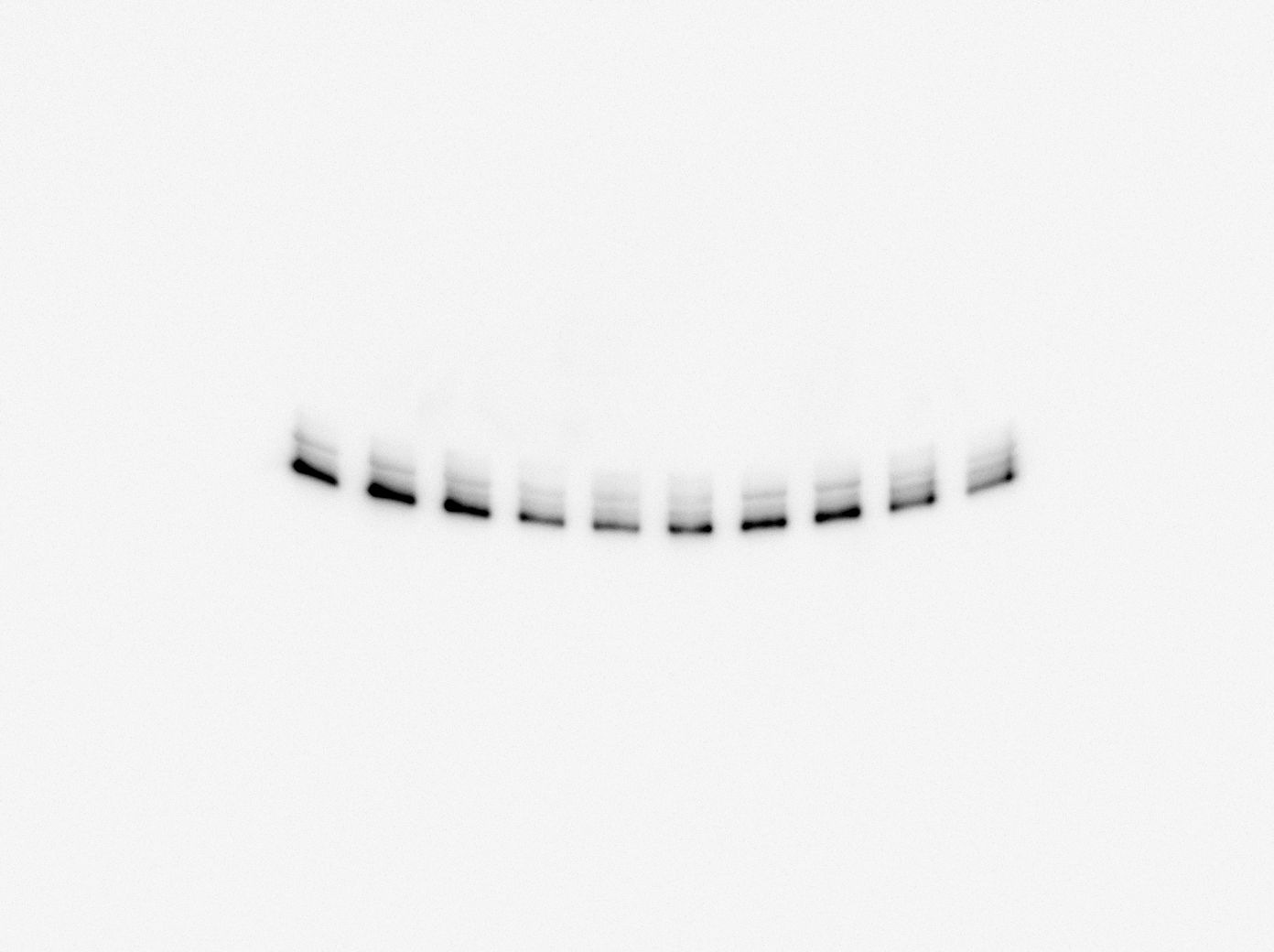

Supplement: Supplementary file 1 [file cancers-13-00862-s001.zip › WBdata_cancers/20201009_TCO1_totalERK_E7080/20201009_TCO1_totalERK_E7080_b.tif]

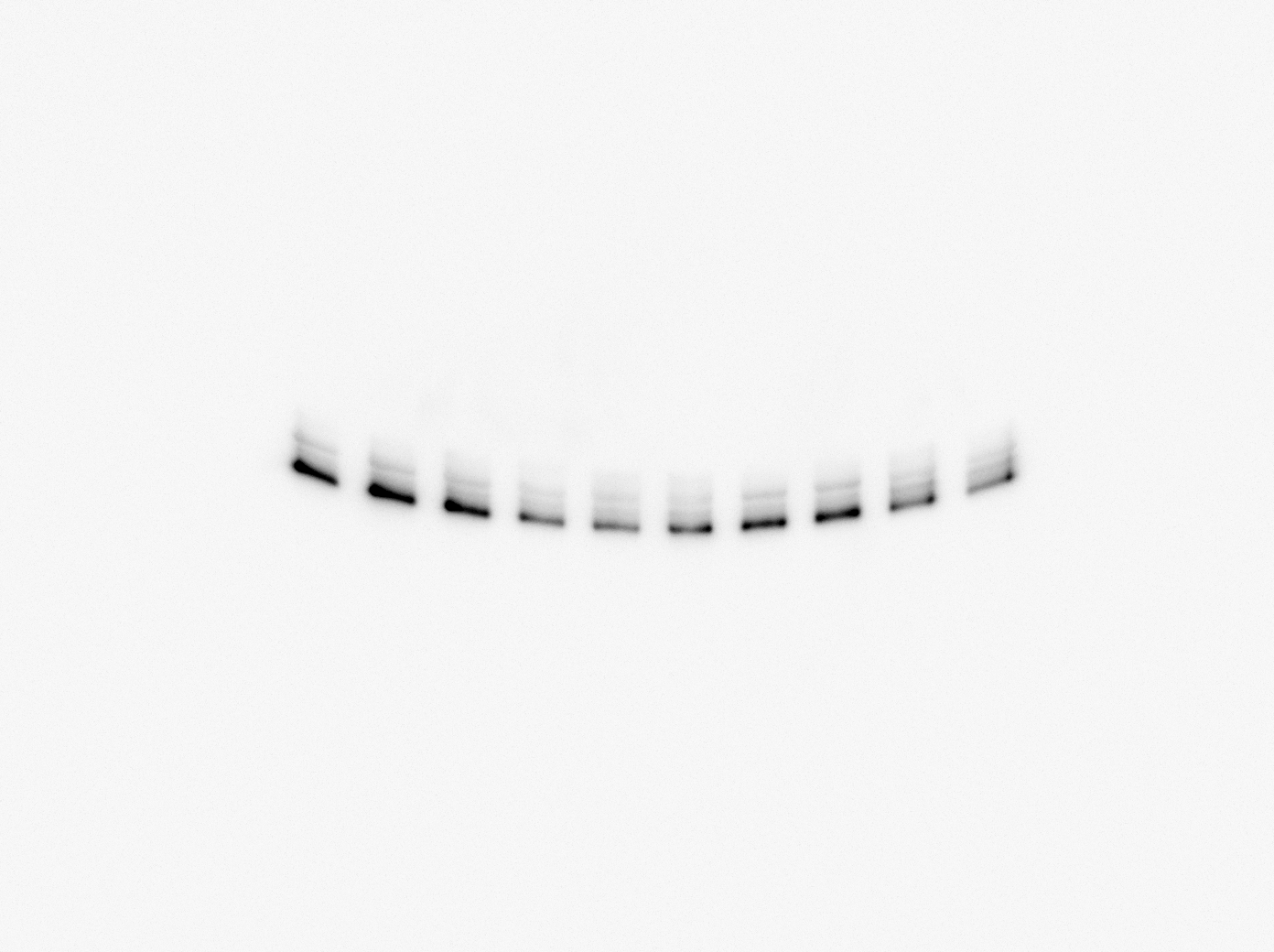

Supplement: Supplementary file 1 [file cancers-13-00862-s001.zip › WBdata_cancers/20201009_TCO1_totalERK_E7080/20201009_TCO1_totalERK_E7080_c.tif]

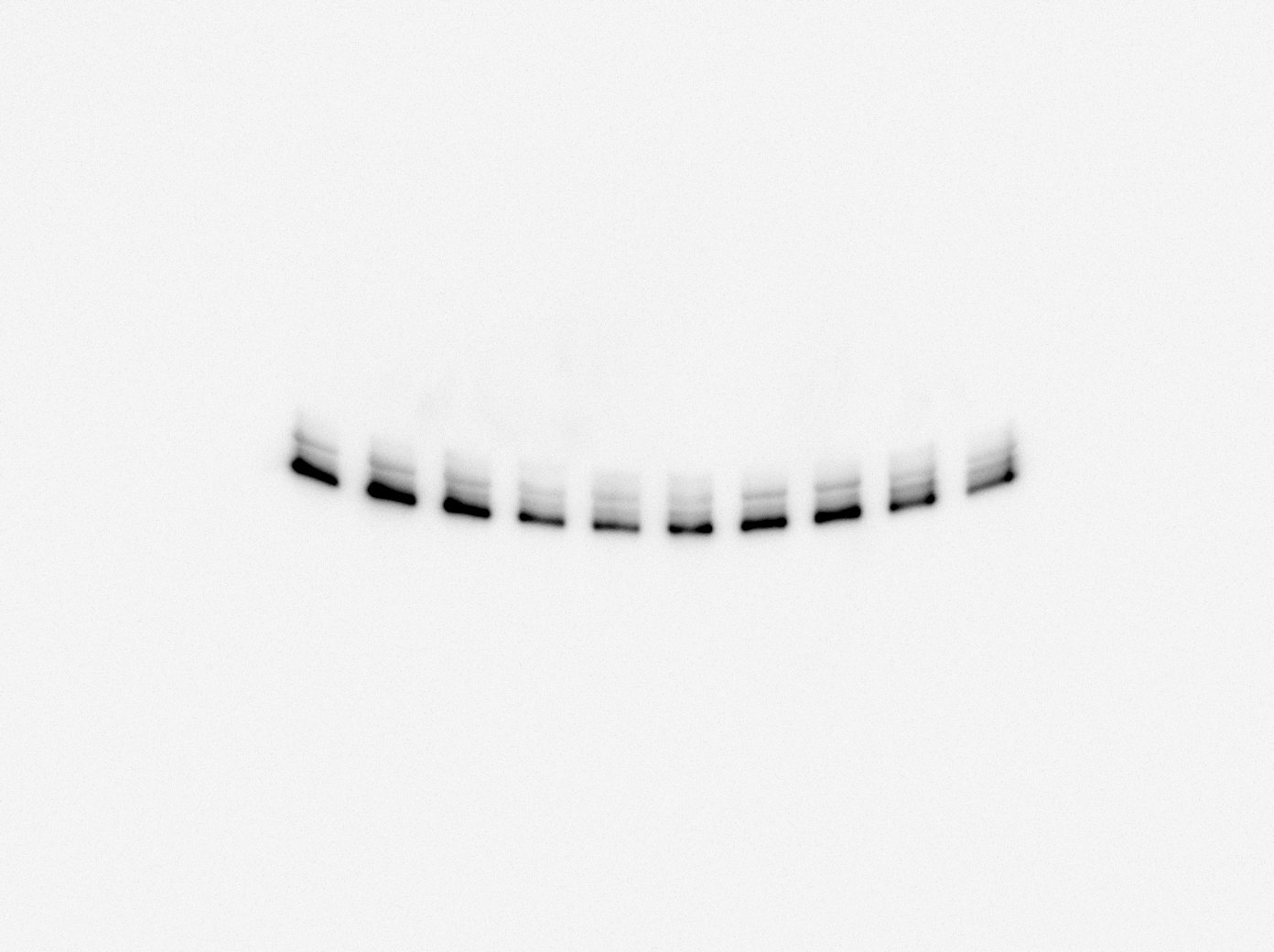

Supplement: Supplementary file 1 [file cancers-13-00862-s001.zip › WBdata_cancers/20201009_TCO1_totalERK_E7080/20201009_TCO1_totalERK_E7080_d.tif]

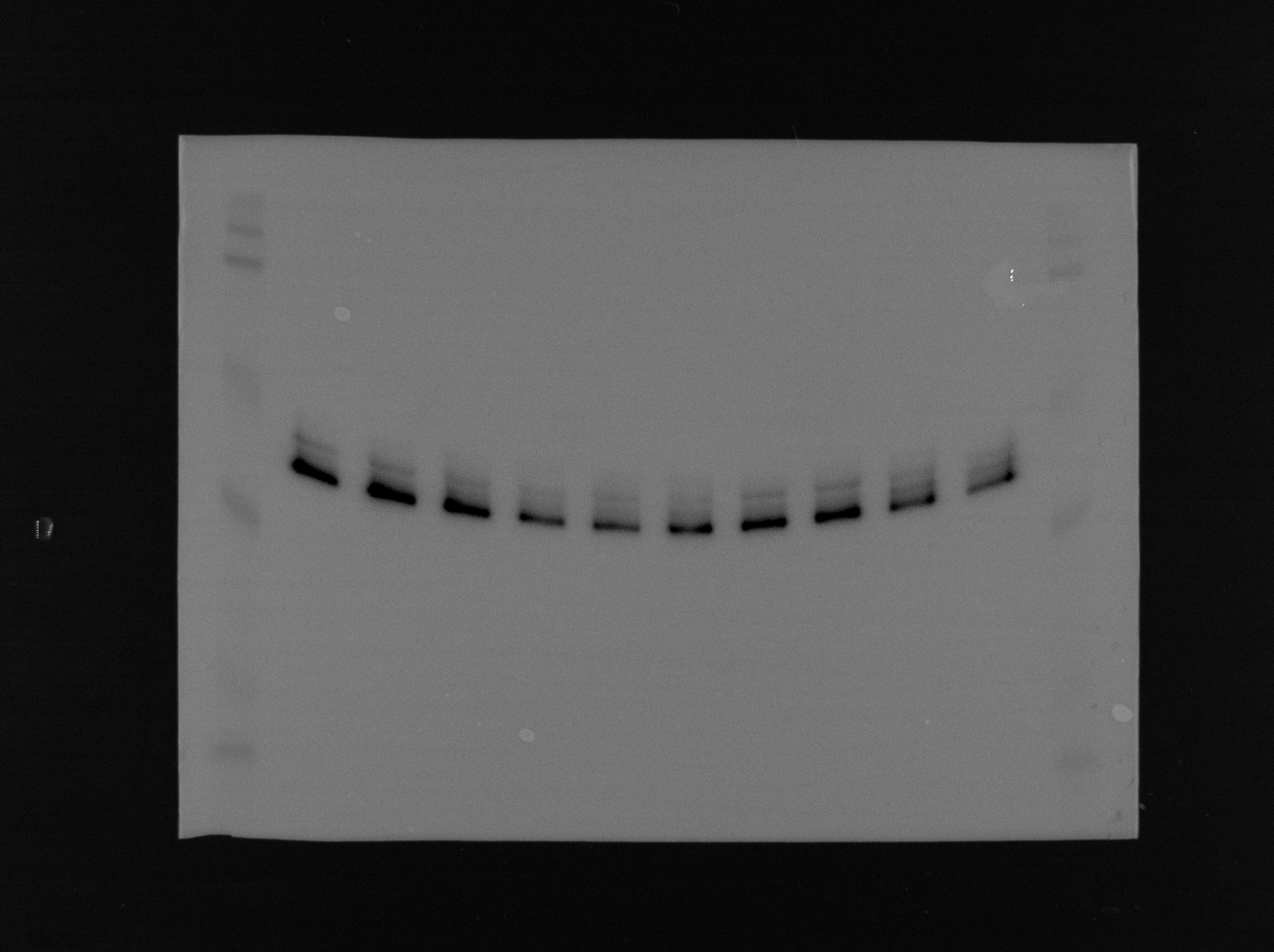

Supplement: Supplementary file 1 [file cancers-13-00862-s001.zip › WBdata_cancers/20201009_TCO1_totalERK_E7080/20201009_TCO1_totalERK_E7080_Merge.tif]

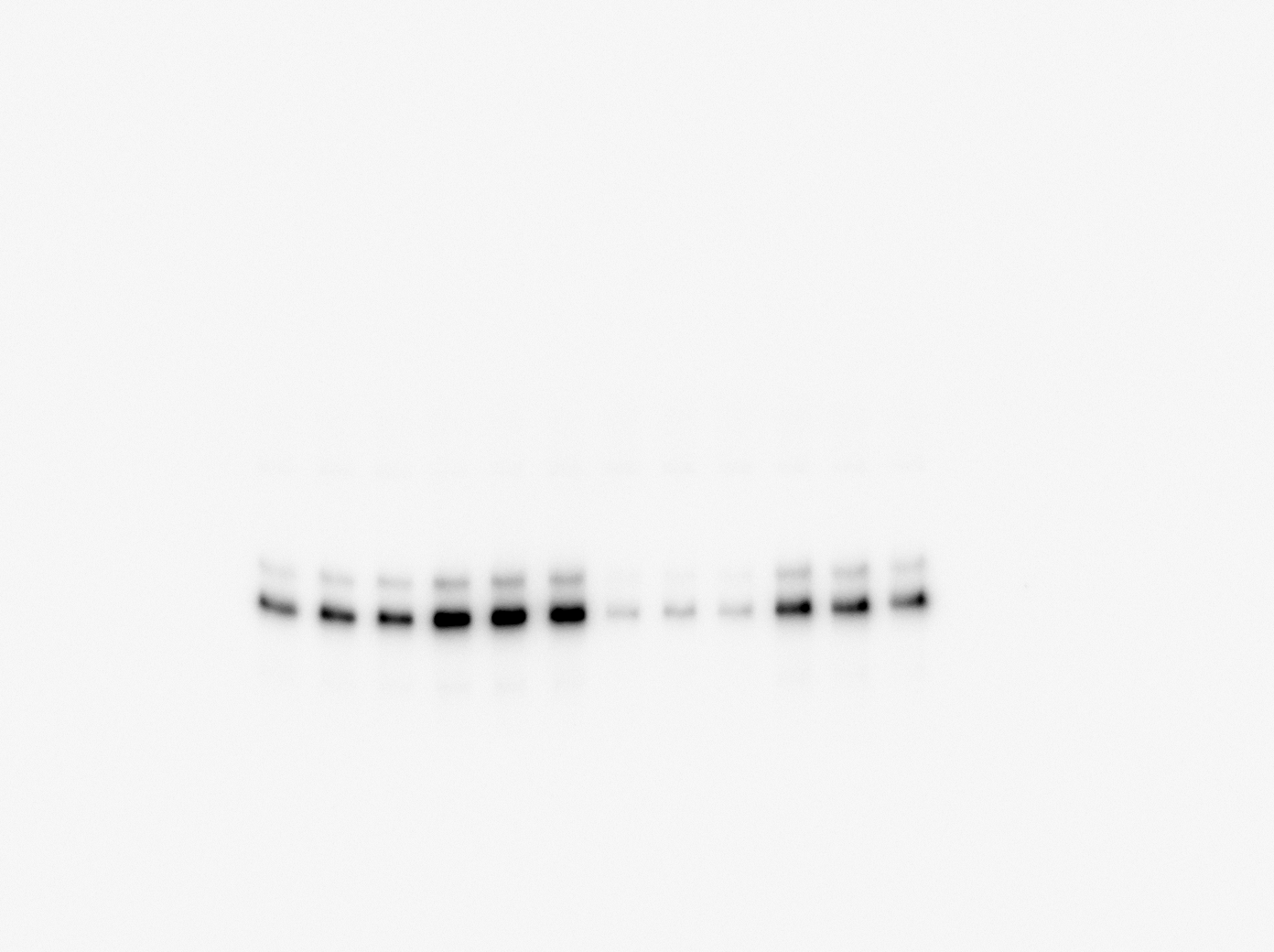

Supplement: Supplementary file 1 [file cancers-13-00862-s001.zip › WBdata_cancers/20201119_TCO1_Mix_pERK/201119_TCO1_Mix_pERK_a.tif]

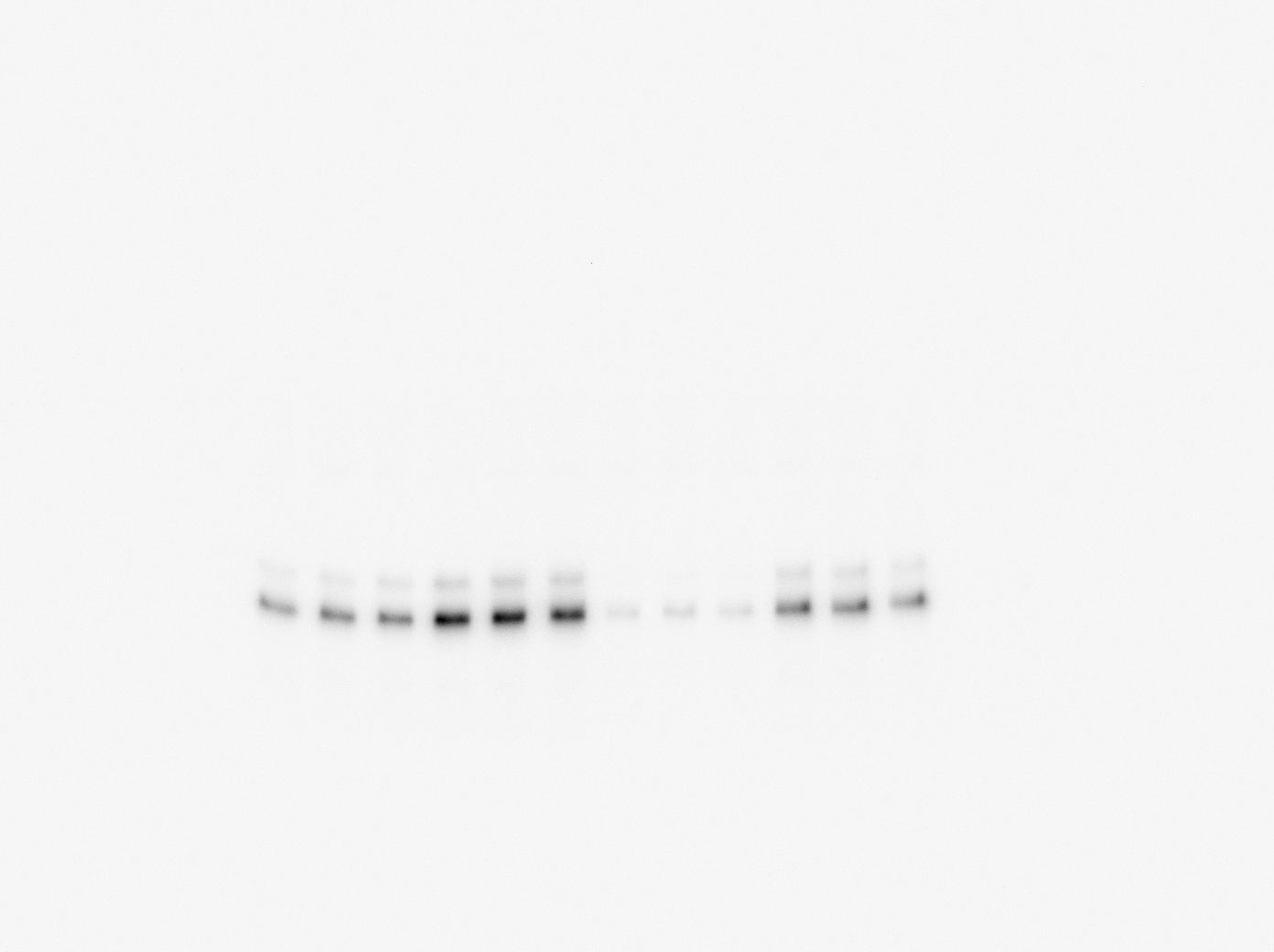

Supplement: Supplementary file 1 [file cancers-13-00862-s001.zip › WBdata_cancers/20201119_TCO1_Mix_pERK/201119_TCO1_Mix_pERK_b.tif]

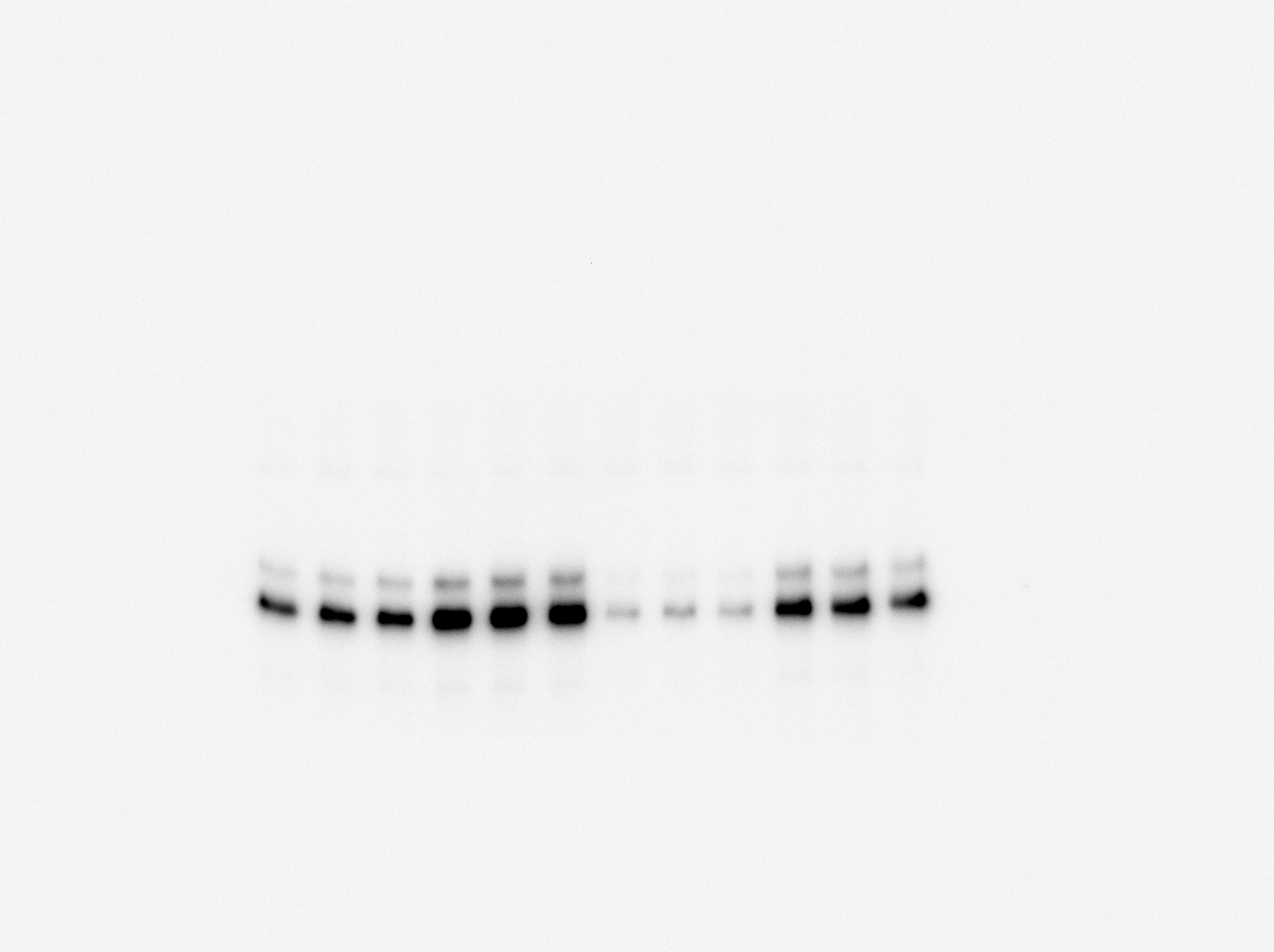

Supplement: Supplementary file 1 [file cancers-13-00862-s001.zip › WBdata_cancers/20201119_TCO1_Mix_pERK/201119_TCO1_Mix_pERK_b_2.tif]

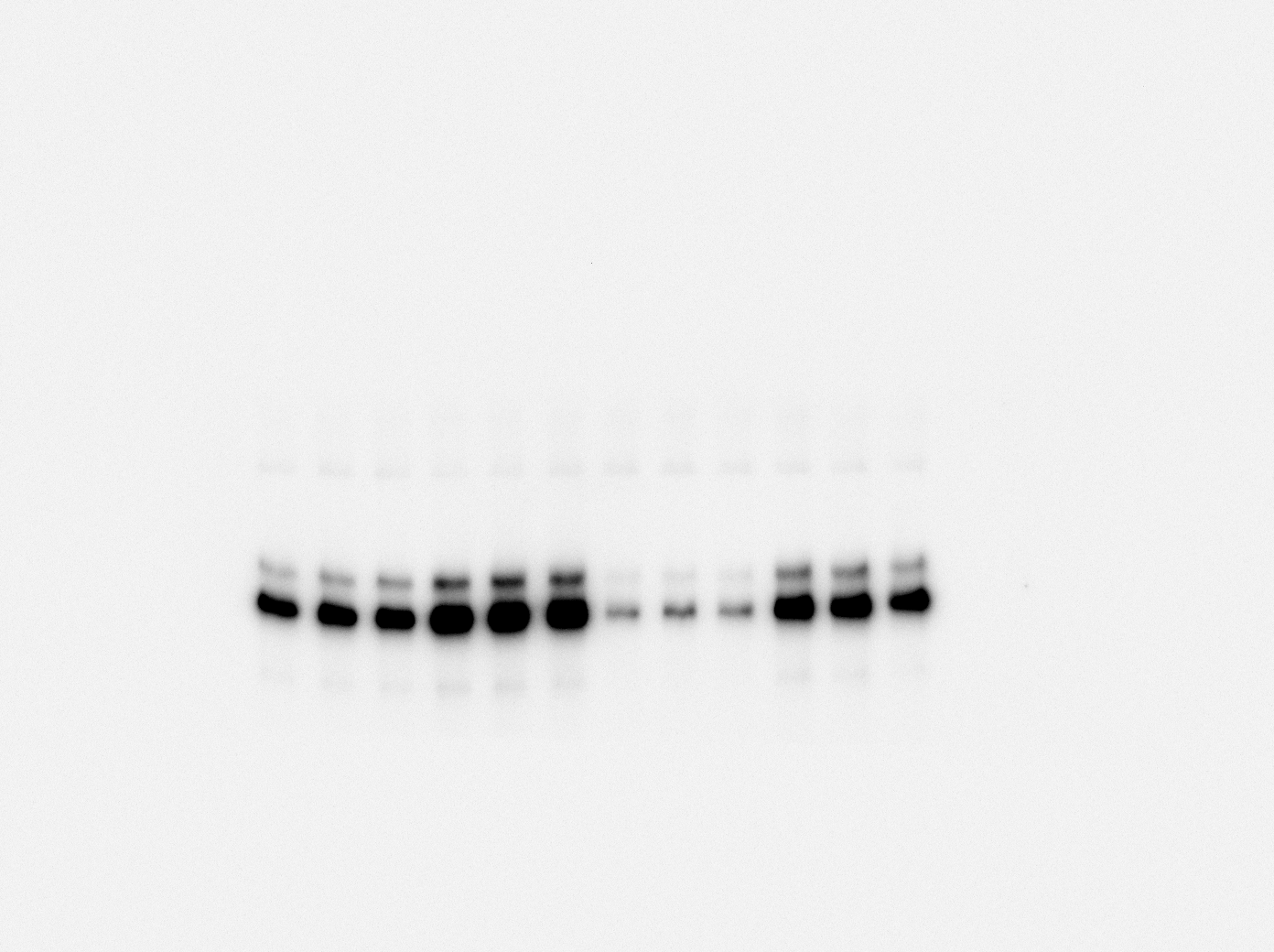

Supplement: Supplementary file 1 [file cancers-13-00862-s001.zip › WBdata_cancers/20201119_TCO1_Mix_pERK/201119_TCO1_Mix_pERK_d.tif]

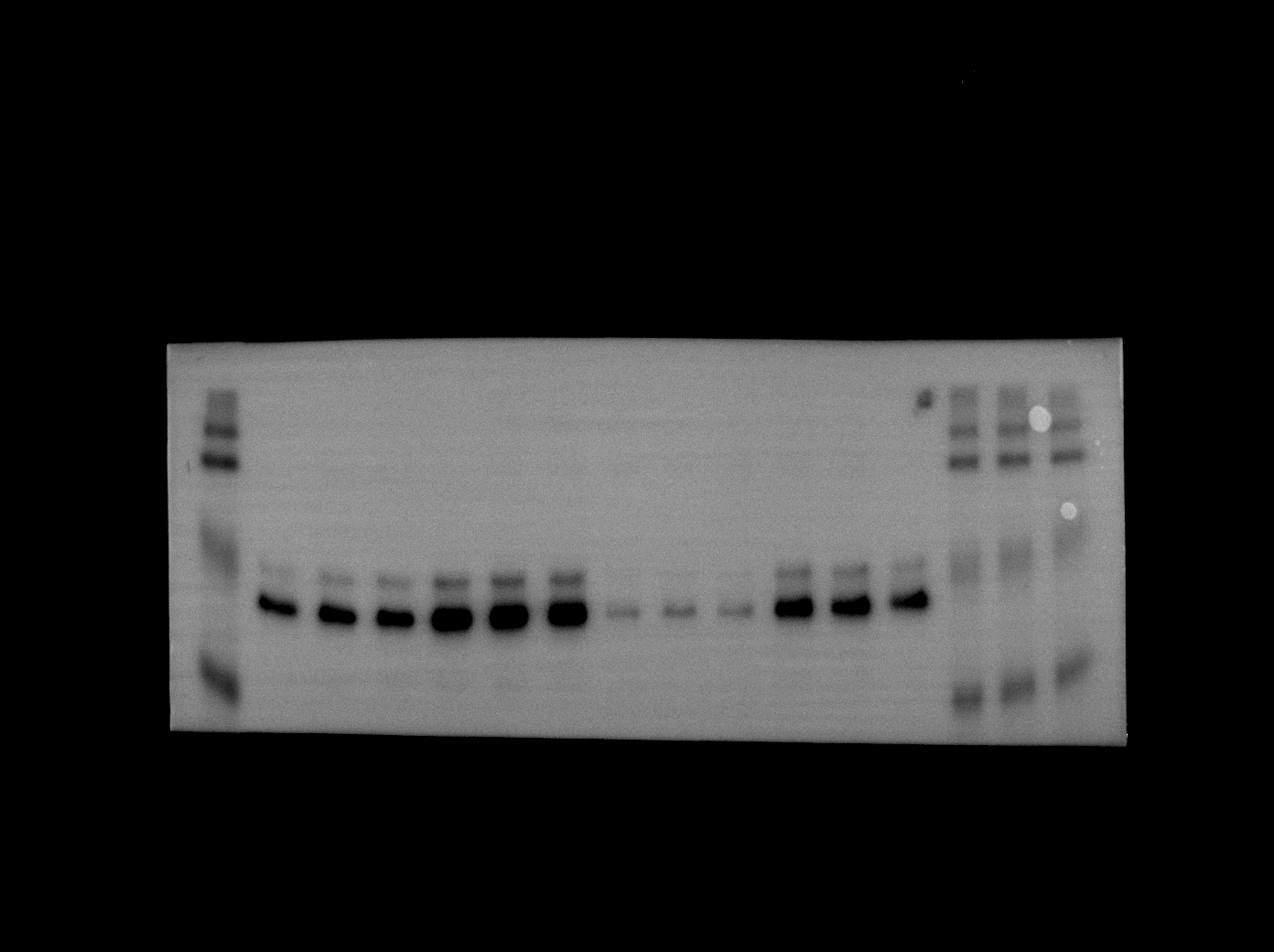

Supplement: Supplementary file 1 [file cancers-13-00862-s001.zip › WBdata_cancers/20201119_TCO1_Mix_pERK/201119_TCO1_Mix_pERK_Merge.tif]
