# Supplementary figures and images for: Synergistic Effects of Lenvatinib (E7080) and MEK Inhibitors against Anaplastic Thyroid Cancer in Preclinical Models (part 1 of 2)
Source: Cancers (Basel). 2021 Feb 18;13(4):862. doi: 10.3390/cancers13040862 (PMC7922355; doi:10.3390/cancers13040862)

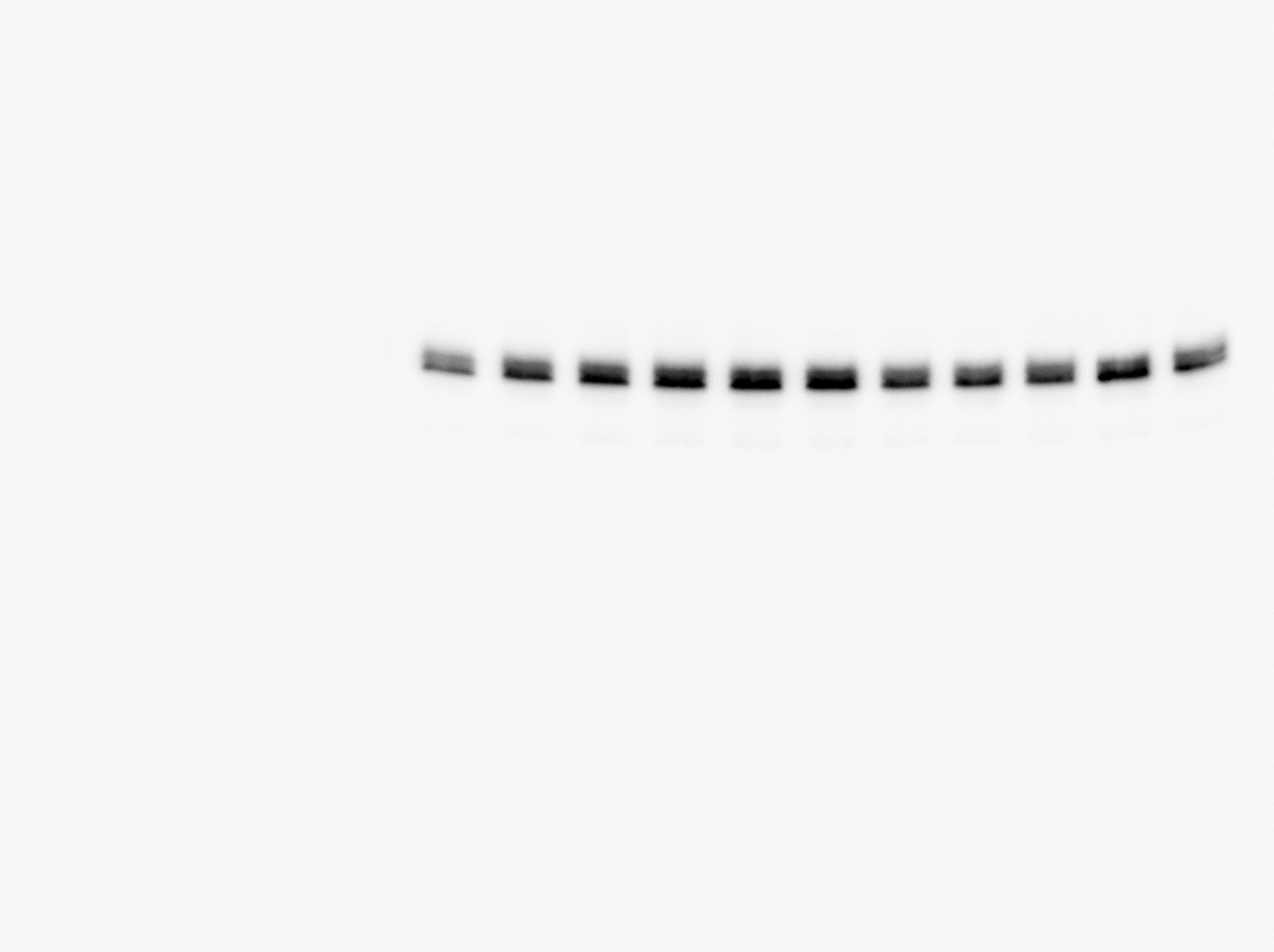

Supplement: Supplementary file 1 [file cancers-13-00862-s001.zip › WBdata_cancers/200114_8505C_E7080_pERK/200114_8505C_E7080_pERK.tif]

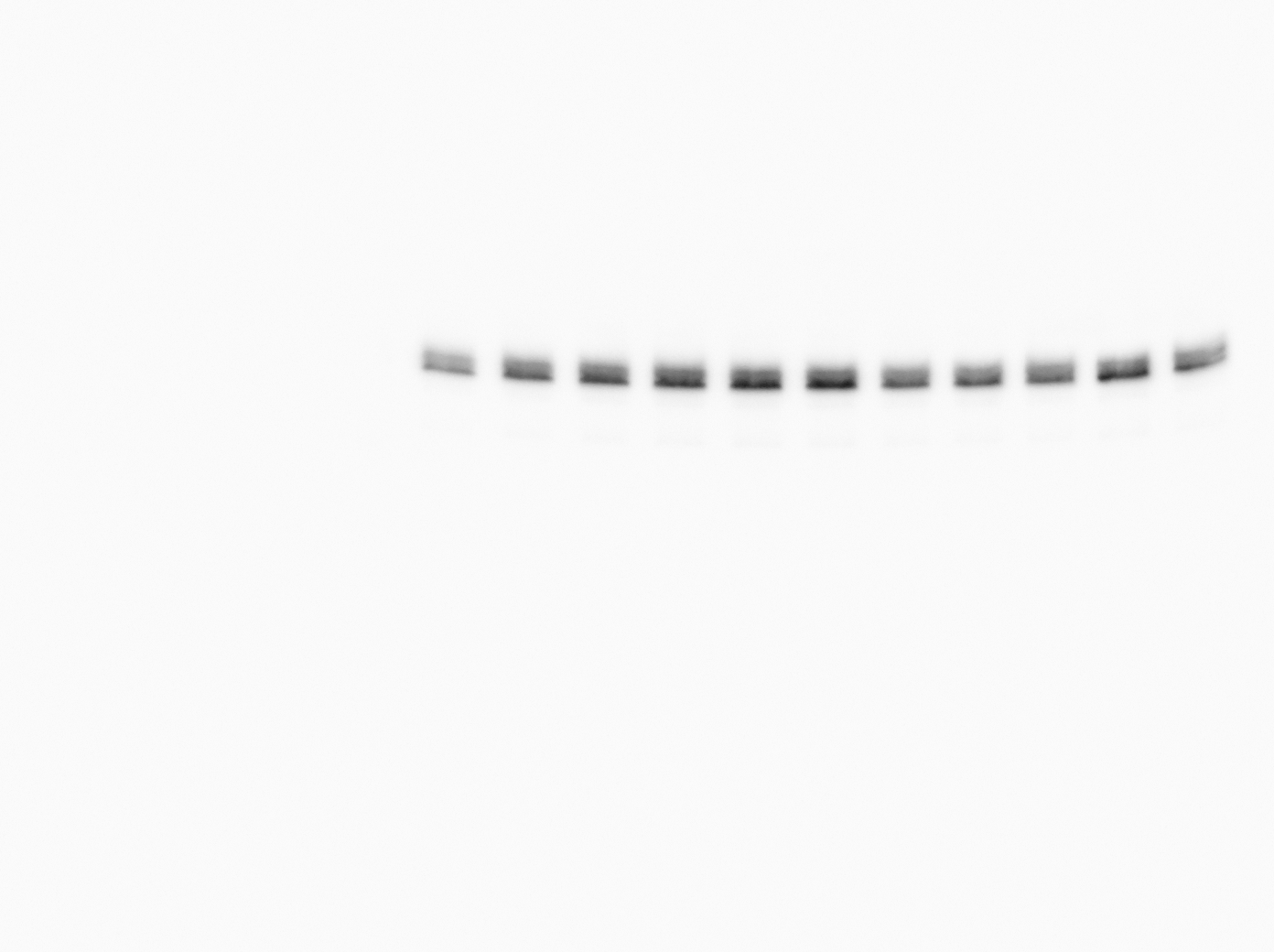

Supplement: Supplementary file 1 [file cancers-13-00862-s001.zip › WBdata_cancers/200114_8505C_E7080_pERK/200114_8505C_E7080_pERK_b.tif]

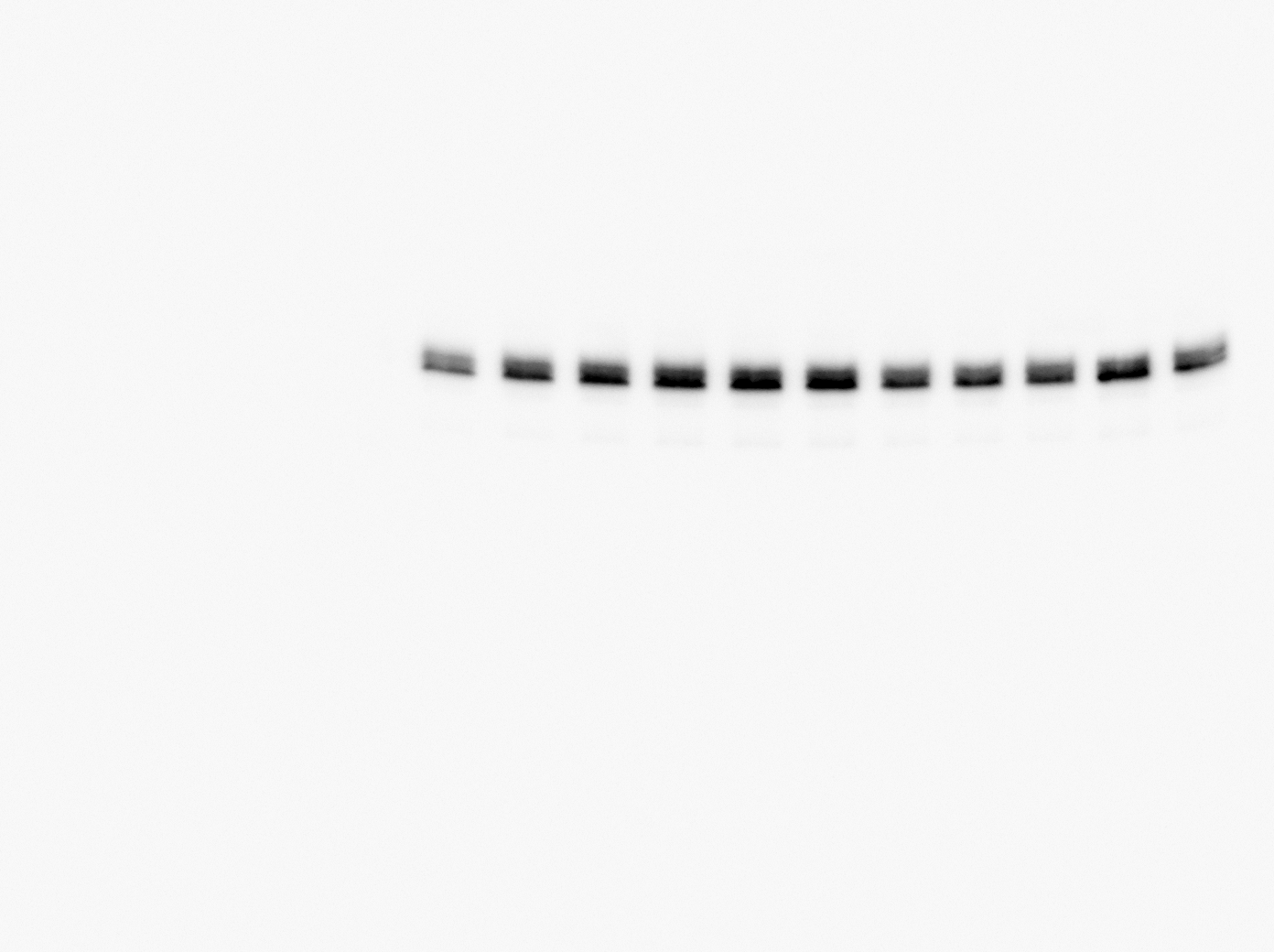

Supplement: Supplementary file 1 [file cancers-13-00862-s001.zip › WBdata_cancers/200114_8505C_E7080_pERK/200114_8505C_E7080_pERK_c.tif]

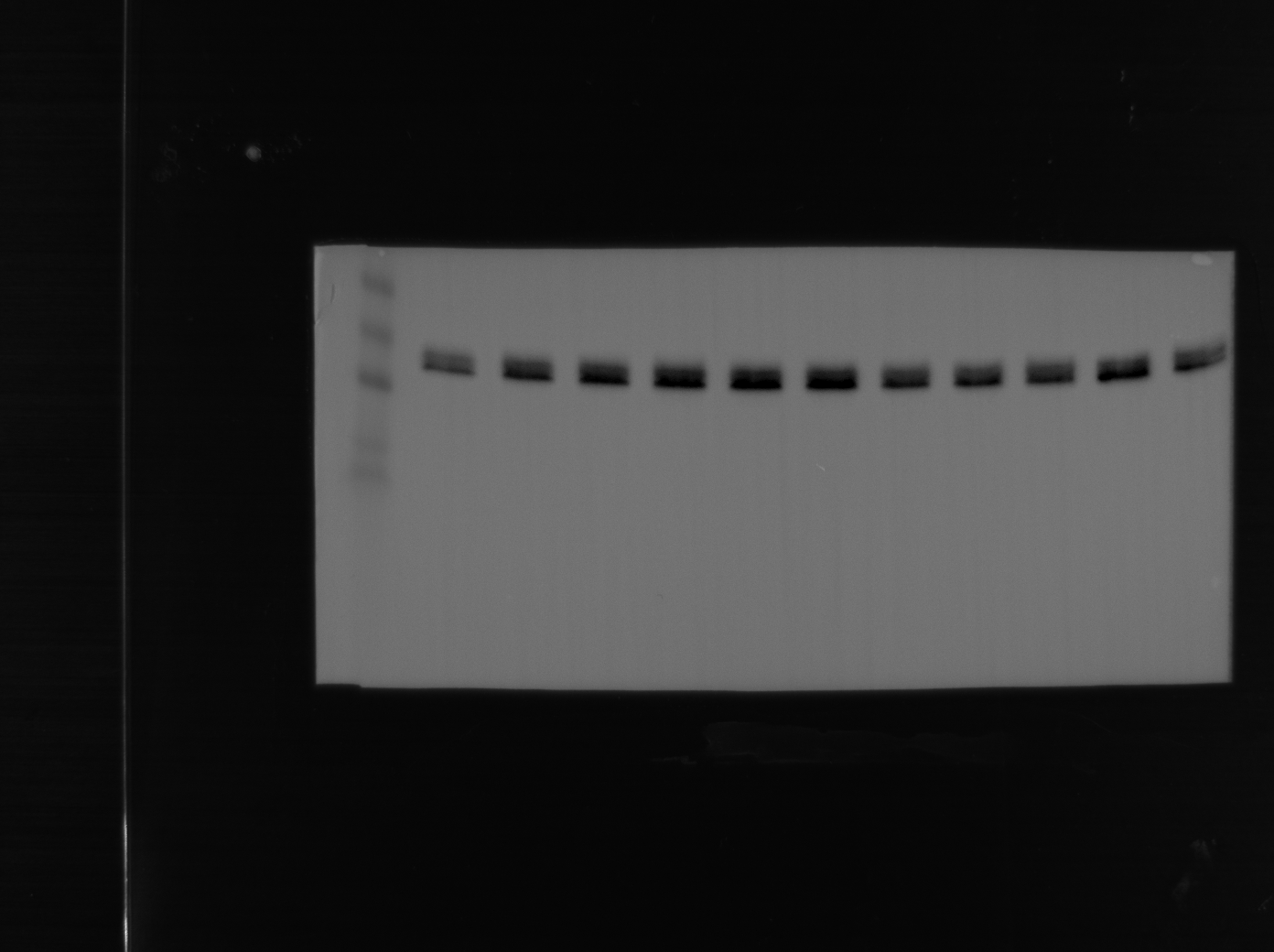

Supplement: Supplementary file 1 [file cancers-13-00862-s001.zip › WBdata_cancers/200114_8505C_E7080_pERK/200114_8505C_E7080_pERK_merge.tif]

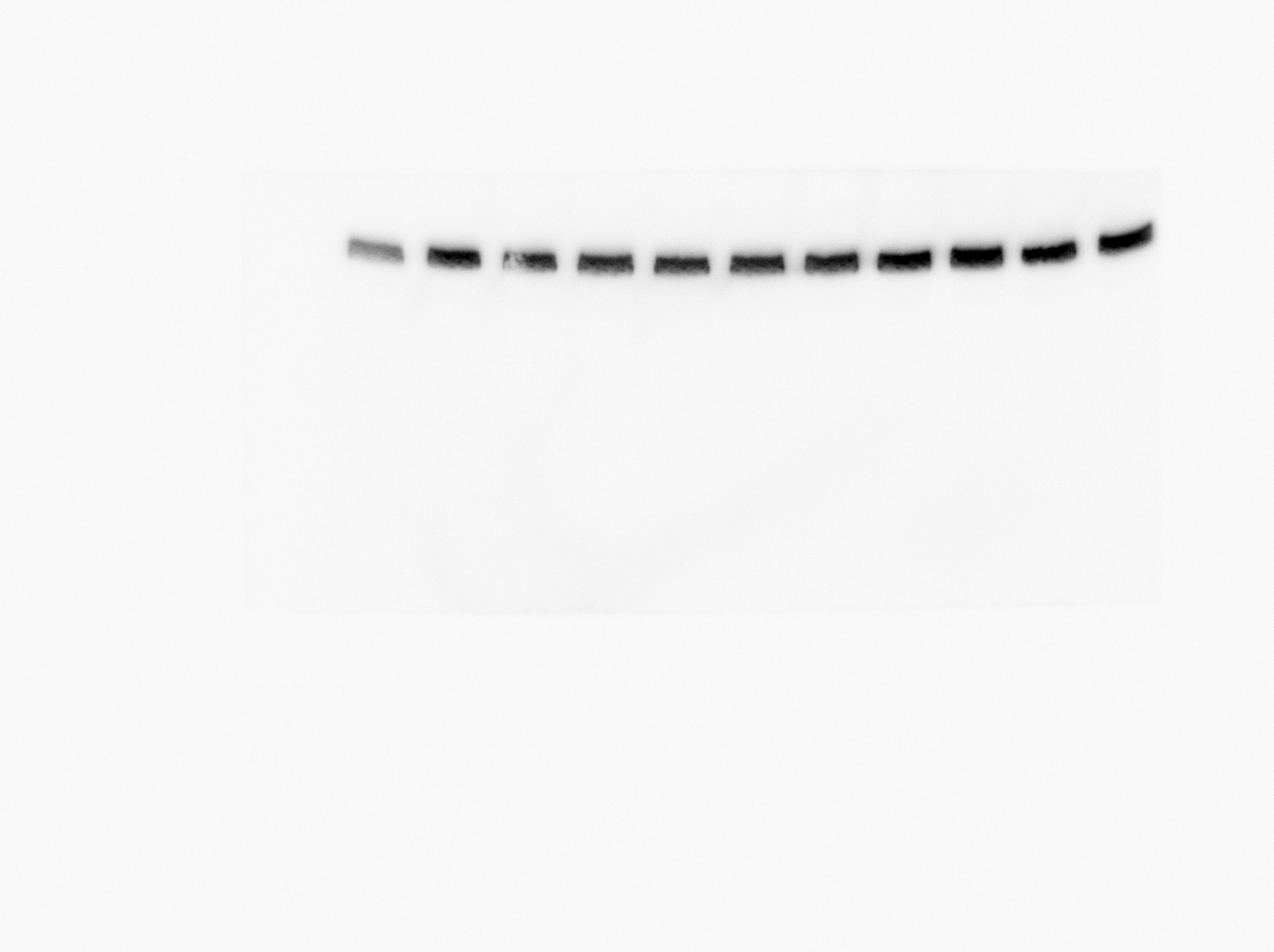

Supplement: Supplementary file 1 [file cancers-13-00862-s001.zip › WBdata_cancers/200115_8505C_E7080_aTub/200115_8505C_E7080_aTub_a.tif]

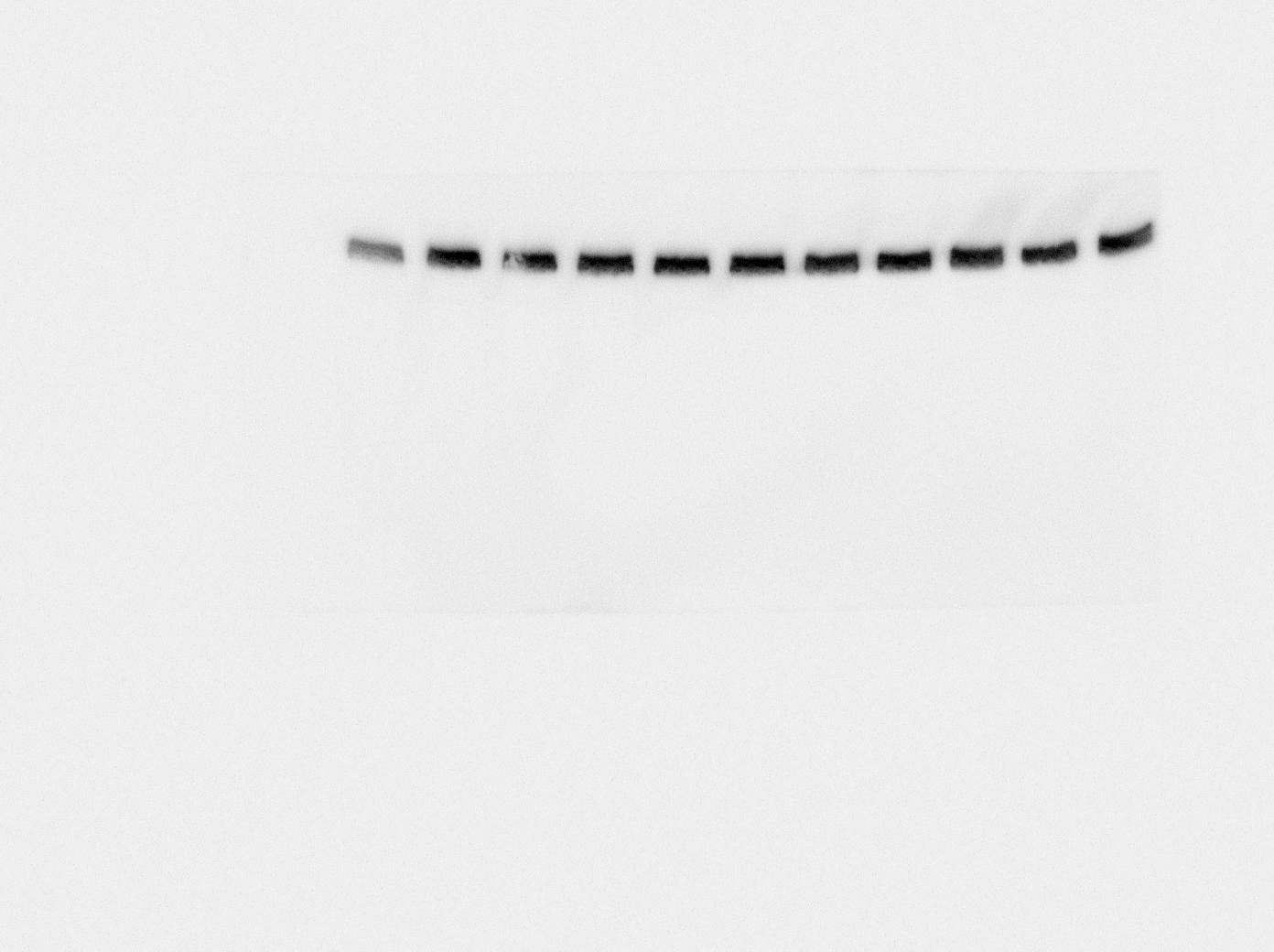

Supplement: Supplementary file 1 [file cancers-13-00862-s001.zip › WBdata_cancers/200115_8505C_E7080_aTub/200115_8505C_E7080_aTub_b.tif]

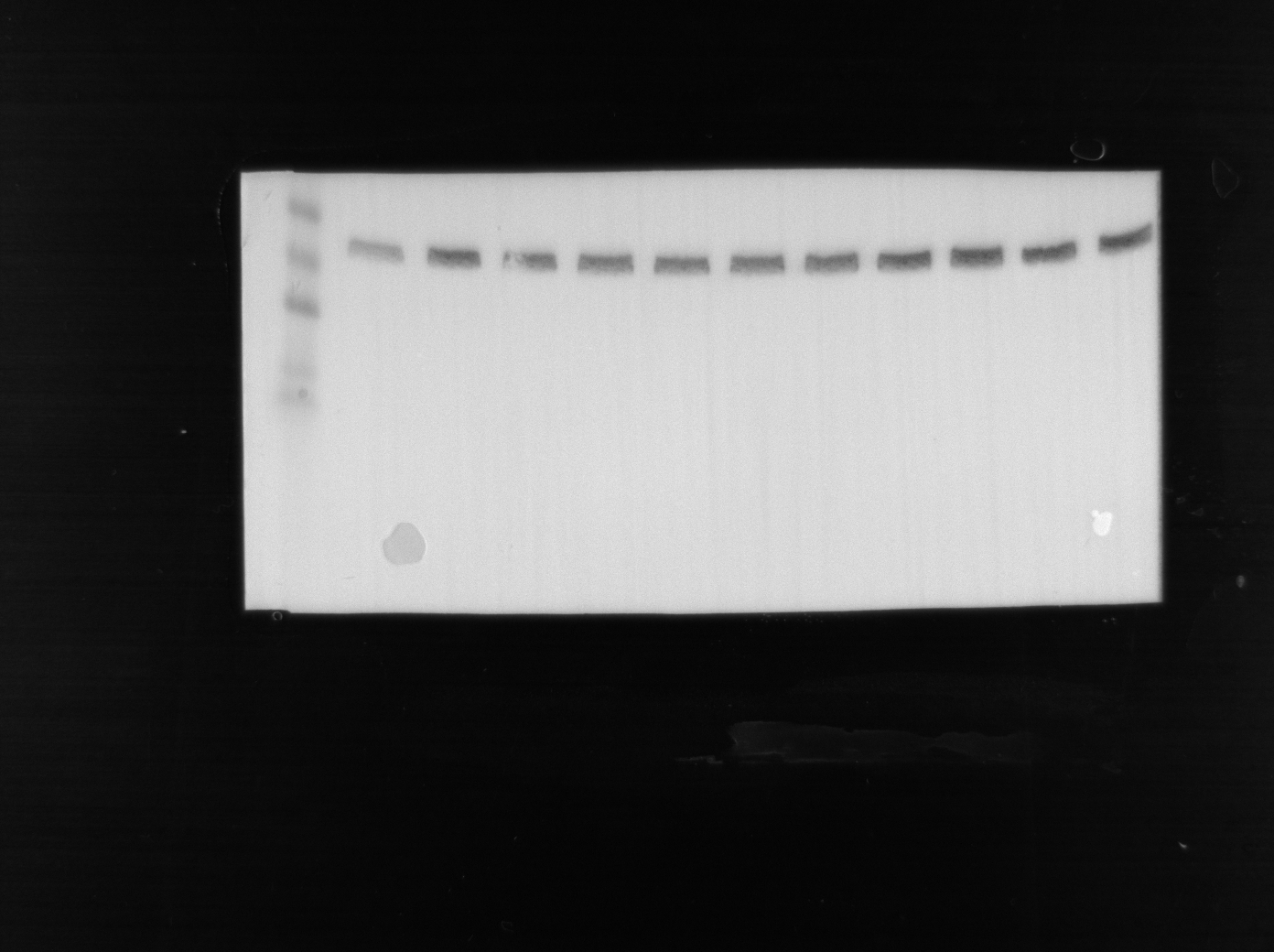

Supplement: Supplementary file 1 [file cancers-13-00862-s001.zip › WBdata_cancers/200115_8505C_E7080_aTub/200115_8505C_E7080_aTub_Merge.tif]

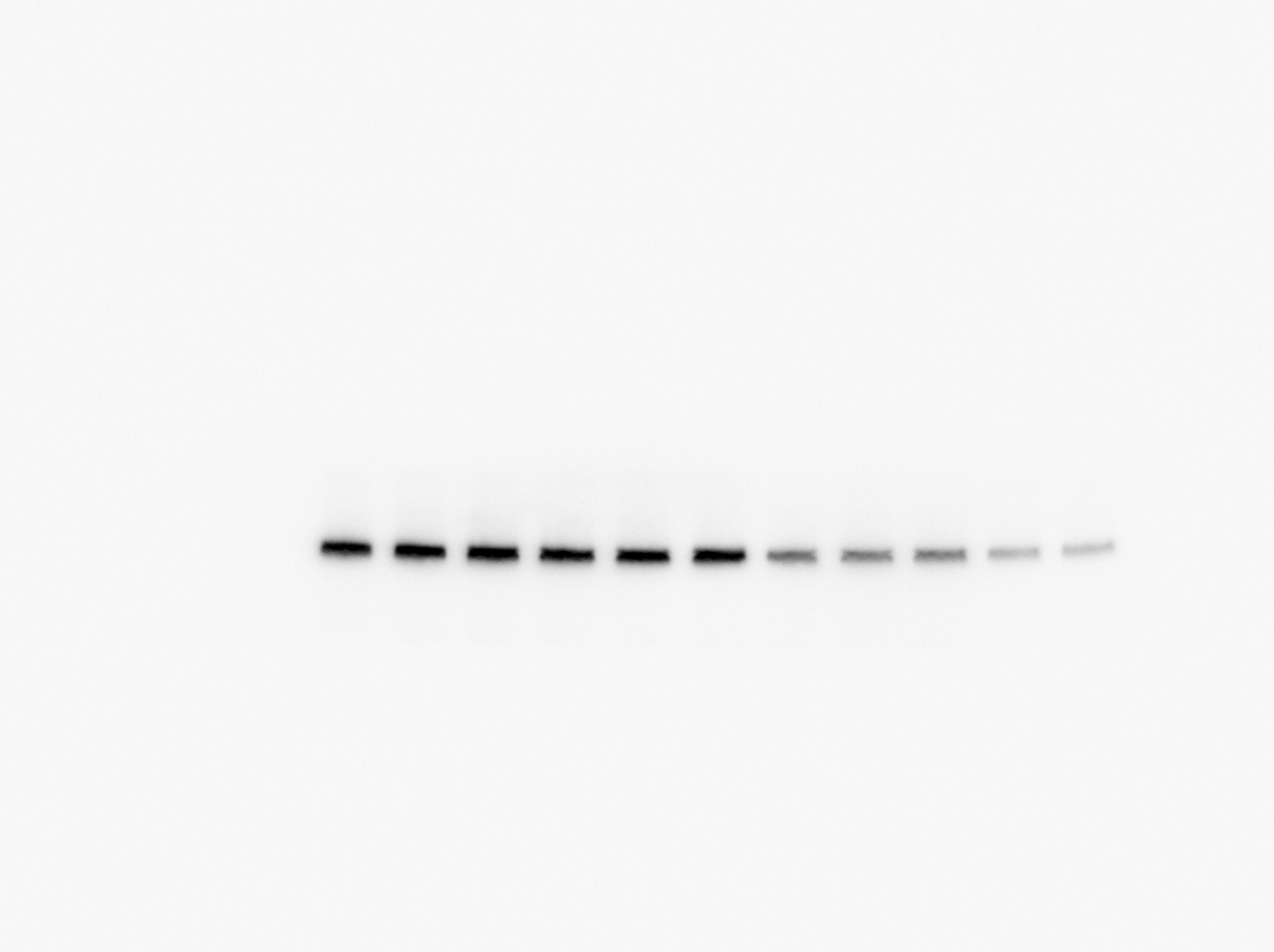

Supplement: Supplementary file 1 [file cancers-13-00862-s001.zip › WBdata_cancers/200117_8505C_E7080_pAKT/200117_8505C_E7080_pAKT_a.tif]

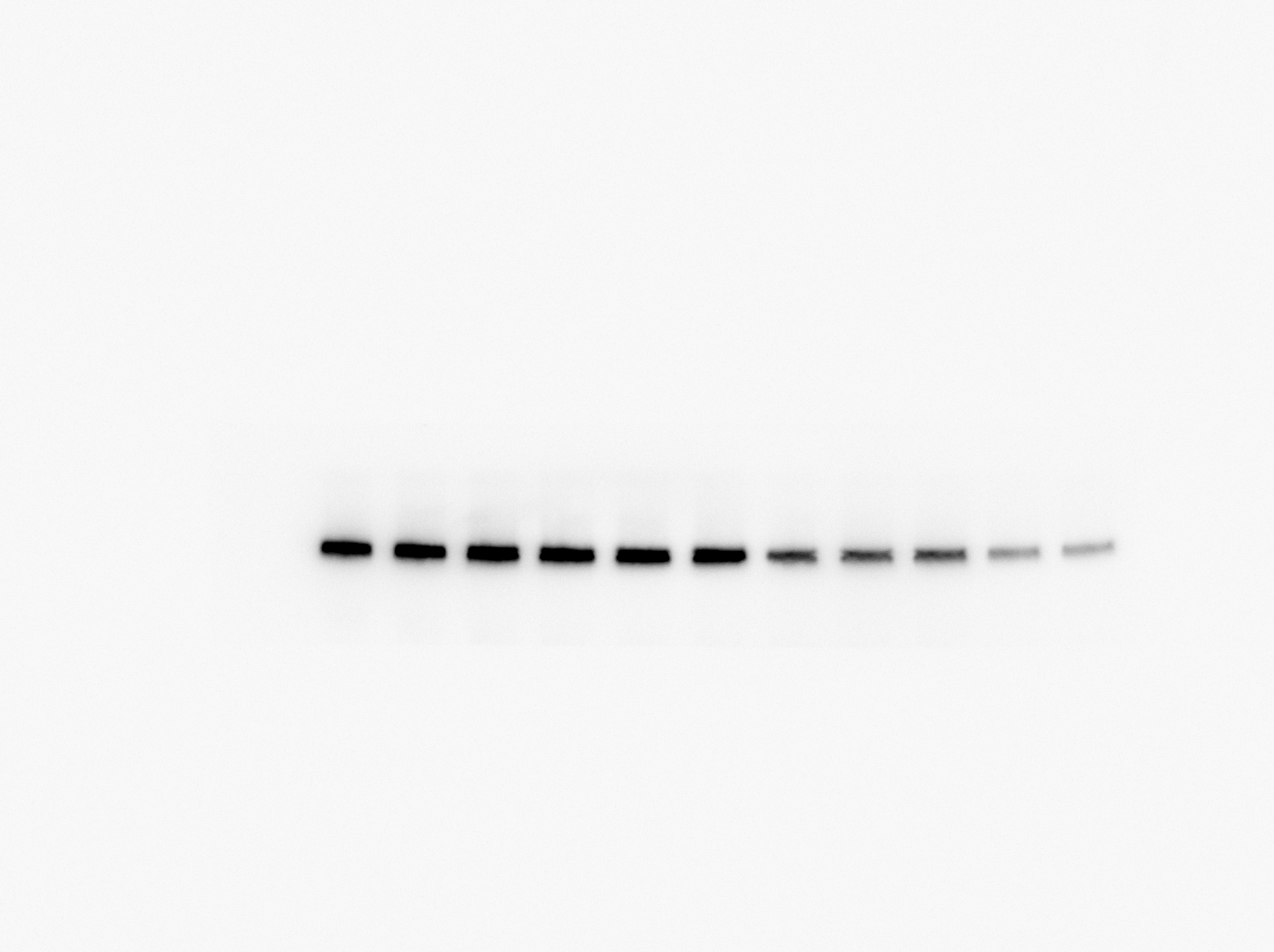

Supplement: Supplementary file 1 [file cancers-13-00862-s001.zip › WBdata_cancers/200117_8505C_E7080_pAKT/200117_8505C_E7080_pAKT_b.tif]

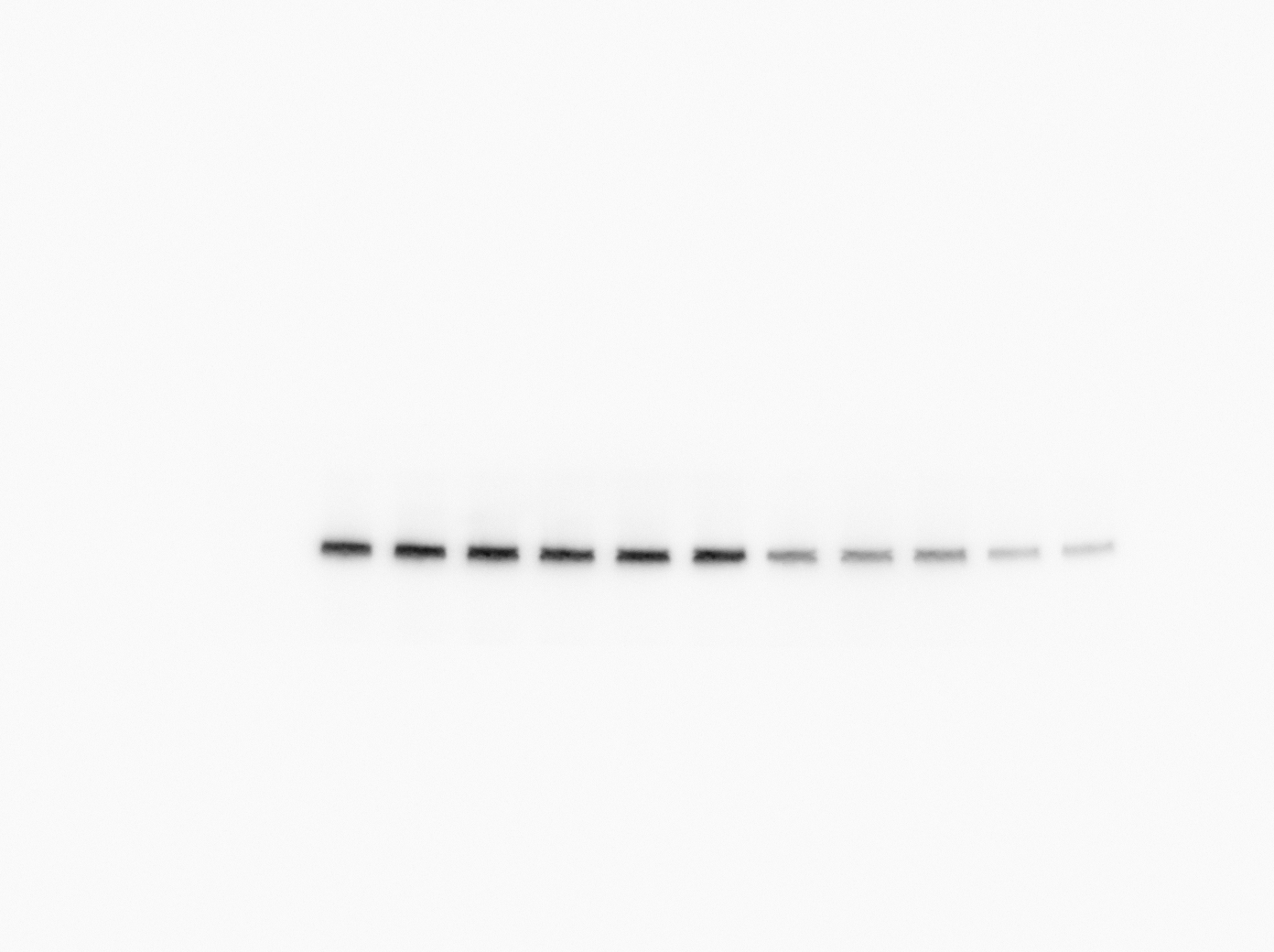

Supplement: Supplementary file 1 [file cancers-13-00862-s001.zip › WBdata_cancers/200117_8505C_E7080_pAKT/200117_8505C_E7080_pAKT_c.tif]

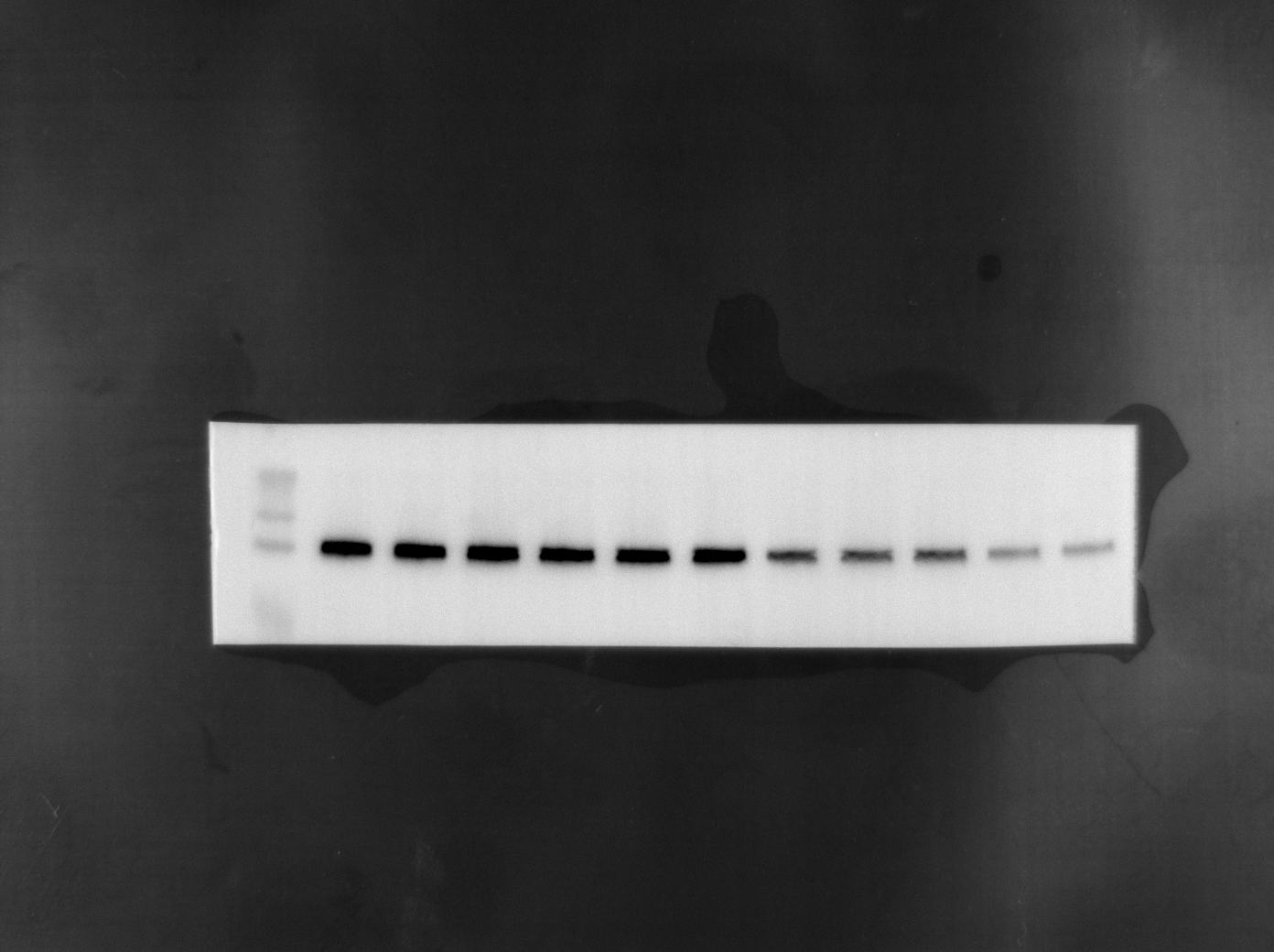

Supplement: Supplementary file 1 [file cancers-13-00862-s001.zip › WBdata_cancers/200117_8505C_E7080_pAKT/200117_8505C_E7080_pAKT_Merge.tif]

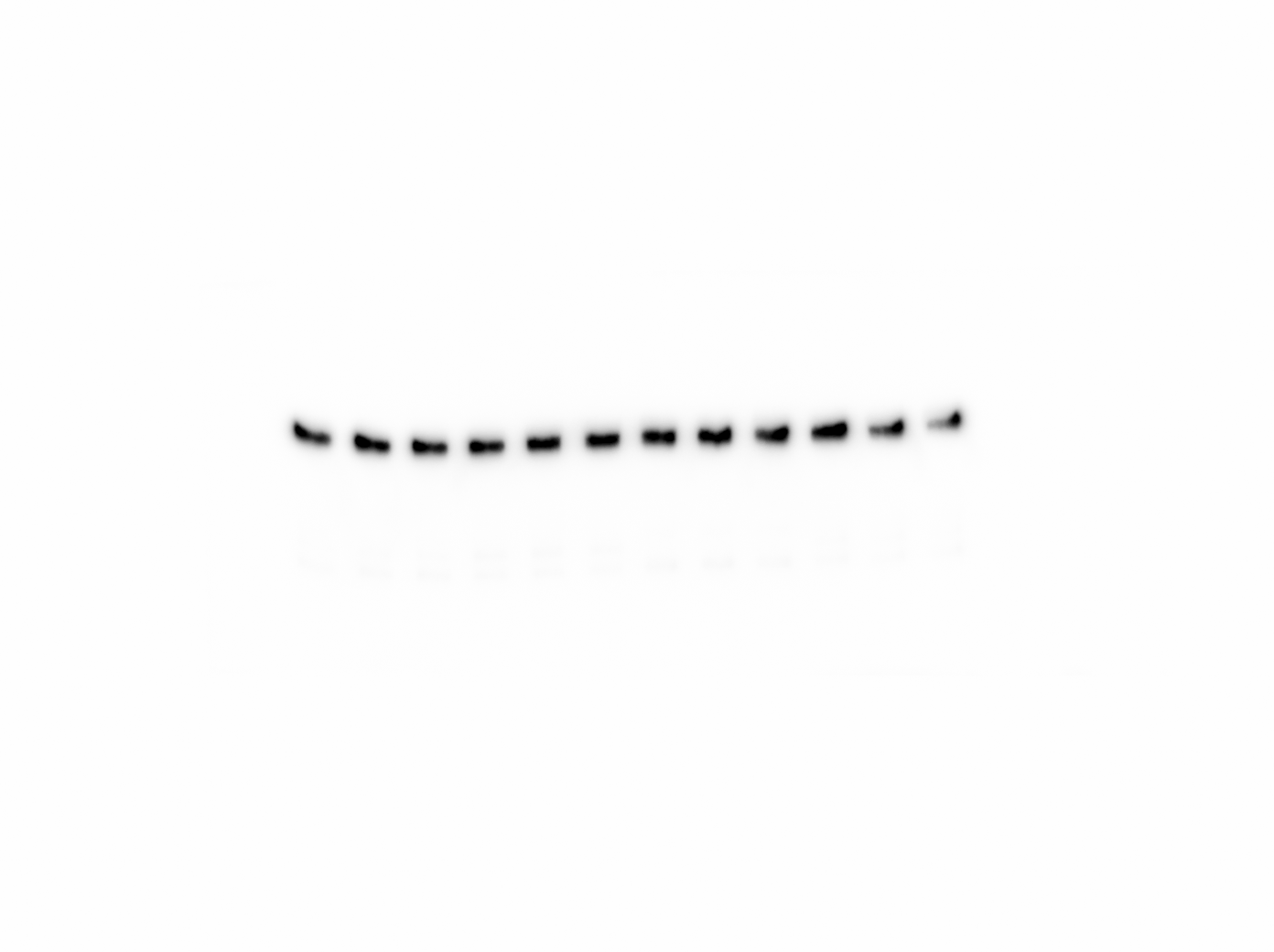

Supplement: Supplementary file 1 [file cancers-13-00862-s001.zip › WBdata_cancers/201120_TCO1_Mix_aTub/201120_TCO1_Mix_aTub_a.tif]

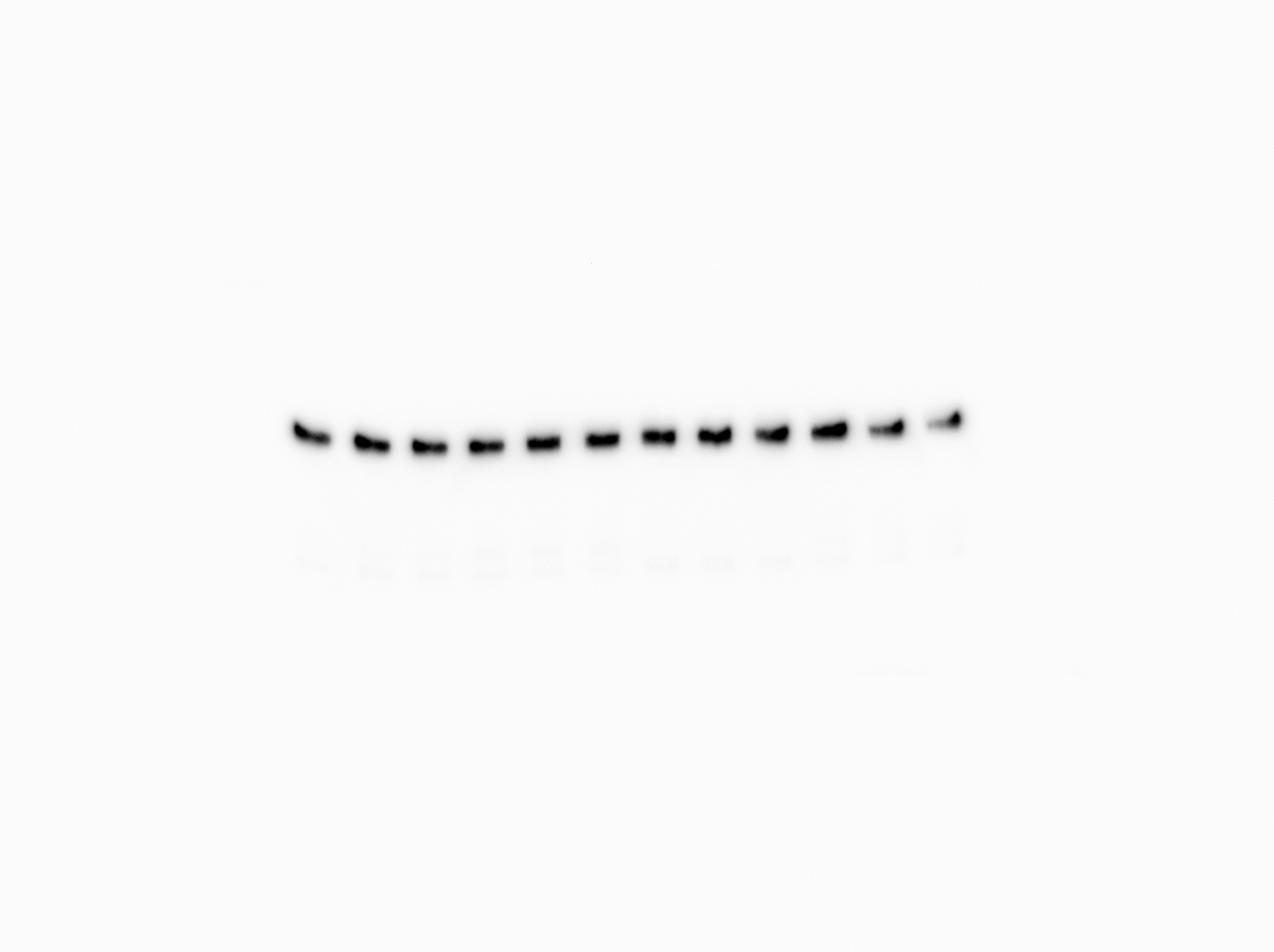

Supplement: Supplementary file 1 [file cancers-13-00862-s001.zip › WBdata_cancers/201120_TCO1_Mix_aTub/201120_TCO1_Mix_aTub_b.tif]

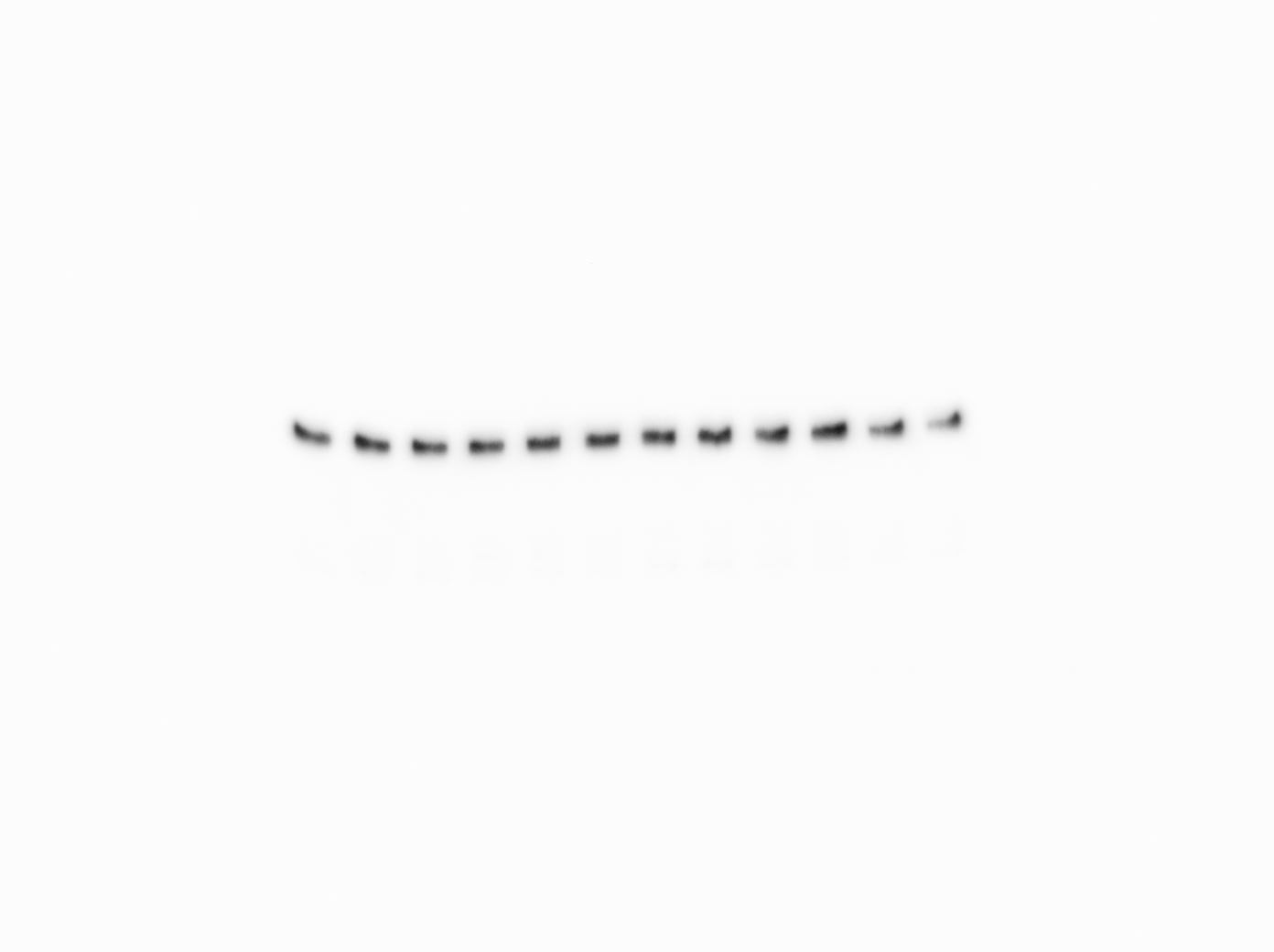

Supplement: Supplementary file 1 [file cancers-13-00862-s001.zip › WBdata_cancers/201120_TCO1_Mix_aTub/201120_TCO1_Mix_aTub_c.tif]

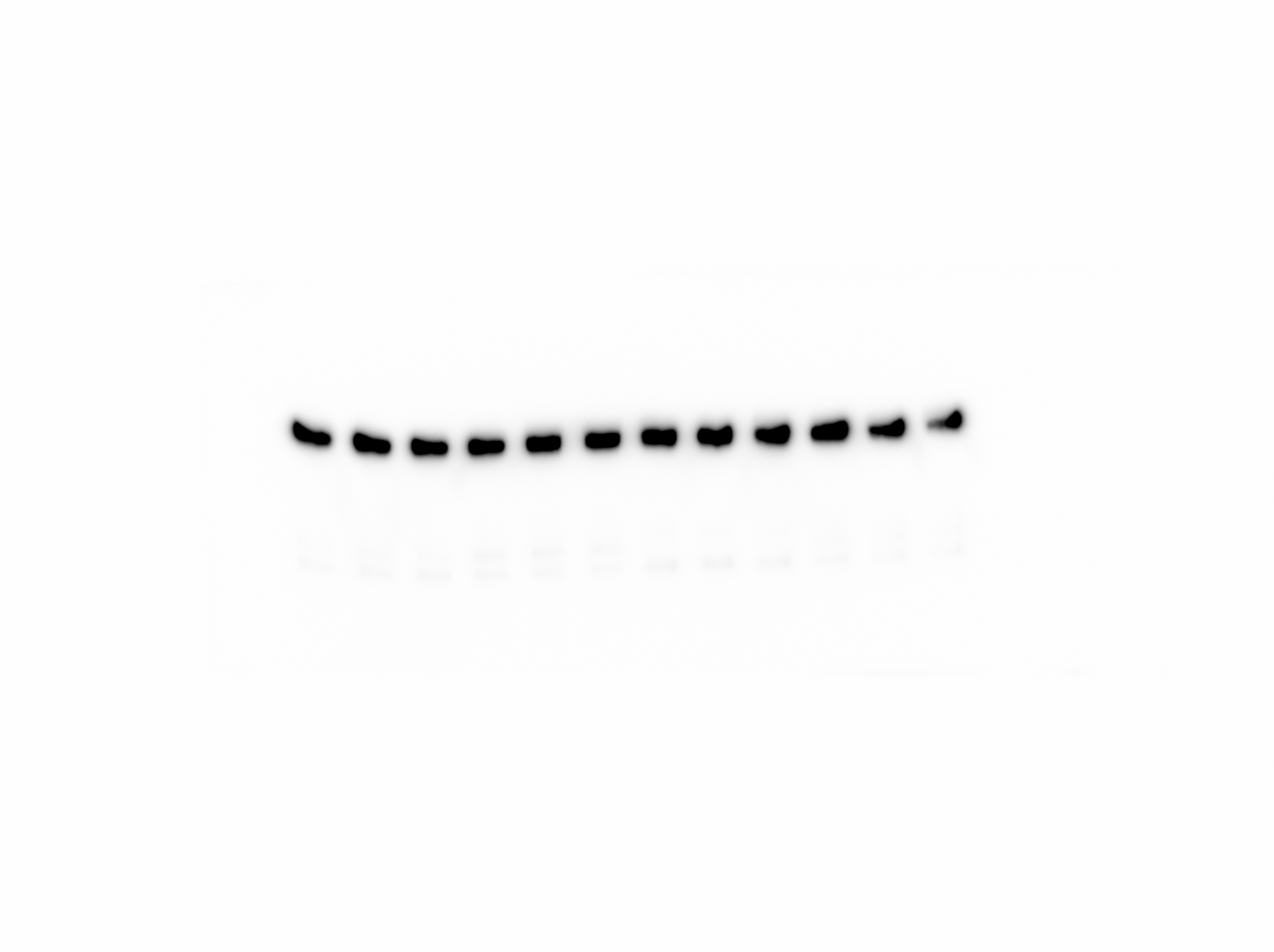

Supplement: Supplementary file 1 [file cancers-13-00862-s001.zip › WBdata_cancers/201120_TCO1_Mix_aTub/201120_TCO1_Mix_aTub_d.tif]

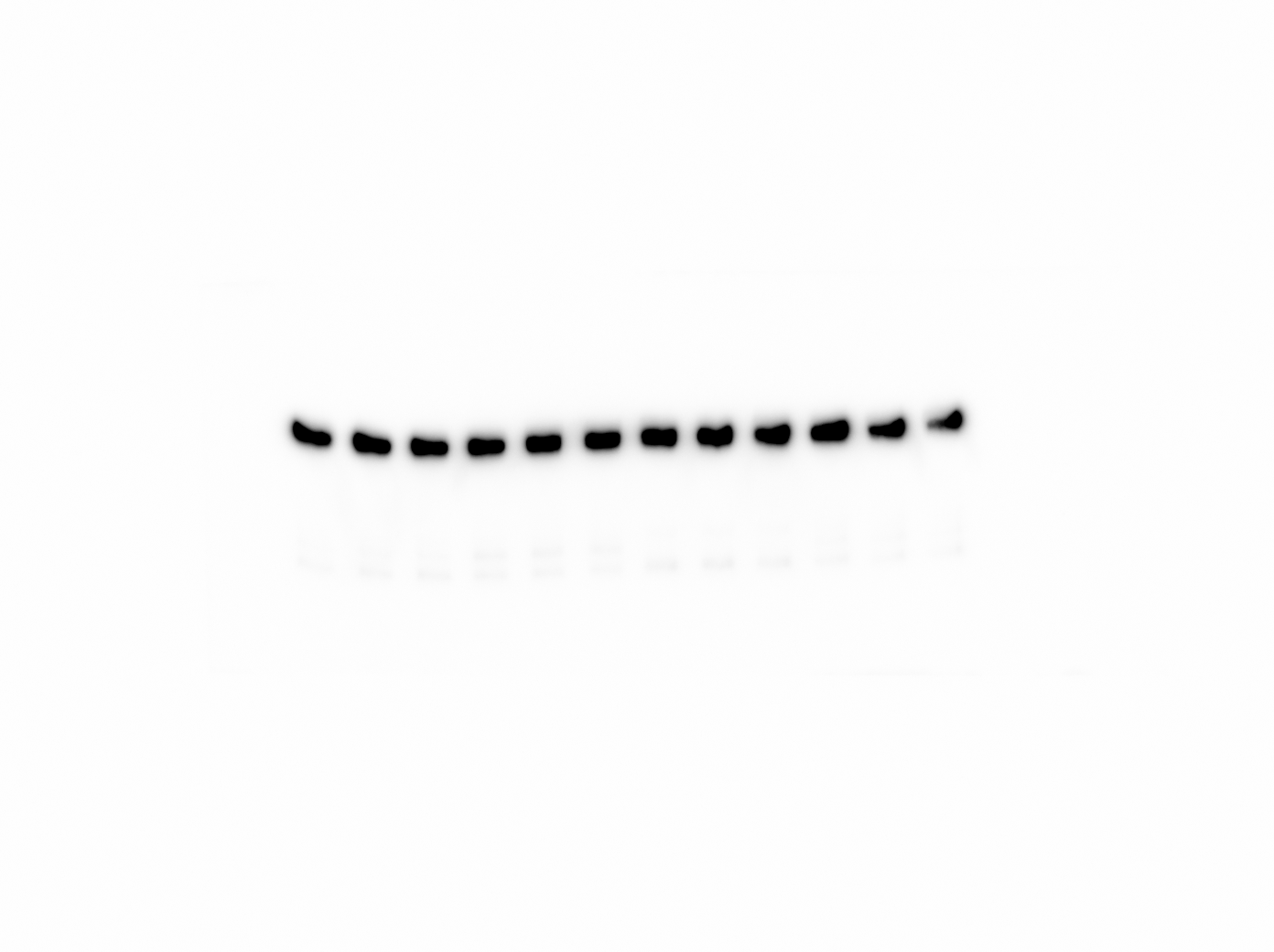

Supplement: Supplementary file 1 [file cancers-13-00862-s001.zip › WBdata_cancers/201120_TCO1_Mix_aTub/201120_TCO1_Mix_aTub_e.tif]

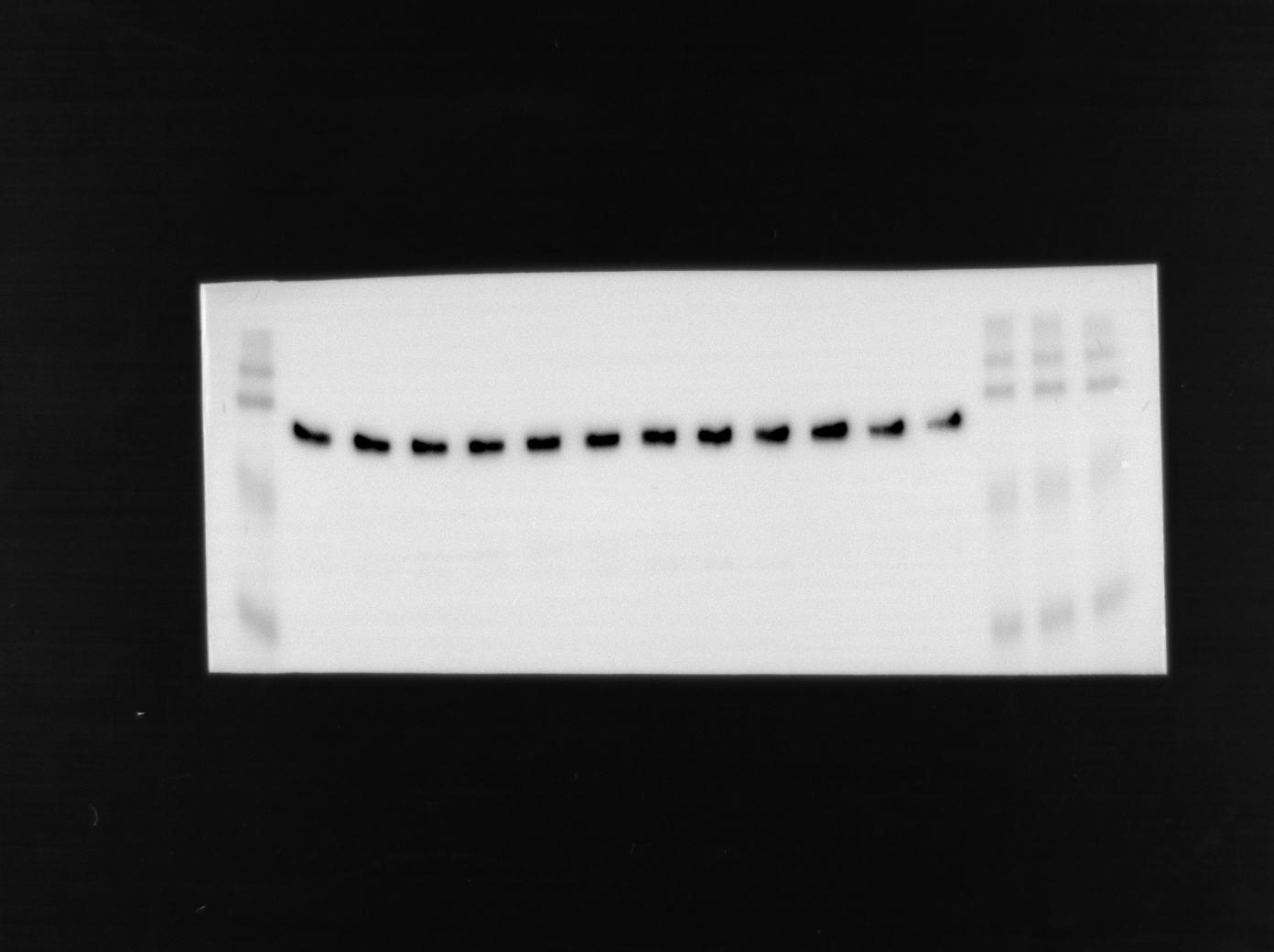

Supplement: Supplementary file 1 [file cancers-13-00862-s001.zip › WBdata_cancers/201120_TCO1_Mix_aTub/201120_TCO1_Mix_aTub_Merge.tif]

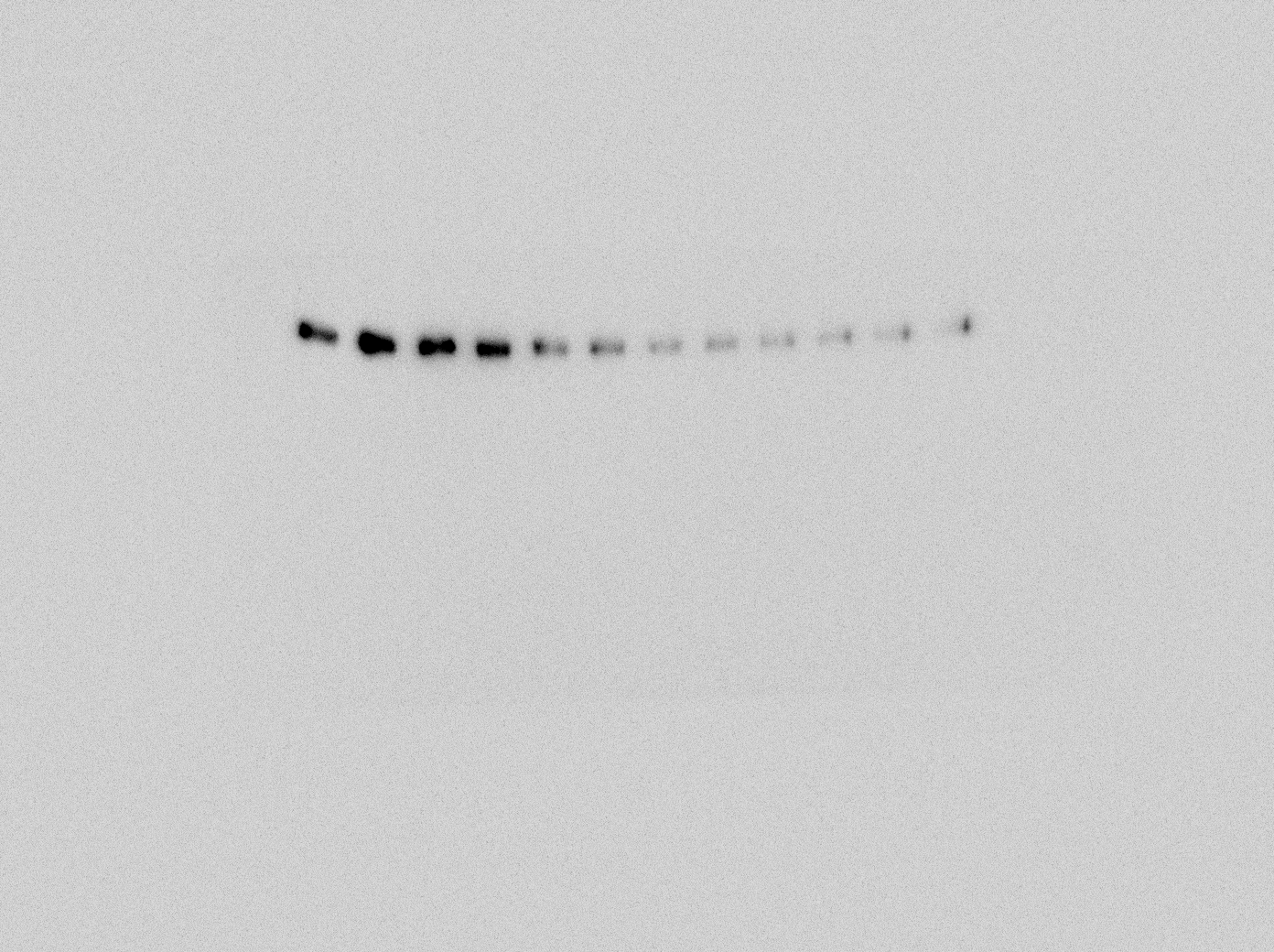

Supplement: Supplementary file 1 [file cancers-13-00862-s001.zip › WBdata_cancers/201120_TCO1_Mix_D1/201120_TCO1_Mix_D1_a.tif]

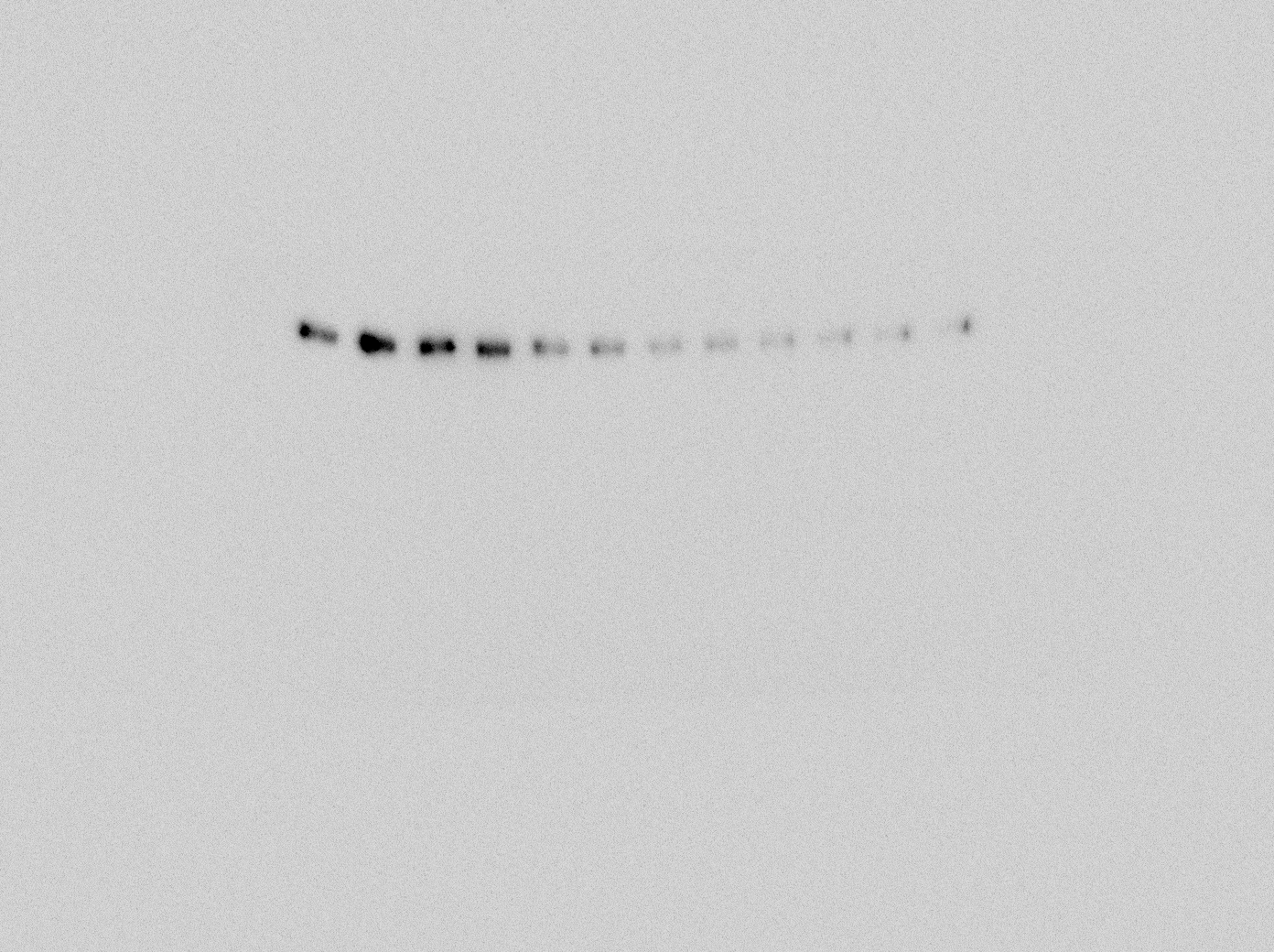

Supplement: Supplementary file 1 [file cancers-13-00862-s001.zip › WBdata_cancers/201120_TCO1_Mix_D1/201120_TCO1_Mix_D1_b.tif]

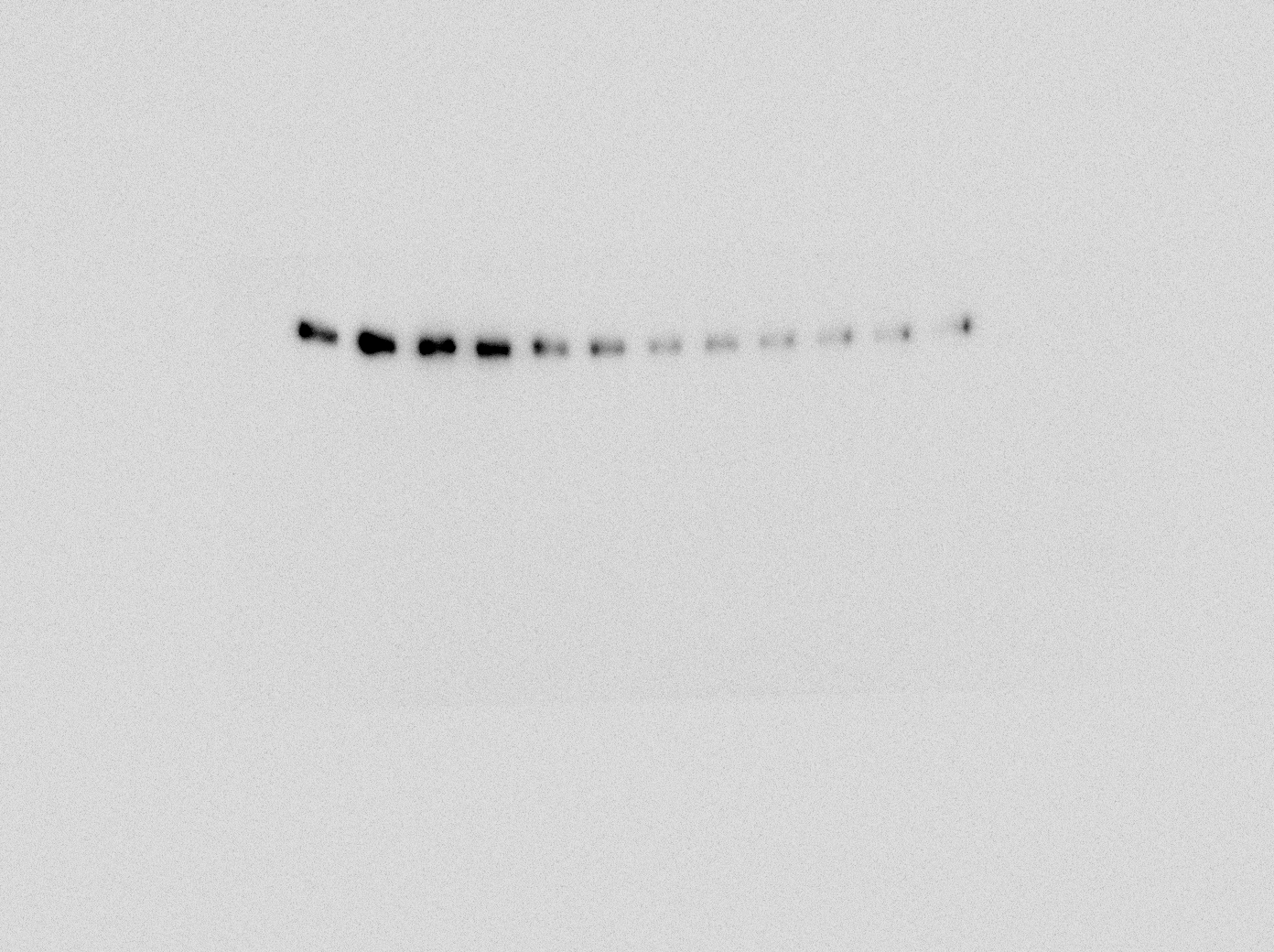

Supplement: Supplementary file 1 [file cancers-13-00862-s001.zip › WBdata_cancers/201120_TCO1_Mix_D1/201120_TCO1_Mix_D1_c.tif]

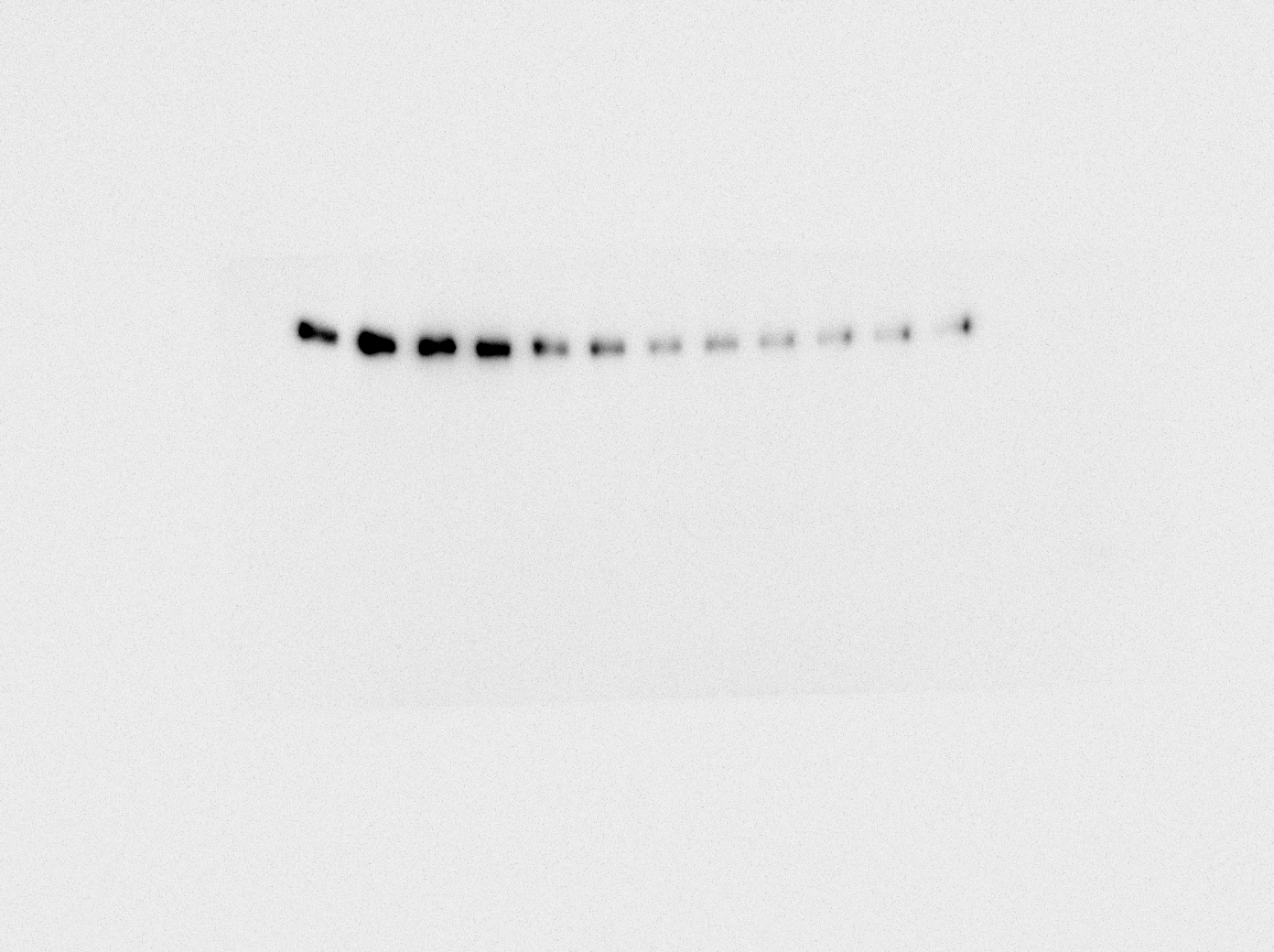

Supplement: Supplementary file 1 [file cancers-13-00862-s001.zip › WBdata_cancers/201120_TCO1_Mix_D1/201120_TCO1_Mix_D1_d.tif]

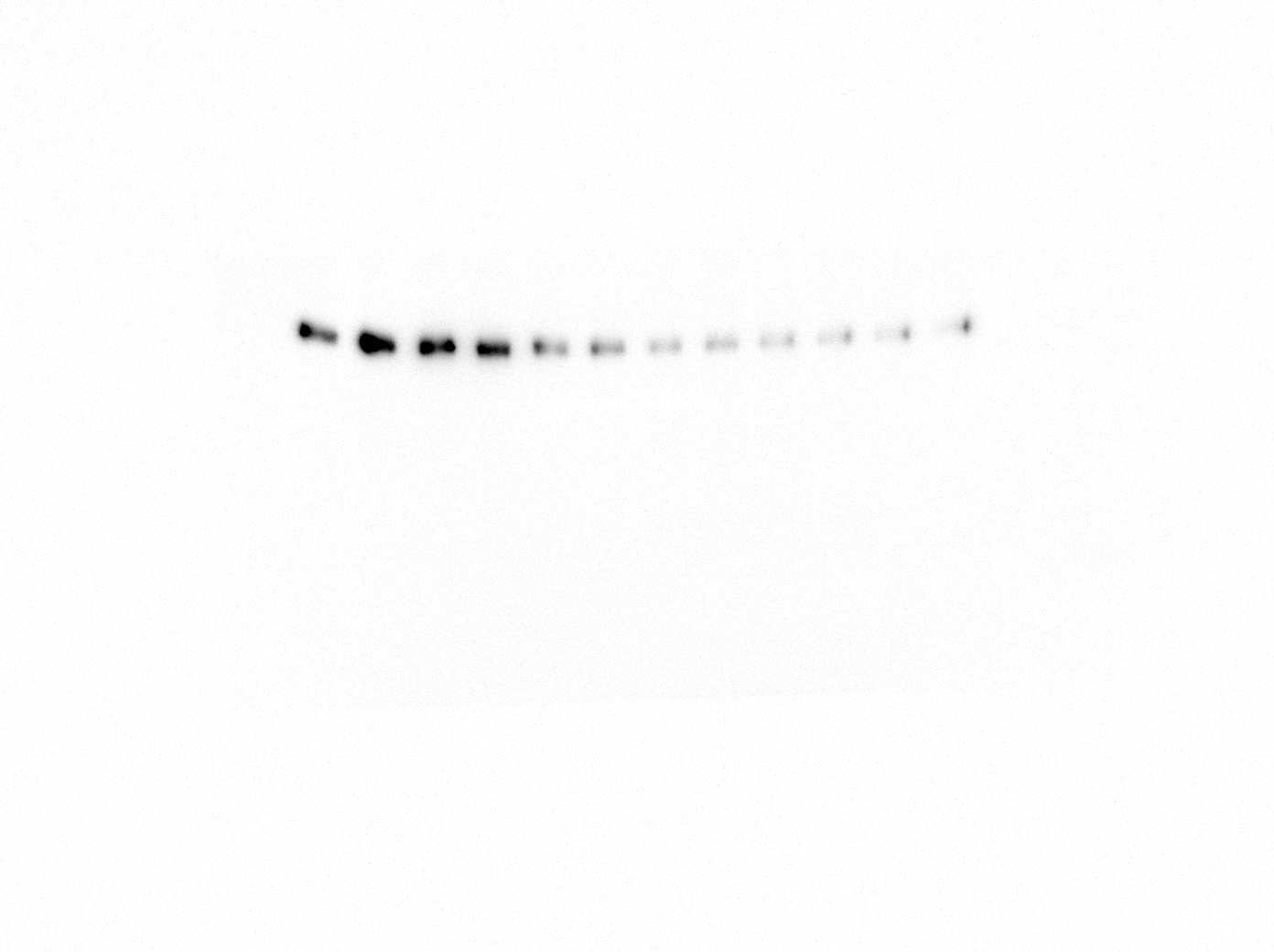

Supplement: Supplementary file 1 [file cancers-13-00862-s001.zip › WBdata_cancers/201120_TCO1_Mix_D1/201120_TCO1_Mix_D1_e.tif]

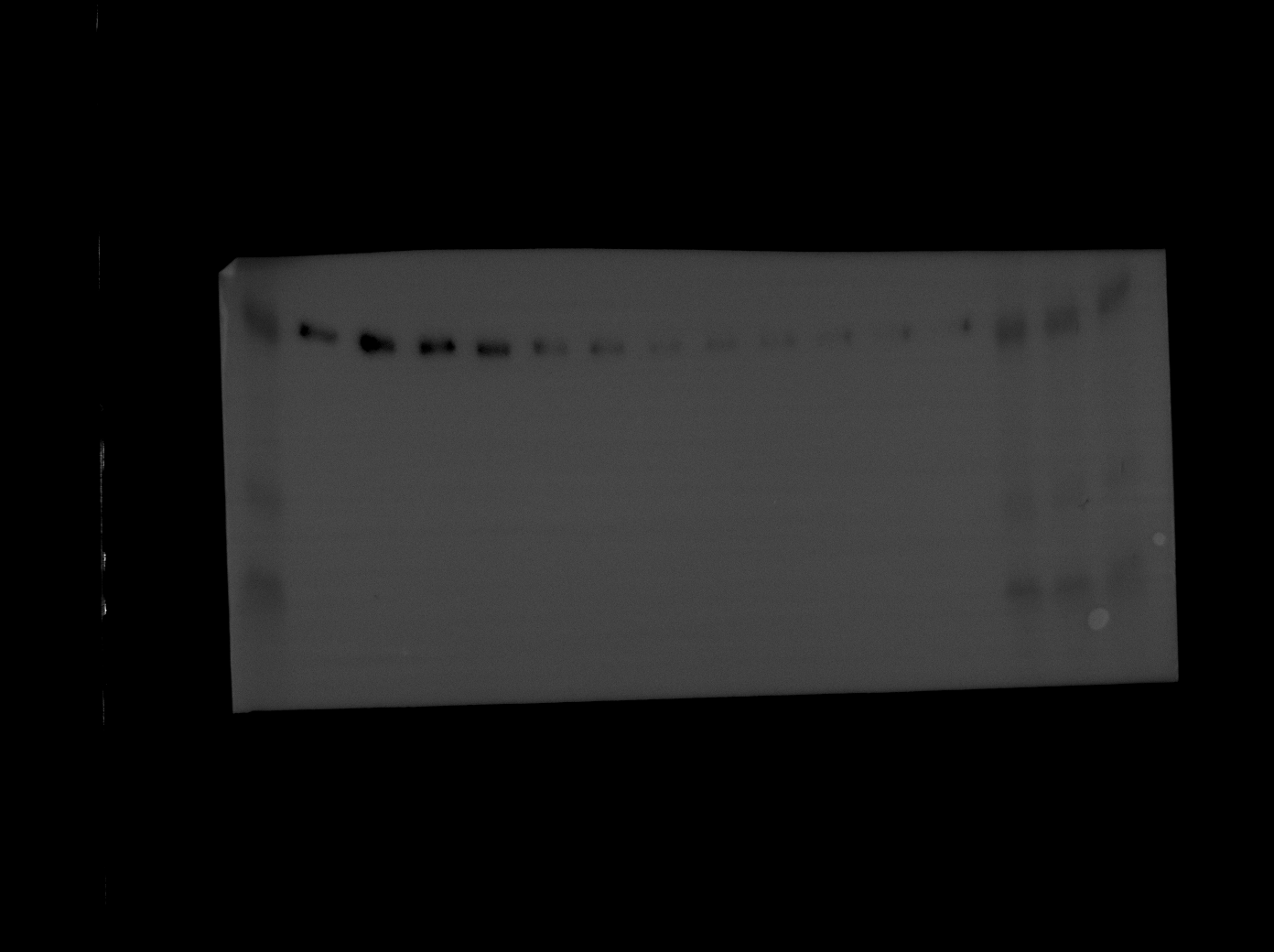

Supplement: Supplementary file 1 [file cancers-13-00862-s001.zip › WBdata_cancers/201120_TCO1_Mix_D1/201120_TCO1_Mix_D1_Merge.tif]

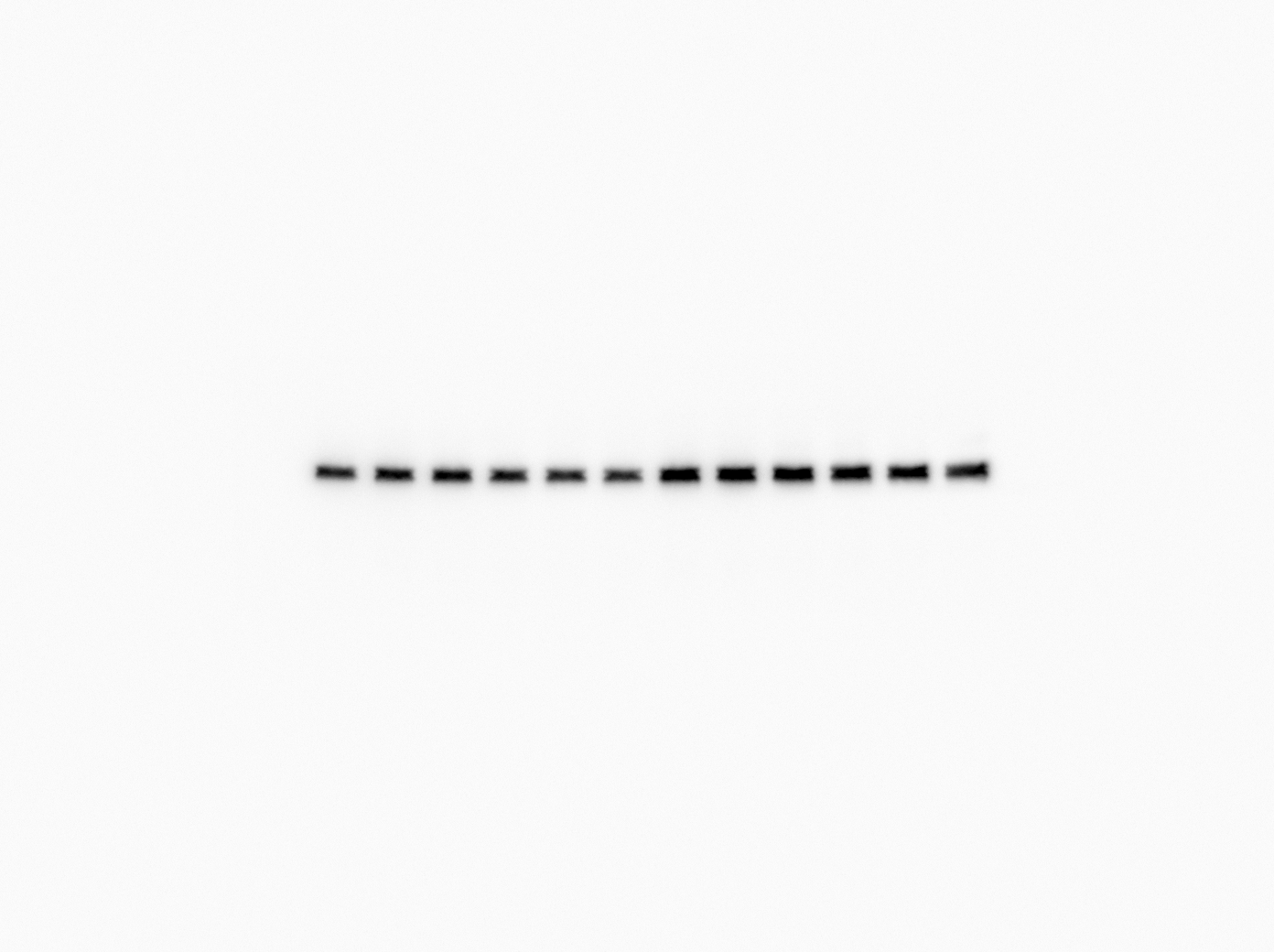

Supplement: Supplementary file 1 [file cancers-13-00862-s001.zip › WBdata_cancers/201120_TCO1_Mix_pAKT/201120_TCO1_Mix_pAKT_a.tif]

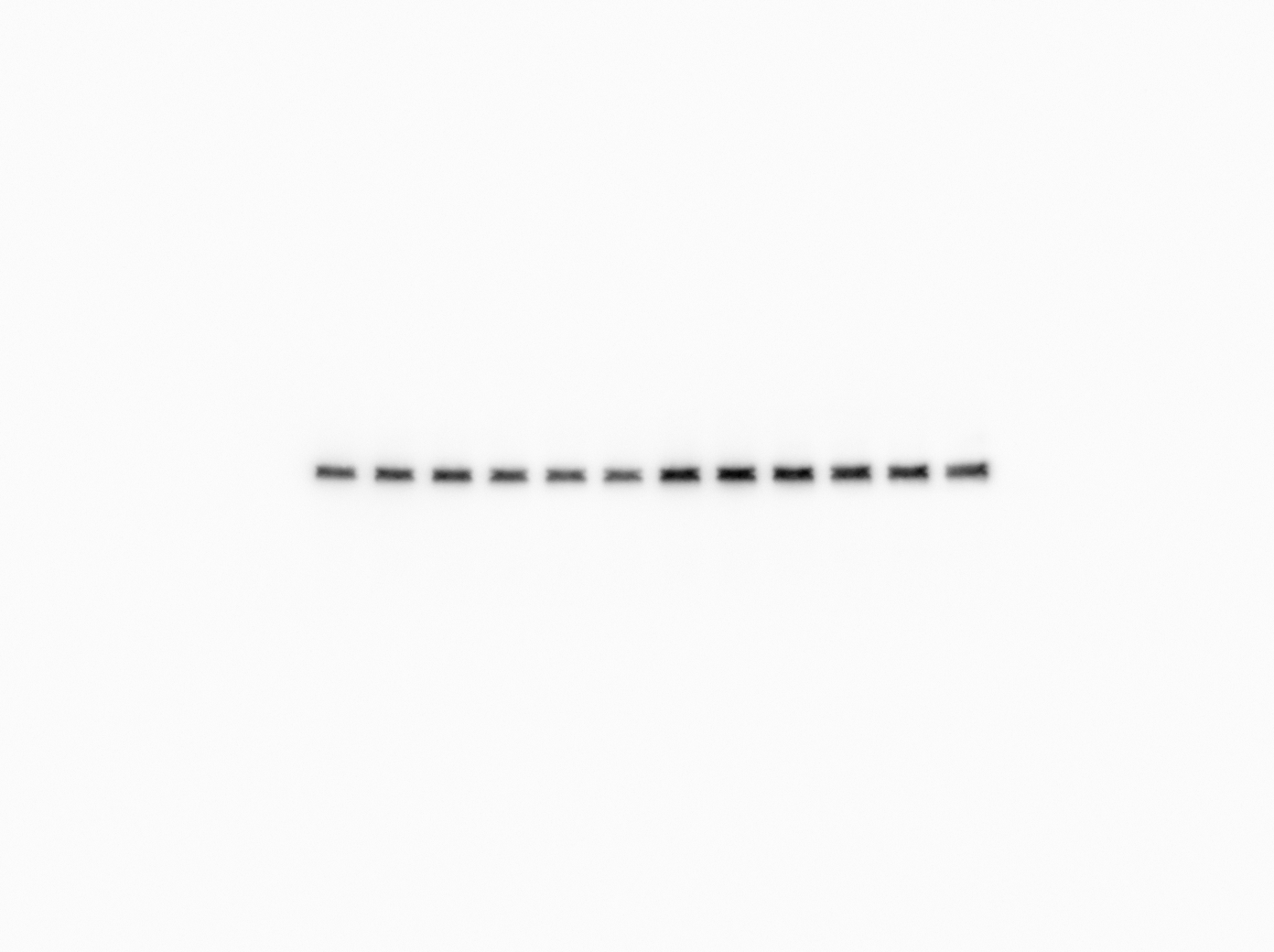

Supplement: Supplementary file 1 [file cancers-13-00862-s001.zip › WBdata_cancers/201120_TCO1_Mix_pAKT/201120_TCO1_Mix_pAKT_b.tif]

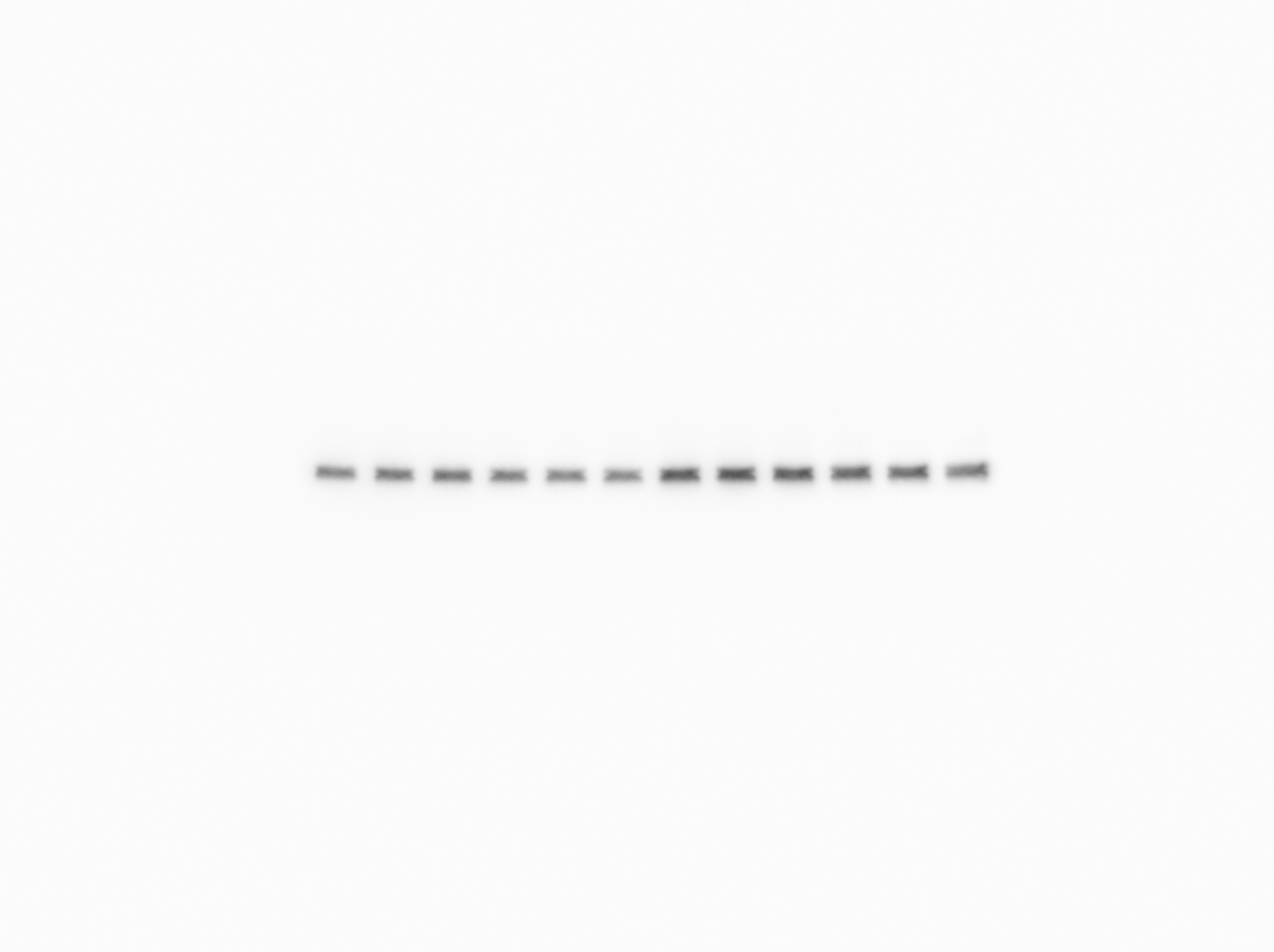

Supplement: Supplementary file 1 [file cancers-13-00862-s001.zip › WBdata_cancers/201120_TCO1_Mix_pAKT/201120_TCO1_Mix_pAKT_c.tif]

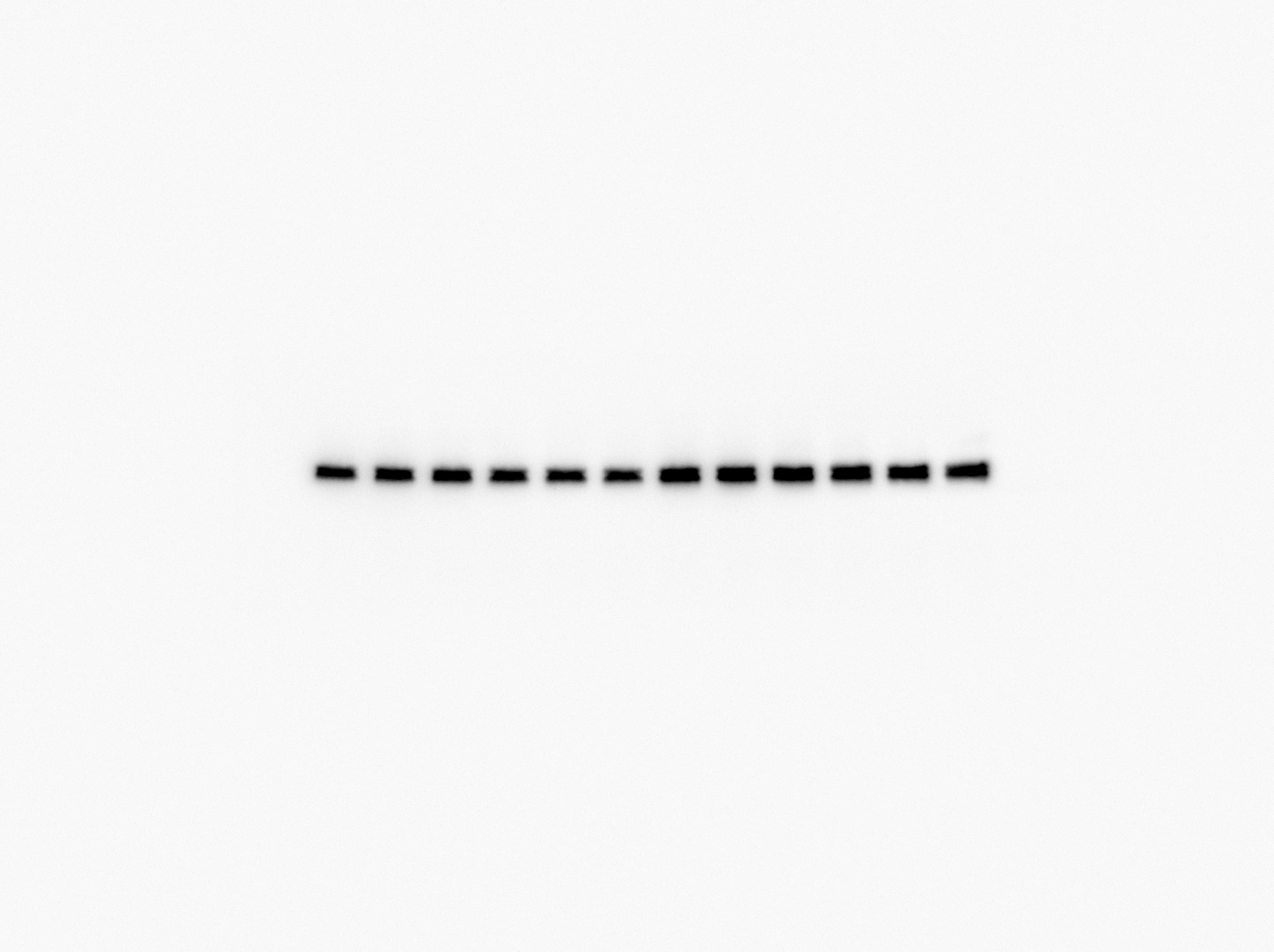

Supplement: Supplementary file 1 [file cancers-13-00862-s001.zip › WBdata_cancers/201120_TCO1_Mix_pAKT/201120_TCO1_Mix_pAKT_d.tif]

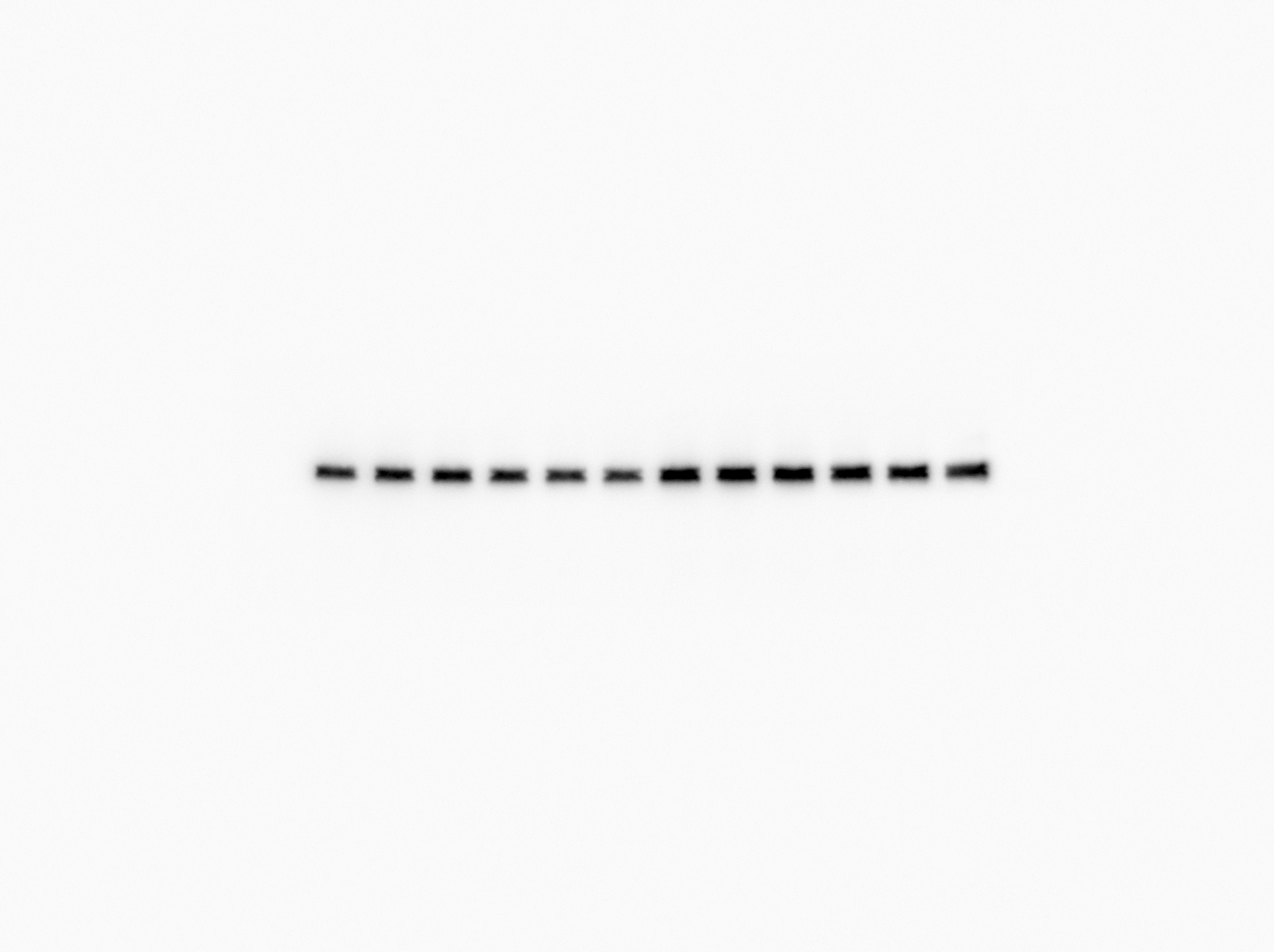

Supplement: Supplementary file 1 [file cancers-13-00862-s001.zip › WBdata_cancers/201120_TCO1_Mix_pAKT/201120_TCO1_Mix_pAKT_e.tif]

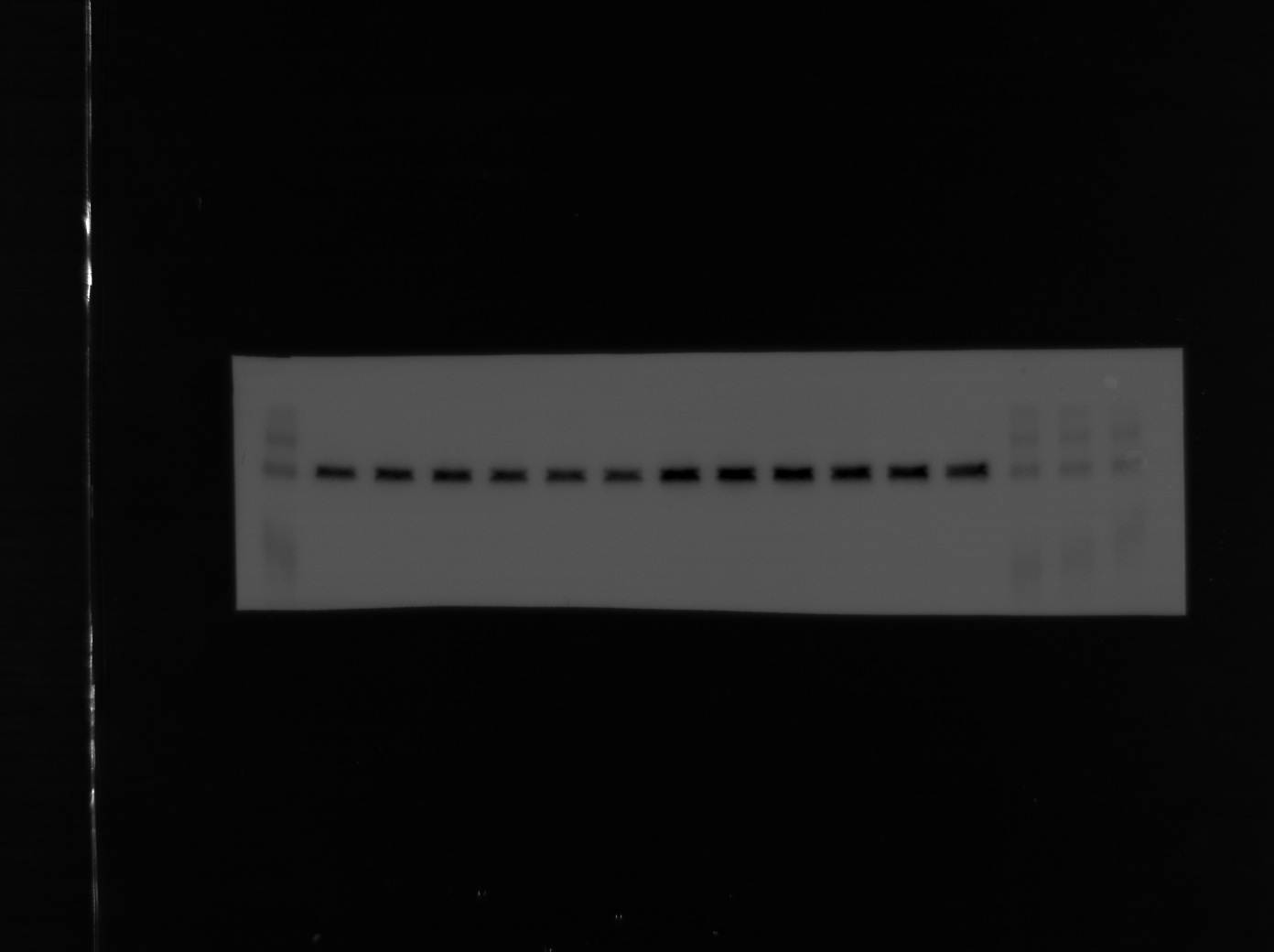

Supplement: Supplementary file 1 [file cancers-13-00862-s001.zip › WBdata_cancers/201120_TCO1_Mix_pAKT/201120_TCO1_Mix_pAKT_Merge.tif]

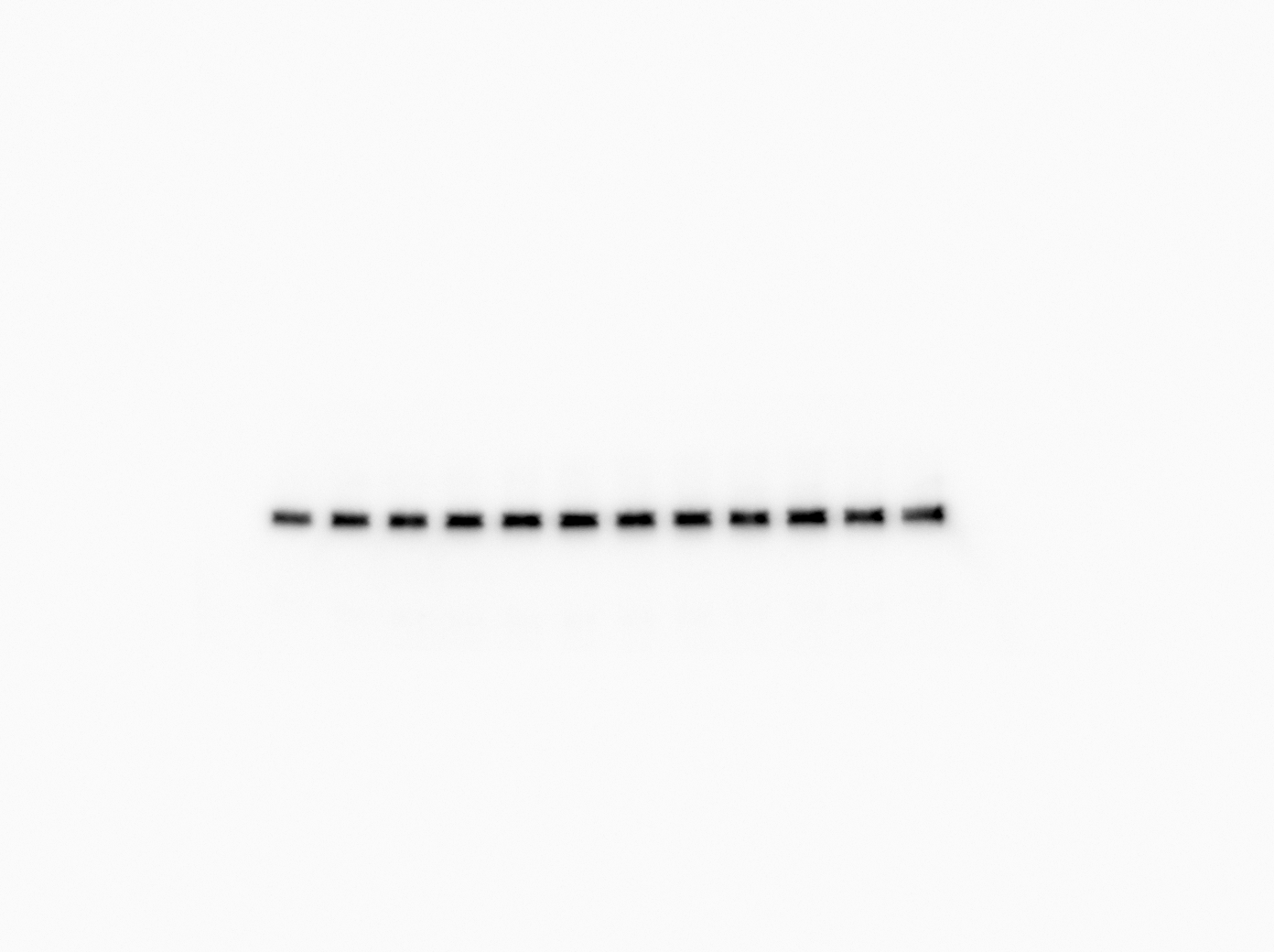

Supplement: Supplementary file 1 [file cancers-13-00862-s001.zip › WBdata_cancers/201120_TCO1_Mix_tAKT/201120_TCO1_Mix_tAKT_a.tif]

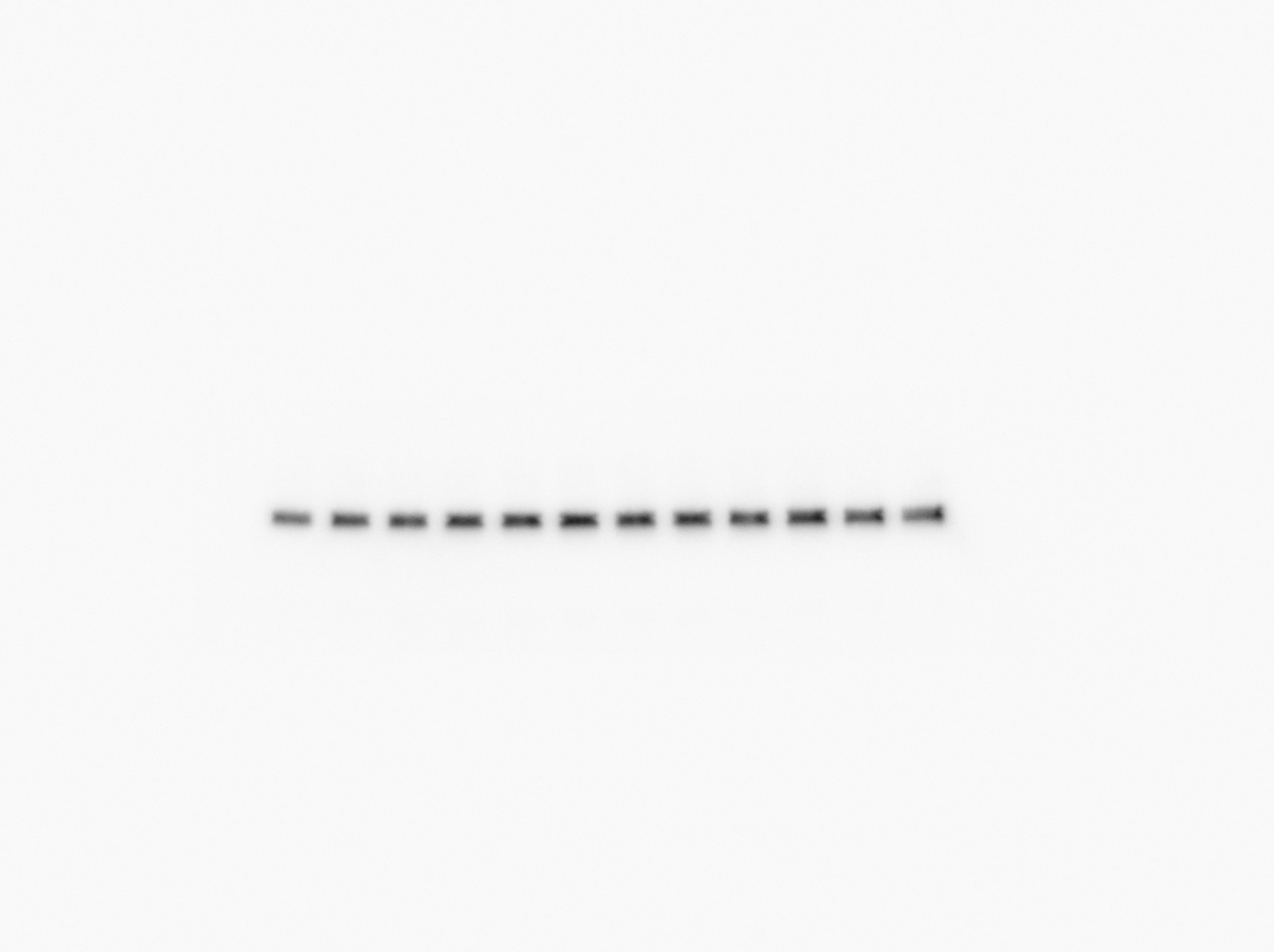

Supplement: Supplementary file 1 [file cancers-13-00862-s001.zip › WBdata_cancers/201120_TCO1_Mix_tAKT/201120_TCO1_Mix_tAKT_b.tif]

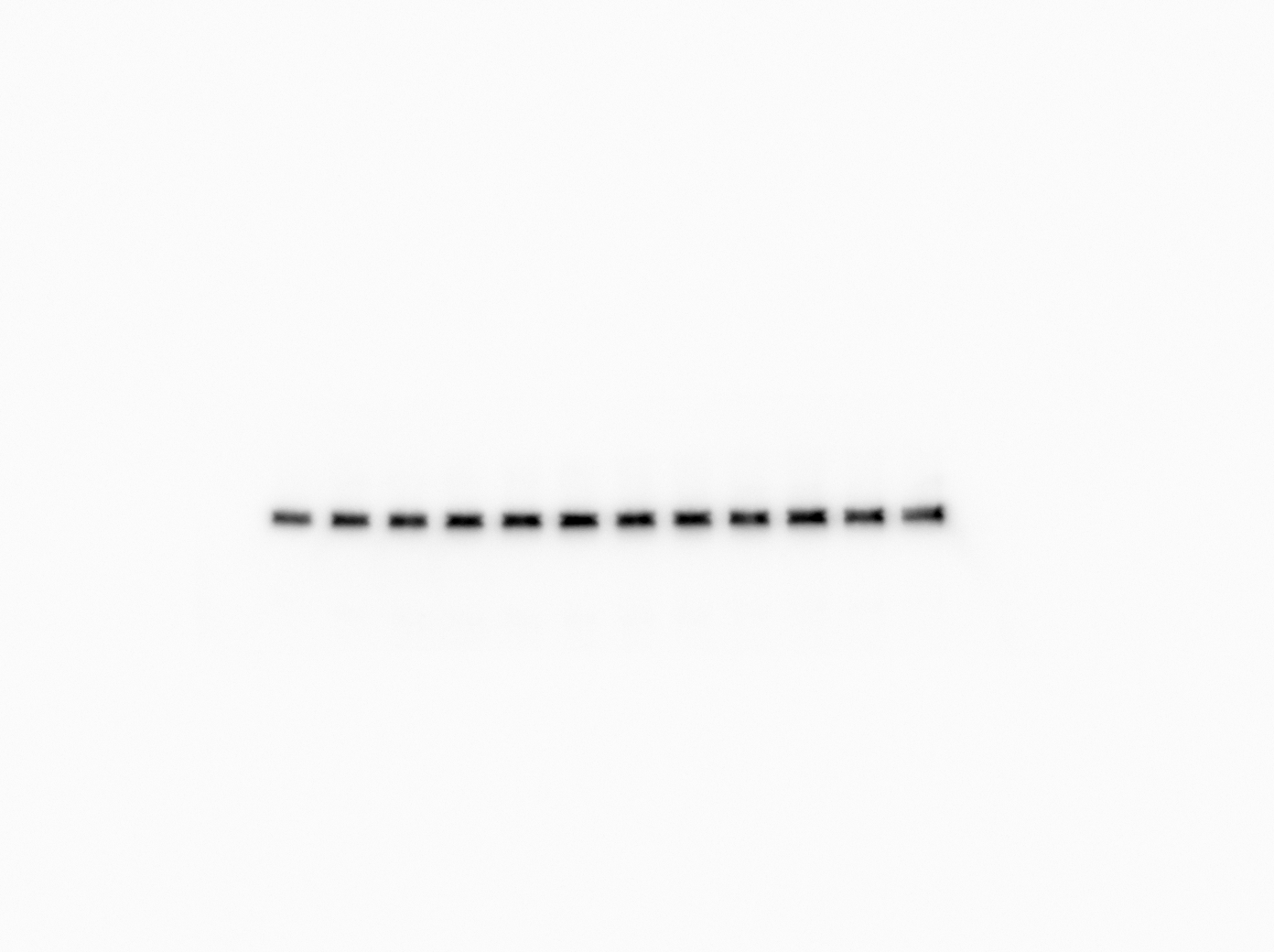

Supplement: Supplementary file 1 [file cancers-13-00862-s001.zip › WBdata_cancers/201120_TCO1_Mix_tAKT/201120_TCO1_Mix_tAKT_c.tif]

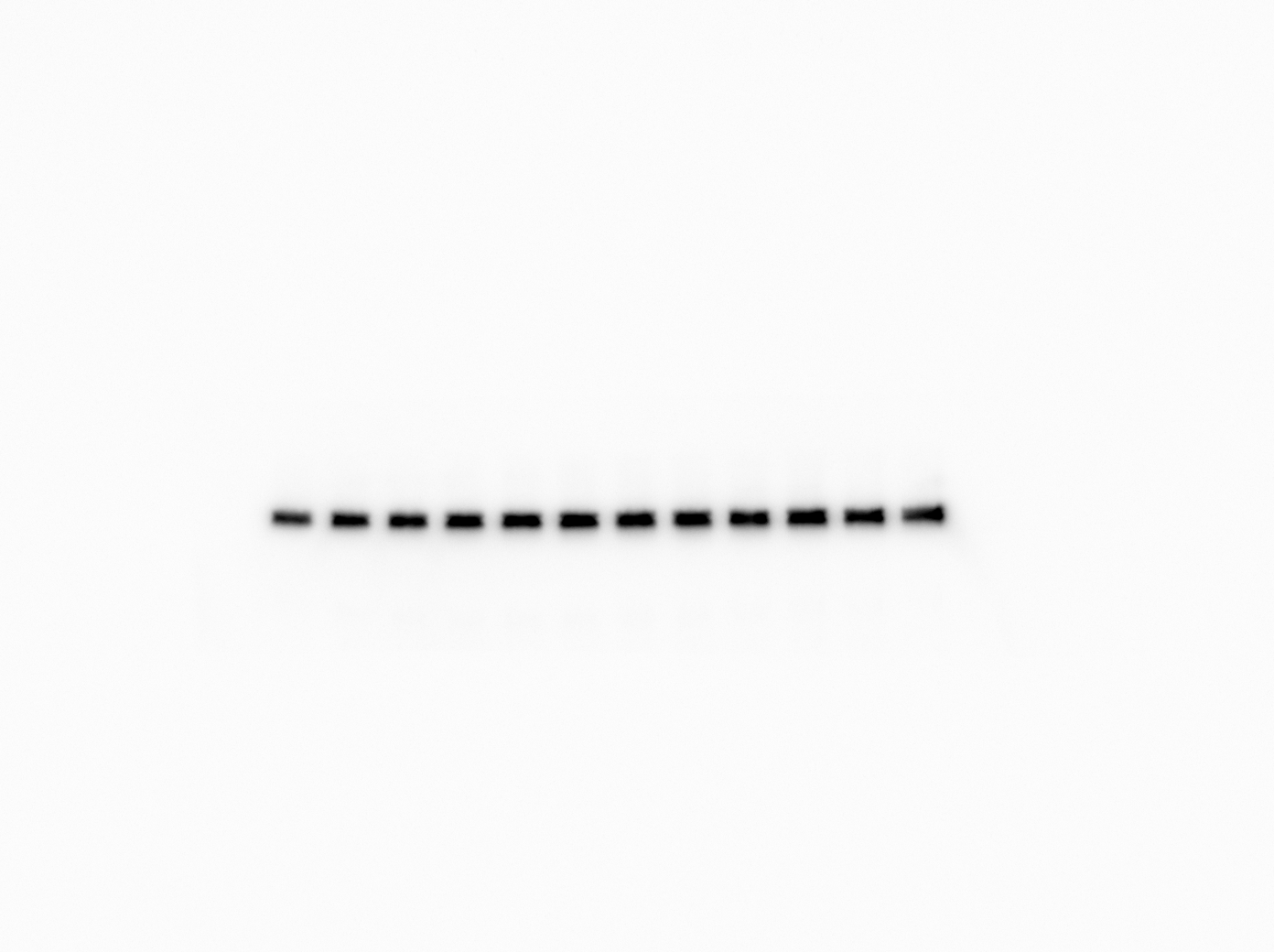

Supplement: Supplementary file 1 [file cancers-13-00862-s001.zip › WBdata_cancers/201120_TCO1_Mix_tAKT/201120_TCO1_Mix_tAKT_d.tif]

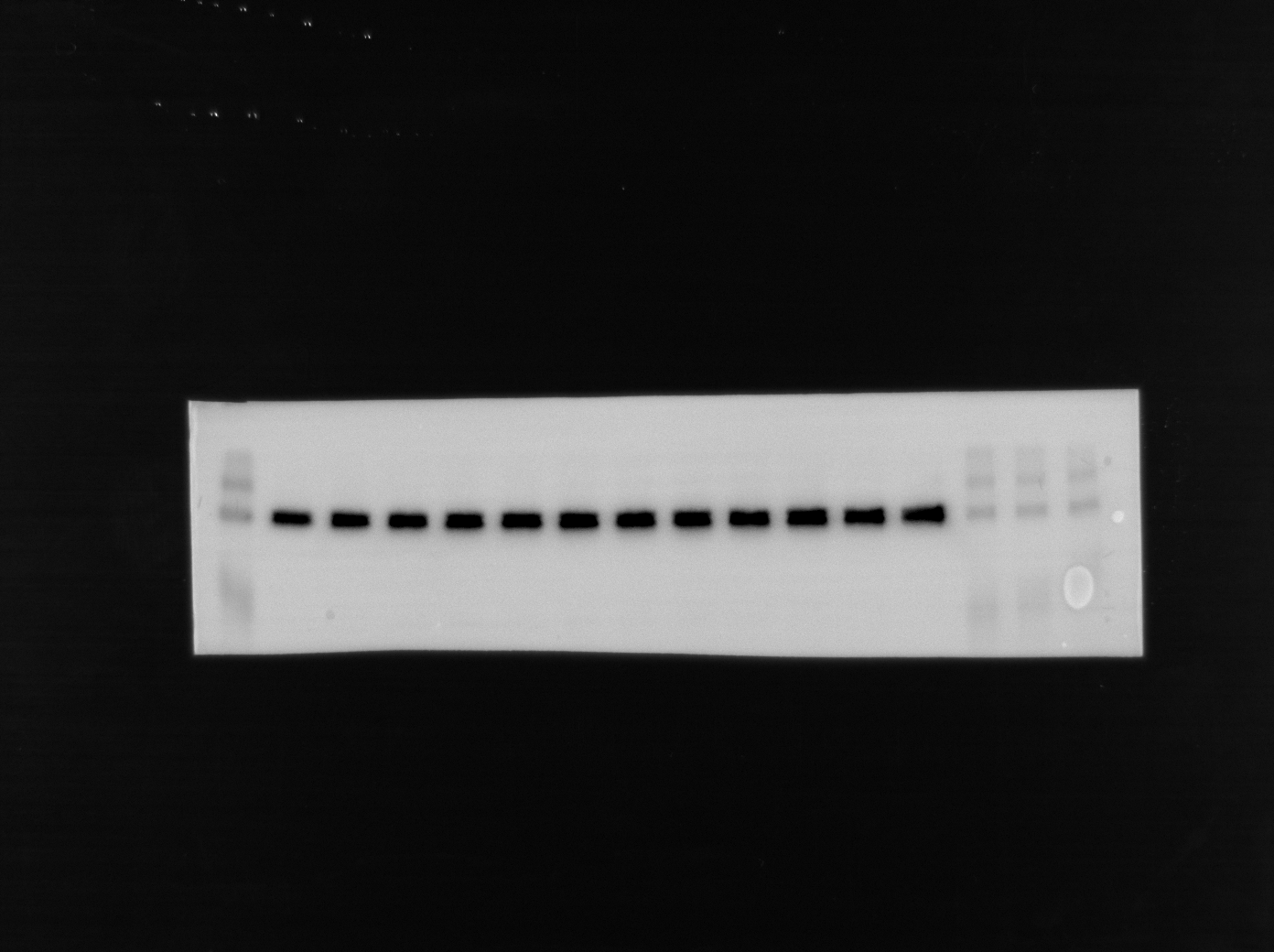

Supplement: Supplementary file 1 [file cancers-13-00862-s001.zip › WBdata_cancers/201120_TCO1_Mix_tAKT/201120_TCO1_Mix_tAKT_Merge.tif]

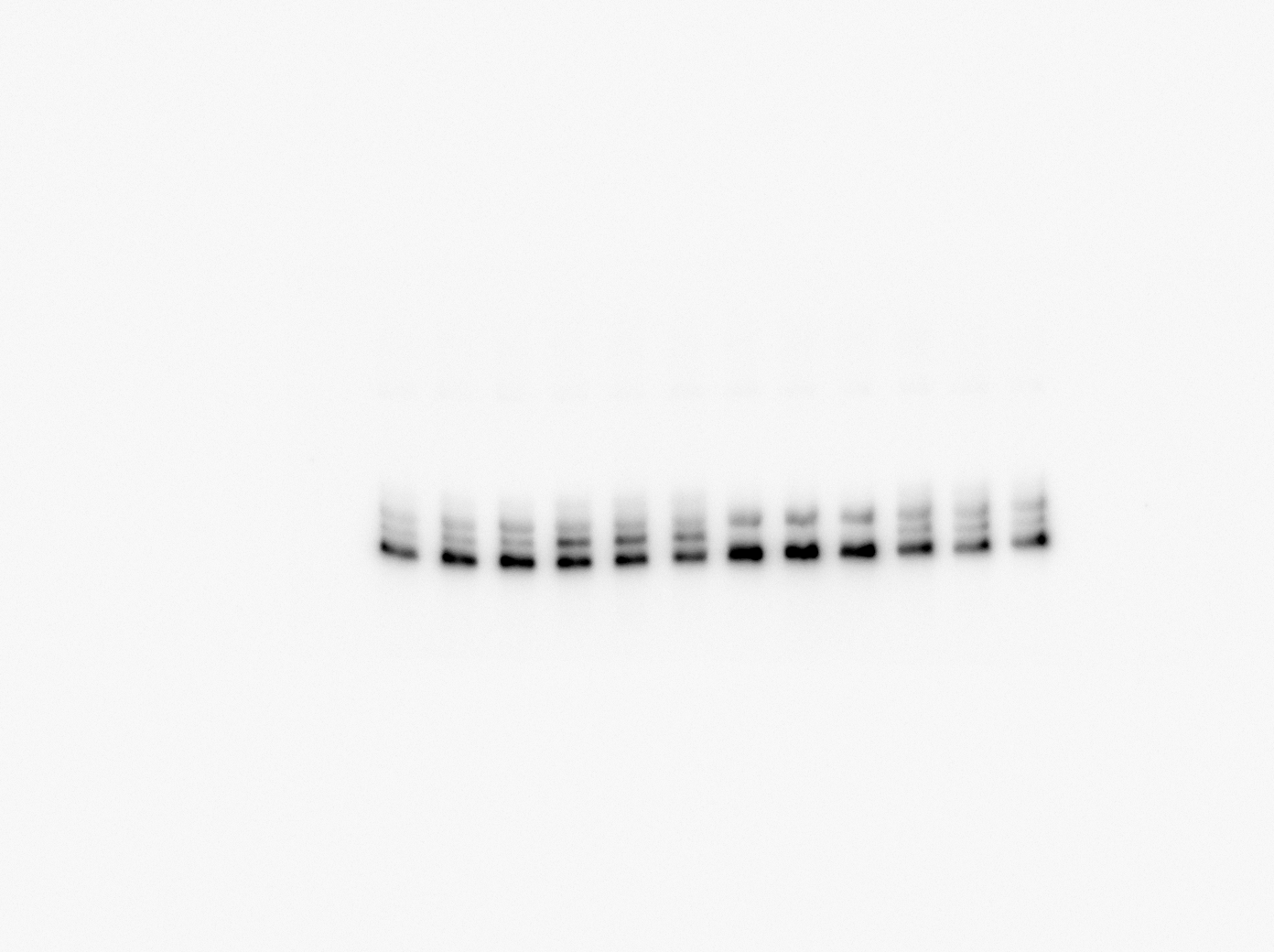

Supplement: Supplementary file 1 [file cancers-13-00862-s001.zip › WBdata_cancers/201120_TCO1_Mix_tERK/201120_TCO1_Mix_tERKa.tif]

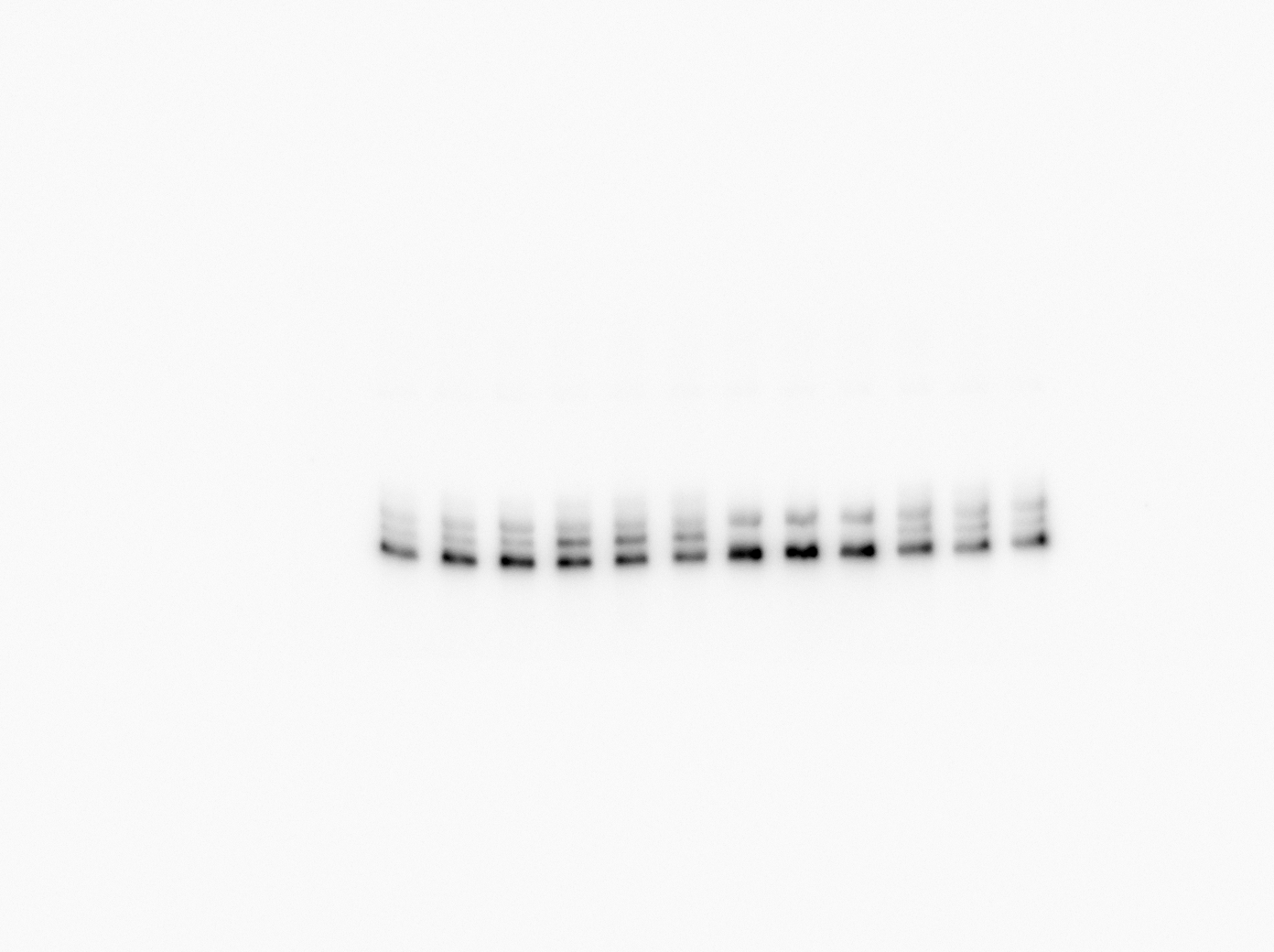

Supplement: Supplementary file 1 [file cancers-13-00862-s001.zip › WBdata_cancers/201120_TCO1_Mix_tERK/201120_TCO1_Mix_tERKb.tif]

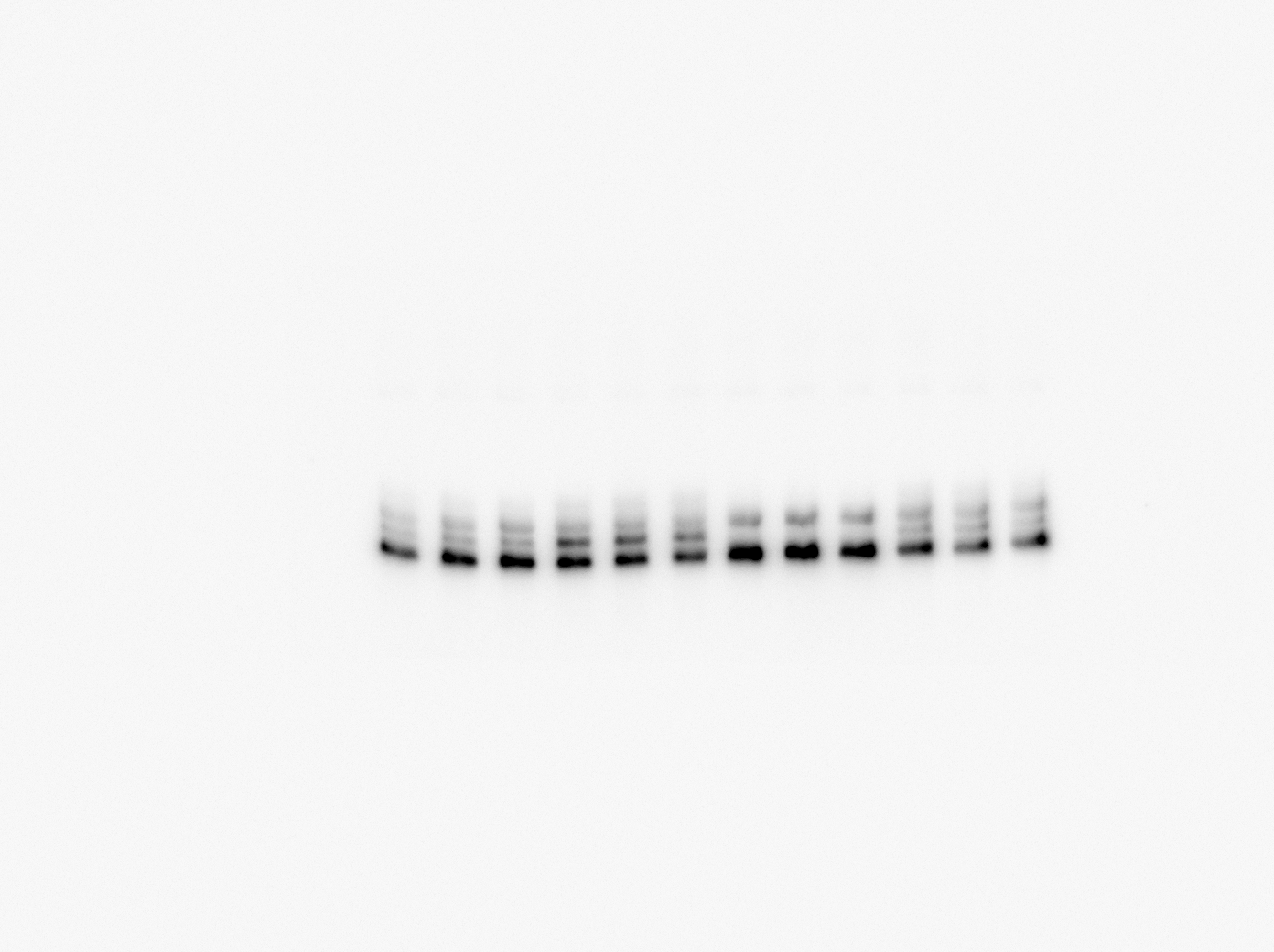

Supplement: Supplementary file 1 [file cancers-13-00862-s001.zip › WBdata_cancers/201120_TCO1_Mix_tERK/201120_TCO1_Mix_tERKc.tif]

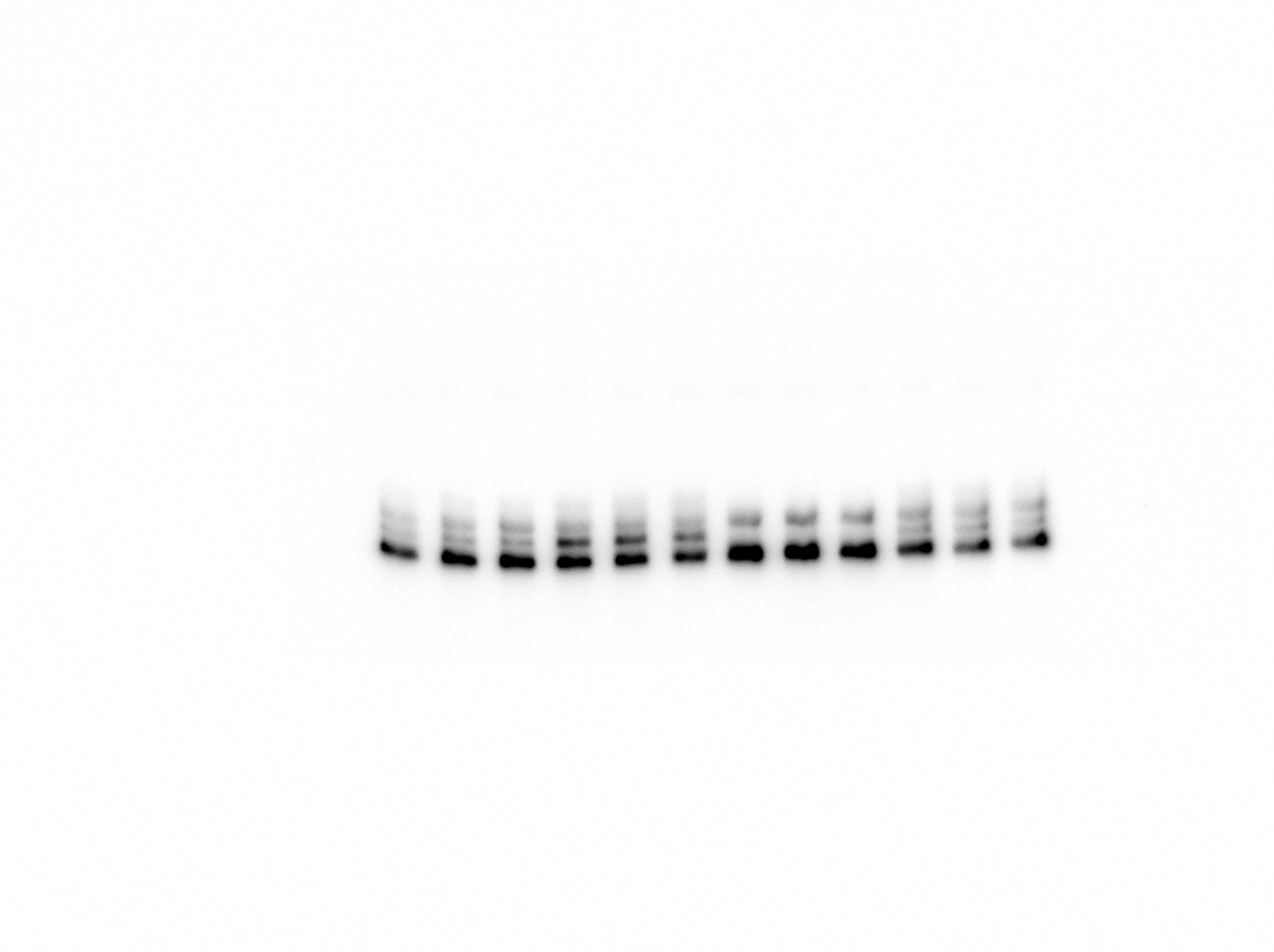

Supplement: Supplementary file 1 [file cancers-13-00862-s001.zip › WBdata_cancers/201120_TCO1_Mix_tERK/201120_TCO1_Mix_tERKd.tif]

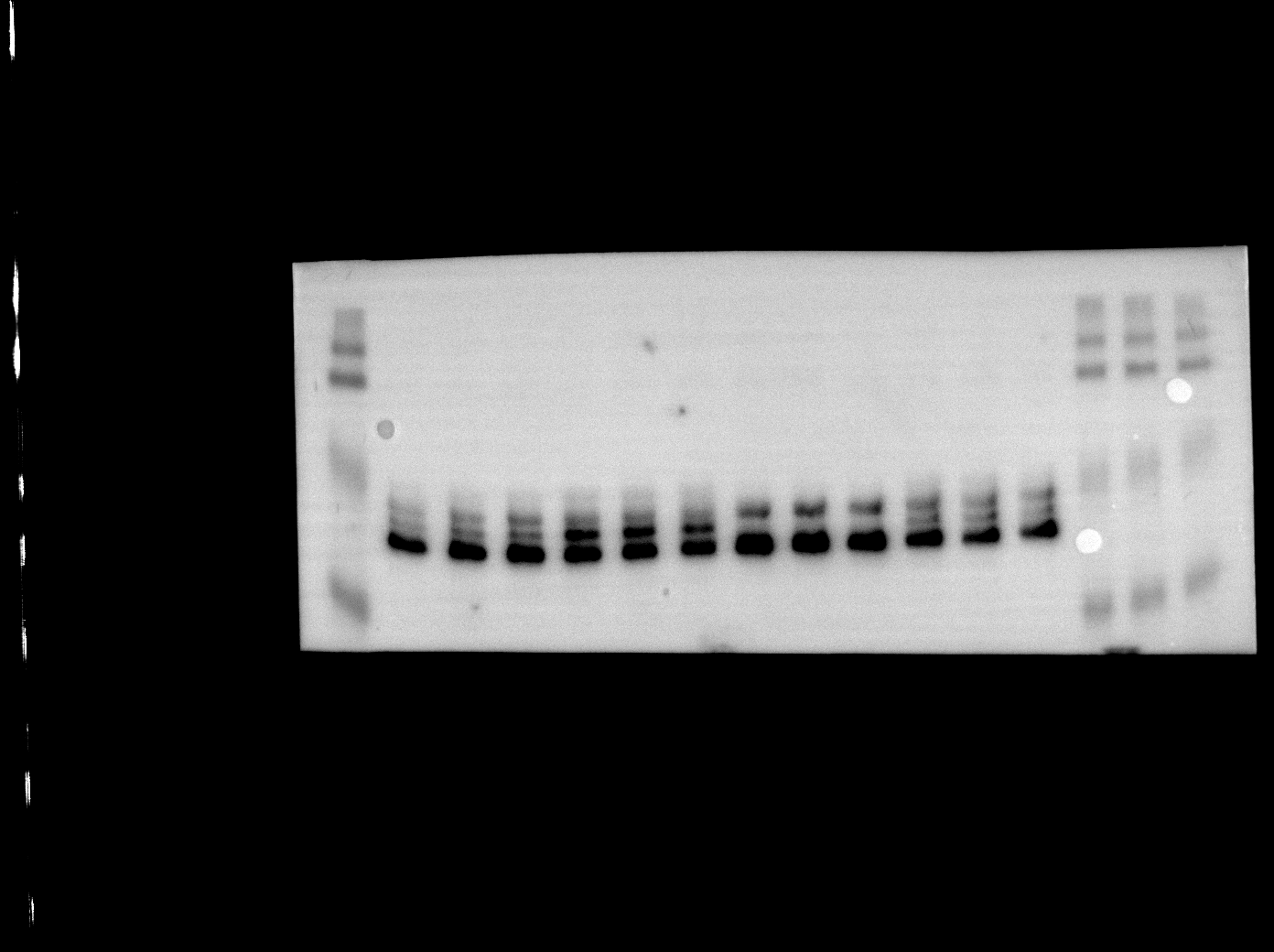

Supplement: Supplementary file 1 [file cancers-13-00862-s001.zip › WBdata_cancers/201120_TCO1_Mix_tERK/201120_TCO1_Mix_tERK_Merge.tif]

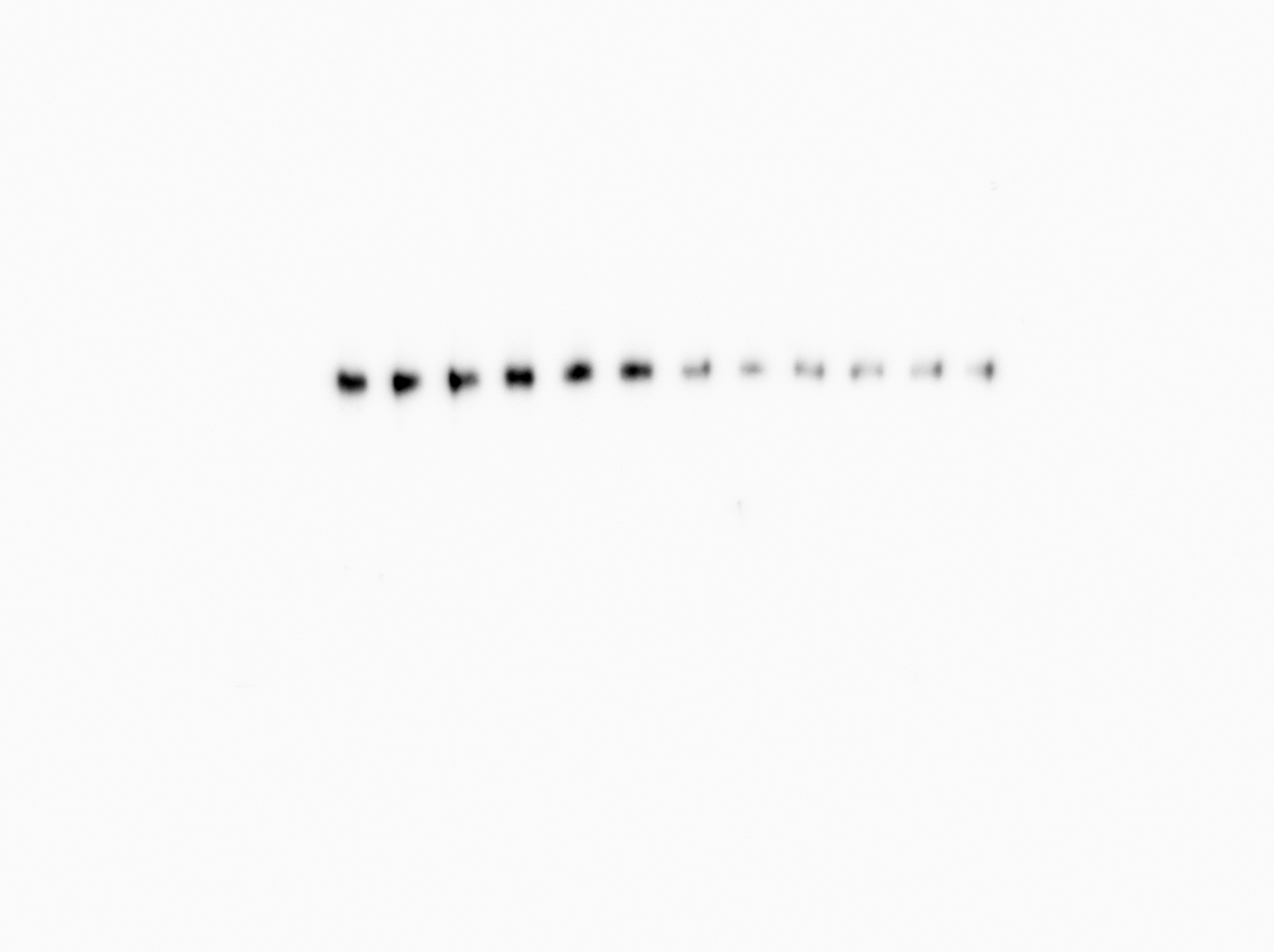

Supplement: Supplementary file 1 [file cancers-13-00862-s001.zip › WBdata_cancers/20200228_8505_E7080U0126_D1/20200228_8505_E7080U0126_D1_a.tif]

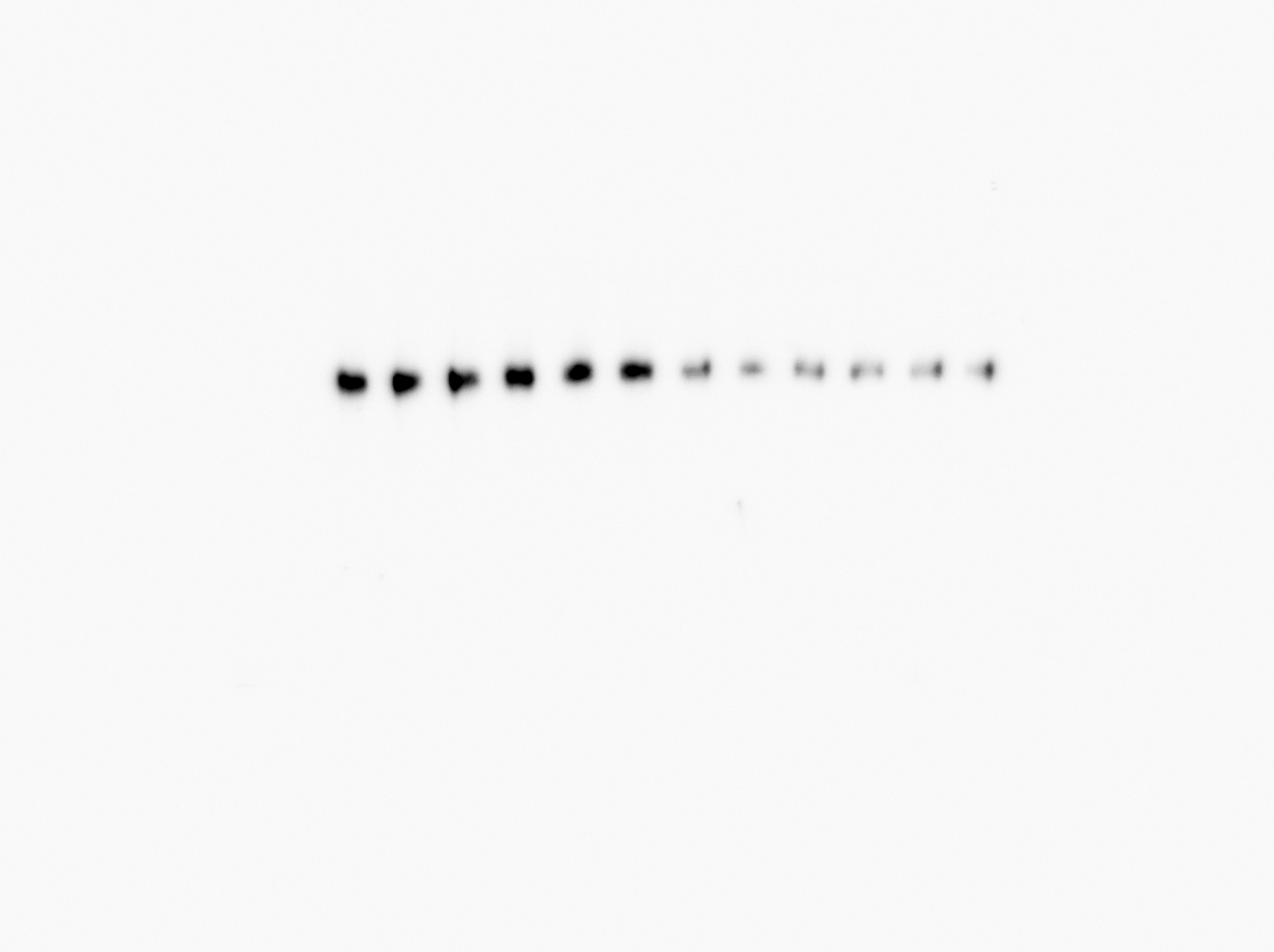

Supplement: Supplementary file 1 [file cancers-13-00862-s001.zip › WBdata_cancers/20200228_8505_E7080U0126_D1/20200228_8505_E7080U0126_D1_b.tif]

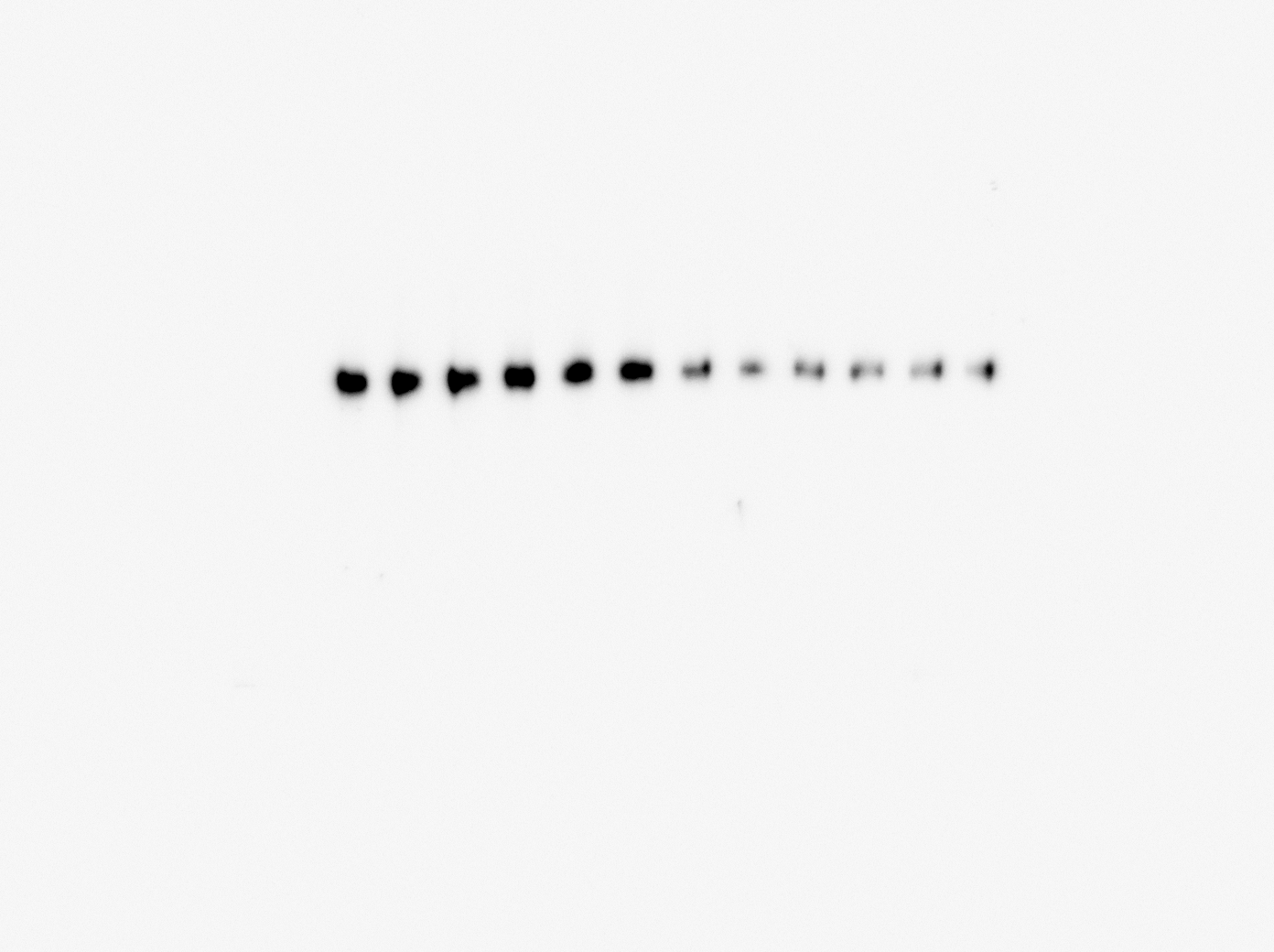

Supplement: Supplementary file 1 [file cancers-13-00862-s001.zip › WBdata_cancers/20200228_8505_E7080U0126_D1/20200228_8505_E7080U0126_D1_c.tif]

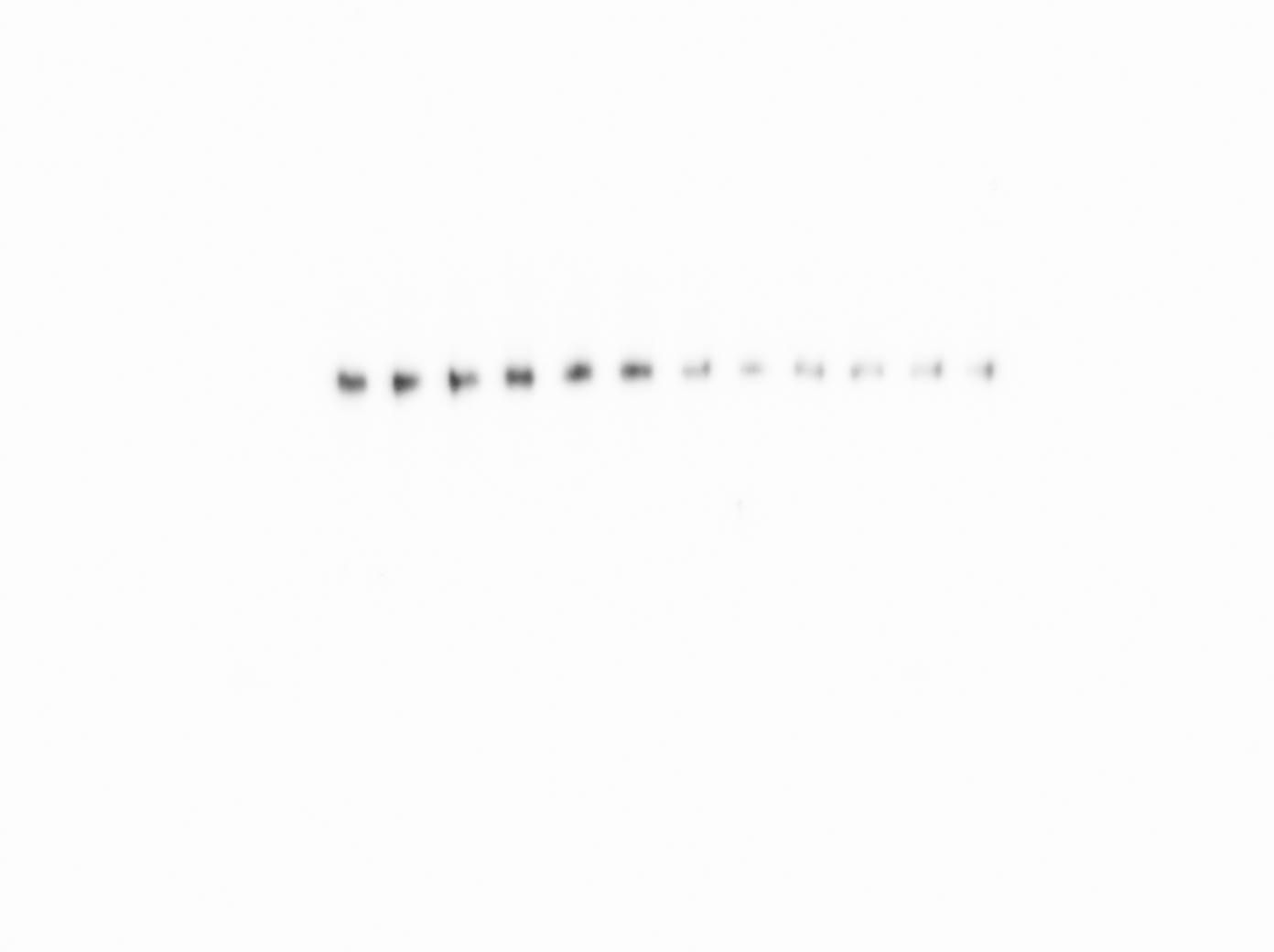

Supplement: Supplementary file 1 [file cancers-13-00862-s001.zip › WBdata_cancers/20200228_8505_E7080U0126_D1/20200228_8505_E7080U0126_D1_d.tif]

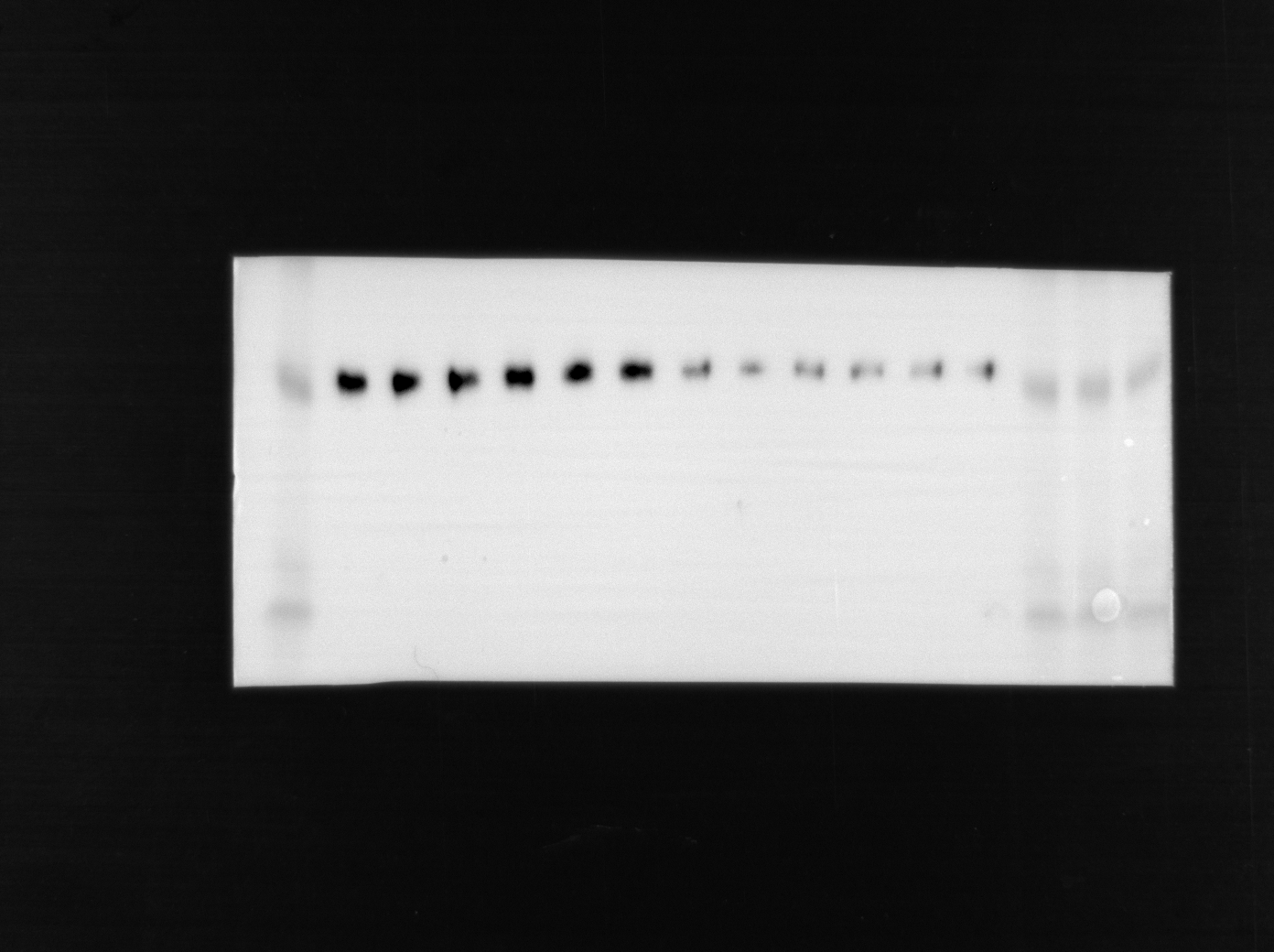

Supplement: Supplementary file 1 [file cancers-13-00862-s001.zip › WBdata_cancers/20200228_8505_E7080U0126_D1/20200228_8505_E7080U0126_D1_Merge.tif]

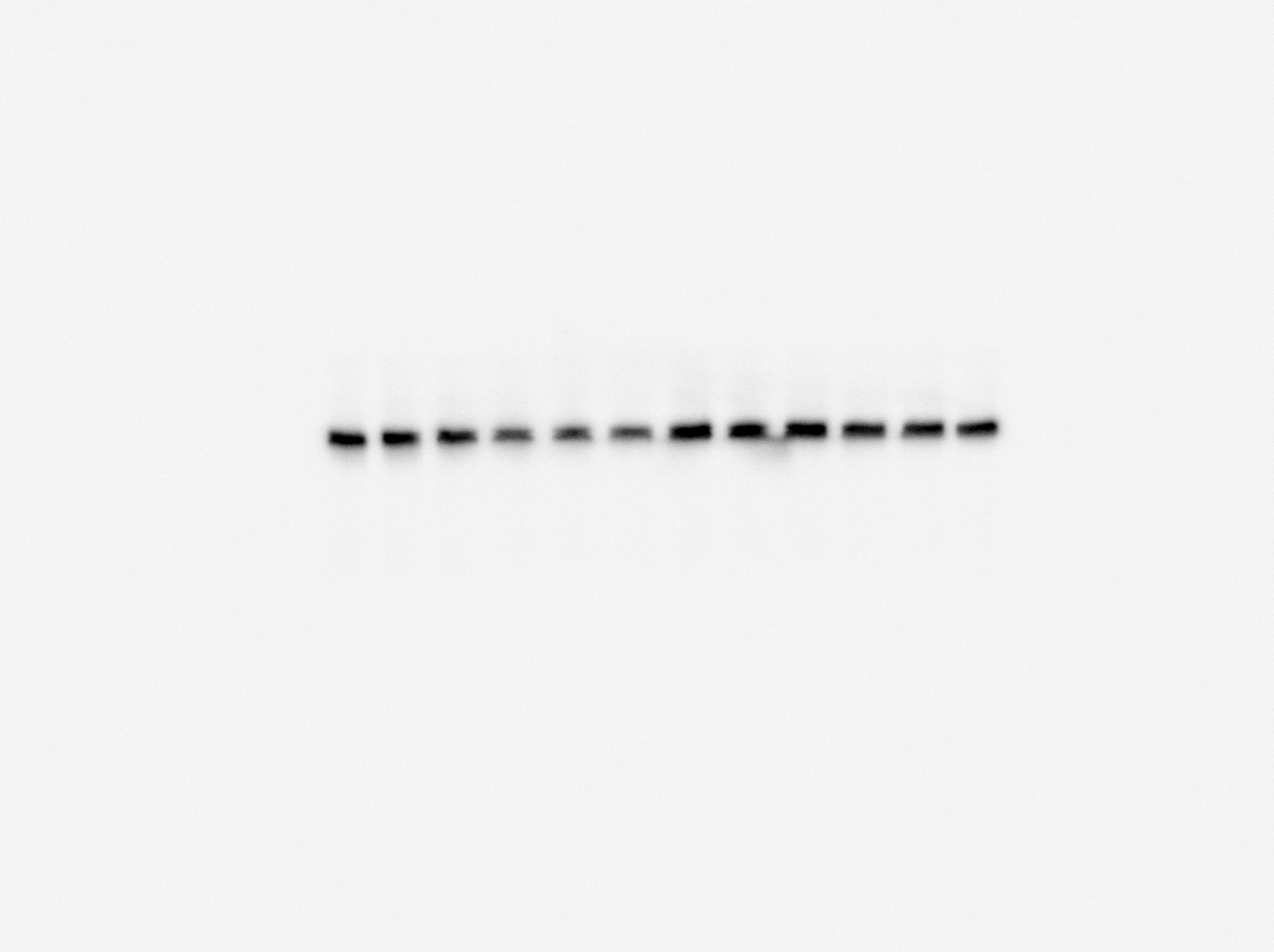

Supplement: Supplementary file 1 [file cancers-13-00862-s001.zip › WBdata_cancers/20200228_8505_E7080U0126_pAKT/200228_8505_E7080U0126_pAKT_a.tif]

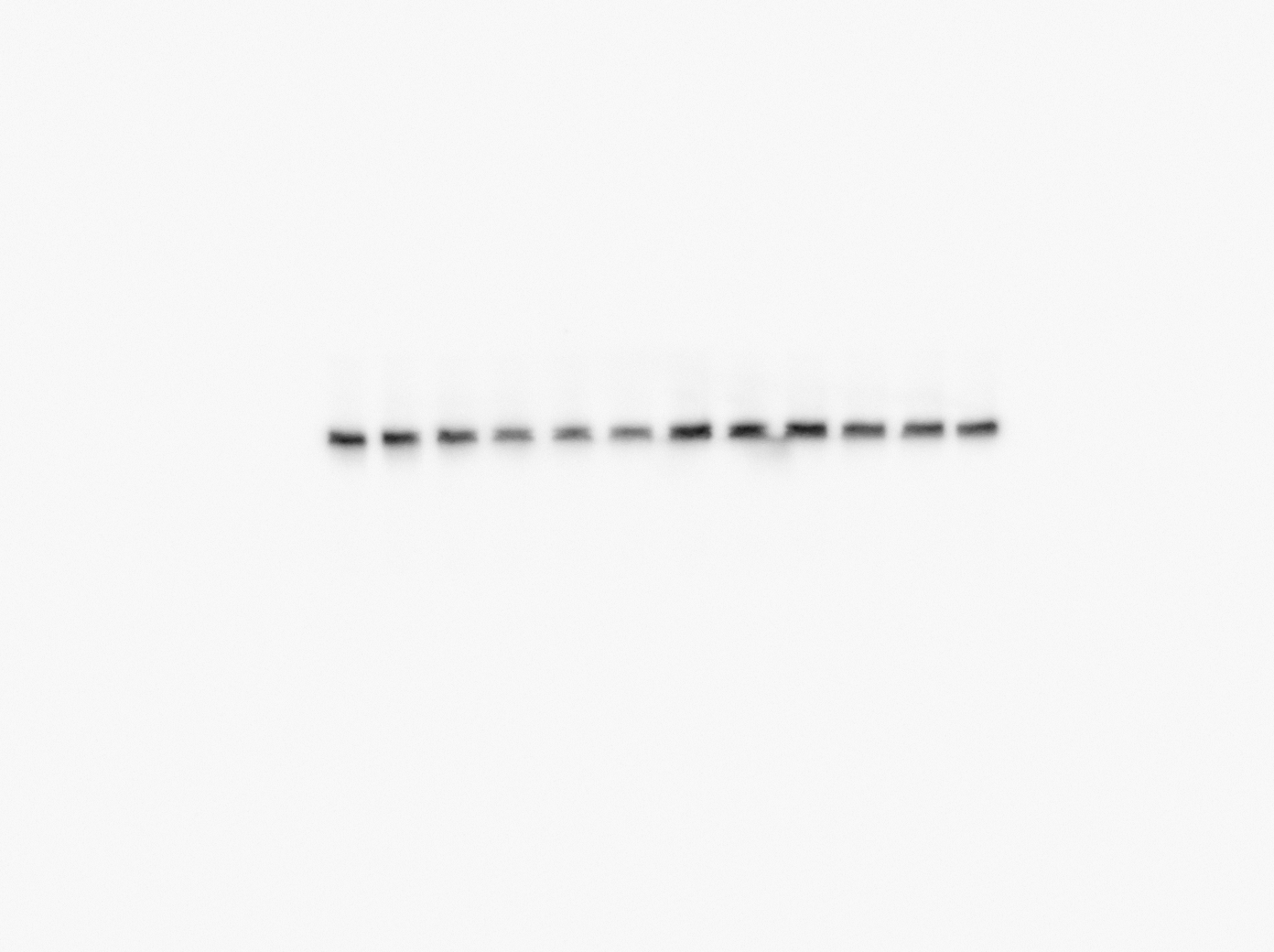

Supplement: Supplementary file 1 [file cancers-13-00862-s001.zip › WBdata_cancers/20200228_8505_E7080U0126_pAKT/200228_8505_E7080U0126_pAKT_b.tif]

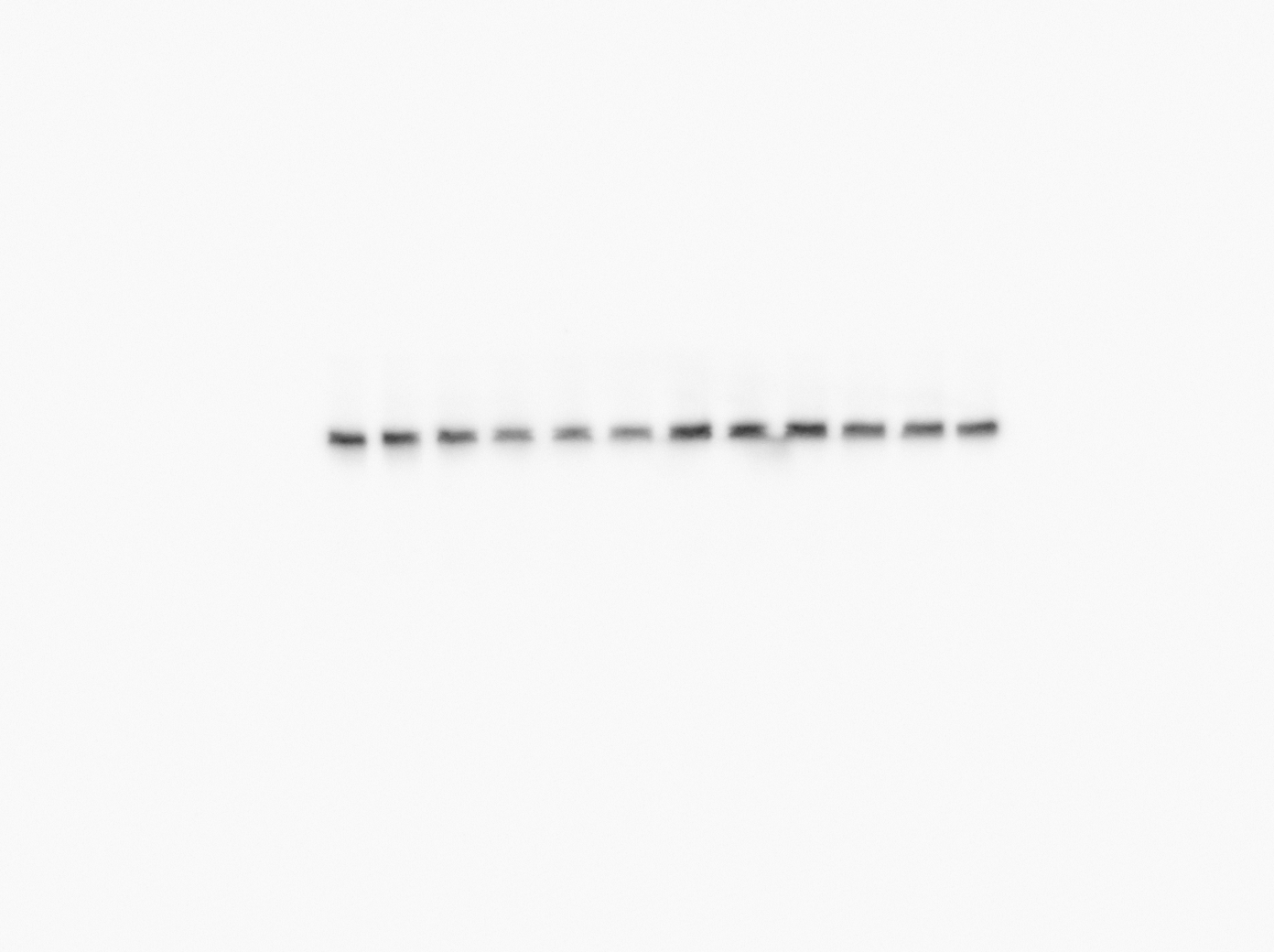

Supplement: Supplementary file 1 [file cancers-13-00862-s001.zip › WBdata_cancers/20200228_8505_E7080U0126_pAKT/200228_8505_E7080U0126_pAKT_c.tif]

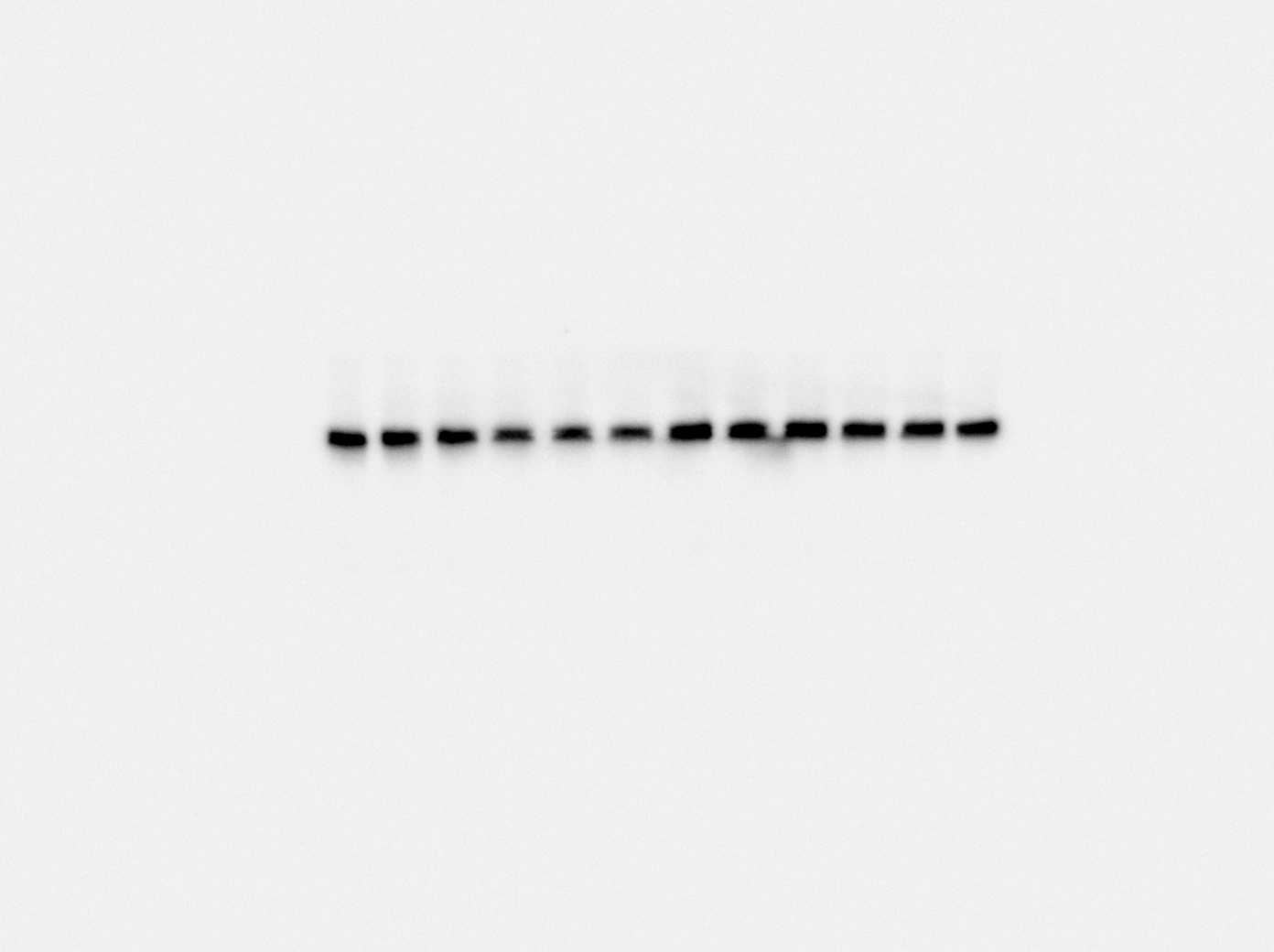

Supplement: Supplementary file 1 [file cancers-13-00862-s001.zip › WBdata_cancers/20200228_8505_E7080U0126_pAKT/200228_8505_E7080U0126_pAKT_d.tif]

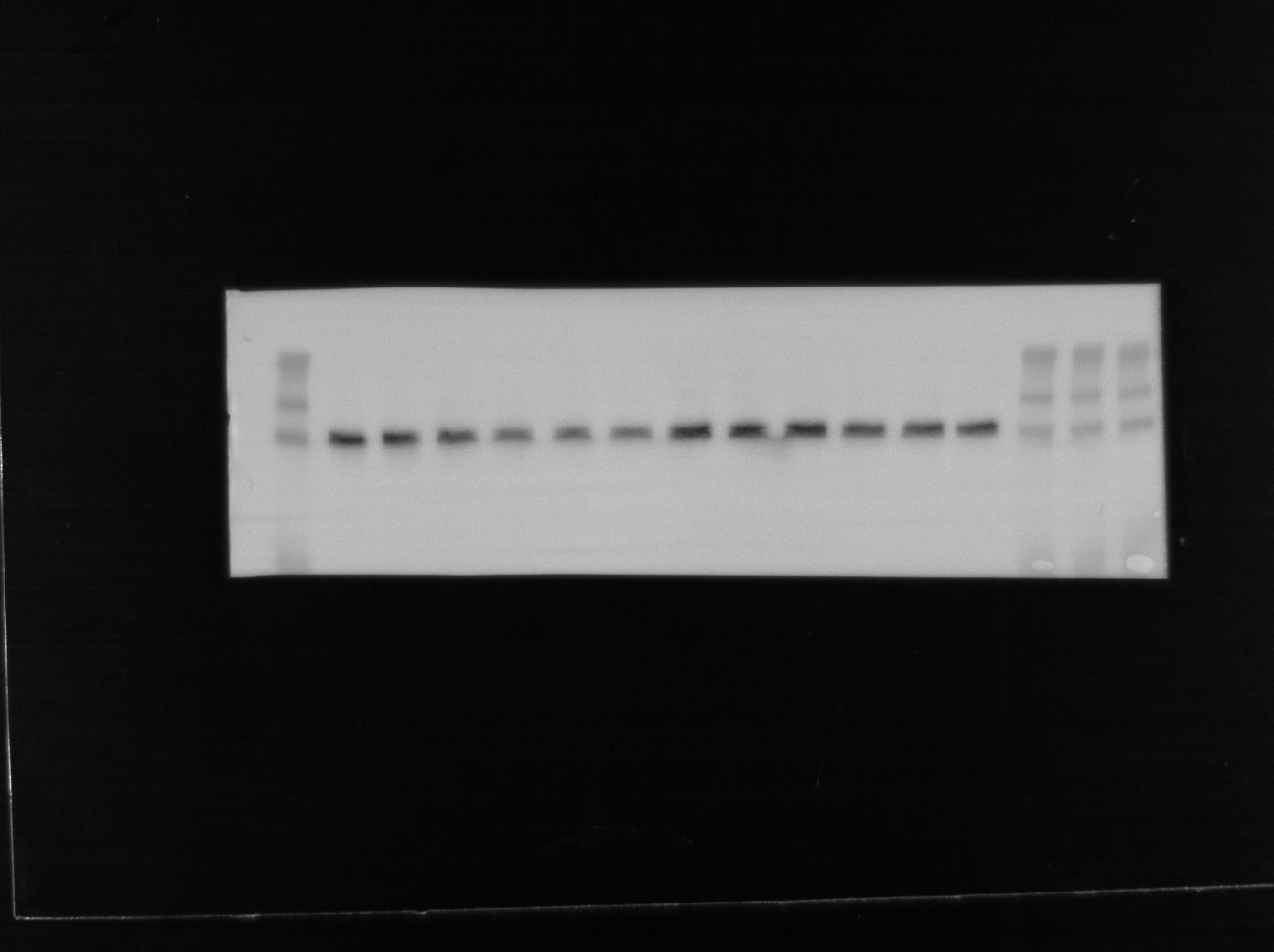

Supplement: Supplementary file 1 [file cancers-13-00862-s001.zip › WBdata_cancers/20200228_8505_E7080U0126_pAKT/200228_8505_E7080U0126_pAKT_Merge.tif]

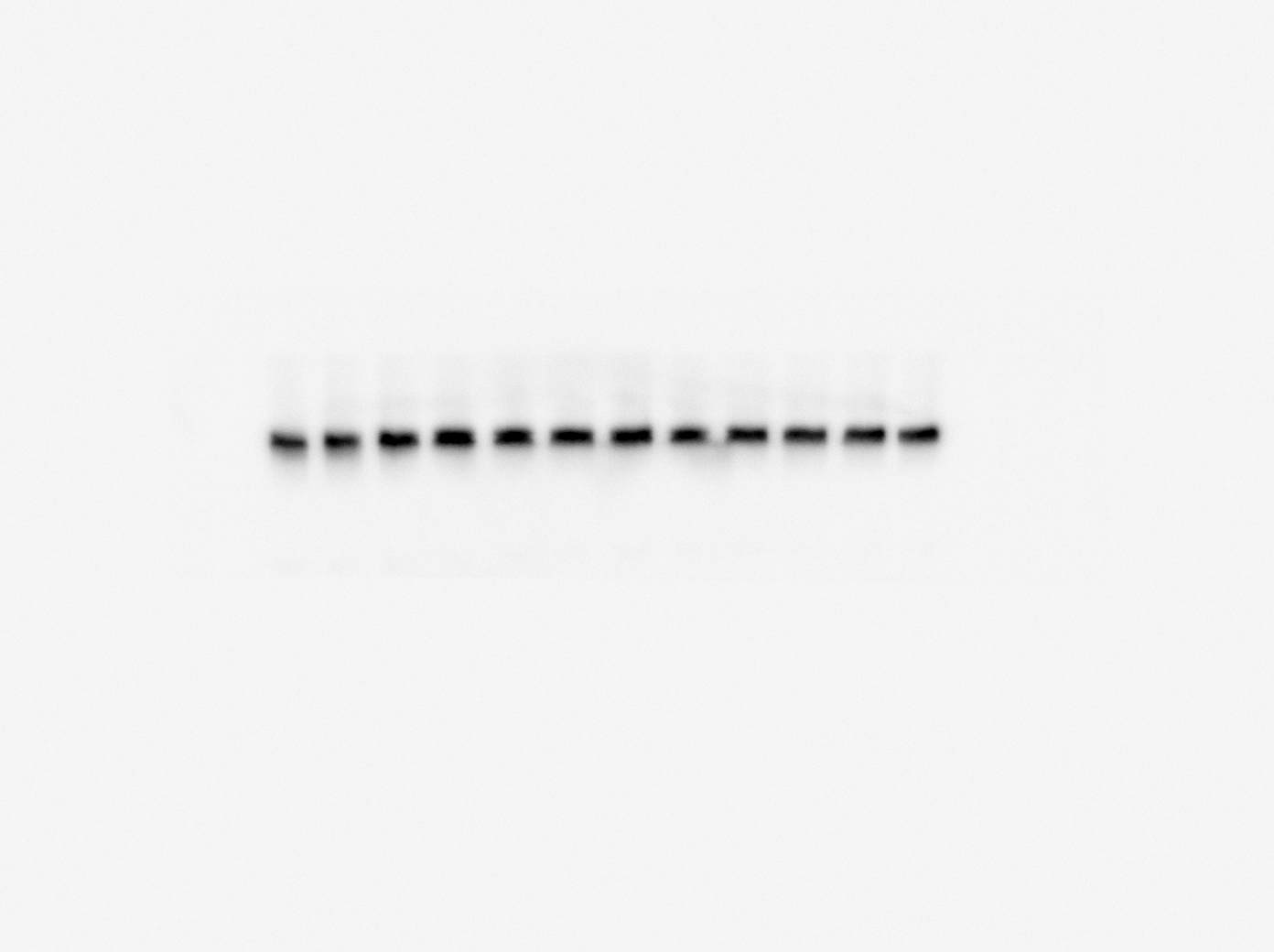

Supplement: Supplementary file 1 [file cancers-13-00862-s001.zip › WBdata_cancers/20200228_8505_E7080U0126_tAKT/20200228_8505_E7080U0126_tAKT_a.tif]

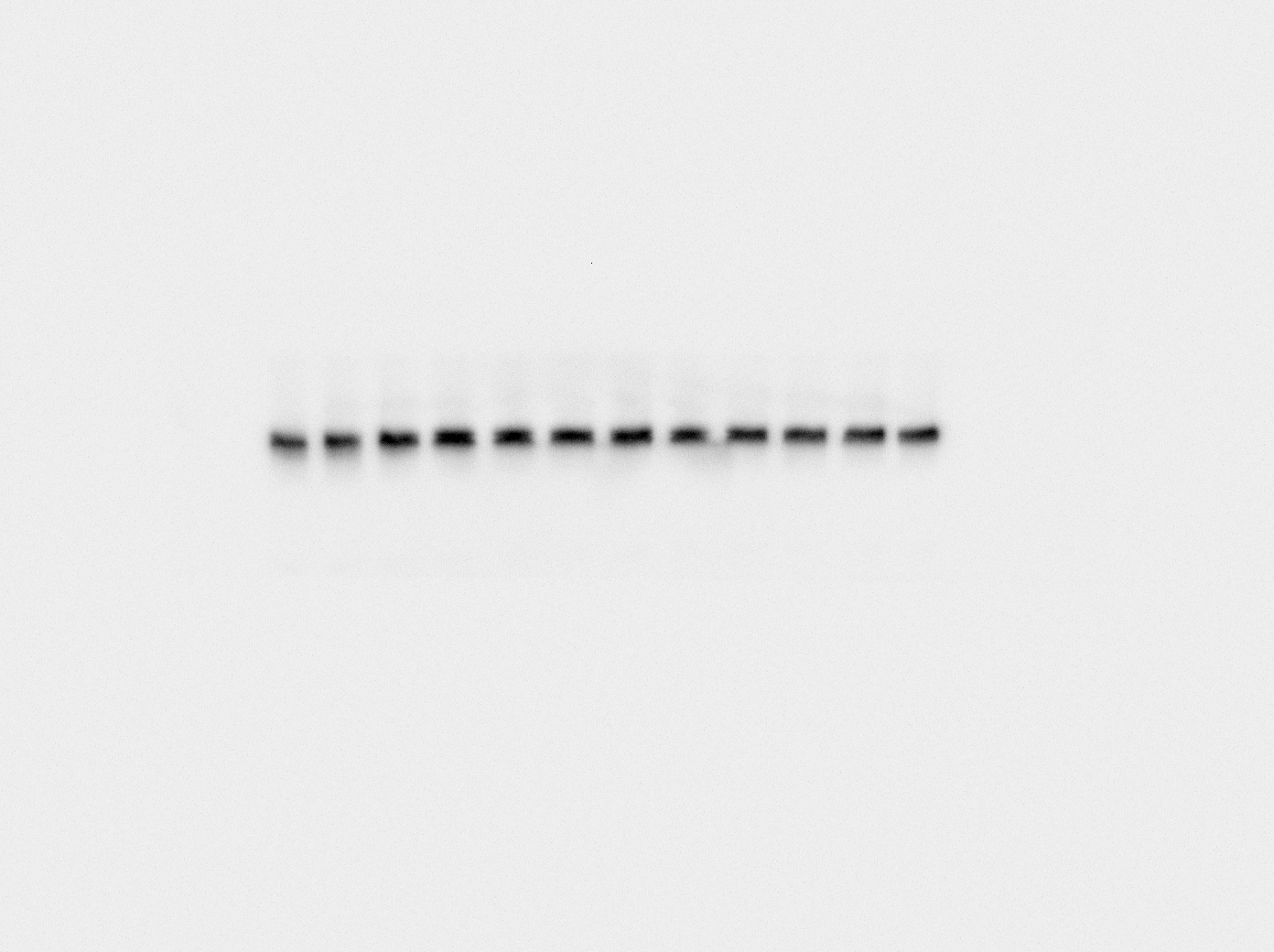

Supplement: Supplementary file 1 [file cancers-13-00862-s001.zip › WBdata_cancers/20200228_8505_E7080U0126_tAKT/20200228_8505_E7080U0126_tAKT_b.tif]

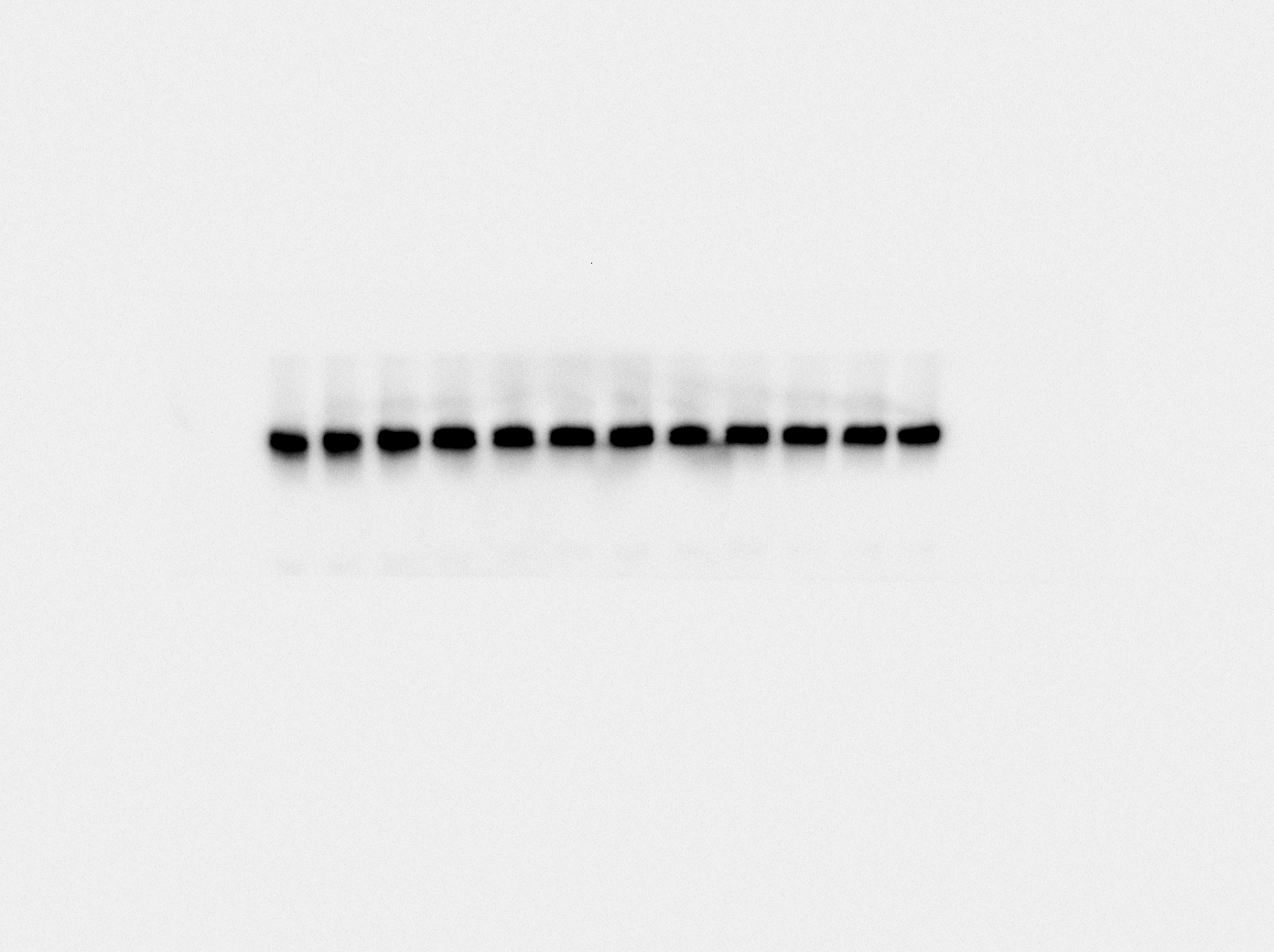

Supplement: Supplementary file 1 [file cancers-13-00862-s001.zip › WBdata_cancers/20200228_8505_E7080U0126_tAKT/20200228_8505_E7080U0126_tAKT_c.tif]

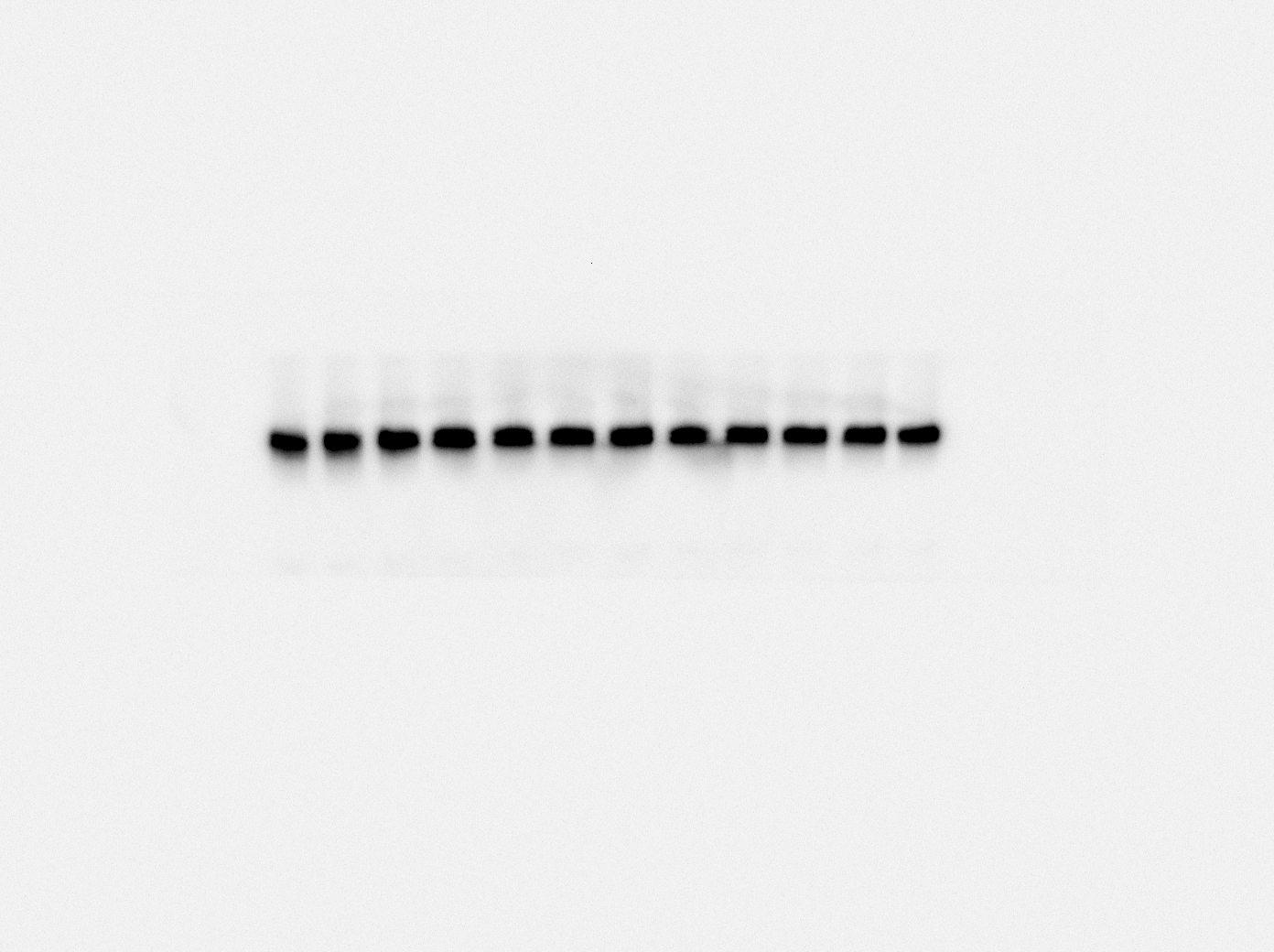

Supplement: Supplementary file 1 [file cancers-13-00862-s001.zip › WBdata_cancers/20200228_8505_E7080U0126_tAKT/20200228_8505_E7080U0126_tAKT_d.tif]

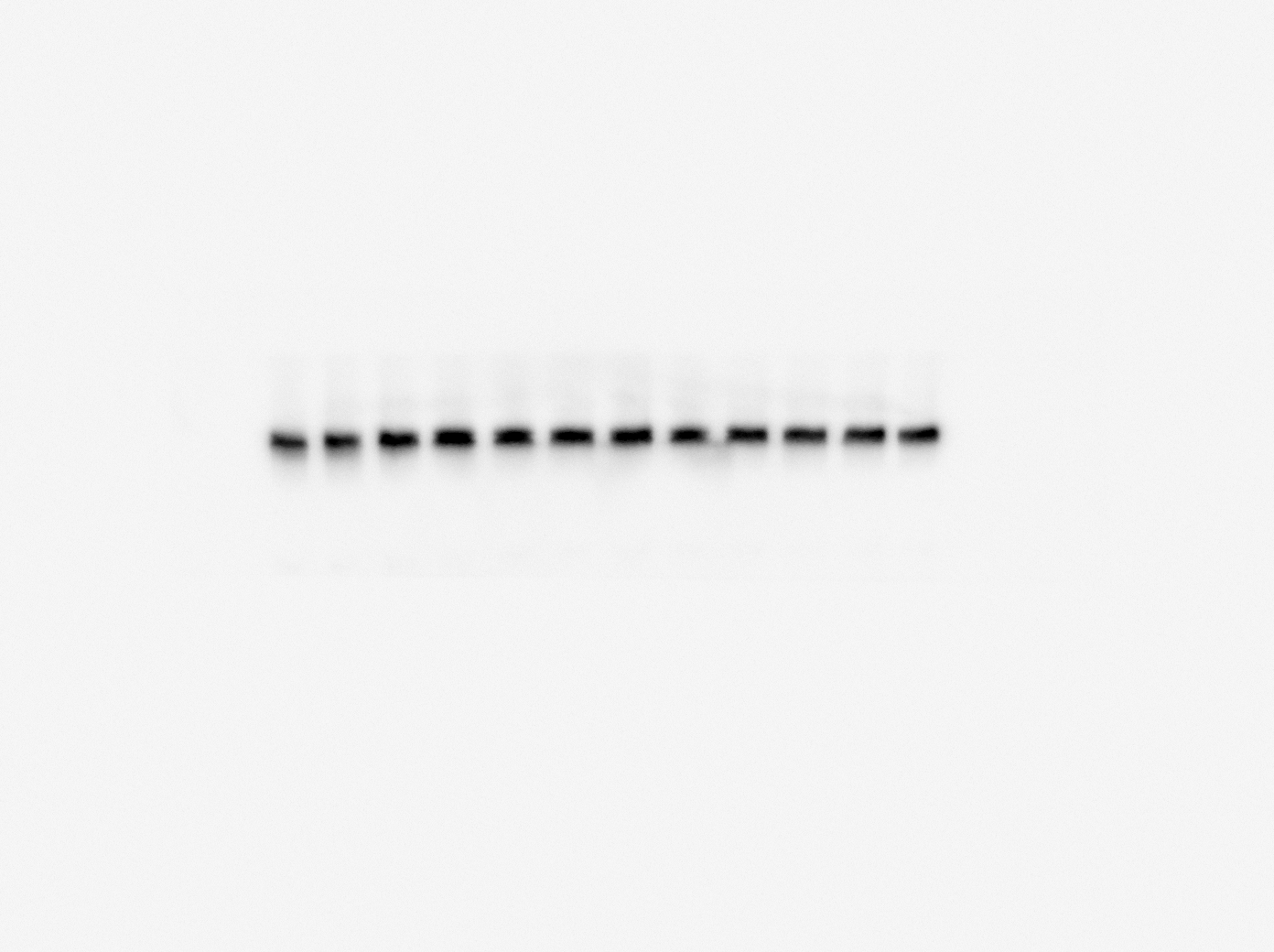

Supplement: Supplementary file 1 [file cancers-13-00862-s001.zip › WBdata_cancers/20200228_8505_E7080U0126_tAKT/20200228_8505_E7080U0126_tAKT_e.tif]

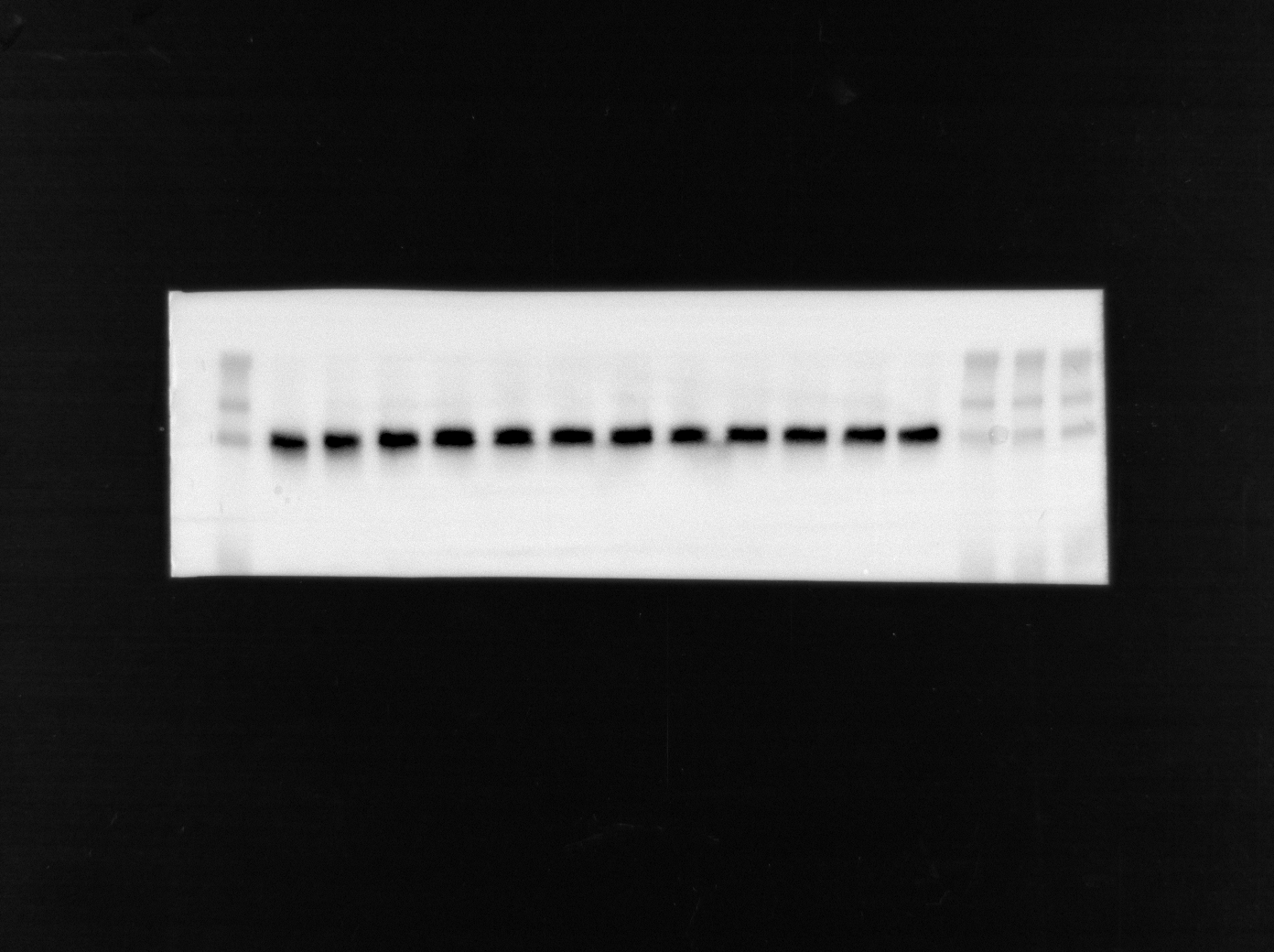

Supplement: Supplementary file 1 [file cancers-13-00862-s001.zip › WBdata_cancers/20200228_8505_E7080U0126_tAKT/20200228_8505_E7080U0126_tAKT_Merge.tif]

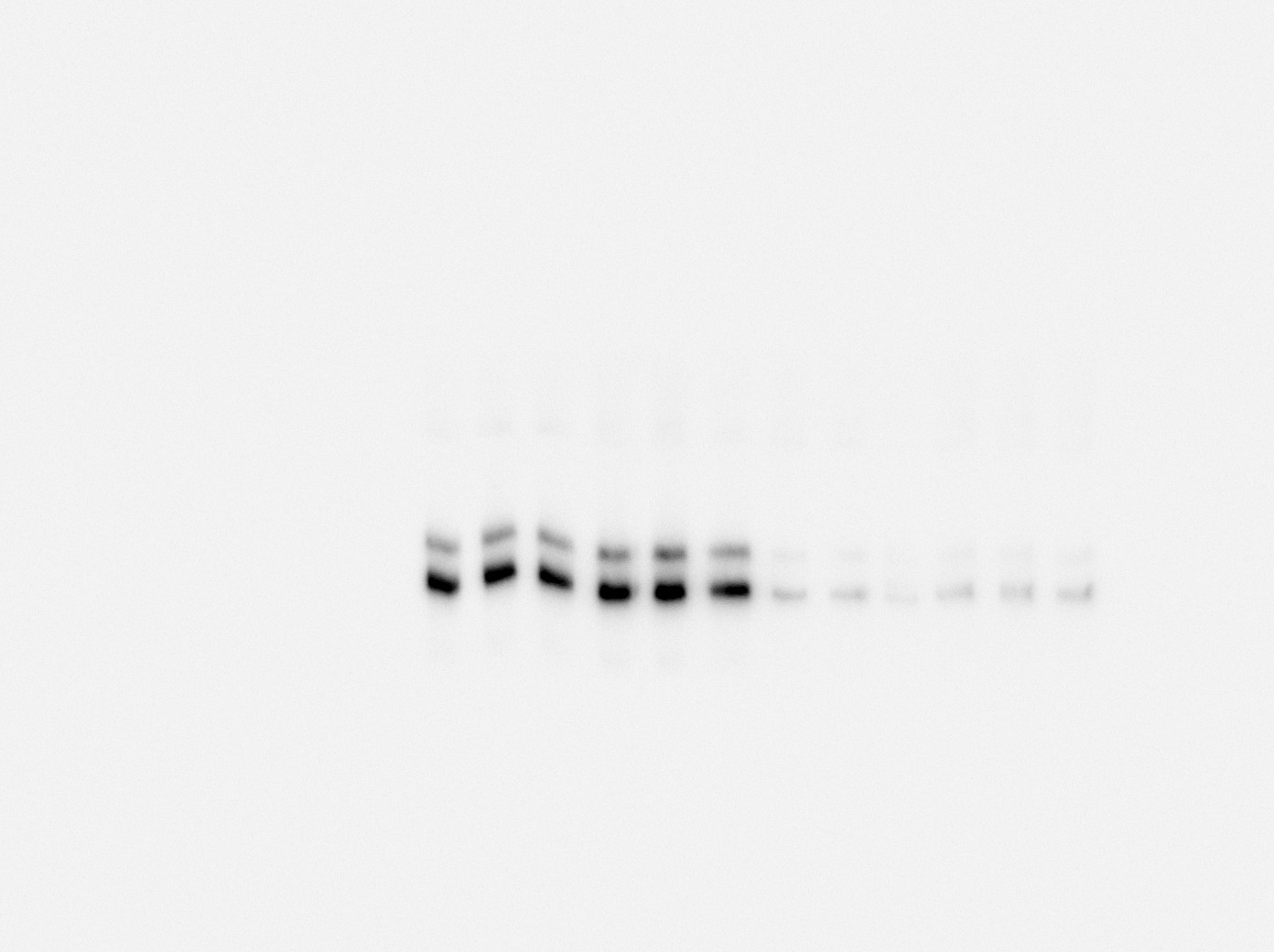

Supplement: Supplementary file 1 [file cancers-13-00862-s001.zip › WBdata_cancers/20200229_8505_E7080U0126_pERK/20200229_8505_E7080U0126_pERK_a.tif]

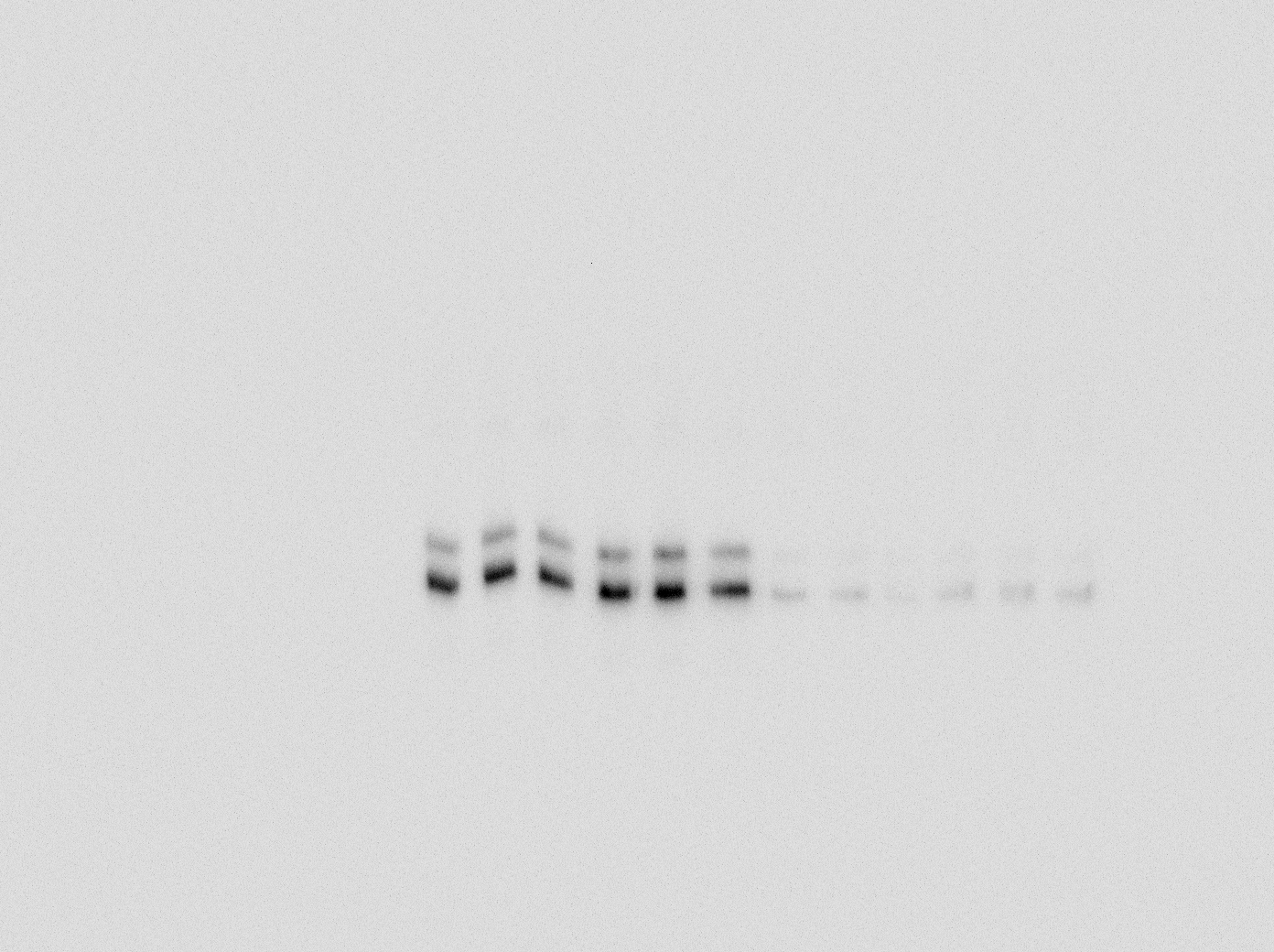

Supplement: Supplementary file 1 [file cancers-13-00862-s001.zip › WBdata_cancers/20200229_8505_E7080U0126_pERK/20200229_8505_E7080U0126_pERK_b.tif]

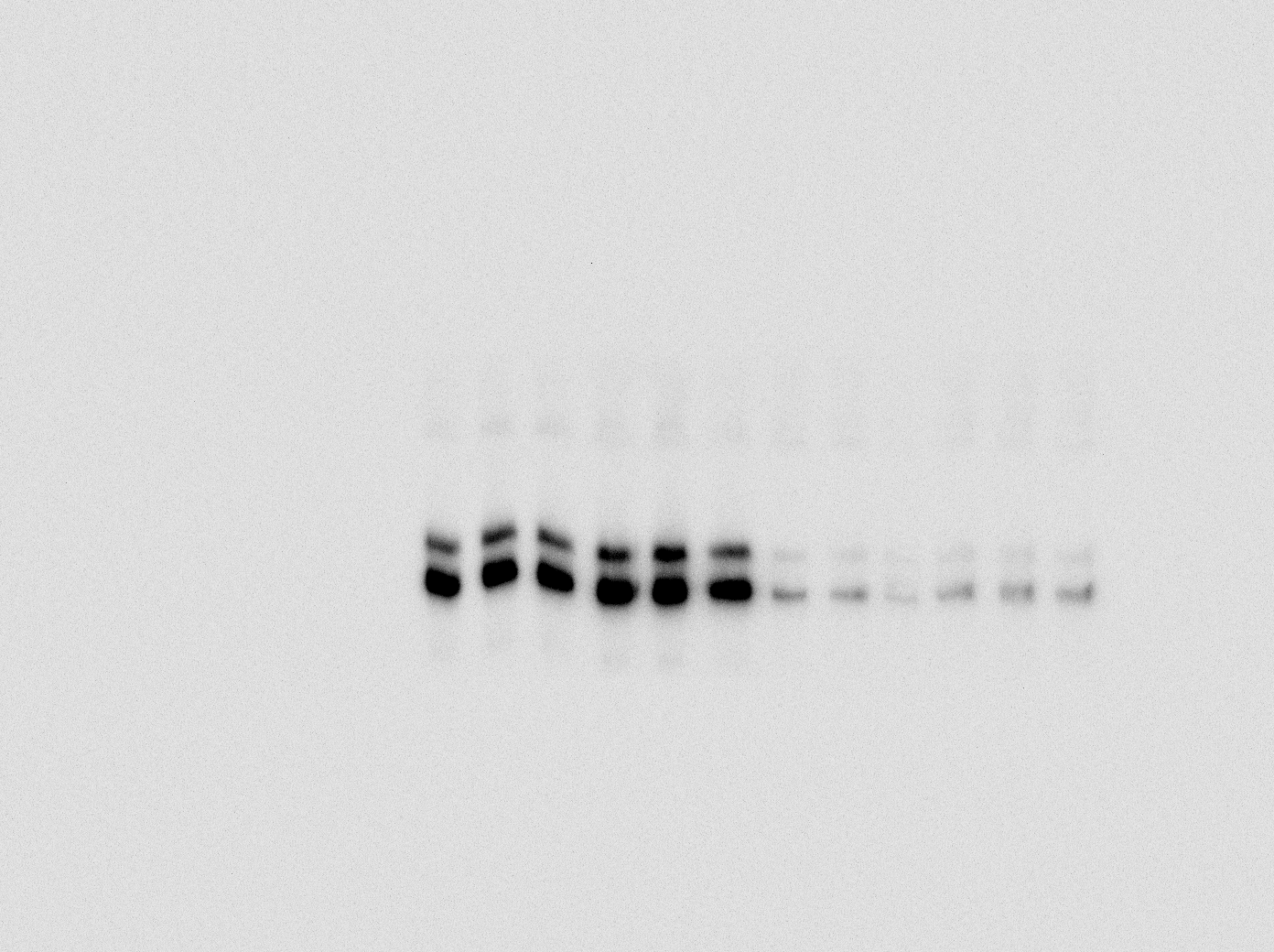

Supplement: Supplementary file 1 [file cancers-13-00862-s001.zip › WBdata_cancers/20200229_8505_E7080U0126_pERK/20200229_8505_E7080U0126_pERK_c.tif]

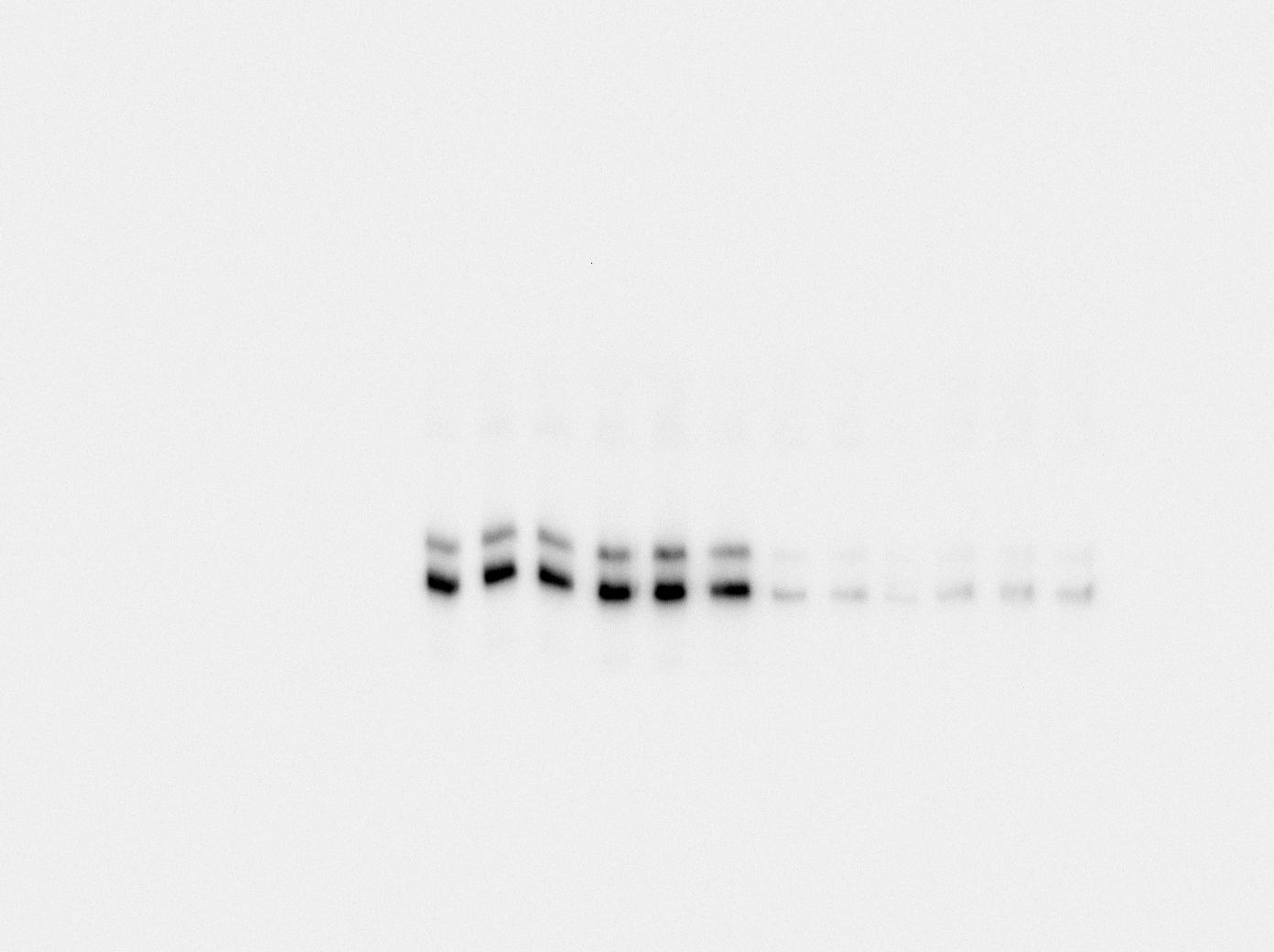

Supplement: Supplementary file 1 [file cancers-13-00862-s001.zip › WBdata_cancers/20200229_8505_E7080U0126_pERK/20200229_8505_E7080U0126_pERK_d.tif]

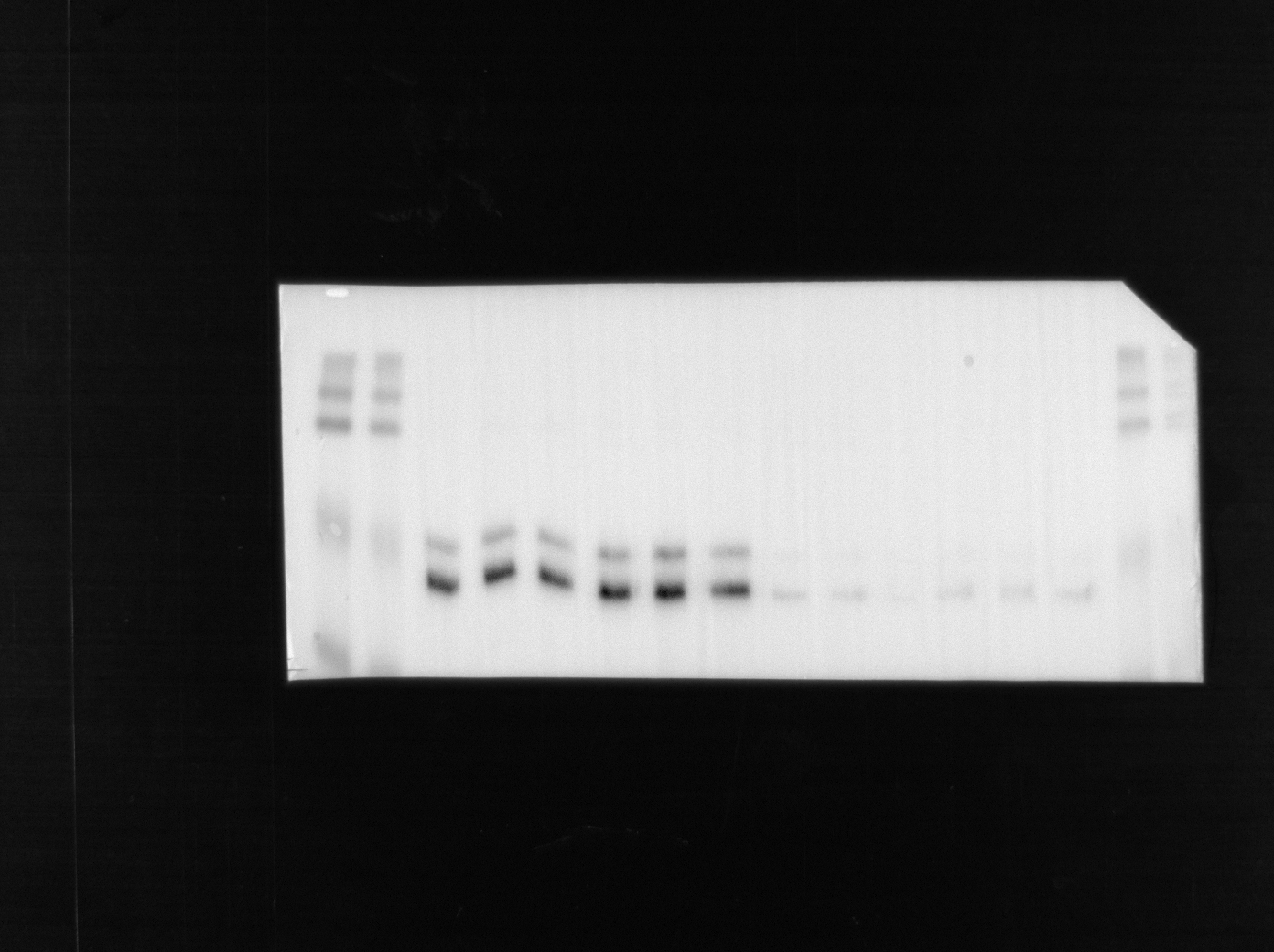

Supplement: Supplementary file 1 [file cancers-13-00862-s001.zip › WBdata_cancers/20200229_8505_E7080U0126_pERK/20200229_8505_E7080U0126_pERK_Merge.tif]

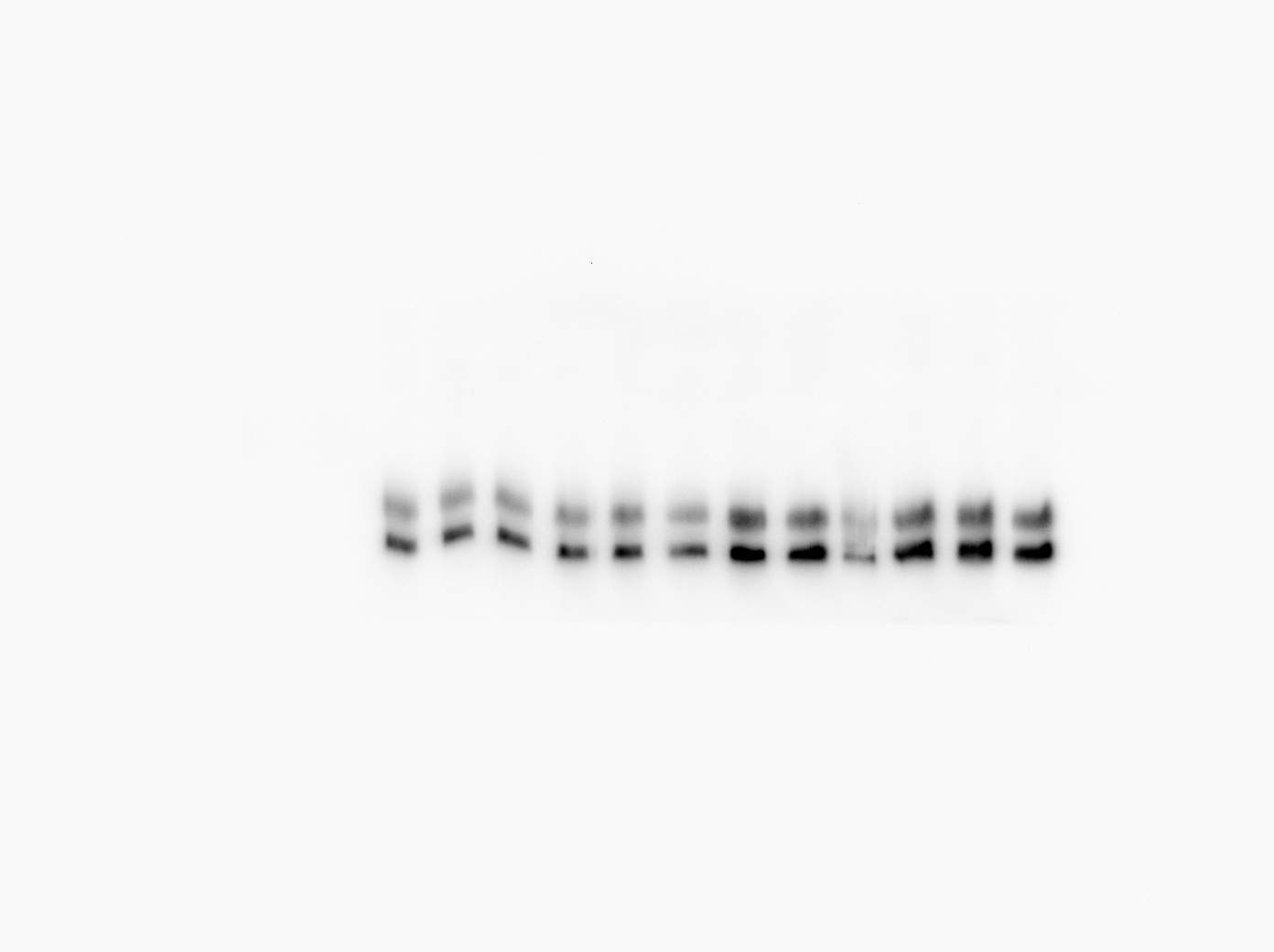

Supplement: Supplementary file 1 [file cancers-13-00862-s001.zip › WBdata_cancers/20200229_8505_E7080U0126_tERK/20200229_8505_E7080U0126_tERK_a.tif]

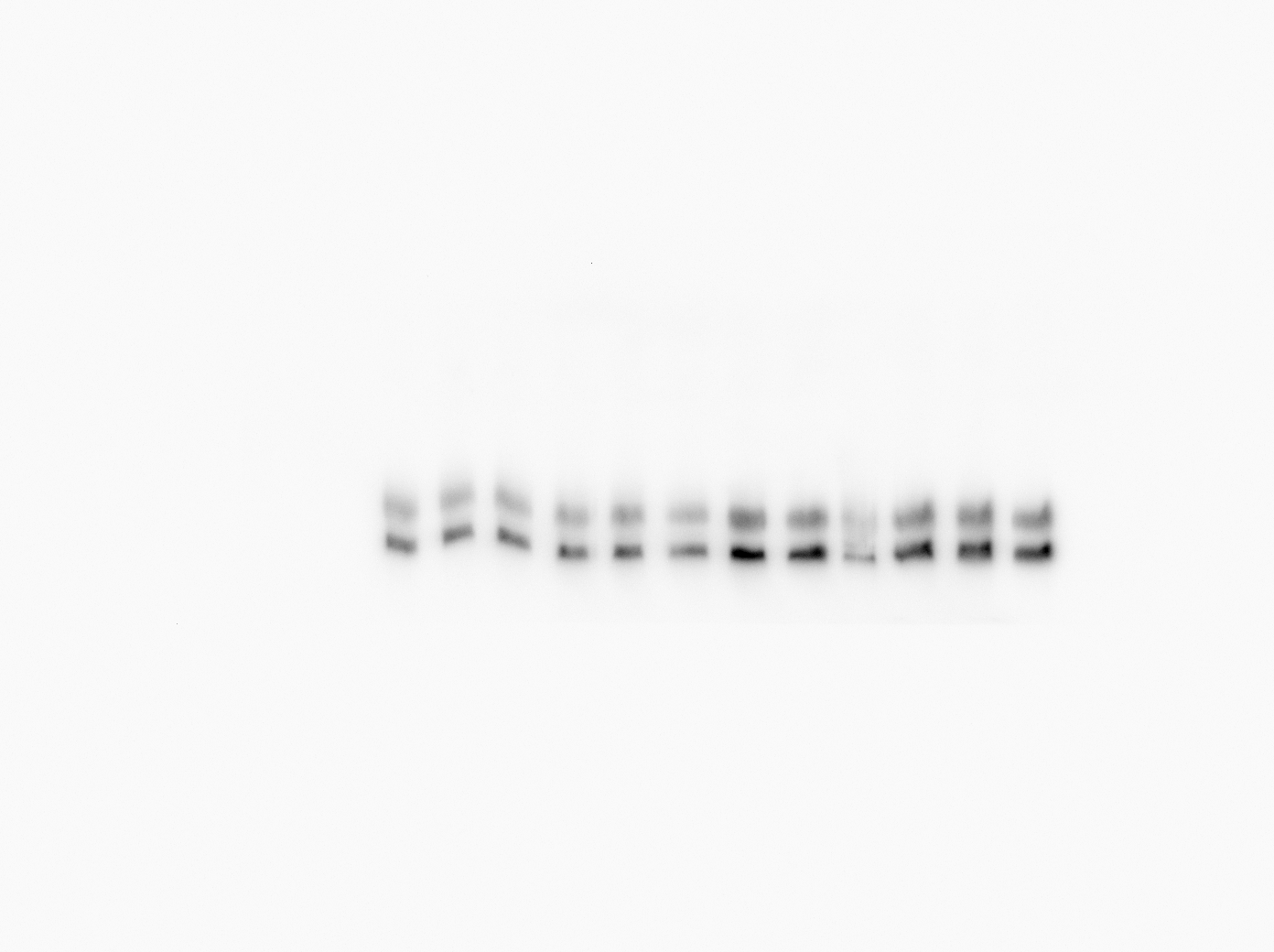

Supplement: Supplementary file 1 [file cancers-13-00862-s001.zip › WBdata_cancers/20200229_8505_E7080U0126_tERK/20200229_8505_E7080U0126_tERK_b.tif]

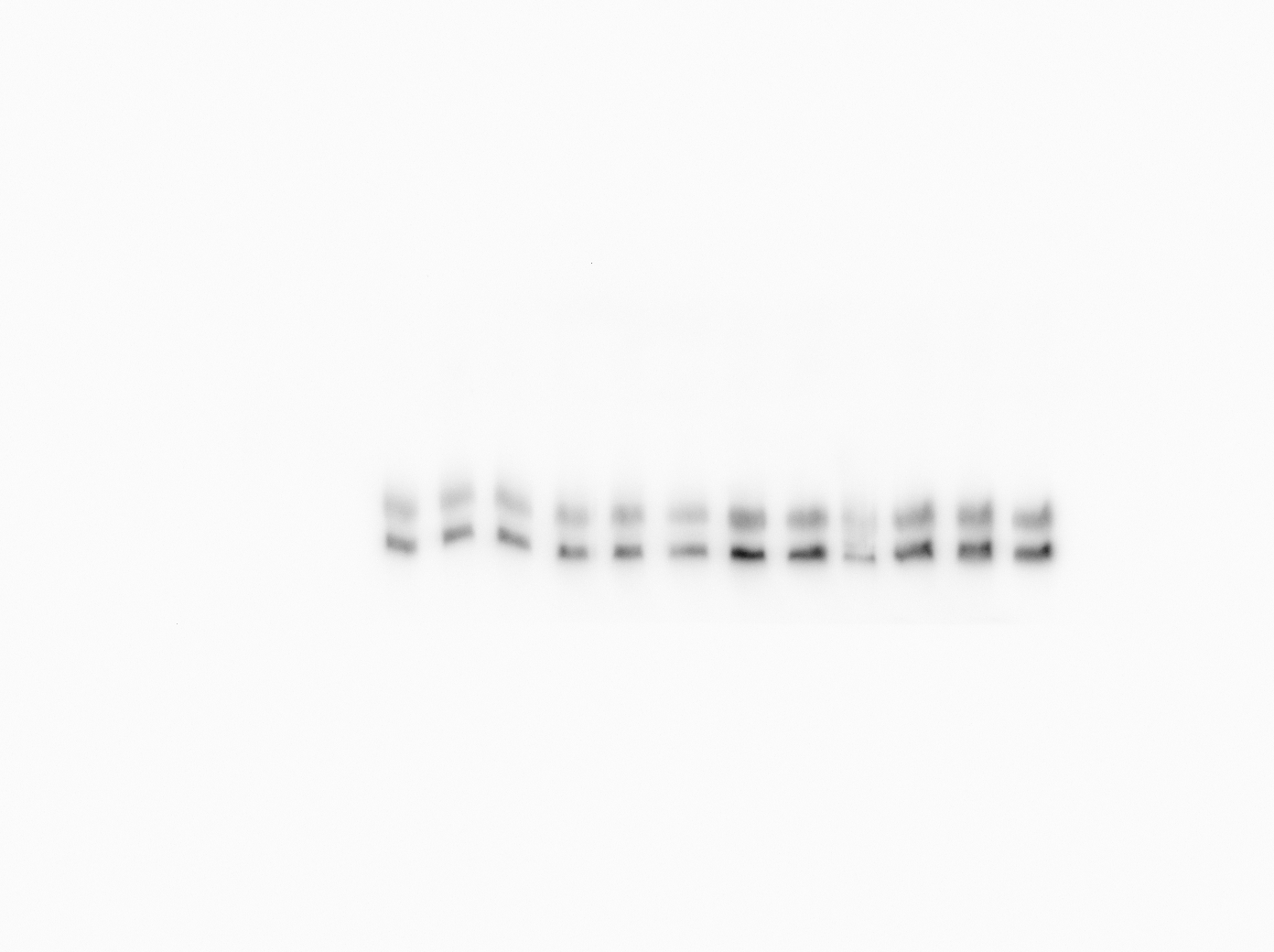

Supplement: Supplementary file 1 [file cancers-13-00862-s001.zip › WBdata_cancers/20200229_8505_E7080U0126_tERK/20200229_8505_E7080U0126_tERK_c.tif]

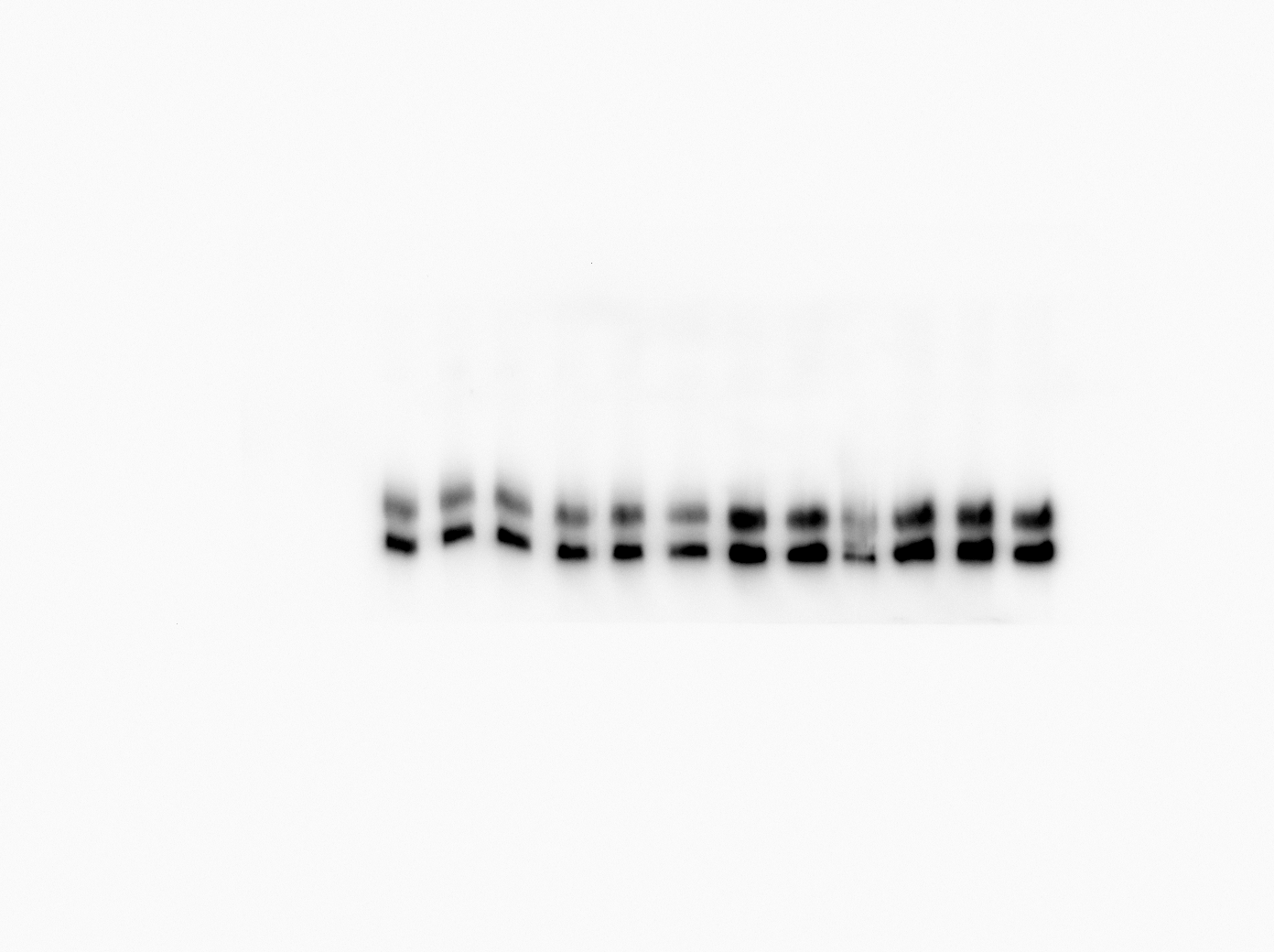

Supplement: Supplementary file 1 [file cancers-13-00862-s001.zip › WBdata_cancers/20200229_8505_E7080U0126_tERK/20200229_8505_E7080U0126_tERK_d.tif]

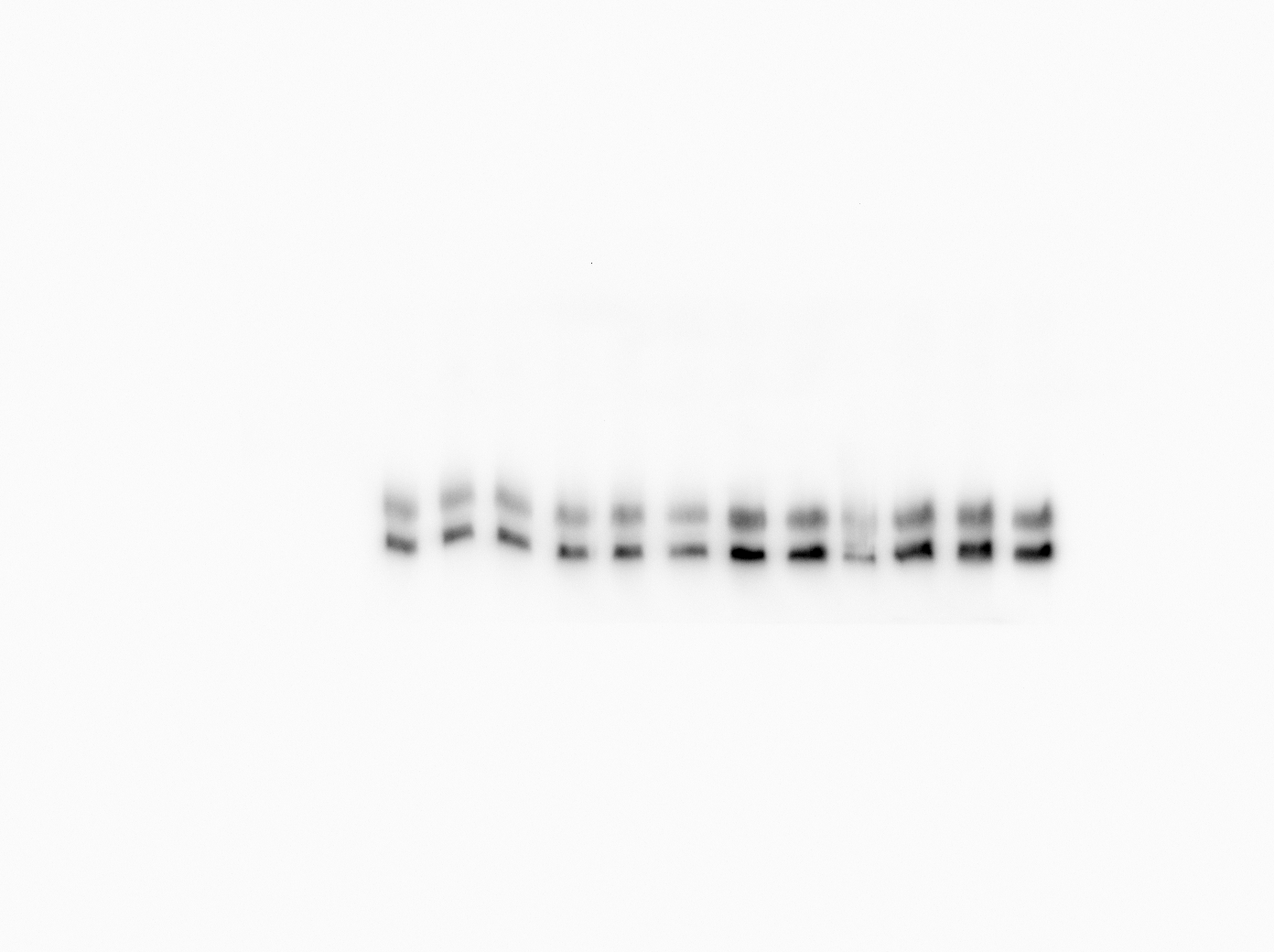

Supplement: Supplementary file 1 [file cancers-13-00862-s001.zip › WBdata_cancers/20200229_8505_E7080U0126_tERK/20200229_8505_E7080U0126_tERK_e.tif]

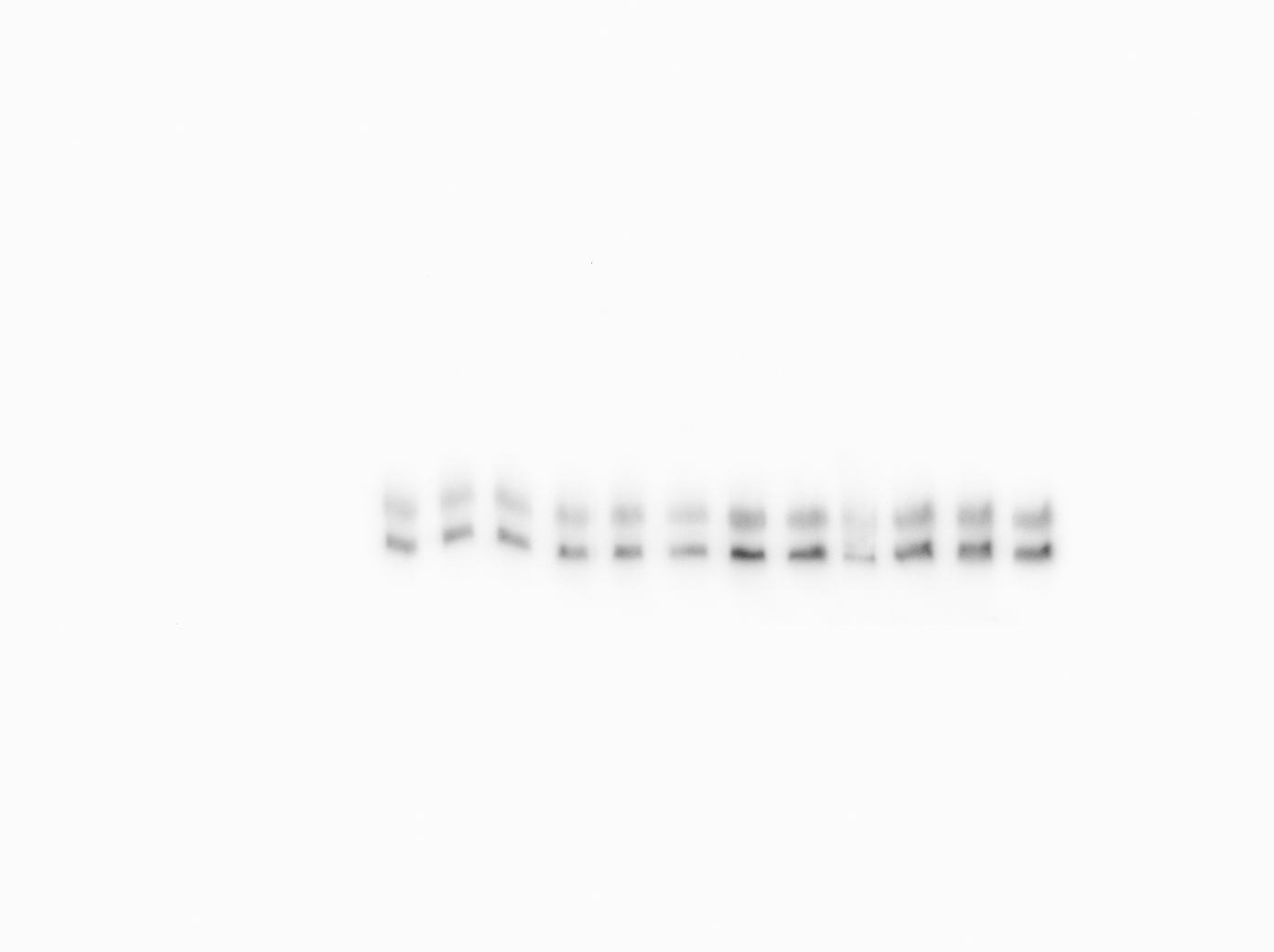

Supplement: Supplementary file 1 [file cancers-13-00862-s001.zip › WBdata_cancers/20200229_8505_E7080U0126_tERK/20200229_8505_E7080U0126_tERK_f.tif]

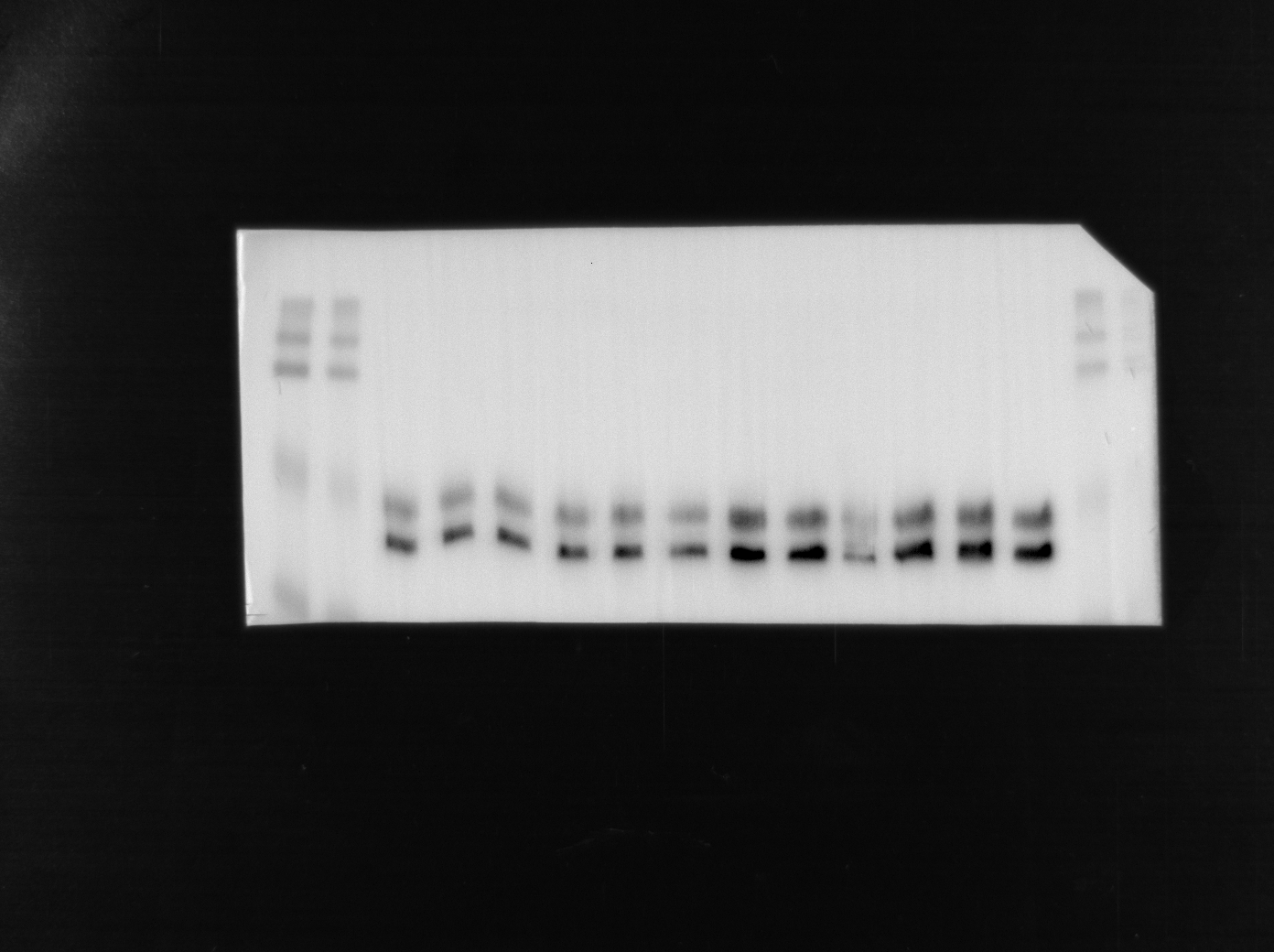

Supplement: Supplementary file 1 [file cancers-13-00862-s001.zip › WBdata_cancers/20200229_8505_E7080U0126_tERK/20200229_8505_E7080U0126_tERK_Merge.tif]

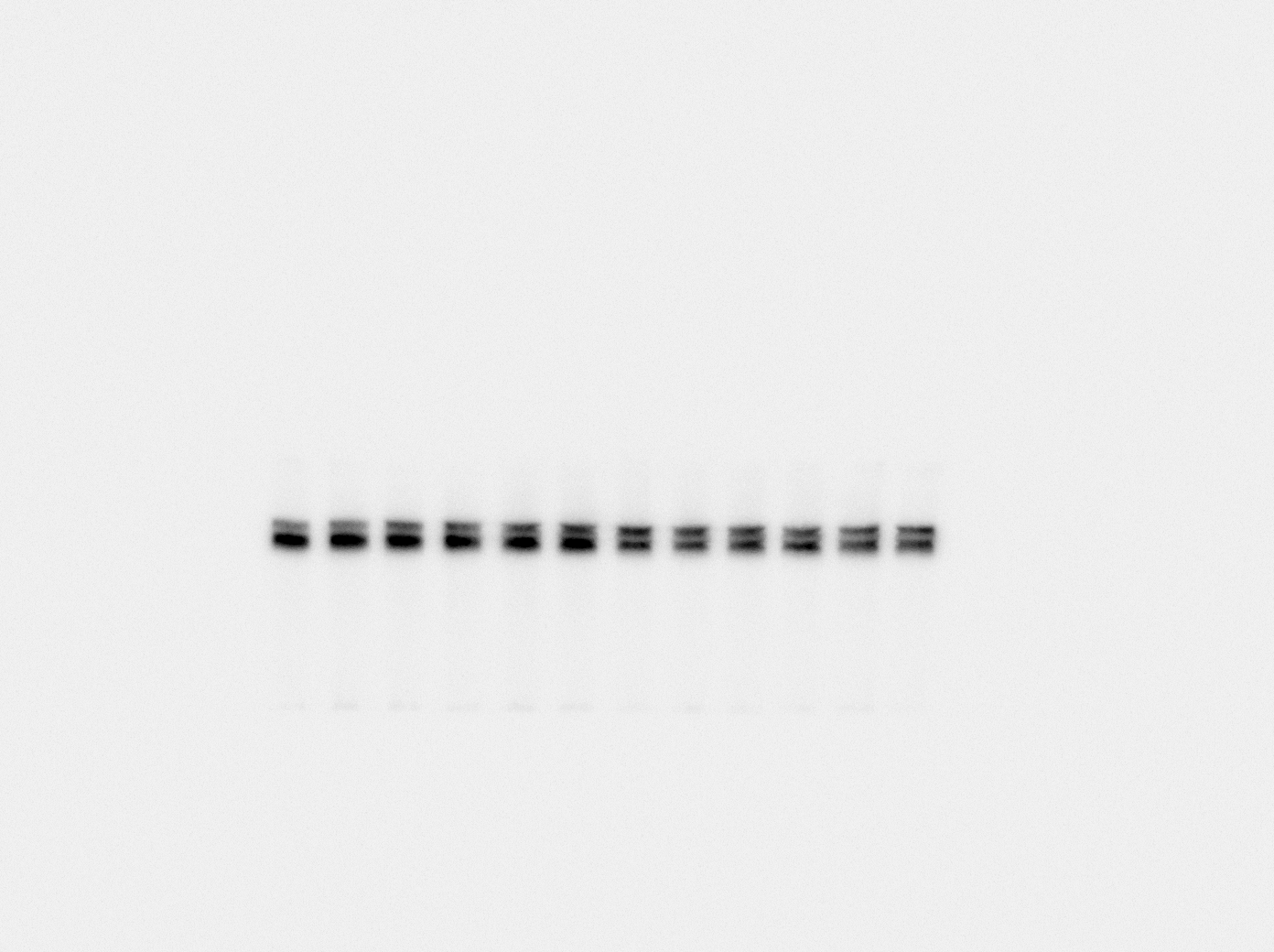

Supplement: Supplementary file 1 [file cancers-13-00862-s001.zip › WBdata_cancers/20200301_8505_E7080U0126_E2F1/20200301_8505_E7080U0126_E2F1_a.tif]

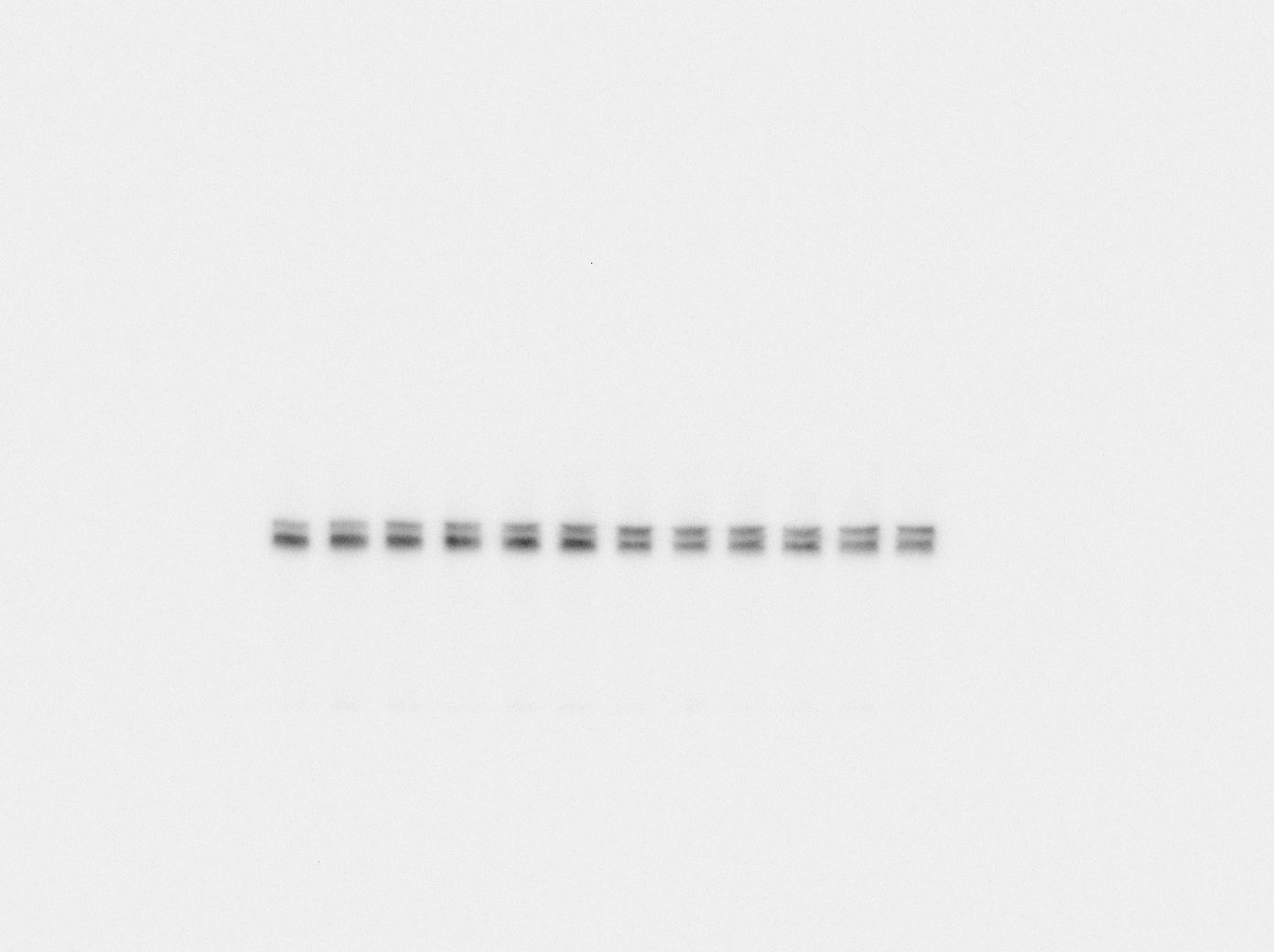

Supplement: Supplementary file 1 [file cancers-13-00862-s001.zip › WBdata_cancers/20200301_8505_E7080U0126_E2F1/20200301_8505_E7080U0126_E2F1_b.tif]

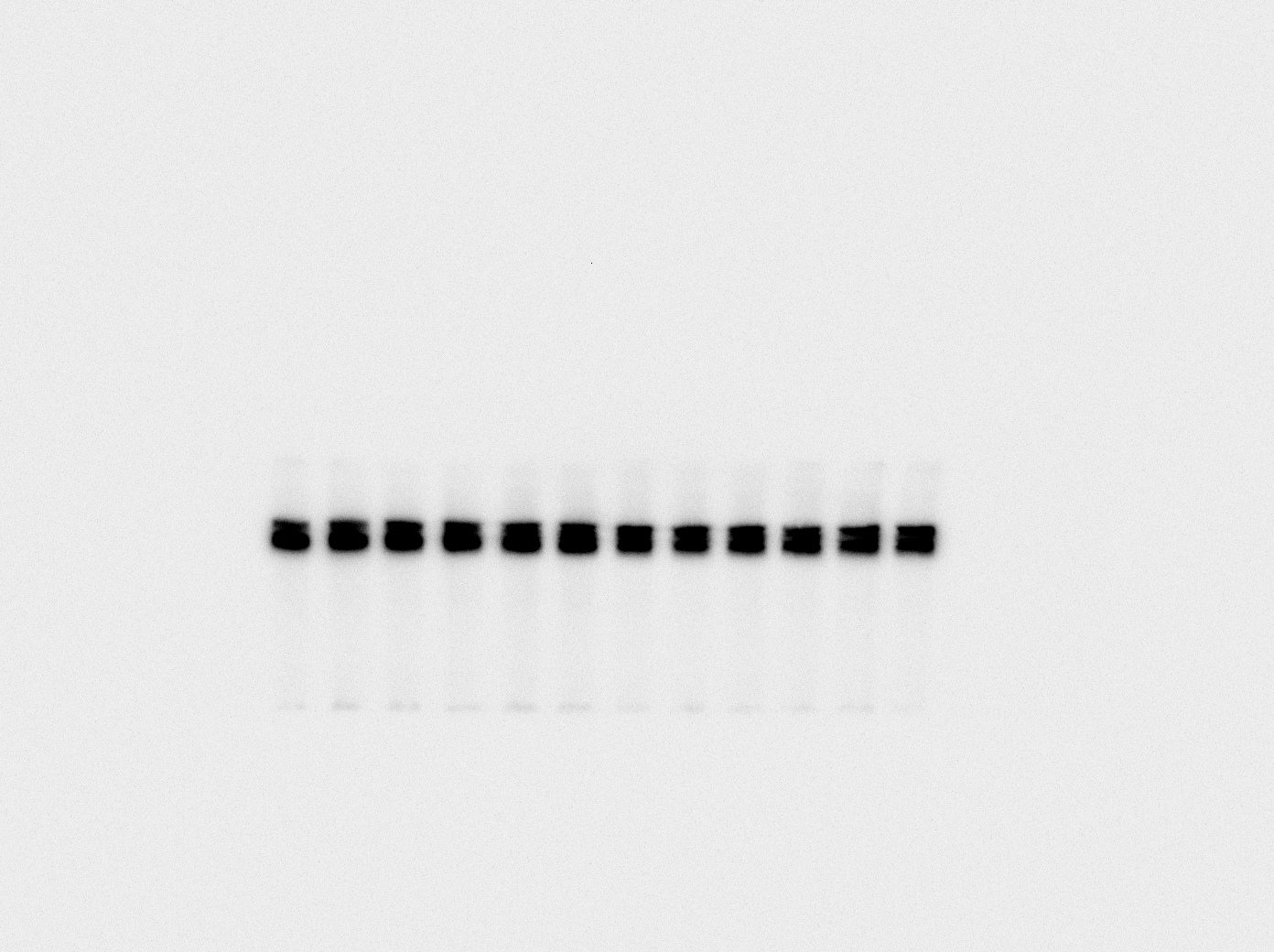

Supplement: Supplementary file 1 [file cancers-13-00862-s001.zip › WBdata_cancers/20200301_8505_E7080U0126_E2F1/20200301_8505_E7080U0126_E2F1_c.tif]

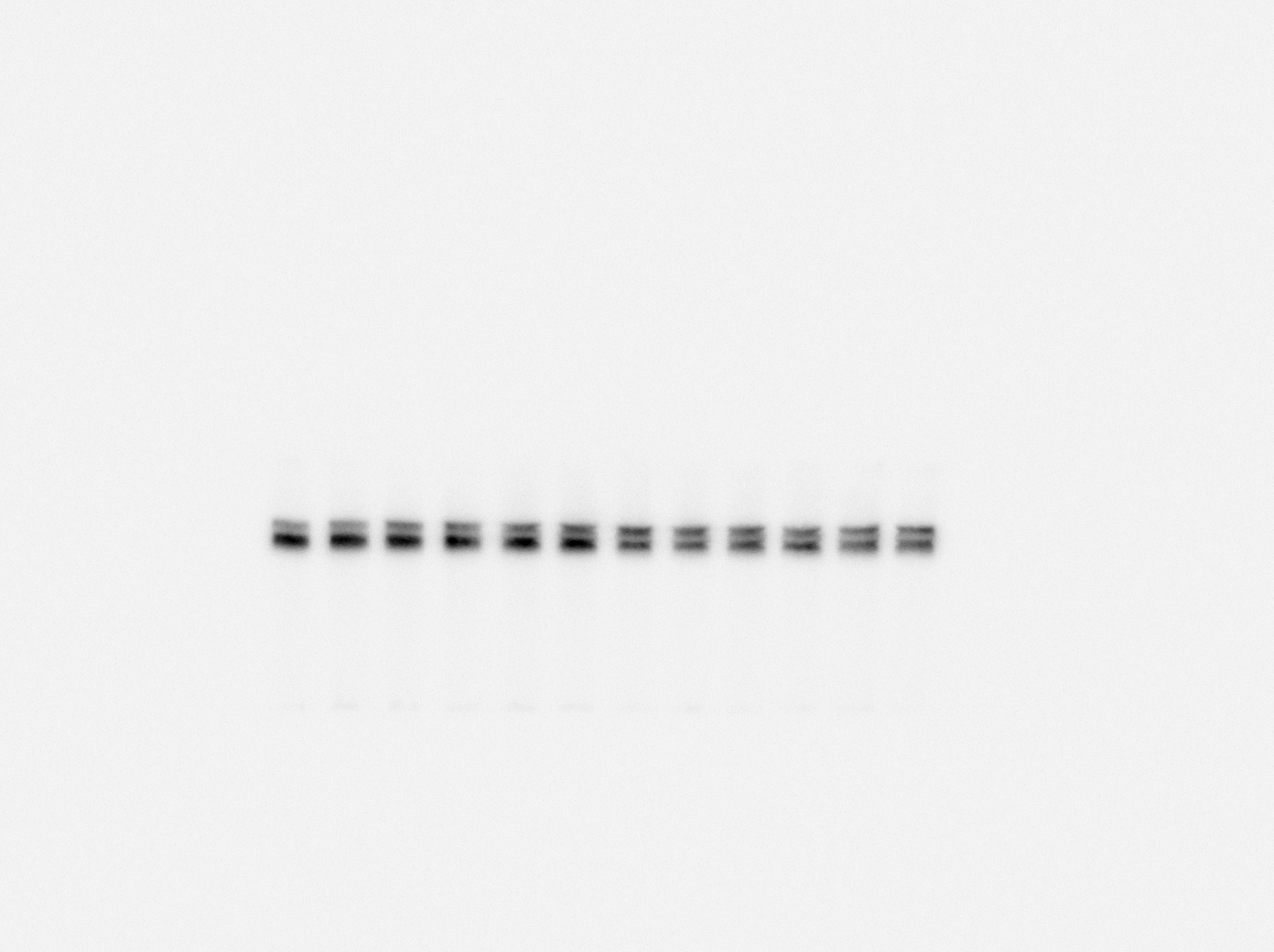

Supplement: Supplementary file 1 [file cancers-13-00862-s001.zip › WBdata_cancers/20200301_8505_E7080U0126_E2F1/20200301_8505_E7080U0126_E2F1_d.tif]

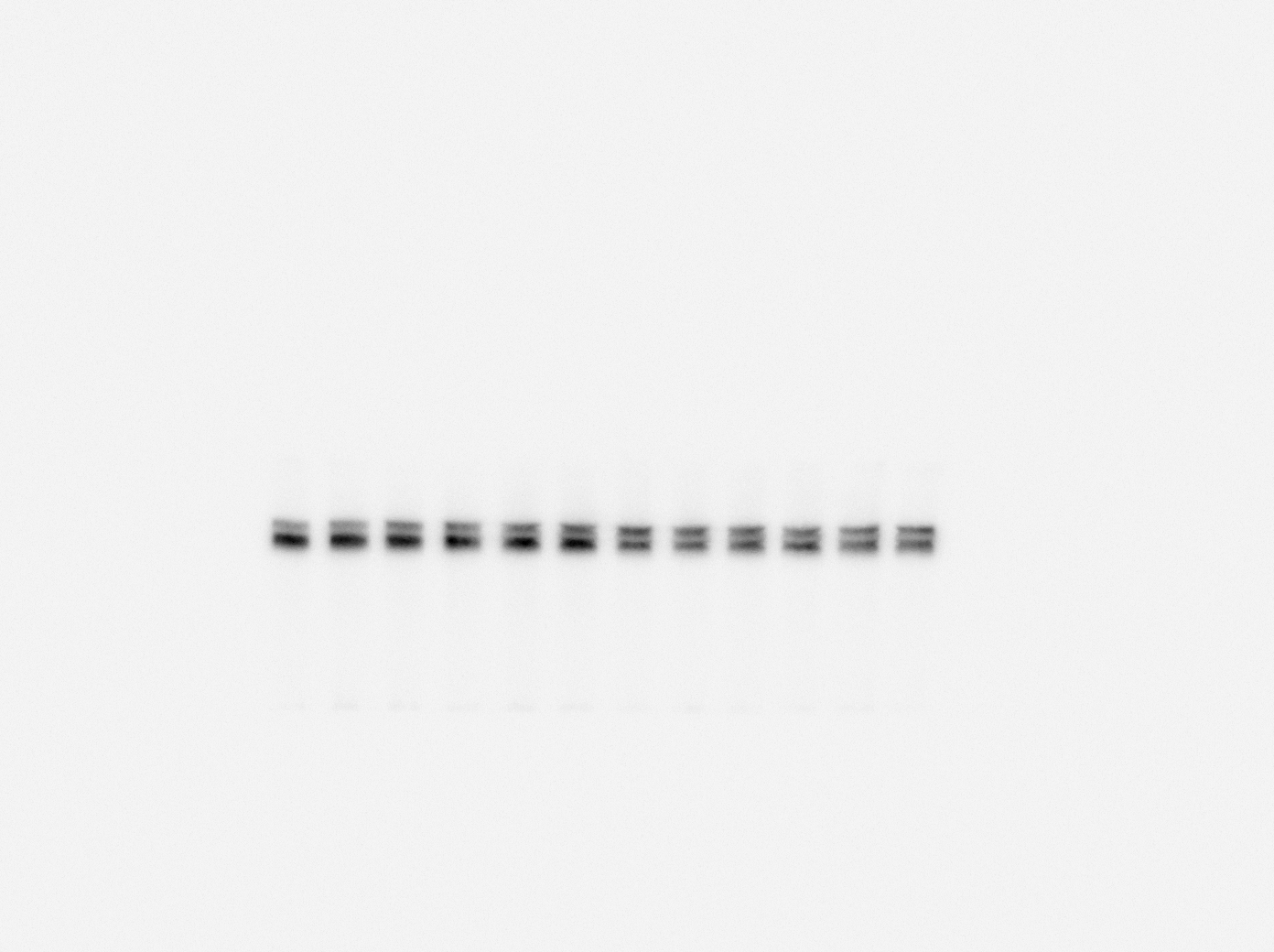

Supplement: Supplementary file 1 [file cancers-13-00862-s001.zip › WBdata_cancers/20200301_8505_E7080U0126_E2F1/20200301_8505_E7080U0126_E2F1_e.tif]

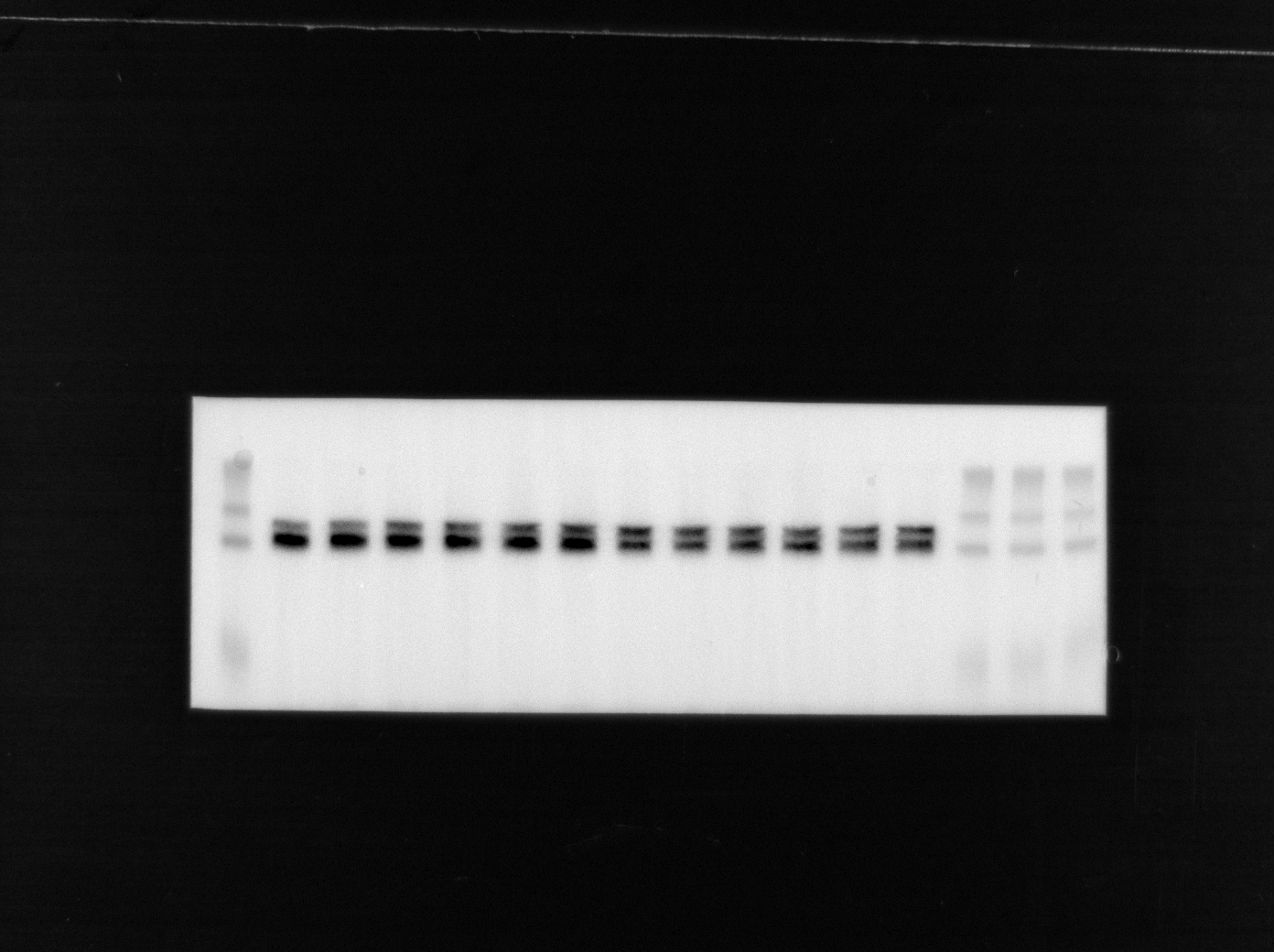

Supplement: Supplementary file 1 [file cancers-13-00862-s001.zip › WBdata_cancers/20200301_8505_E7080U0126_E2F1/20200301_8505_E7080U0126_E2F1_Merge.tif]

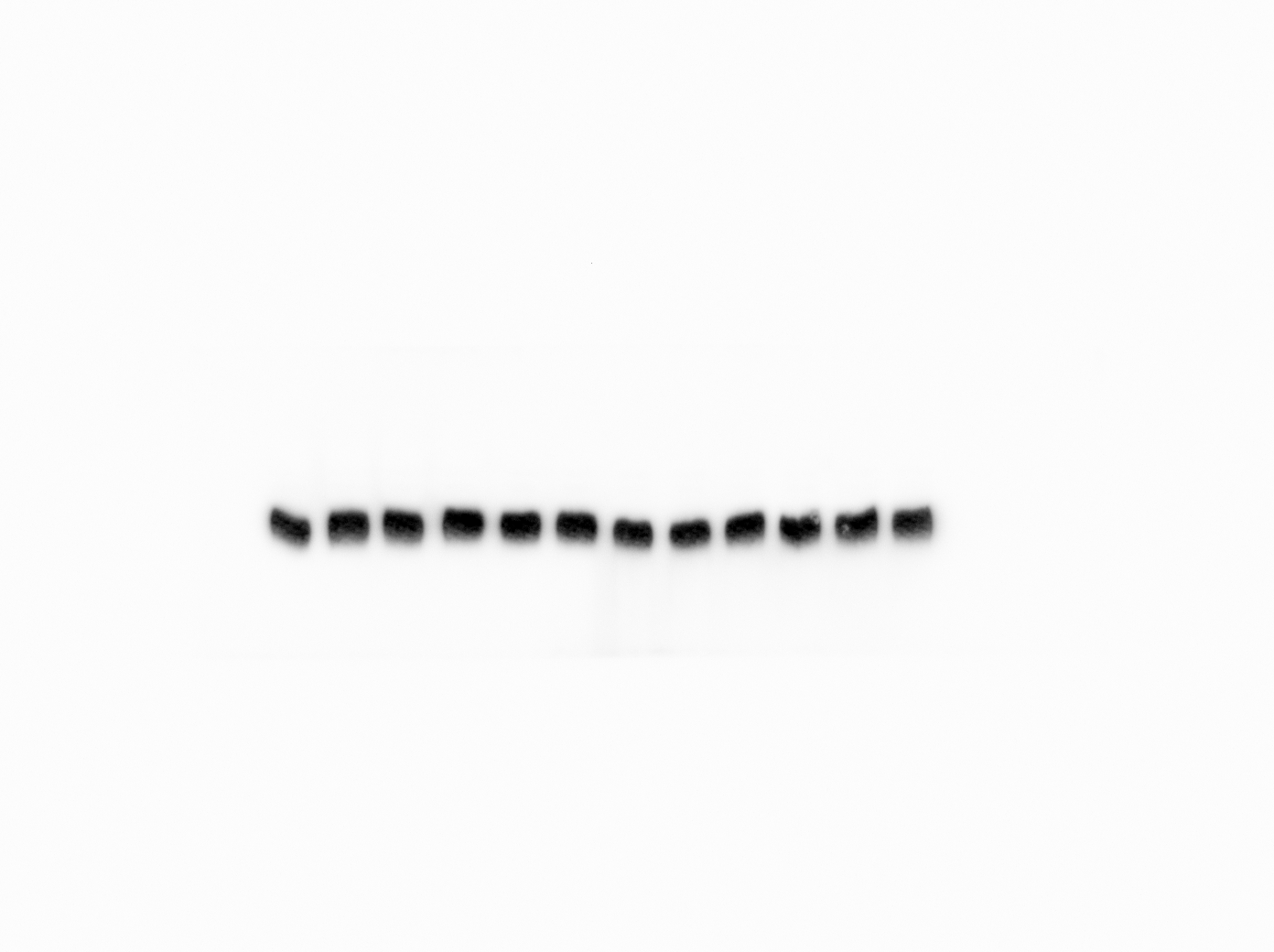

Supplement: Supplementary file 1 [file cancers-13-00862-s001.zip › WBdata_cancers/20200302_8505_E7080U0126_aTub/20200302_8505_E7080U0126_aTub_a.tif]

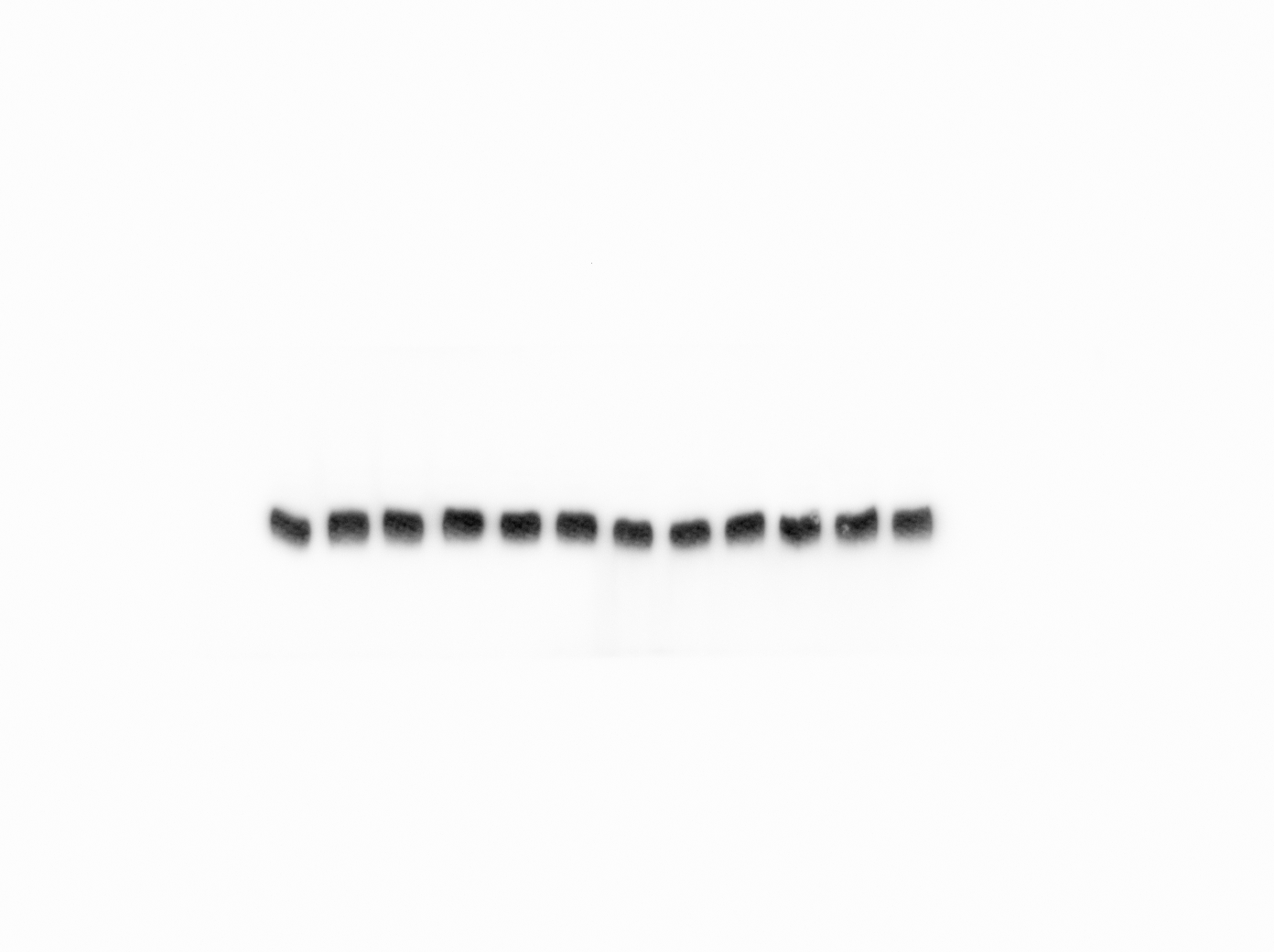

Supplement: Supplementary file 1 [file cancers-13-00862-s001.zip › WBdata_cancers/20200302_8505_E7080U0126_aTub/20200302_8505_E7080U0126_aTub_b.tif]

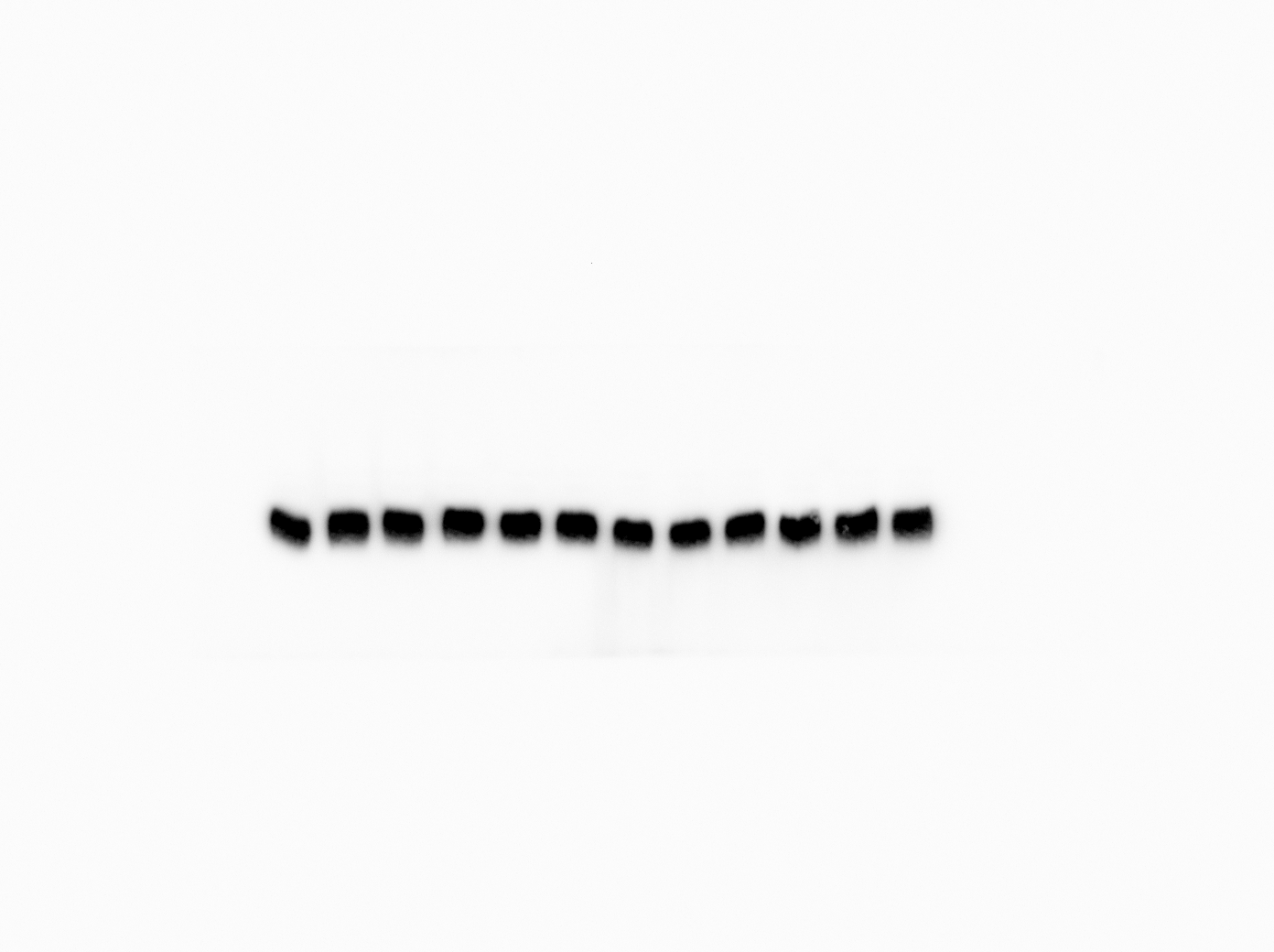

Supplement: Supplementary file 1 [file cancers-13-00862-s001.zip › WBdata_cancers/20200302_8505_E7080U0126_aTub/20200302_8505_E7080U0126_aTub_c.tif]

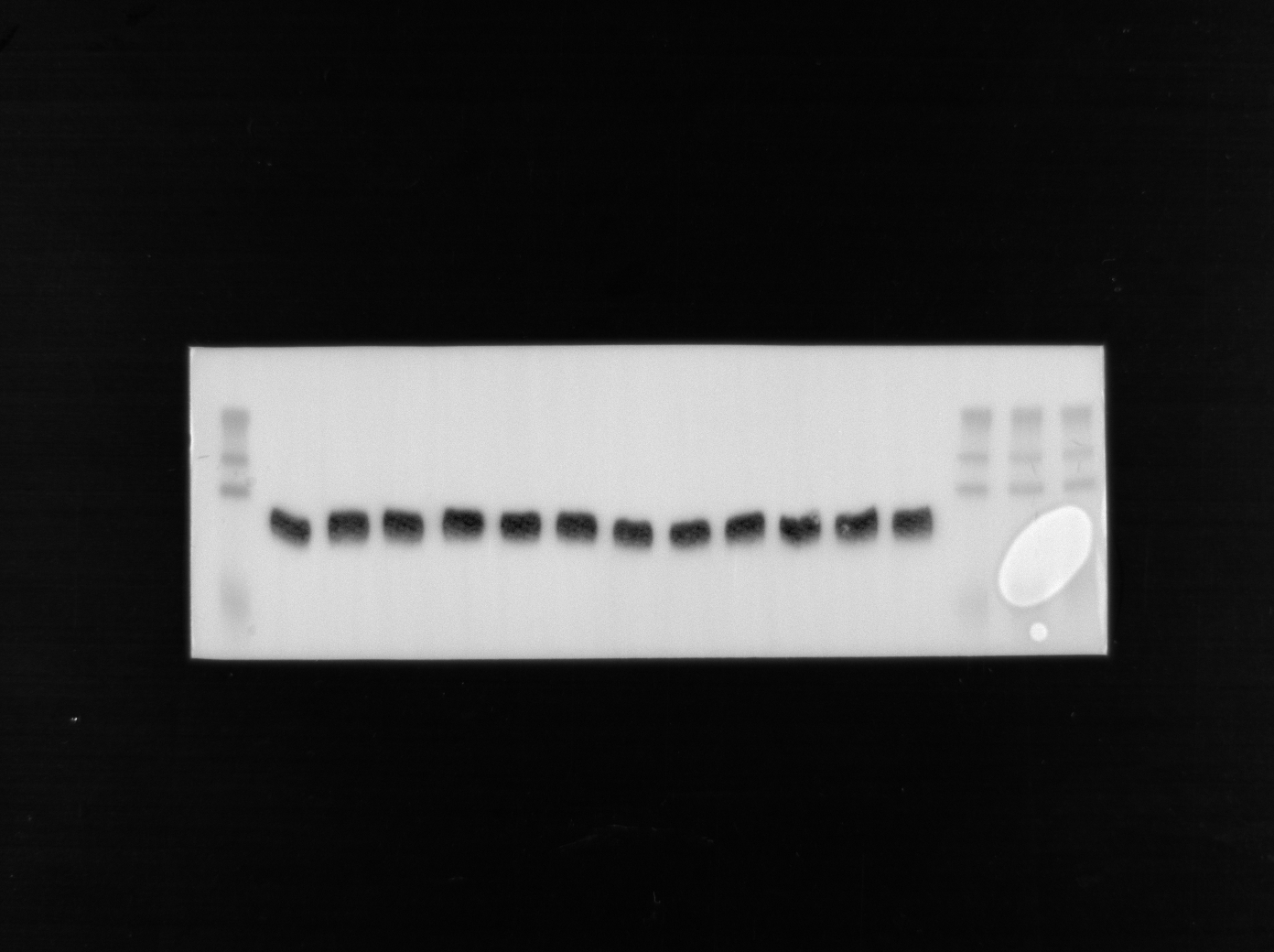

Supplement: Supplementary file 1 [file cancers-13-00862-s001.zip › WBdata_cancers/20200302_8505_E7080U0126_aTub/20200302_8505_E7080U0126_aTub_Merge.tif]

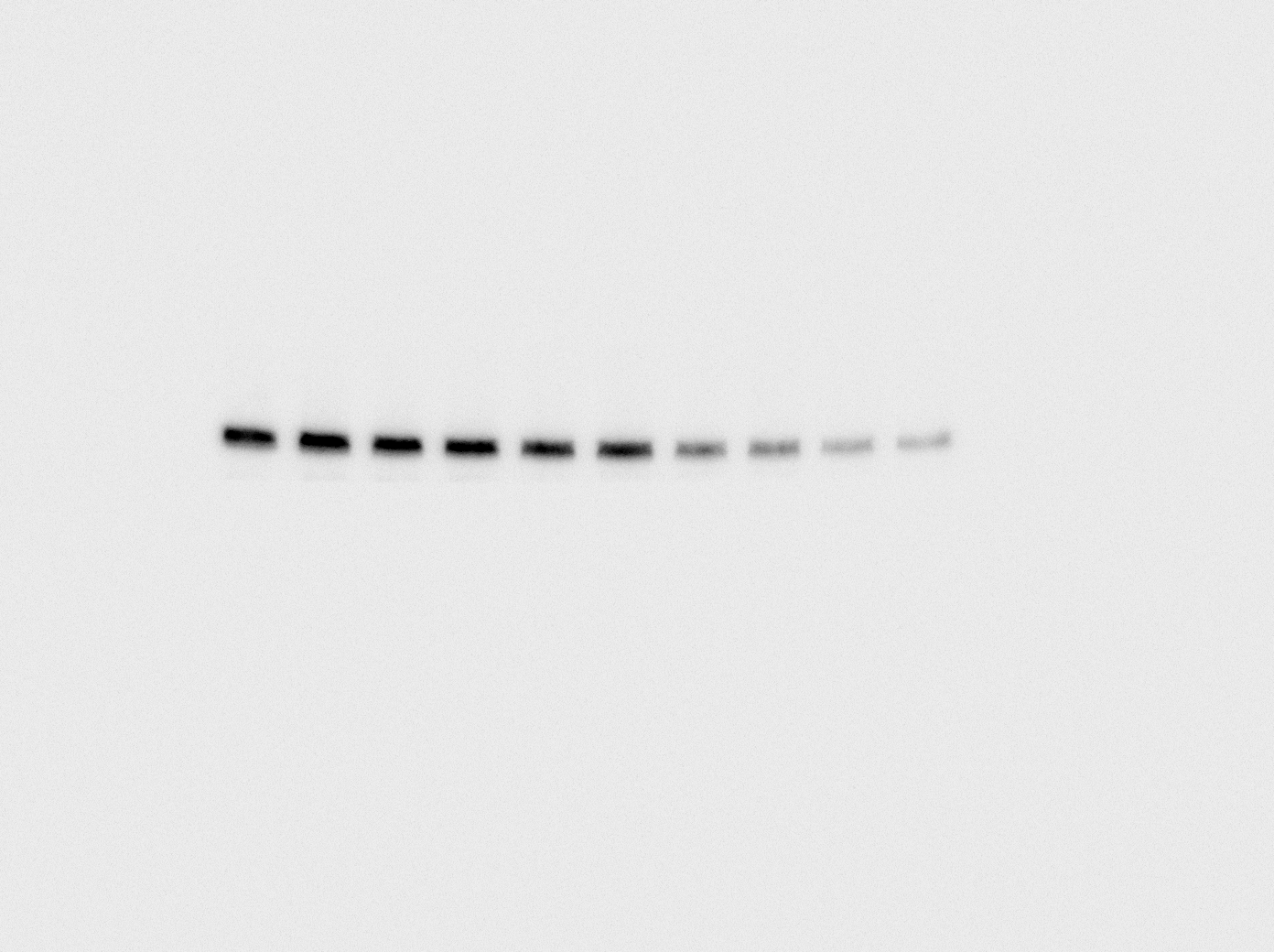

Supplement: Supplementary file 1 [file cancers-13-00862-s001.zip › WBdata_cancers/20200401_8505_E7080_pAKT/20200401_8505_E7080_pAKT_a.tif]

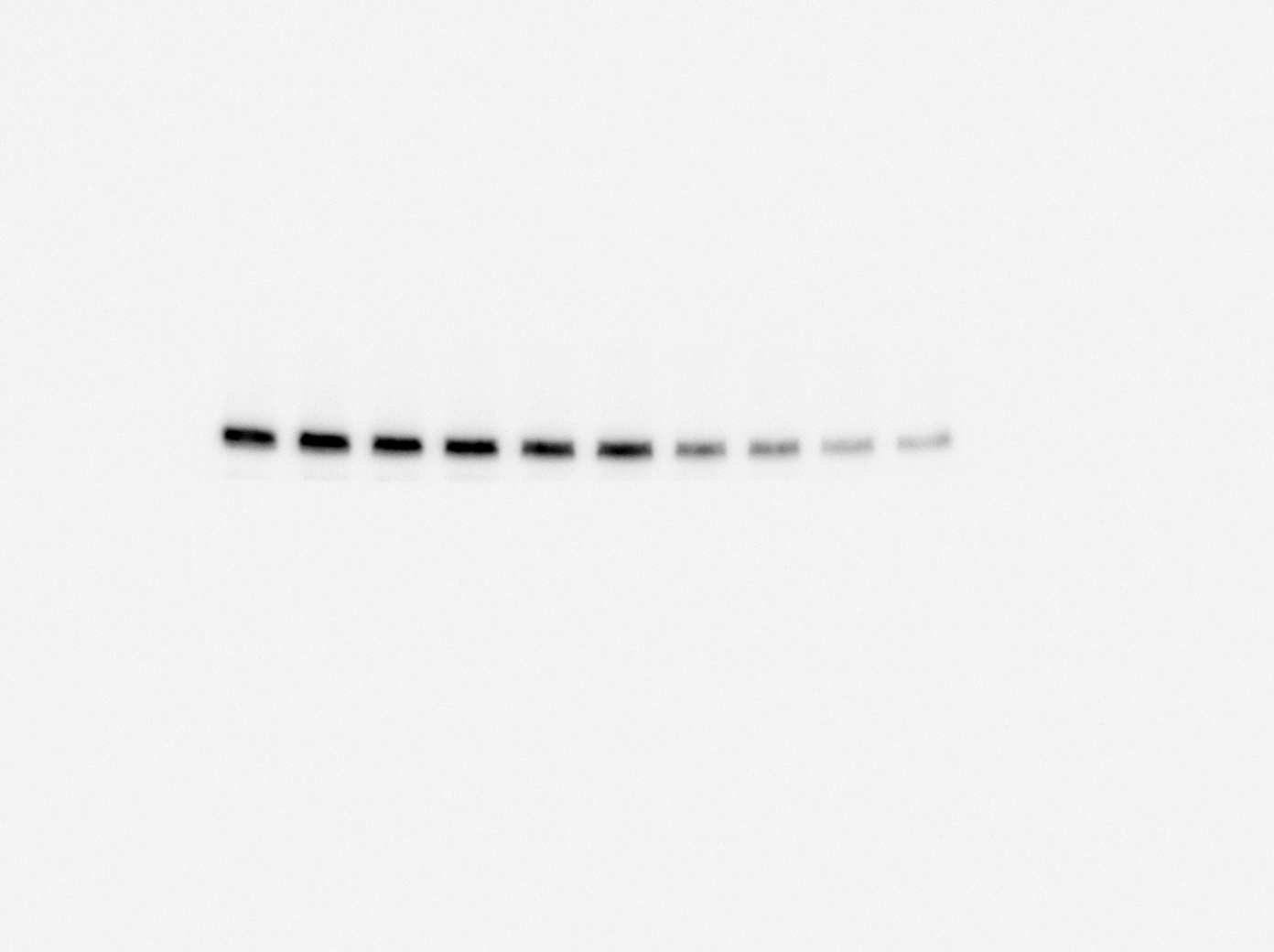

Supplement: Supplementary file 1 [file cancers-13-00862-s001.zip › WBdata_cancers/20200401_8505_E7080_pAKT/20200401_8505_E7080_pAKT_b.tif]

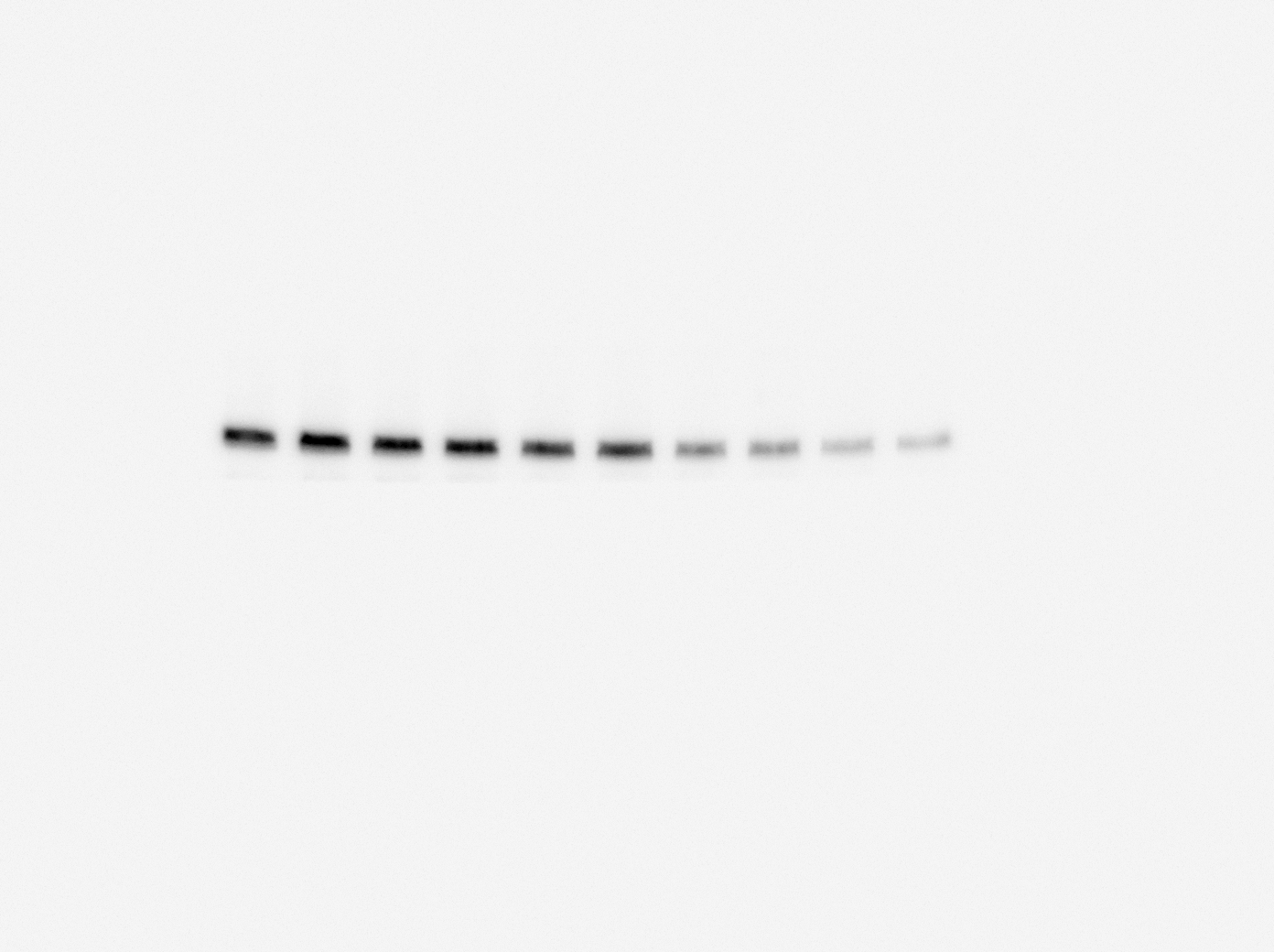

Supplement: Supplementary file 1 [file cancers-13-00862-s001.zip › WBdata_cancers/20200401_8505_E7080_pAKT/20200401_8505_E7080_pAKT_c.tif]

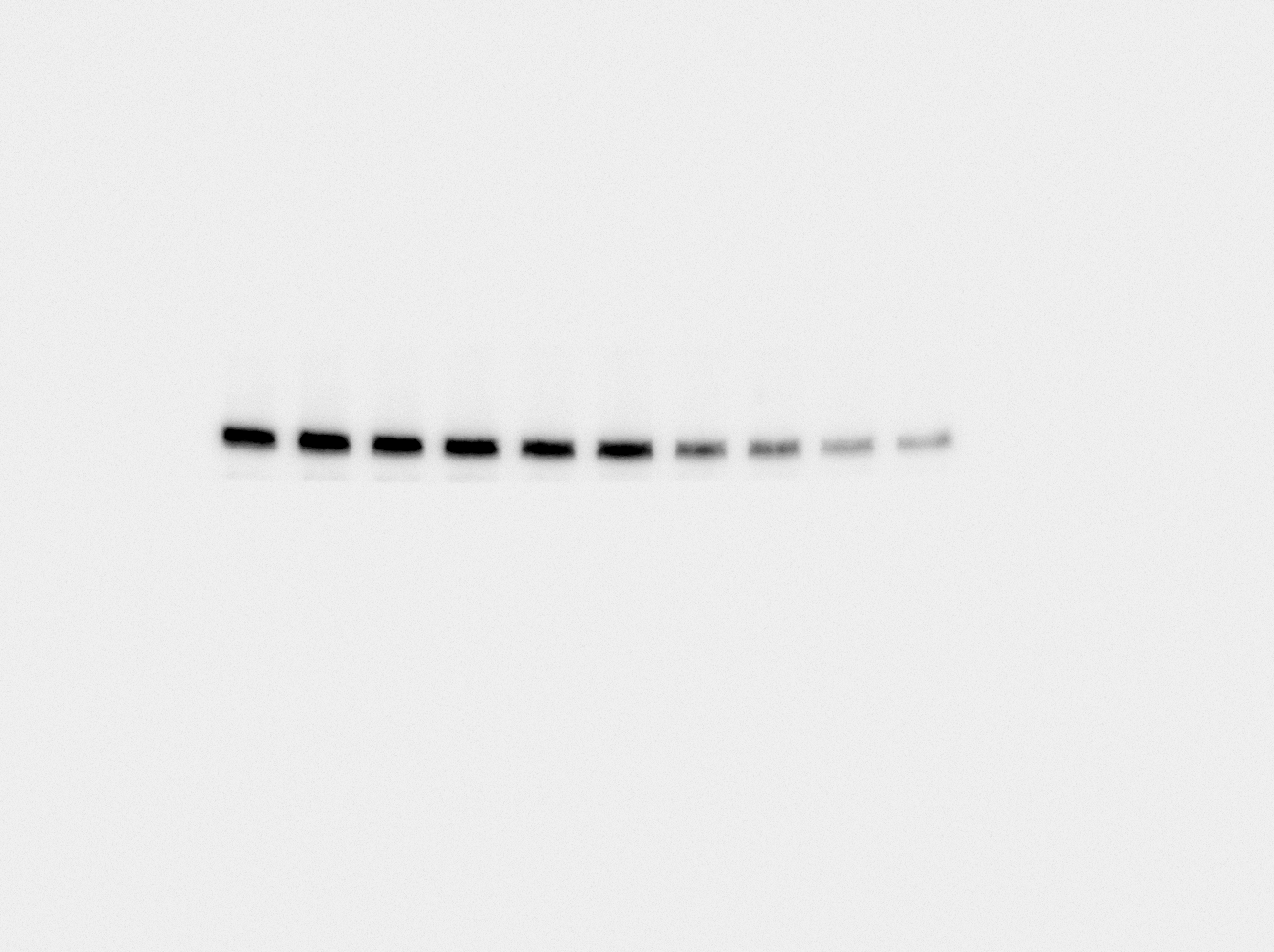

Supplement: Supplementary file 1 [file cancers-13-00862-s001.zip › WBdata_cancers/20200401_8505_E7080_pAKT/20200401_8505_E7080_pAKT_d.tif]

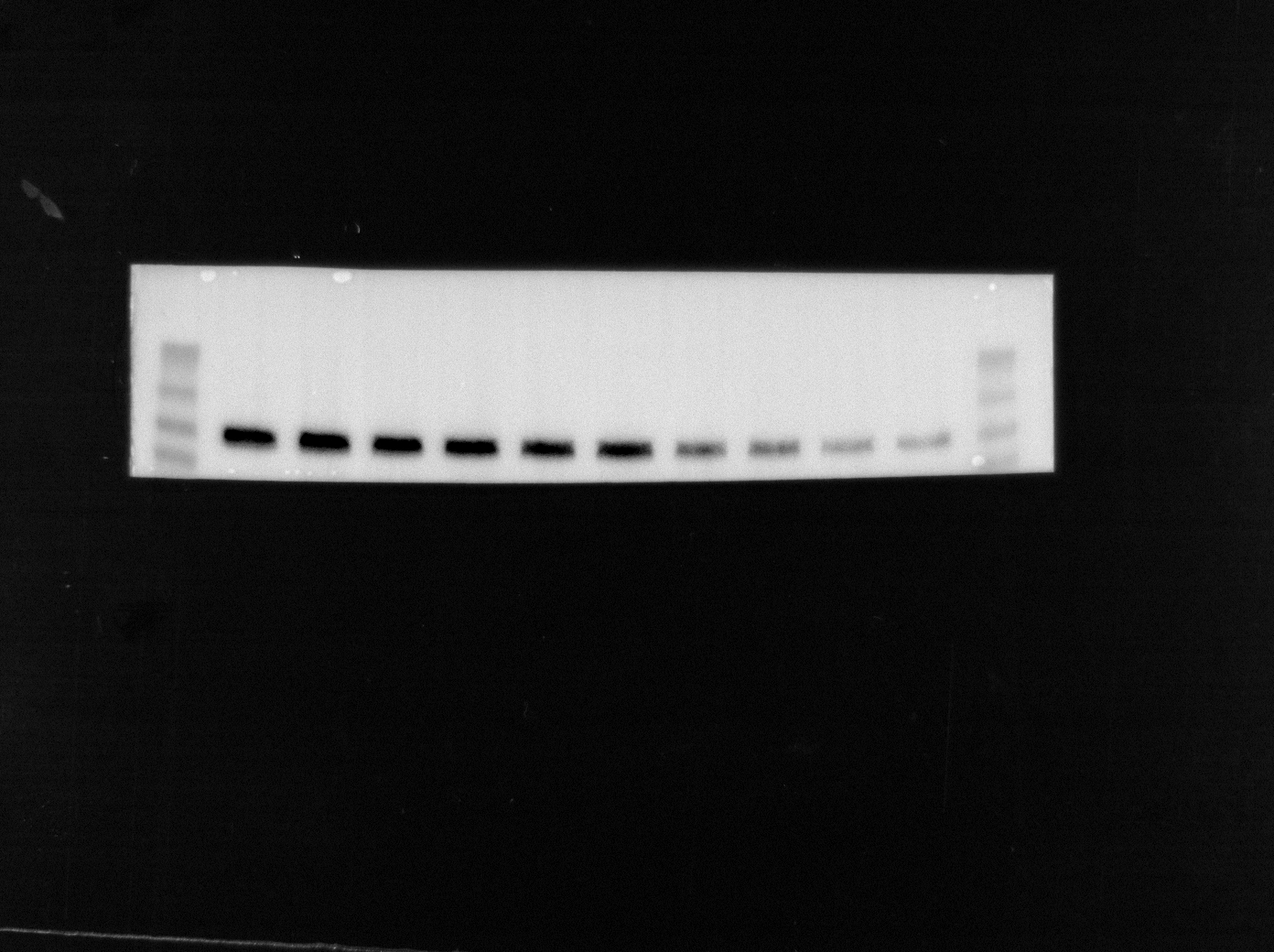

Supplement: Supplementary file 1 [file cancers-13-00862-s001.zip › WBdata_cancers/20200401_8505_E7080_pAKT/20200401_8505_E7080_pAKT_Merge.tif]

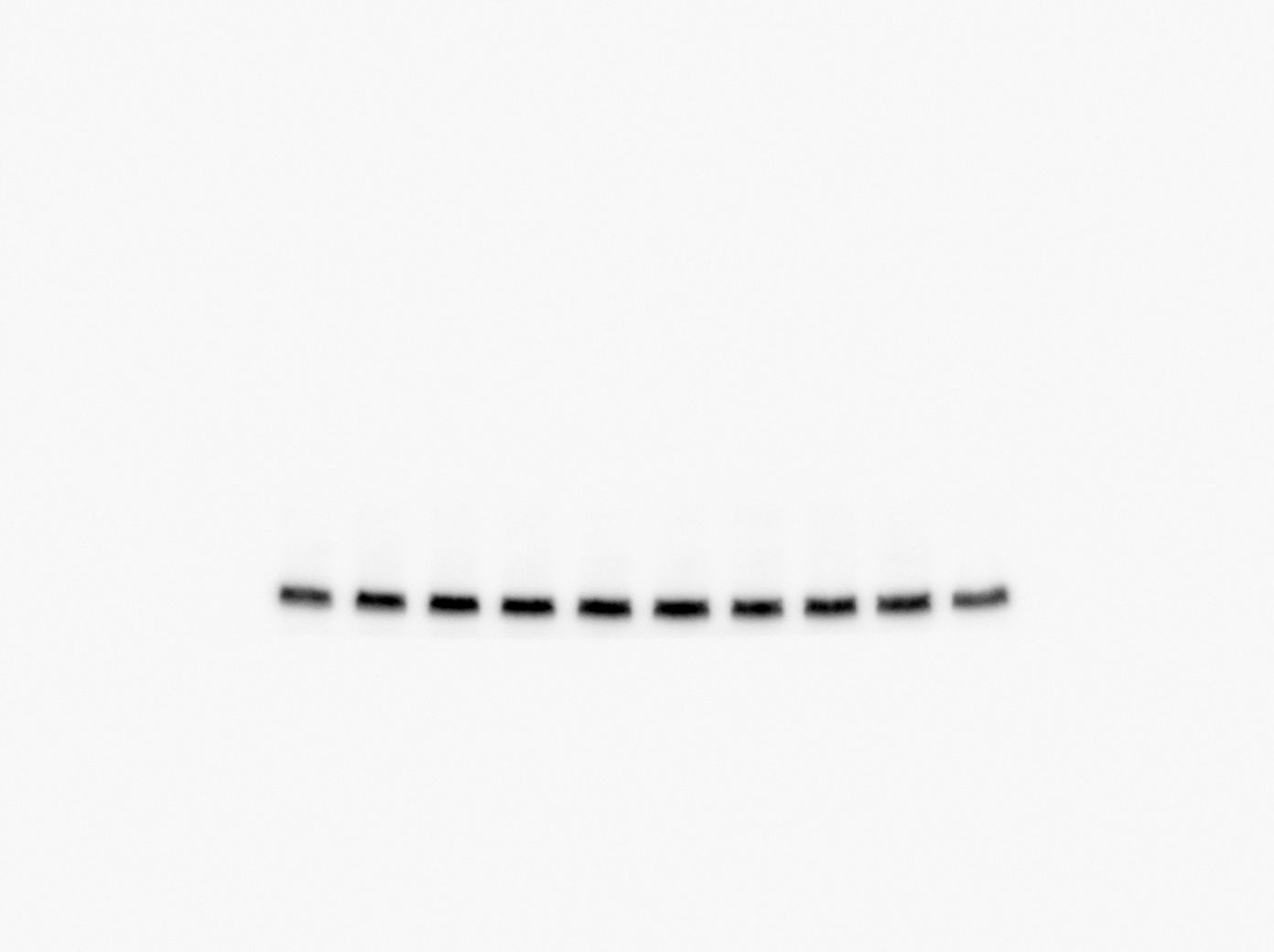

Supplement: Supplementary file 1 [file cancers-13-00862-s001.zip › WBdata_cancers/20200402_8505_E7080_tAKT/20200402_8505_E7080_tAKT_a.tif]

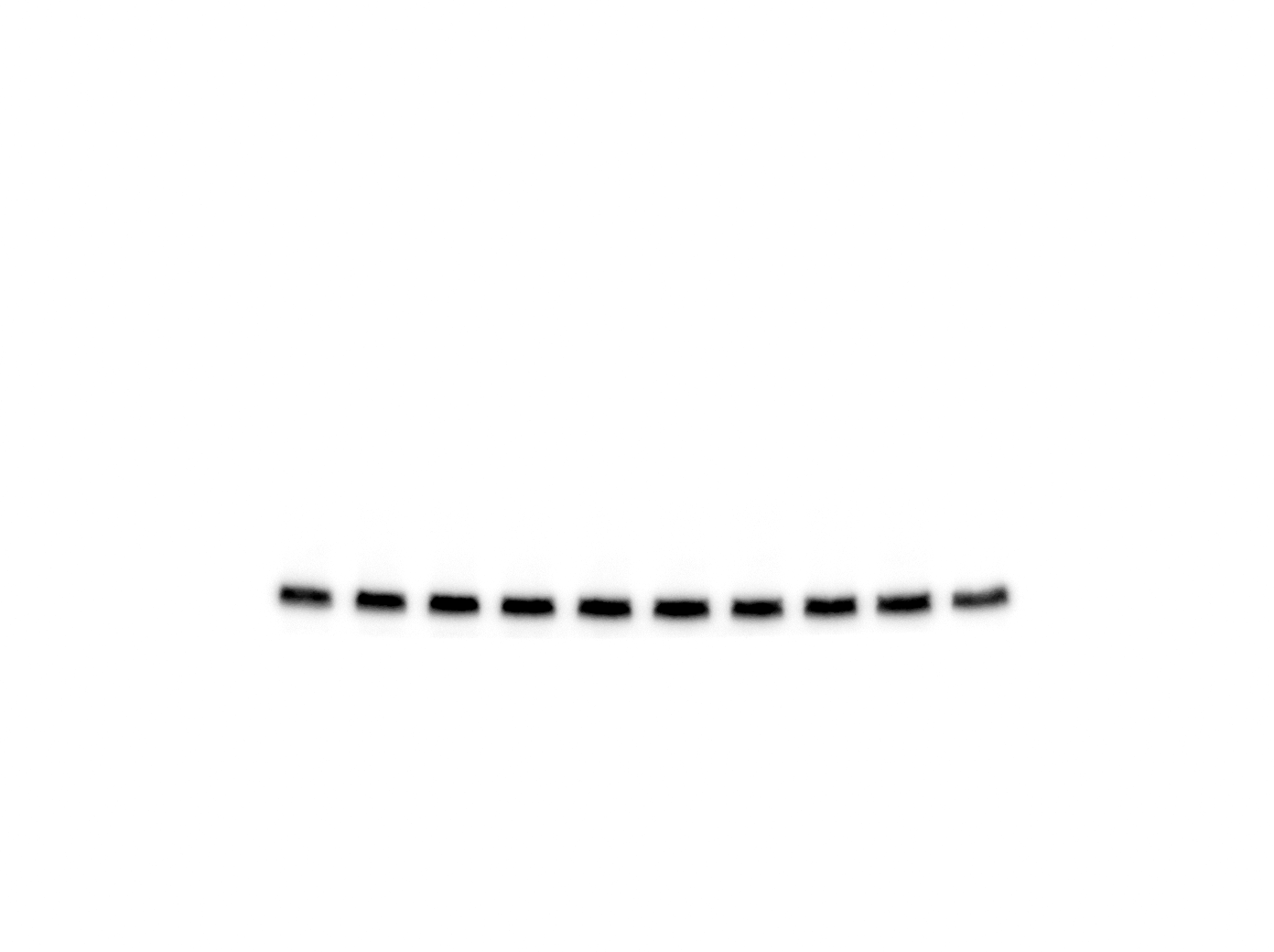

Supplement: Supplementary file 1 [file cancers-13-00862-s001.zip › WBdata_cancers/20200402_8505_E7080_tAKT/20200402_8505_E7080_tAKT_b.tif]

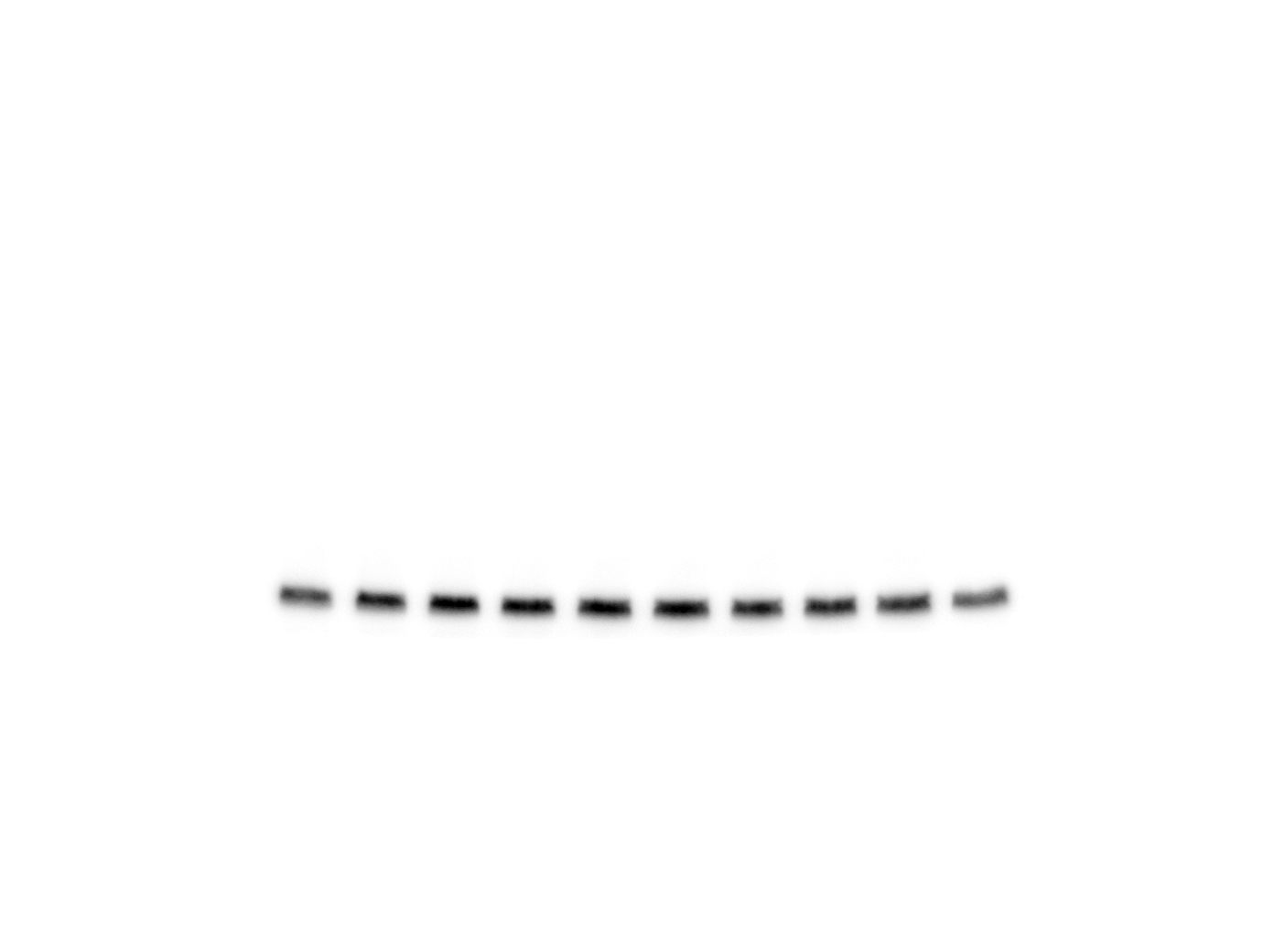

Supplement: Supplementary file 1 [file cancers-13-00862-s001.zip › WBdata_cancers/20200402_8505_E7080_tAKT/20200402_8505_E7080_tAKT_c.tif]

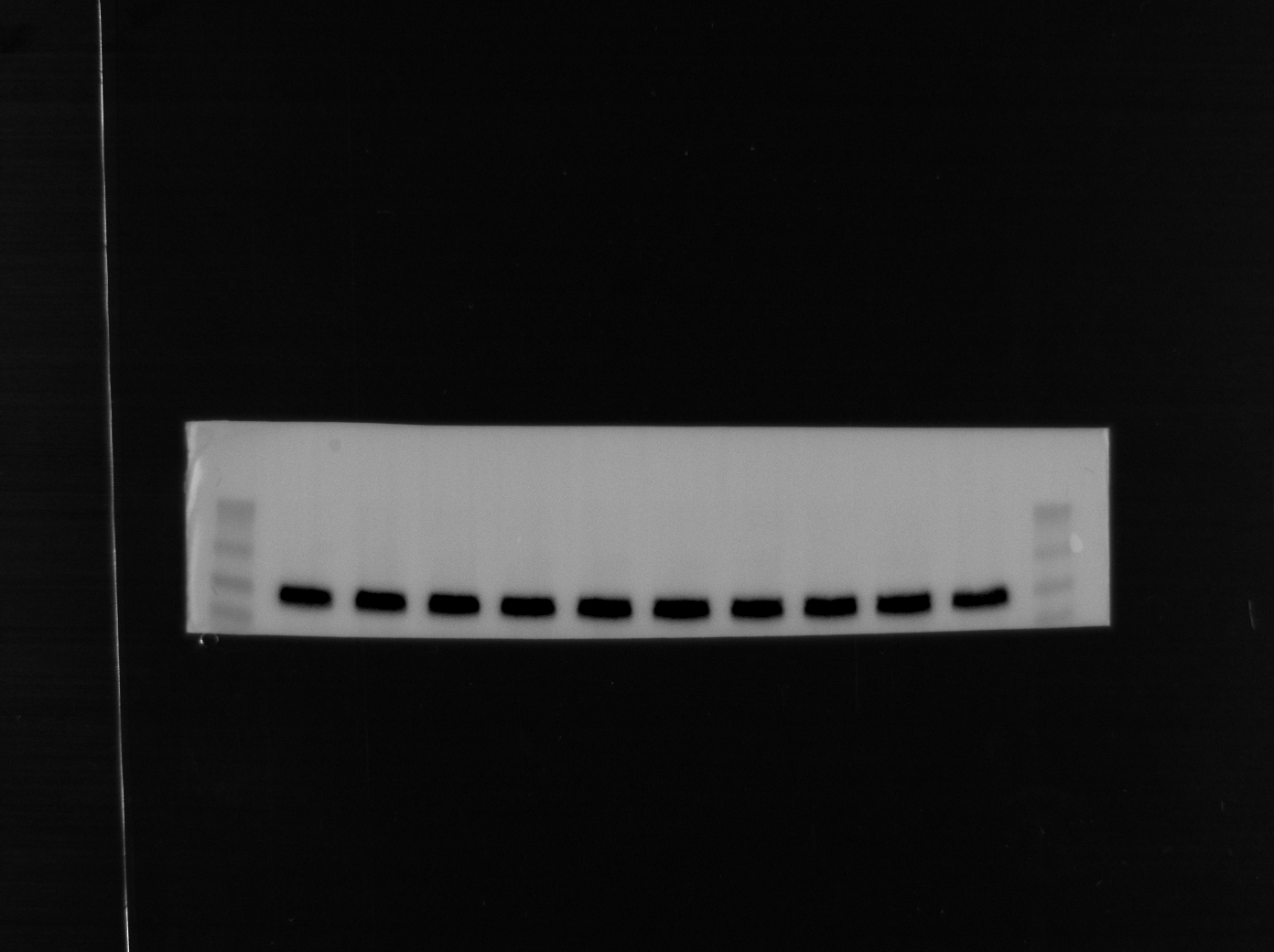

Supplement: Supplementary file 1 [file cancers-13-00862-s001.zip › WBdata_cancers/20200402_8505_E7080_tAKT/20200402_8505_E7080_tAKT_Merge.tif]

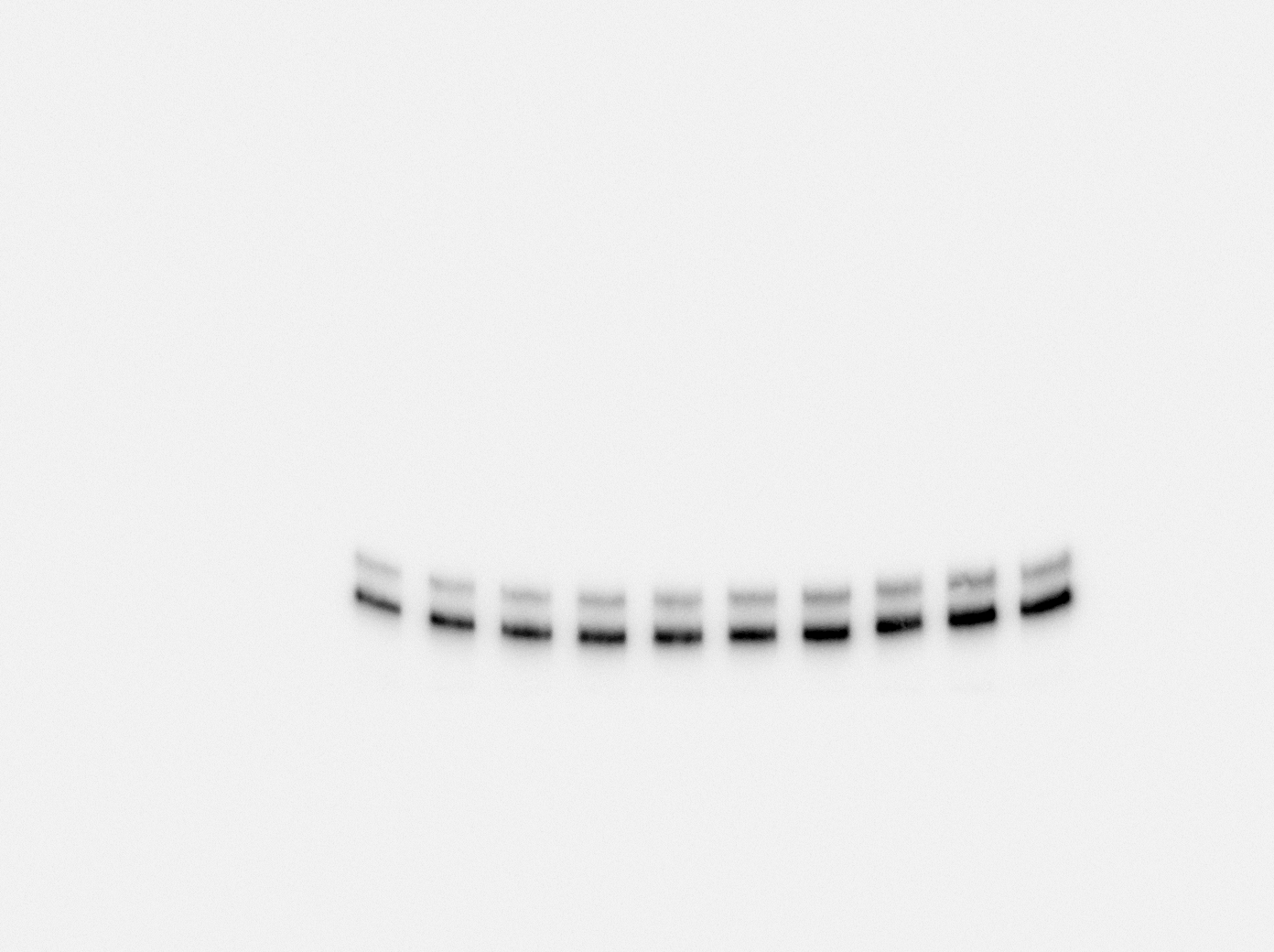

Supplement: Supplementary file 1 [file cancers-13-00862-s001.zip › WBdata_cancers/20200407_8505_E7080_pERK/20200407_8505_E7080_pERK_a.tif]

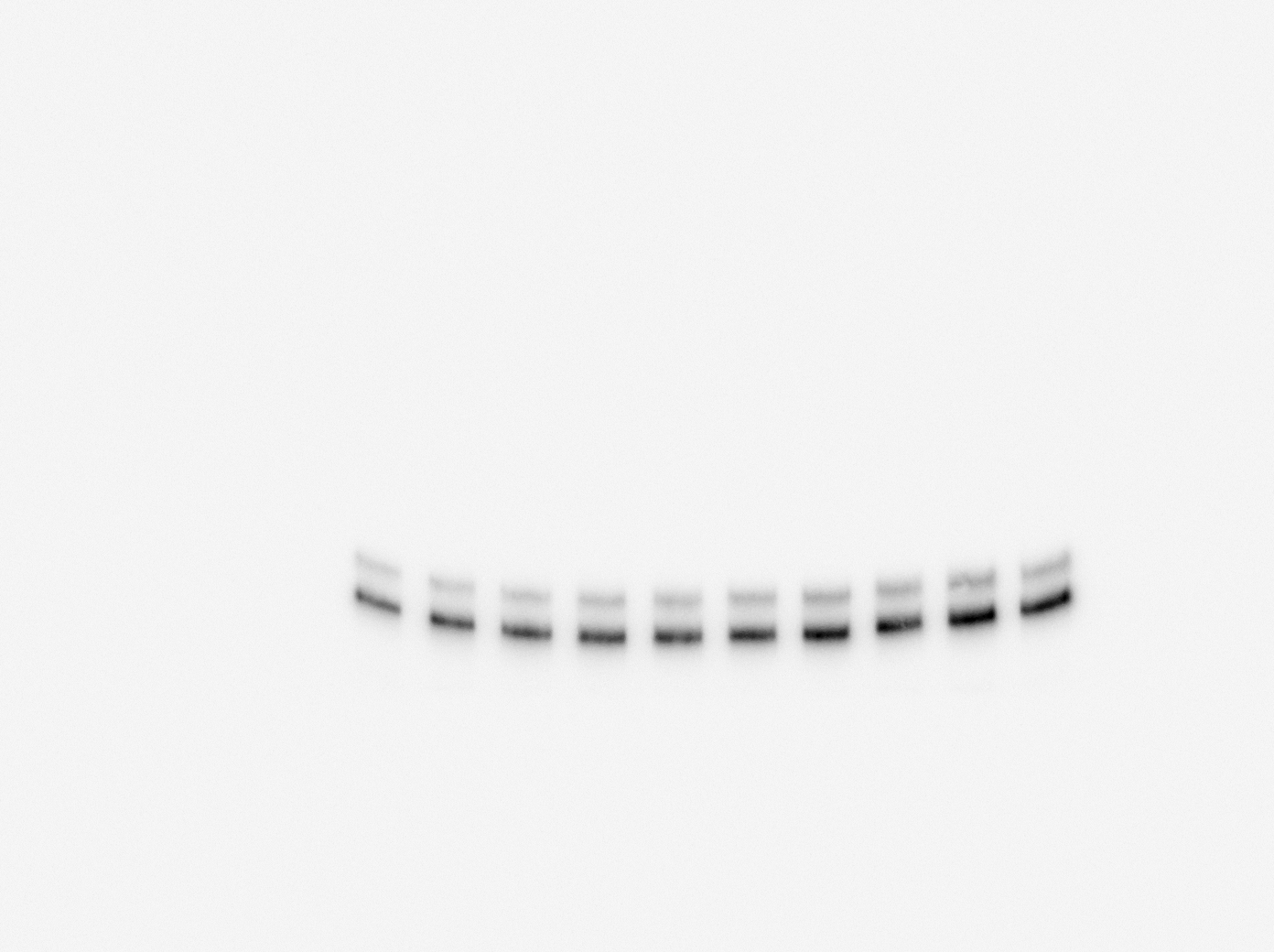

Supplement: Supplementary file 1 [file cancers-13-00862-s001.zip › WBdata_cancers/20200407_8505_E7080_pERK/20200407_8505_E7080_pERK_b.tif]

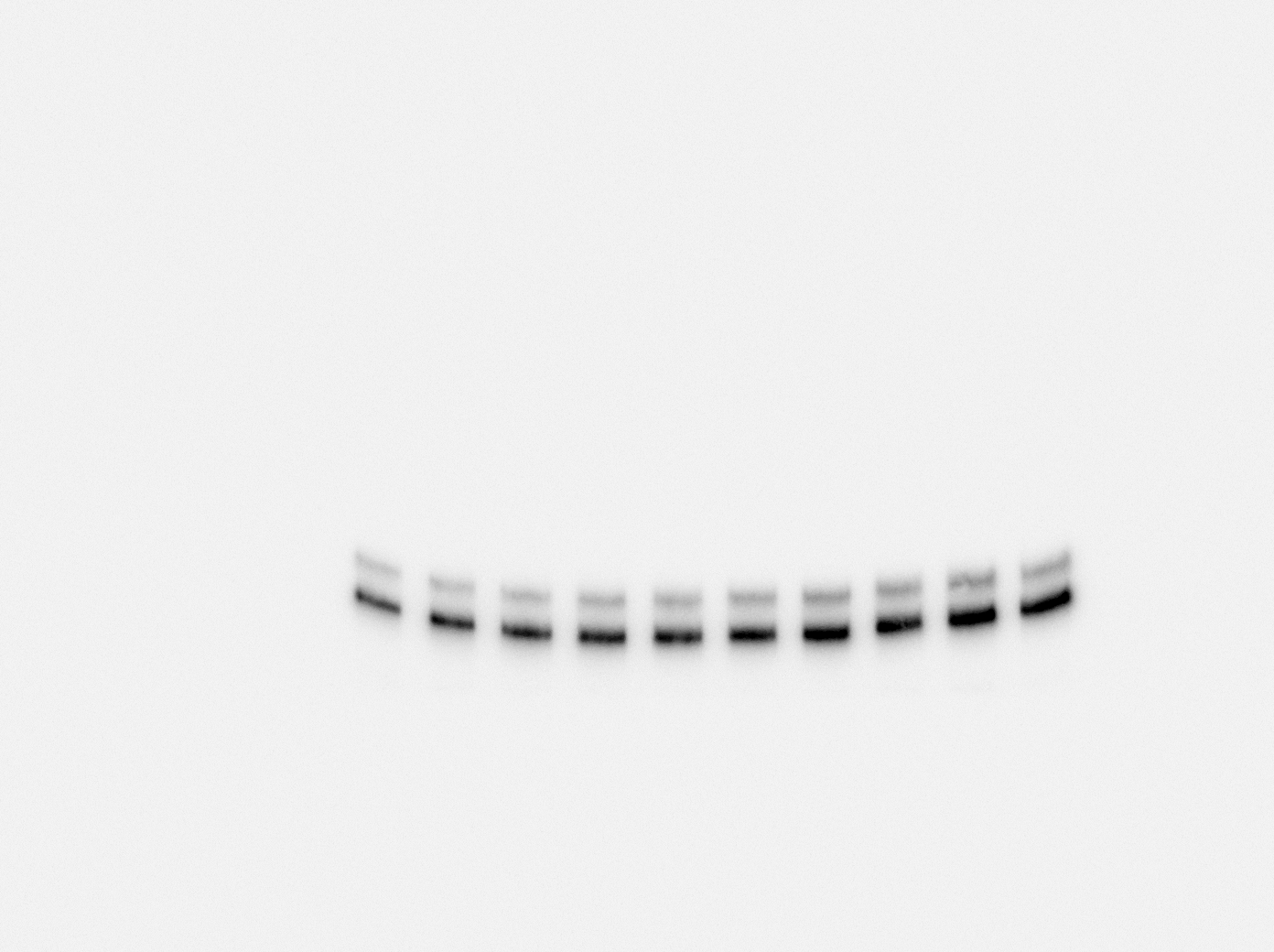

Supplement: Supplementary file 1 [file cancers-13-00862-s001.zip › WBdata_cancers/20200407_8505_E7080_pERK/20200407_8505_E7080_pERK_c.tif]

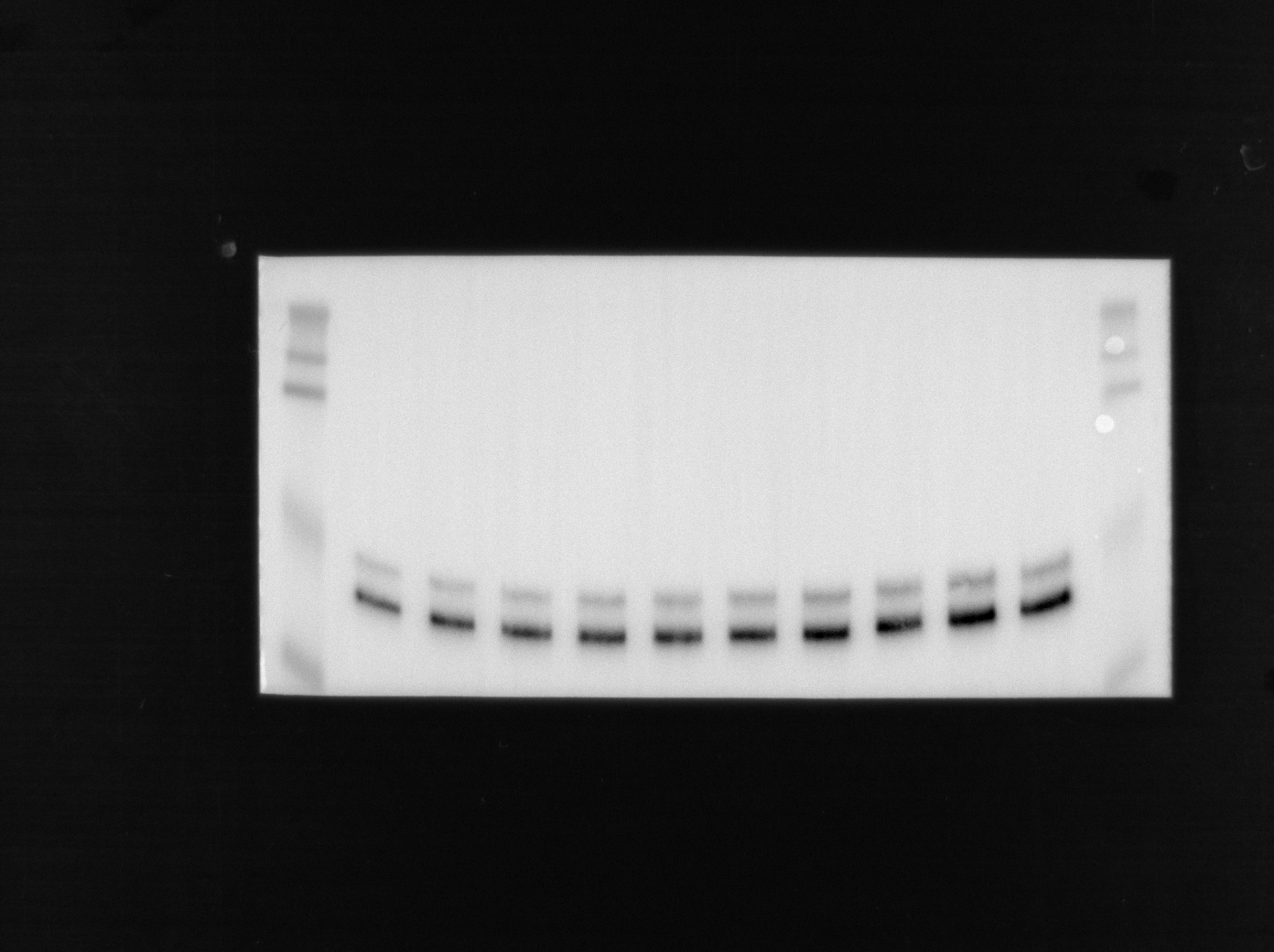

Supplement: Supplementary file 1 [file cancers-13-00862-s001.zip › WBdata_cancers/20200407_8505_E7080_pERK/20200407_8505_E7080_pERK_Merge.tif]

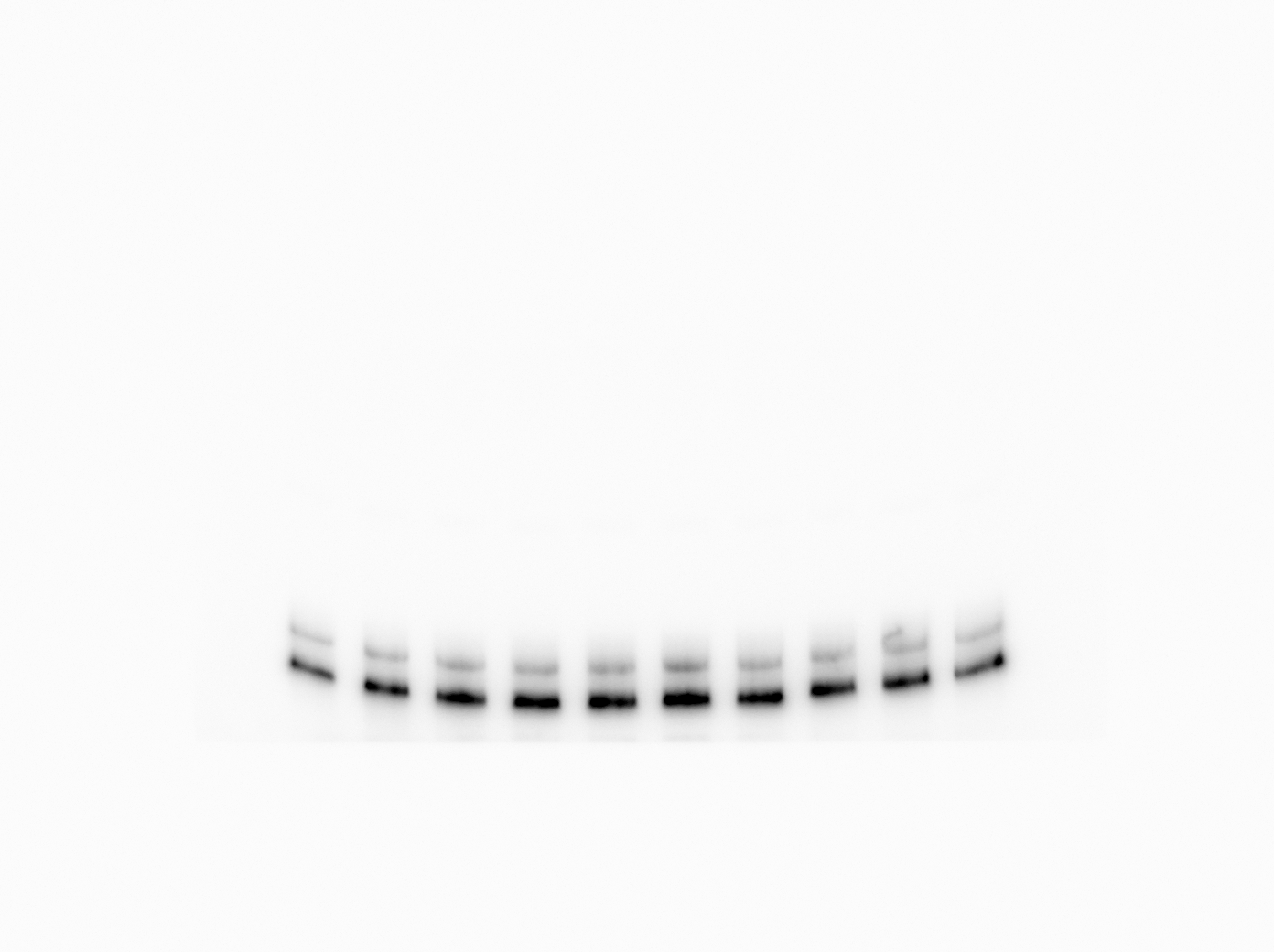

Supplement: Supplementary file 1 [file cancers-13-00862-s001.zip › WBdata_cancers/20200409_8505_E7080_tERK/20200409_8505_E7080_tERK_a.tif]

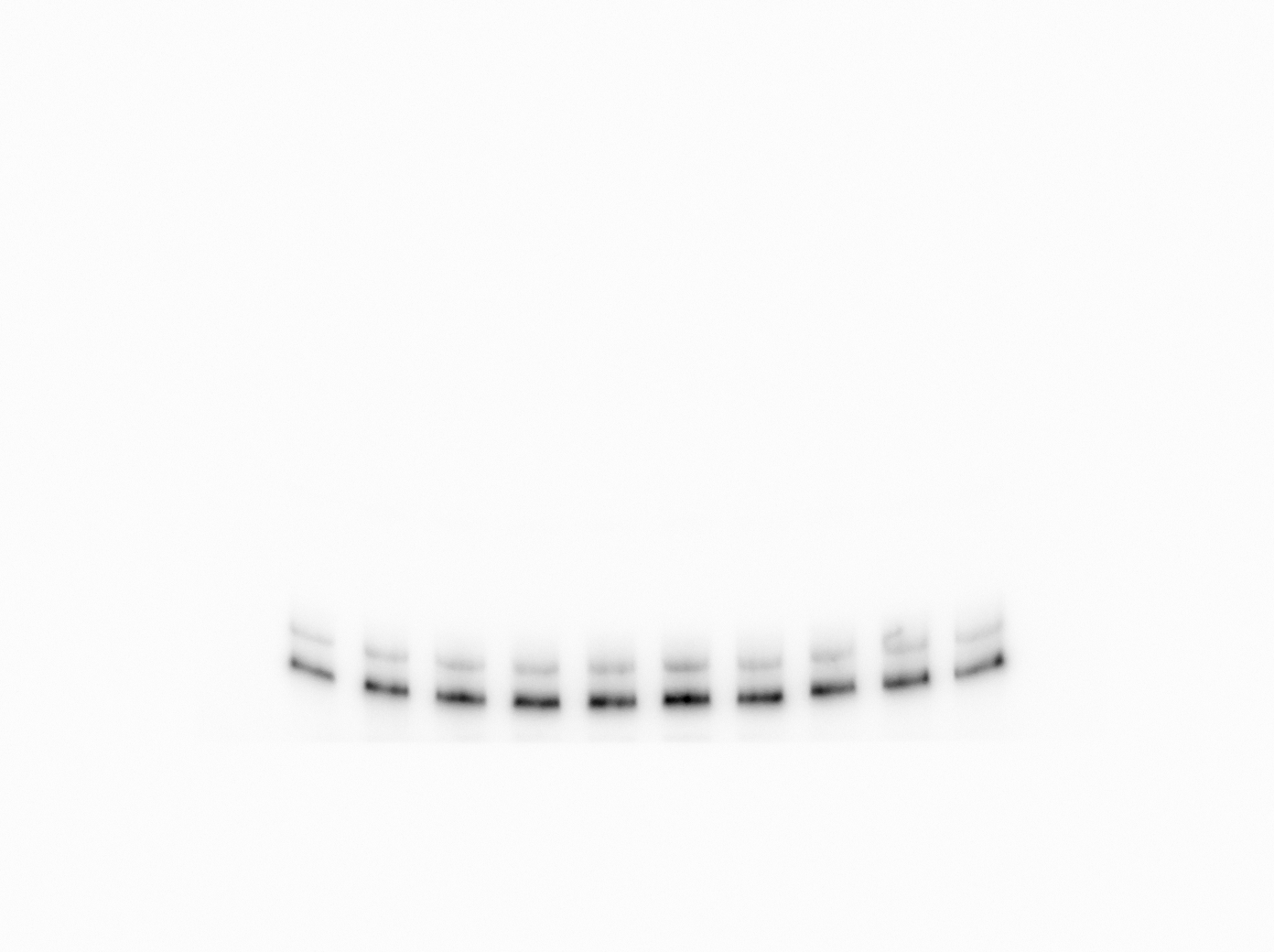

Supplement: Supplementary file 1 [file cancers-13-00862-s001.zip › WBdata_cancers/20200409_8505_E7080_tERK/20200409_8505_E7080_tERK_b.tif]

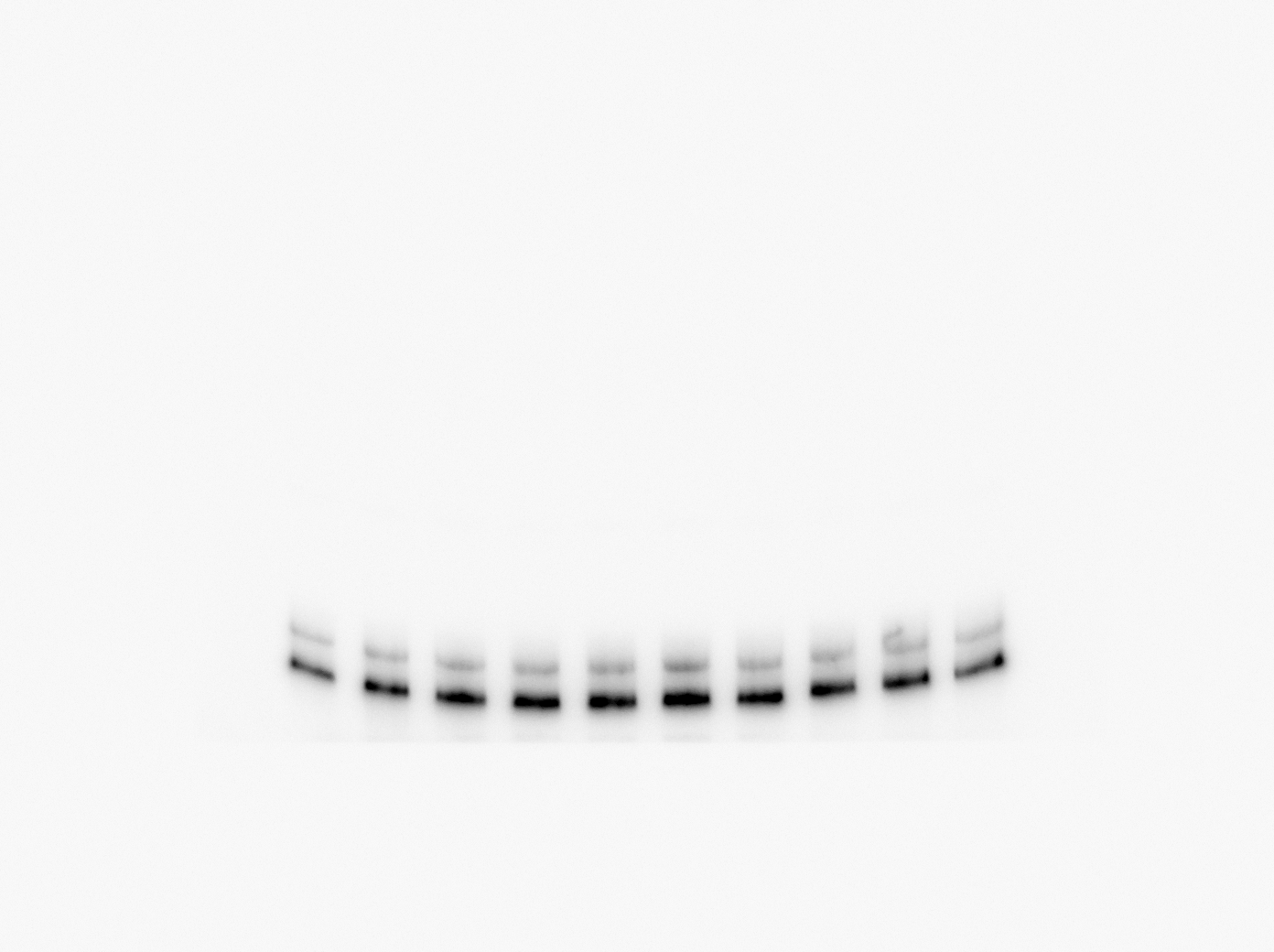

Supplement: Supplementary file 1 [file cancers-13-00862-s001.zip › WBdata_cancers/20200409_8505_E7080_tERK/20200409_8505_E7080_tERK_c.tif]

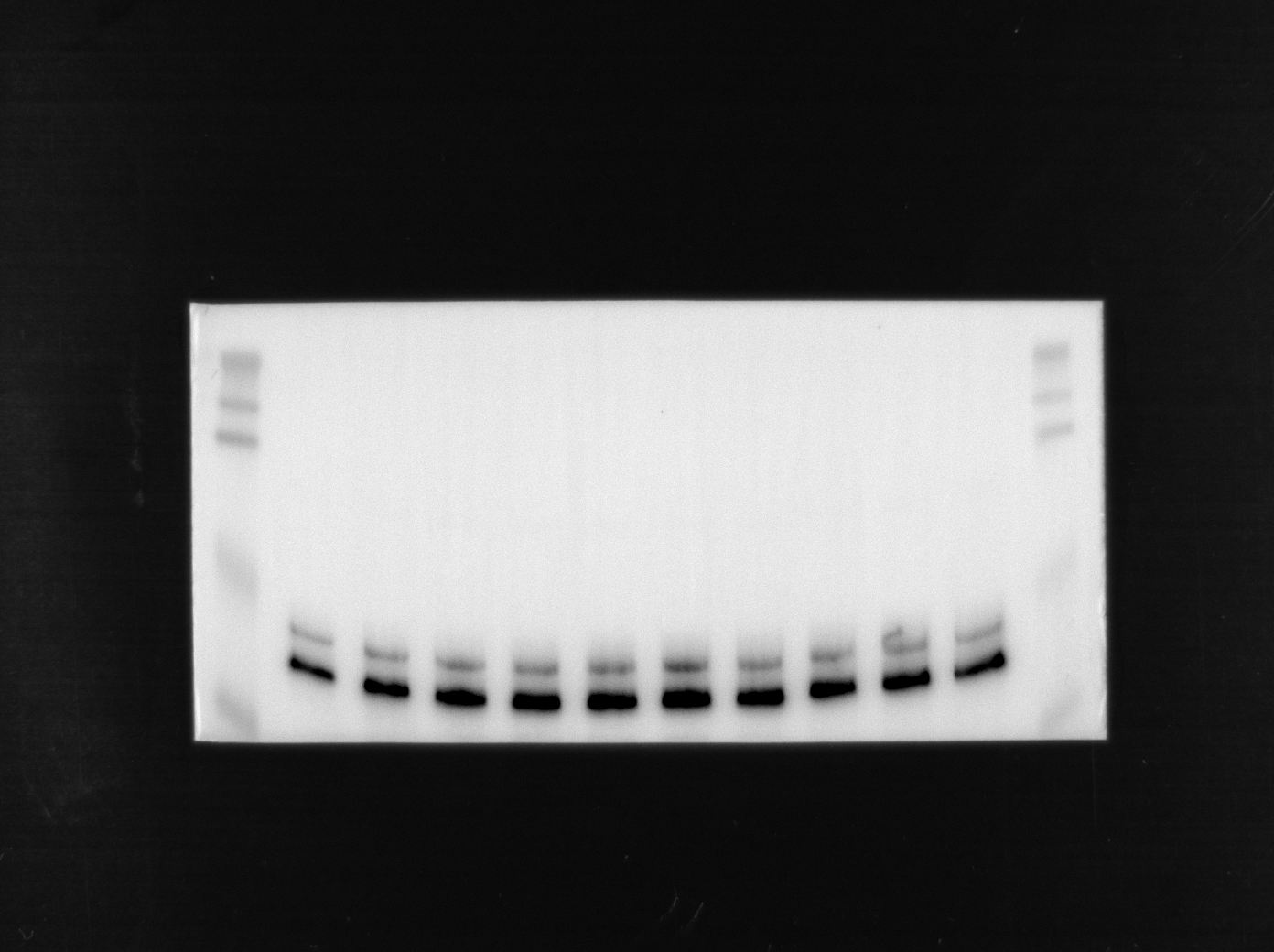

Supplement: Supplementary file 1 [file cancers-13-00862-s001.zip › WBdata_cancers/20200409_8505_E7080_tERK/20200409_8505_E7080_tERK_Merge.tif]

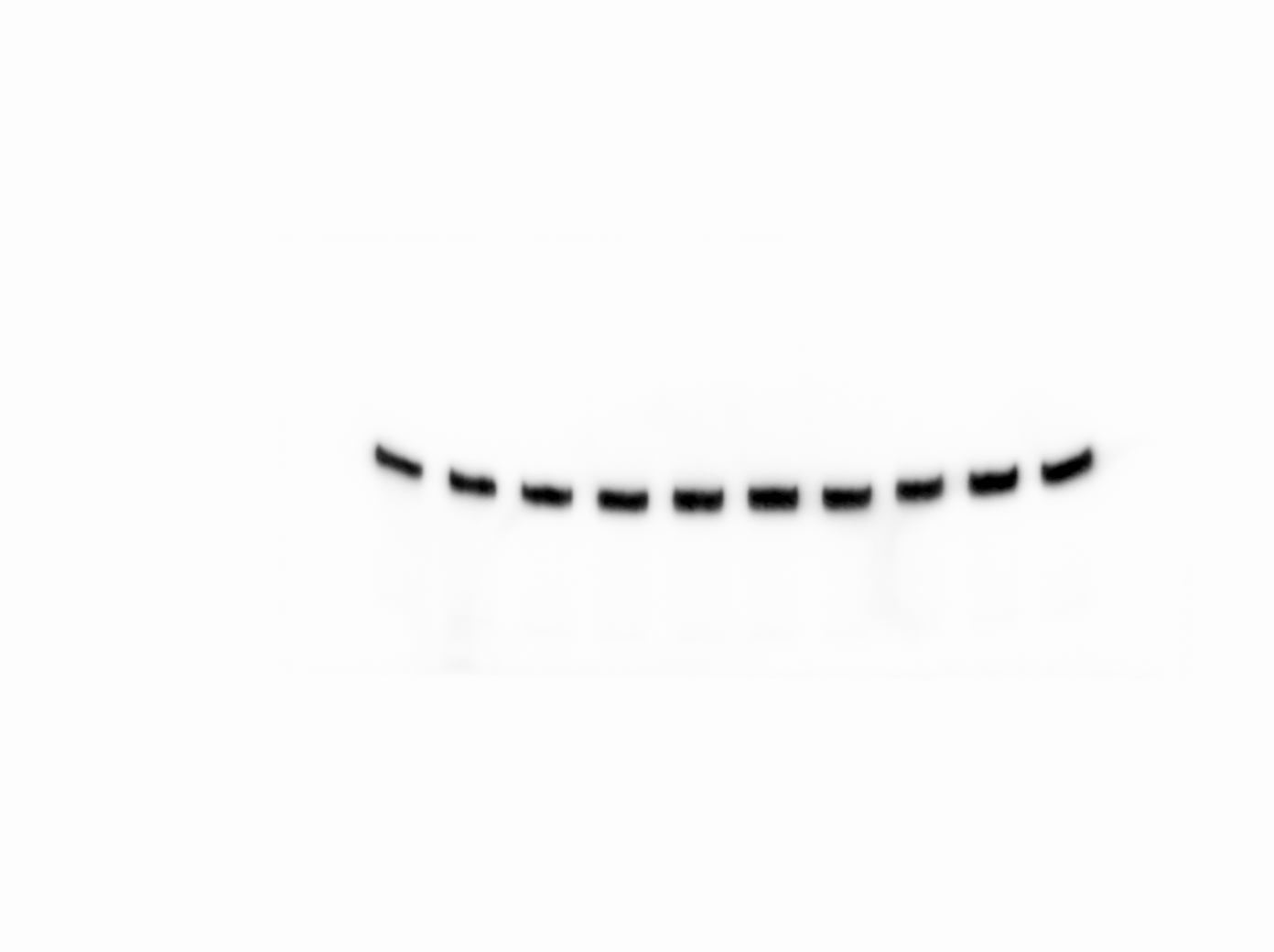

Supplement: Supplementary file 1 [file cancers-13-00862-s001.zip › WBdata_cancers/20200410_8505_E7080_aTub/20200410_8505_E7080_aTub_a.tif]

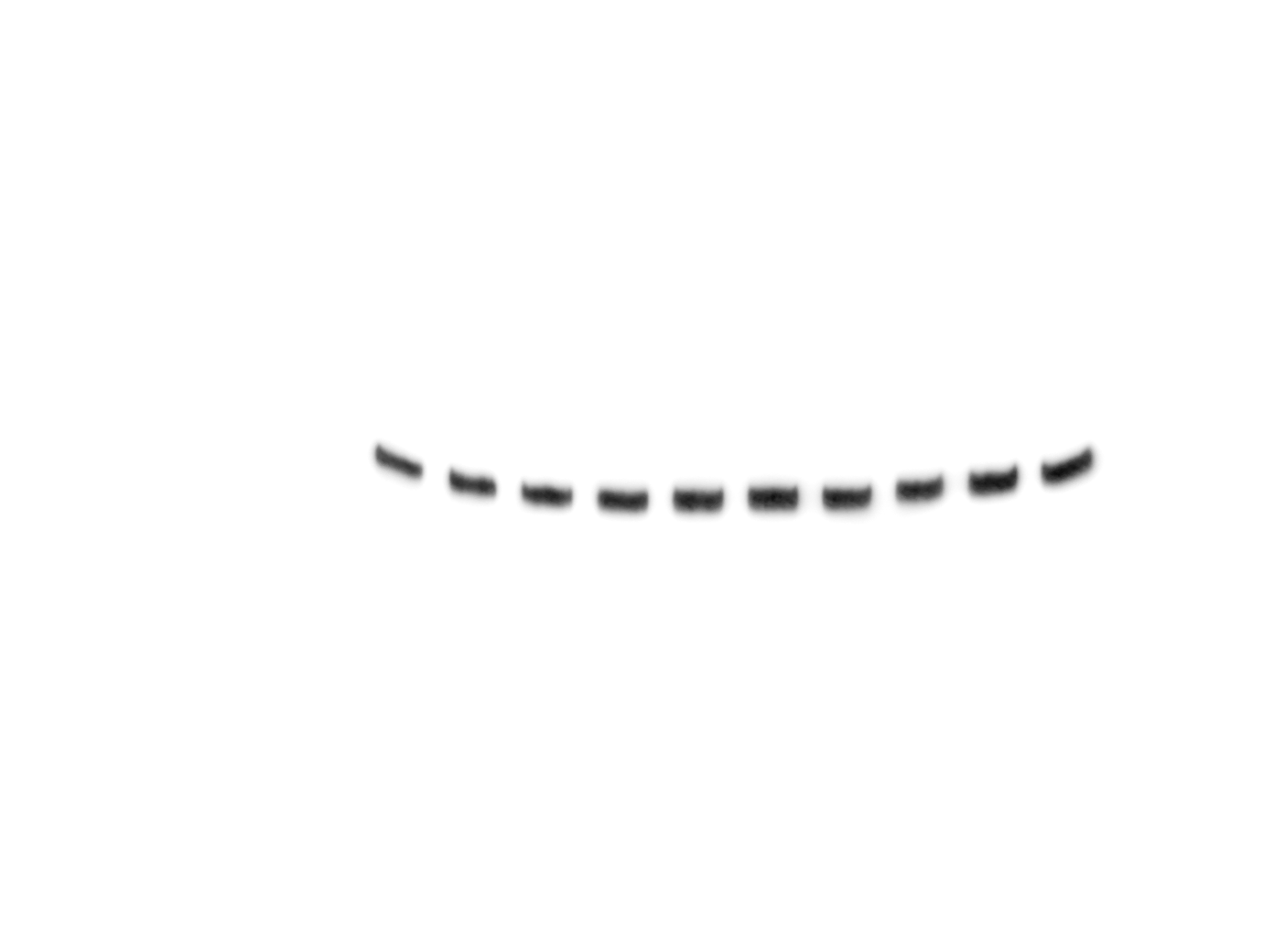

Supplement: Supplementary file 1 [file cancers-13-00862-s001.zip › WBdata_cancers/20200410_8505_E7080_aTub/20200410_8505_E7080_aTub_b.tif]

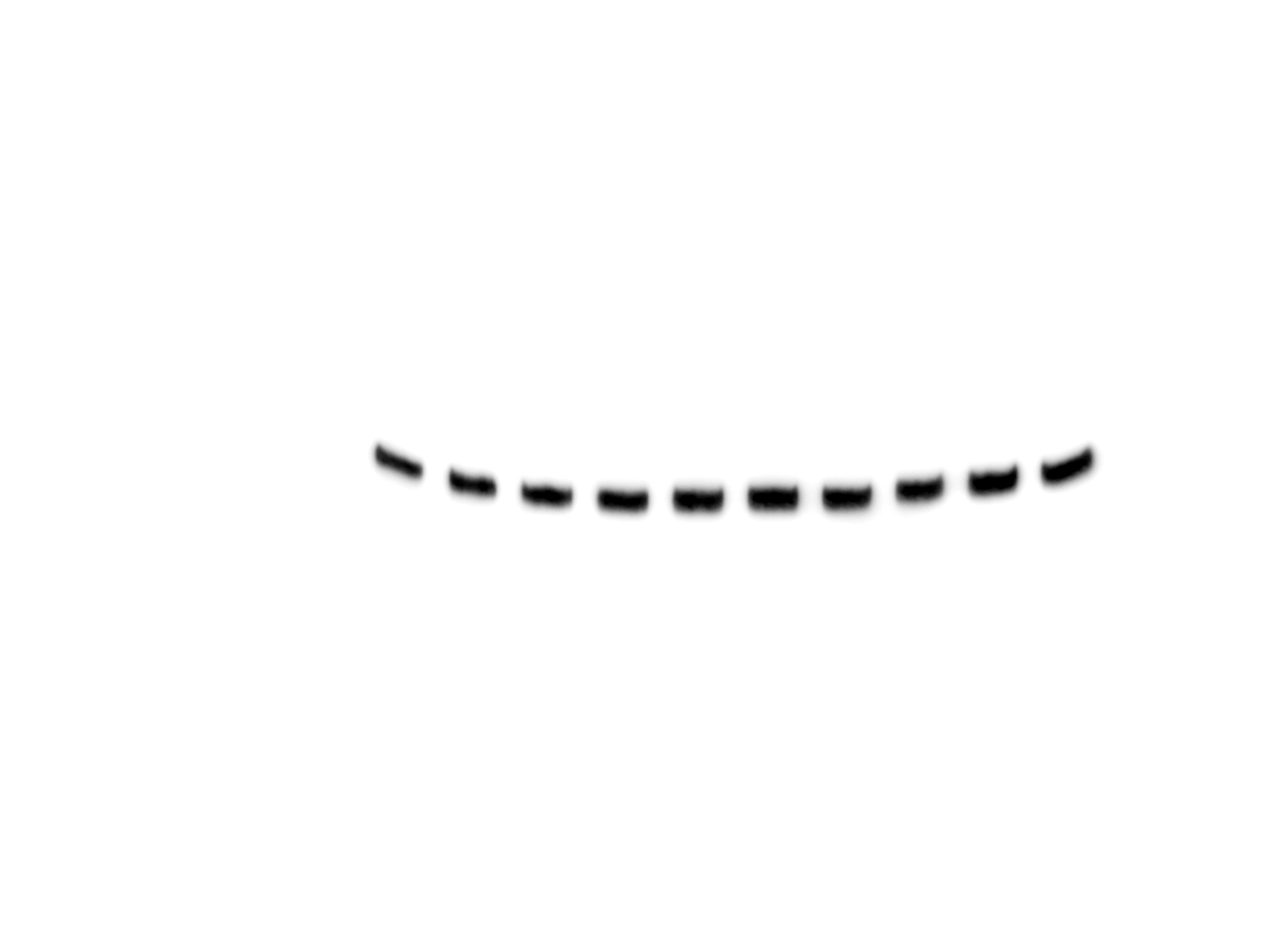

Supplement: Supplementary file 1 [file cancers-13-00862-s001.zip › WBdata_cancers/20200410_8505_E7080_aTub/20200410_8505_E7080_aTub_c.tif]

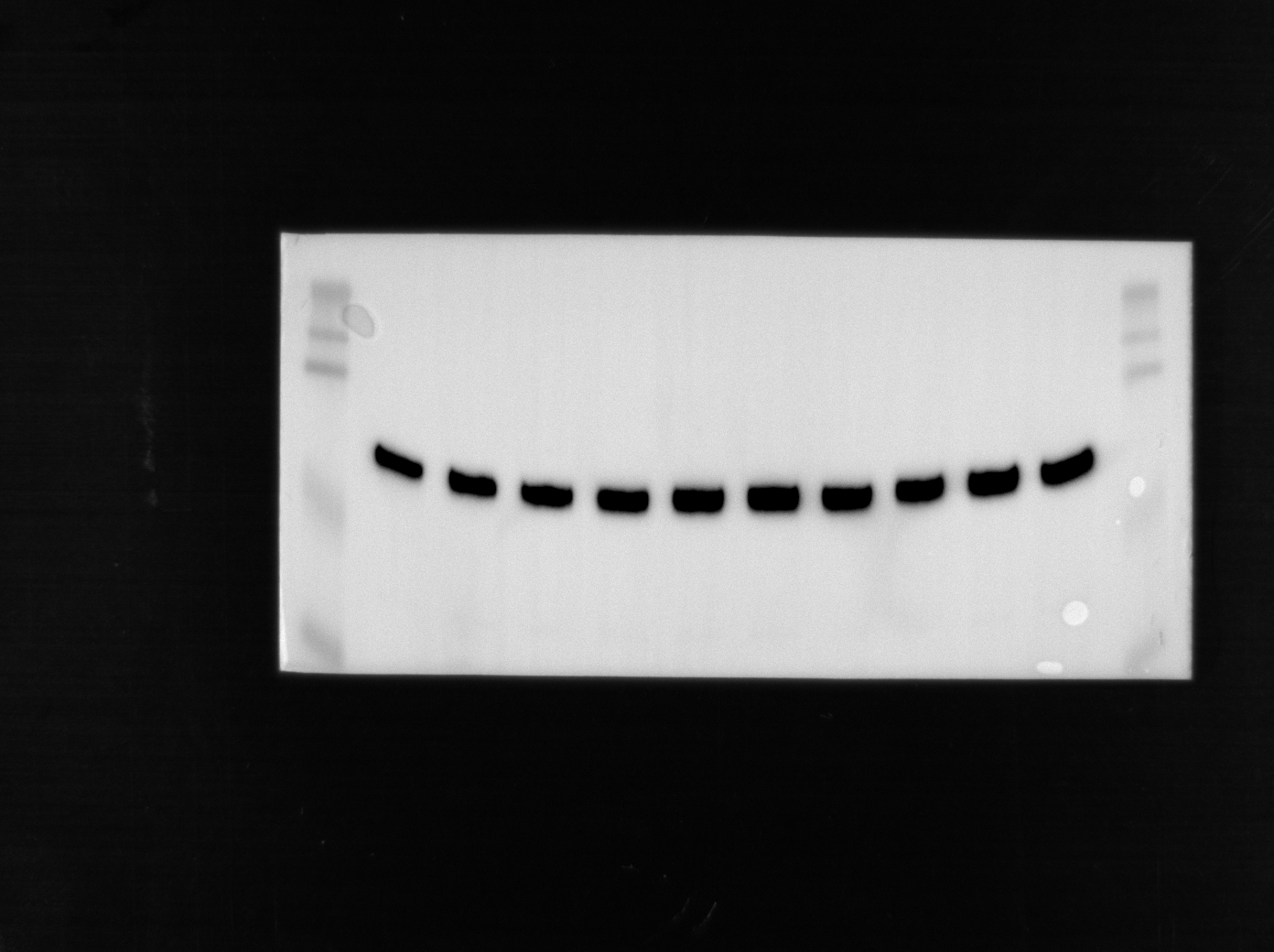

Supplement: Supplementary file 1 [file cancers-13-00862-s001.zip › WBdata_cancers/20200410_8505_E7080_aTub/20200410_8505_E7080_aTub_Merge.tif]

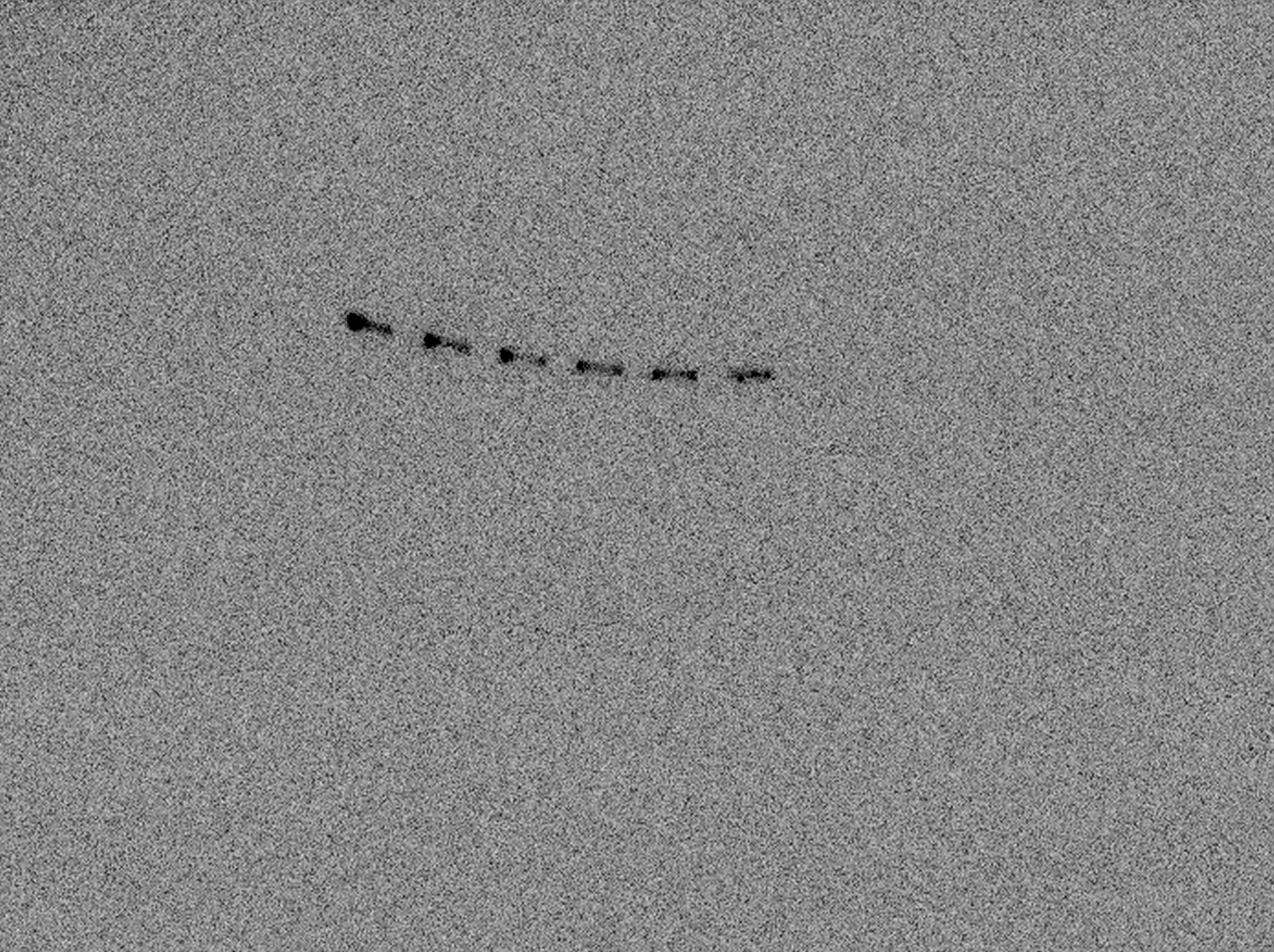

Supplement: Supplementary file 1 [file cancers-13-00862-s001.zip › WBdata_cancers/20201001_TCO1_D1_E7080/201002_TCO1_D1_E7080_a.tif]

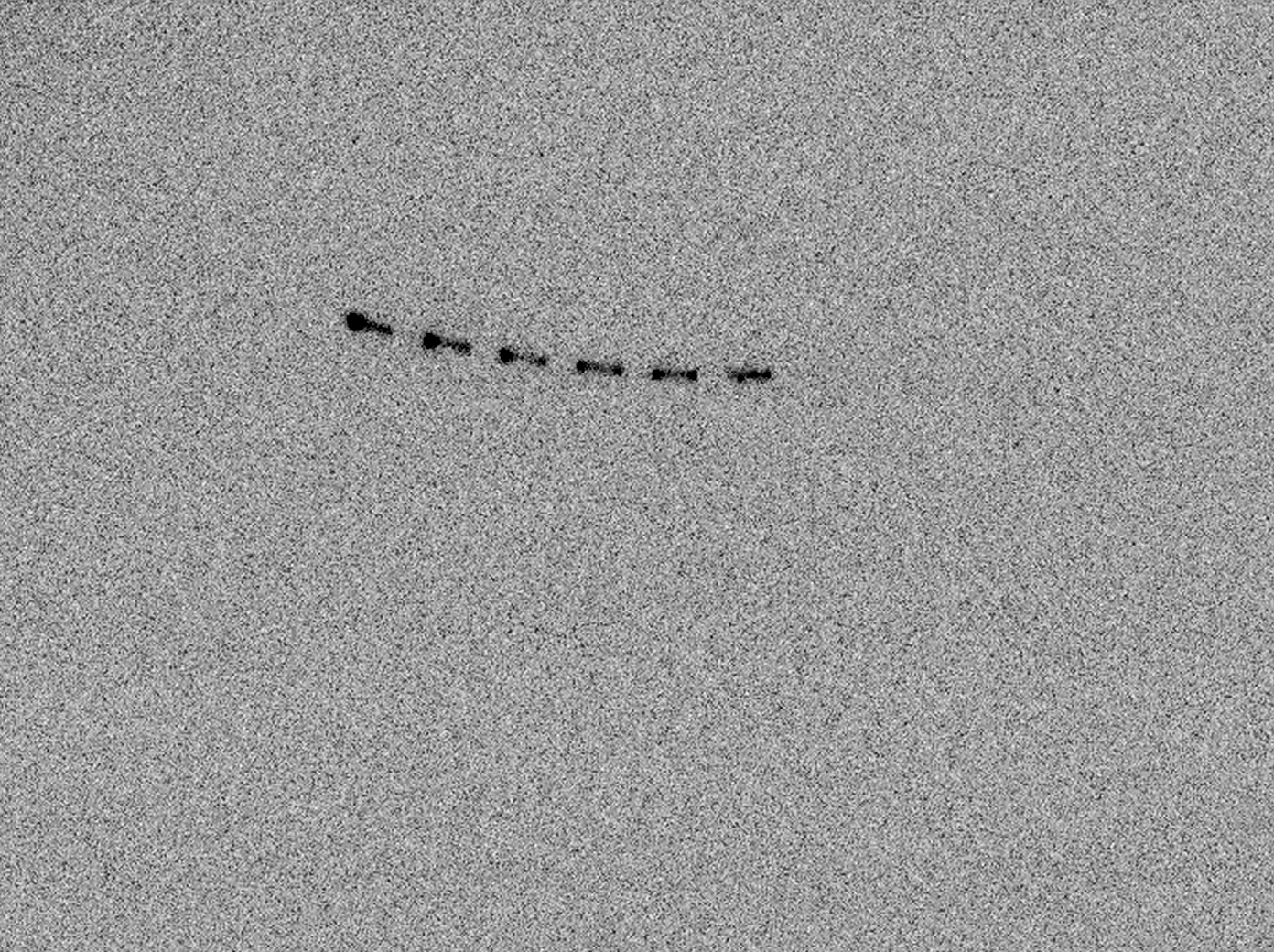

Supplement: Supplementary file 1 [file cancers-13-00862-s001.zip › WBdata_cancers/20201001_TCO1_D1_E7080/201002_TCO1_D1_E7080_b.tif]
